# Stereoselective synthesis of [2.2]triphenylenophanes *via* intramolecular double [2 + 2 + 2] cycloadditions†

**DOI:** 10.1039/d3sc00571b

**Published:** 2023-03-10

**Authors:** Yuya Kawai, Juntaro Nogami, Yuki Nagashima, Ken Tanaka

**Affiliations:** a Department of Chemical Science and Engineering, Tokyo Institute of Technology O-okayama, Meguro-ku Tokyo 152-8550 Japan ktanaka@apc.titech.ac.jp

## Abstract

Planar chiral [2.2]cyclophanes with two aromatic rings in close proximity have attracted much attention for their applications as chiral materials and catalysts because of their stable chirality and transannular interactions. Although numerous [2.2]cyclophanes have been synthesized to date, only a few polycyclic aromatic hydrocarbon (PAH)-based ones have been reported, and the simultaneous control of two planar chiralities of the two aromatic rings facing each other has not been achieved. Here we report the enantio- and/or diastereoselective synthesis of planar chiral PAH-based [2.2]cyclophanes ([2.2]triphenylenophanes) *via* the high-yielding base-mediated intermolecular macrocyclization and Rh- or Ni-catalyzed intramolecular double [2 + 2 + 2] cycloadditions. DFT calculations have revealed that the second [2 + 2 + 2] cycloaddition kinetically determines the diastereoselectivity. Single crystal X-ray diffraction analyses have confirmed that the facing triphenylene or [5]helicene skeletons strongly repel each other, resulting in curved structures with bulged centers.

## Abstract 2

Base-mediated intermolecular macrocyclization and Rh- or Ni-catalyzed intramolecular double [2 + 2 + 2] cycloadditions allow enantio- and diastereoselective synthesis of planar chiral [2.2]triphenylenophanes.

## Graphical abstract

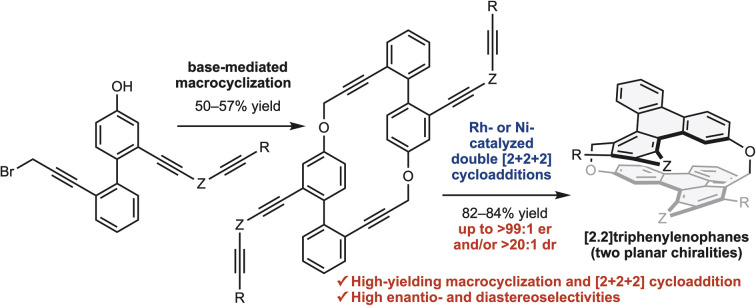

## Introduction

**Fig. 1:**
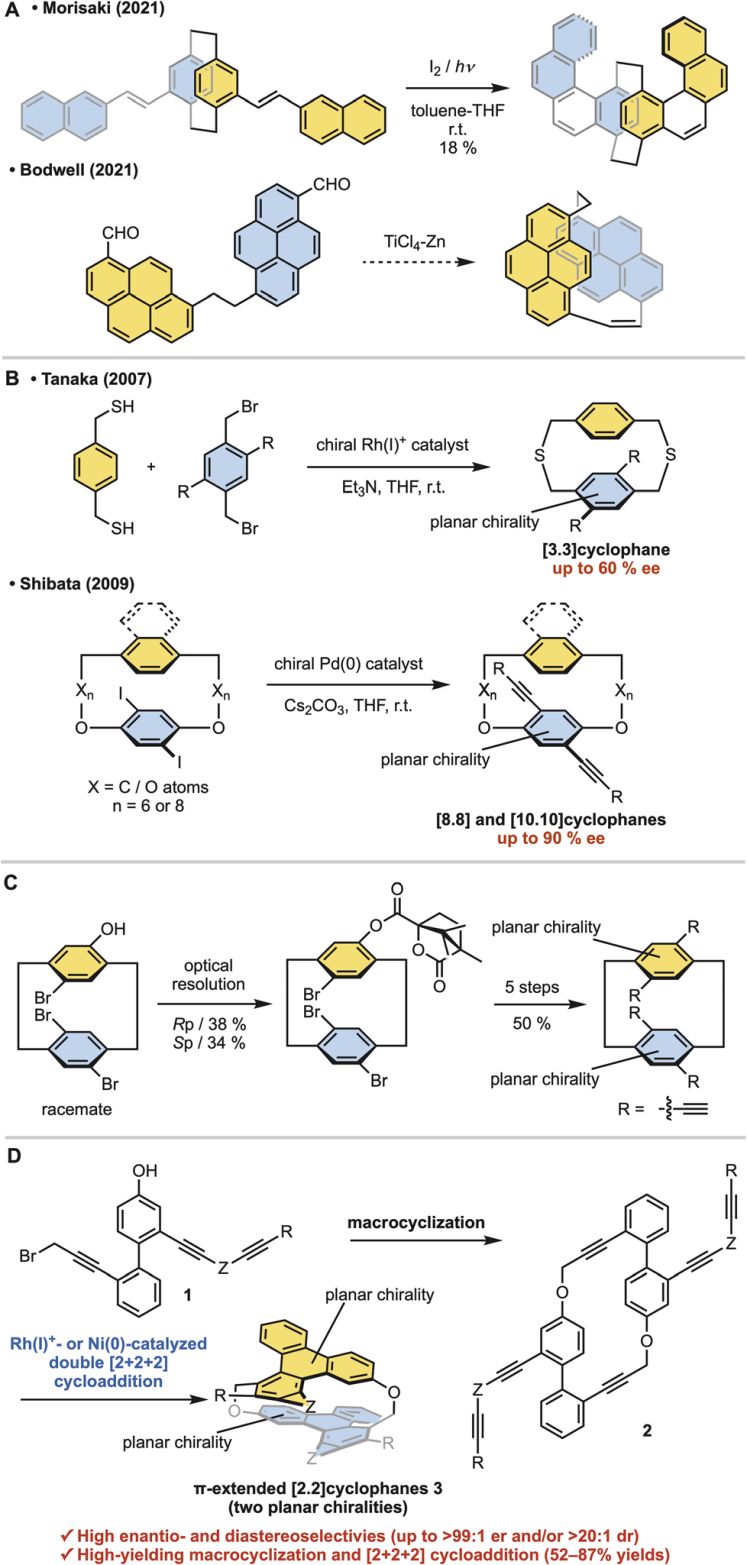
Research backgrounds. (A) Synthesis of racemic planar chiral π-extended [2.2]cyclophanes. (B) Enantioselective synthesis of [*n.n*]cyclophanes with one planar chirality. (C) Optical resolution of racemic [2.2]cyclophane with two planar chiralities (Morisaki, 2014). (D) Enantio- and diastereoselective synthesis of π-extended [2.2]cyclophanes (this work).

Cyclophanes are macrocyclic compounds with a structure in which an aromatic unit is linked into a ring by an ansa chain.^1^ In particular, cyclophanes with two stacked aromatic rings, such as [*n*.*n*]paracyclophanes, have long attracted attention due to their unique properties.^2^ In [2.2]paracyclophanes, where the tether length is two atoms, the interplanar distance is shorter than the interlayer distance of graphene.^3^ This short distance results in an electronic transannular interaction between the two aromatic rings,^4^ highly curved aromatic rings due to steric repulsion,^5^ and stable planar chirality due to rotational inhibition.^6^ Planar chiral polycyclic aromatic hydrocarbon (PAH)-based [2.2]cyclophanes are highly attractive because of their chiral nanographite-like structure.^7^ However, the linkage of two large PAHs with two-atom tethers is troublesome due to difficulty in controlling steric hindrance and stereoselectivity. Thus stepwise π-elongation syntheses from planar chiral [2.2]paracyclophane derivatives have been reported, although their structures are restricted to molecules with a [2.2]paracyclophane core (Fig. 1a, top).^8,9^ Morisaki and co-workers reported the synthesis of [2.2][4]helicenophane by oxidative photocyclization of a planar chiral stilbene-type precursor.^8^ As an exceptional example of the stereoselective direct linkage of two large PAHs with two-atom tethers, Bodwell and co-workers reported the isolation of planar chiral [2.2]pyrenophane as an unexpected side product from the intramolecular McMurry coupling of [10.2]pyrenophane-dialdehyde, presumably through the intramolecular McMurry coupling of the corresponding linear dialdehyde (Fig. 1a, bottom).^10,11^ However, this shadow reaction was not confirmed experimentally. In addition, inserting an atom with a van der Waals radius shorter than the carbon atom into the tether of the [2.2]paracyclophane is troublesome, and no synthetic examples have been reported to date.^12^

Furthermore, the asymmetric synthesis of stacked [*n*.*n*]cyclophanes is more troublesome than that of [*n*]cyclophanes. Our group^13*a*^ and the Shibata group^13*b*^ reported only two examples each of introducing planar chirality on only one side (Fig. 1b). These syntheses have moderate enantioselectivity and structural limitations that prevent shortening the tether length. Thus [*n*.*n*]cyclophanes with planar chirality on both sides have been supplied by the optical resolution of racemates. As a representative example, Morisaki and co-workers reported the optical resolution of planar chiral tetrasubstituted [2.2]paracyclophane derivatives for synthesizing a propeller-shaped macrocyclic compound, showing high fluorescence quantum efficiency and an excellent circularly polarized luminescence dissymmetry factor (Fig. 1c).^14,15^

On the other hand, Rh-catalyzed [2 + 2 + 2] cycloadditions^16–18^ allow enantioselective control of the planar chirality of [*n*]cyclophanes.^19^ Here, we report the stereoselective synthesis of oxamethylene-bridged PAHs-based [2.2]cyclophanes ([2.2]triphenylenophanes) 3*via* the base-mediated intermolecular macrocyclization of triynes 1, leading to cyclic hexaynes 2, and the subsequent Rh- or Ni-catalyzed intramolecular double [2 + 2 + 2] cycloadditions^20–22^ (Fig. 1d). The present stereoselective synthesis realizes high enantioselectivity (up to >99 : 1 er) and complete diastereoselectivity (>20 : 1 dr). Furthermore, the yields of two key reaction steps (the macrocyclization and double [2 + 2 + 2] cycloadditions) are high. Notably, the present asymmetric [2.2]cyclophane synthesis is the first example of diastereo- and enantioselective control of two planar chiralities of the [*n*.*n*]cyclophanes.

## Results and discussion

**Fig. 2:**
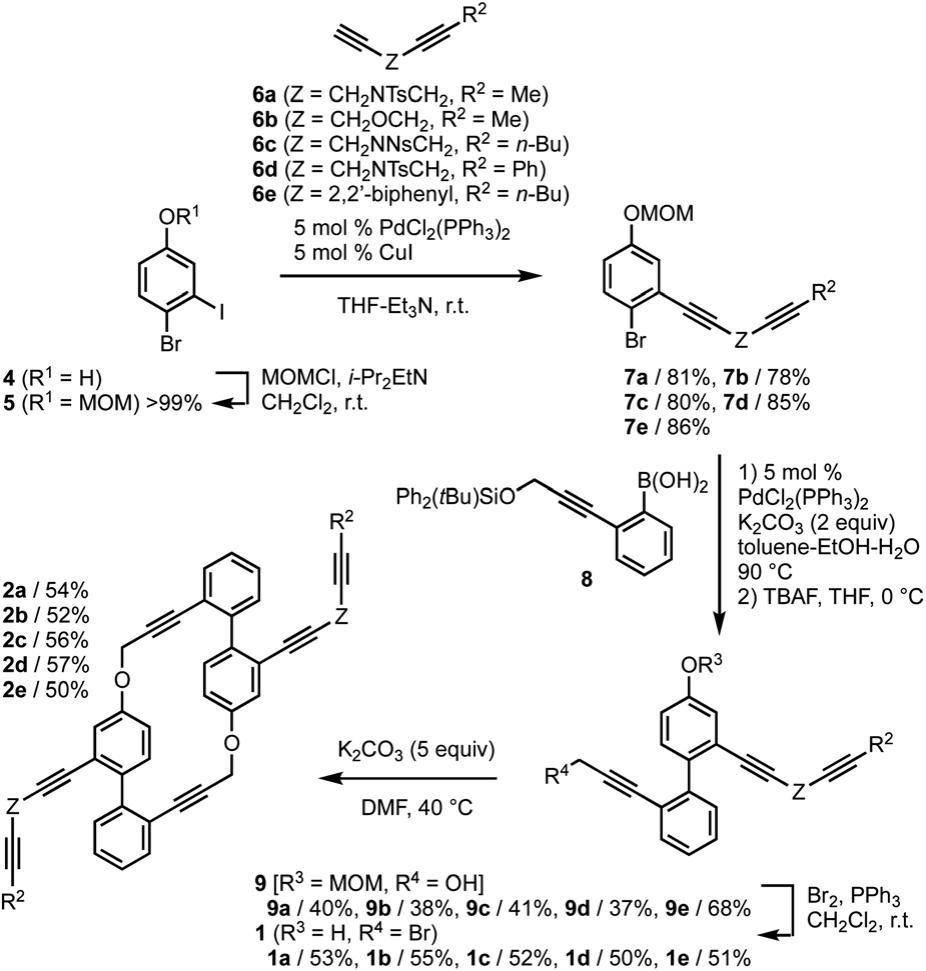
Synthesis of hexaynes 2. MOM = methoxylmethyl. Ts = *p*-toluenesulfonyl. Ns = 2-nitrobenzenesulfonyl. TBAF = tetrabutylammonium fluoride.

First, we examined the synthesis of cyclic hexaynes for the asymmetric intramolecular double [2 + 2 + 2] cycloadditions, as shown in Fig. 2. MOM-protection of 4-bromo-3-iodophenol (4) gave bromide 5, and the subsequent Sonogashira cross-coupling with diynes 6a–e gave diynes 7a–e in high yields (78–86%). The Suzuki–Miyaura cross-coupling between bromides 7a–e and boronic acid 8 followed by deprotection of the silyl protecting group by treatment with TBAF gave alcohols 9a–e in moderate yields. Treatment of 9a–e with Br_2_ and PPh_3_ afforded bromo-phenols 1a–e, in which the bromination of the propargylic alcohol and the deprotection of the MOM protecting group proceeded in one pot. Pleasingly, the base (K_2_CO_3_)-mediated intermolecular macrocyclization gave cyclic hexaynes 2a–e in good yields. Although the base-mediated intermolecular macrocyclization generally yields a mixture of dimers, trimers, and other oligomers,^20^ the ^1^H NMR analyses of the crude reaction mixtures derived from 1a–e confirmed the selective formations of the dimers (hexaynes) 2a–e in this process (Fig. S1†).

Next, we investigated the asymmetric synthesis of [2.2]triphenylenophanes (Table 1). In the presence of 80 mol% of the cationic rhodium(i)/BIPHEP complex, intramolecular double [2 + 2 + 2] cycloadditions of cyclic hexayne 2a proceeded at room temperature to give the desired [2.2]triphenylenophane 3a in a high 87% yield (entry 1, 93% yield per each [2 + 2 + 2] cycloaddition).^23^ Interestingly, the reaction proceeded in complete diastereoselectivity [dr(dl/*meso*) = >20 : 1]. We subsequently examined the enantioselective synthesis of 3a by using axially chiral biaryl bisphosphine ligands and found that enantioselectivity increases with increasing bite angle (entries 2–4, bite angle:^24^ Segphos < BINAP < H_8_-BINAP). Although the use of sterically demanding xyl-H_8_-BINAP afforded 3a in the highest enantioselectivity (>99 : 1 er), the product yield was the lowest 21% due to the undesired intermolecular [2 + 2 + 2] cycloaddition (entry 5). Reducing the catalyst loading to 20 mol% maintained the high yield and enantioselectivity (entry 6). Thus, the conditions of entry 6 were set as the optimum conditions.

**Table 1:** Double [2 + 2 + 2] cycloadditions of hexaynes 2 leading to [2.2]triphenylenophanes 3

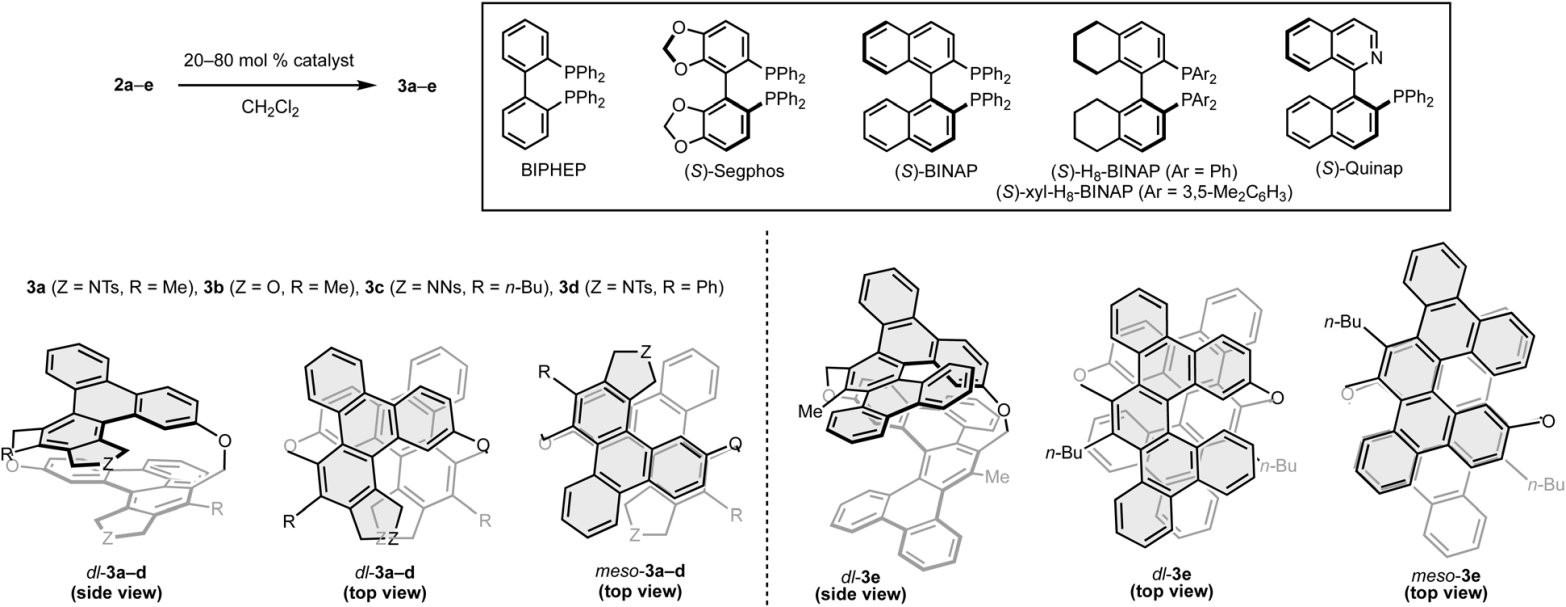					
Entry	2	Catalyst	Catalyst (mol%)	Temp, time	3/% yield^a^ (dl/*meso*, er)
1	2a	[Rh(cod)_2_]BF_4_/BIPHEP	80	r.t, 3 h	3a/87 (>20 : 1)
2	2a	[Rh(cod)_2_]BF_4_/(*S*)-segphos	80	r.t, 3 h	(+)-3a/82 (>20 : 1, 84 : 16)
3	2a	[Rh(cod)_2_]BF_4_/(*S*)-BINAP	80	r.t, 3 h	(+)-3a/84 (>20 : 1, 88 : 12)
4	2a	[Rh(cod)_2_]BF_4_/(*S*)-H_8_-BINAP	80	r.t, 3 h	(+)-3a/80 (>20 : 1, 93 : 7)
5	2a	[Rh(cod)_2_]BF_4_/(*S*)-xyl-H_8_-BINAP	80	r.t, 3 h	(+)-3a/21 (>20 : 1, >99 : 1)
6	2a	[Rh(cod)_2_]BF_4_/(*S*)-H_8_-BINAP	20	r.t, 3 h	(+)-3a/84 (>20 : 1, 95 : 5)
7	2b	[Rh(cod)_2_]BF_4_/(*S*)-H_8_-BINAP	20	r.t, 3 h	(*R*p,*R*p)-(+)-3b/82 (>20 : 1, 93 : 7)
8	2c	[Rh(cod)_2_]BF_4_/(*S*)-H_8_-BINAP	20	r.t, 3 h	(+)-3c/82 (>20 : 1, 94 : 6)
9	2d	[Rh(cod)_2_]BF_4_/(*S*)-H_8_-BINAP	20	r.t, 3 h	3d/insoluble
10	2e	[Rh(cod)_2_]BF_4_/(*S*)-H_8_-BINAP	20	r.t, 3 h	No reaction
11^b^	2e	[Rh(cod)_2_]BF_4_/(*S*)-H_8_-BINAP	20	60 °C, 16 h	Complex mixture
12	2e	Ni(cod)_2_/2PPh_3_	20	40 °C, 16 h	3e/83 (>20 : 1)
13	2e	Ni(cod)_2_/(*S*)-Quinap	20	40 °C, 16 h	Complex mixture

a Isolated yield.

b (CH_2_Cl)_2_ was used instead of CH_2_Cl_2_.

The yields, enantioselectivity, and diastereoselectivity of the products 3b and 3c were all favorable when the linker moiety of the 1,6-diyne side chain was changed from NTs to O and NNs (2b and 2c, entries 7 and 8). However, in the reaction of hexayne 2d with a phenyl group at the terminus of the 1,6-diyne side chain, 2d disappeared completely, but a solid with extremely low solubility was formed (entry 9). We also investigated the synthesis of [2.2][5]helicenophane 3e, which is further π-extended from [2.2]triphenylenophanes, by replacing the 1,6-diyne side chain with a biphenyl-linked 1,7-diyne one (2e). Although we carried out the reaction of hexayne 2e under the above optimal conditions, no conversion of 2e was observed (entry 10). At an elevated reaction temperature of 60 °C, a complete conversion of 2e was observed, but the desired product 3e was not generated at all (entry 11). Pleasingly, the desired intramolecular double [2 + 2 + 2] cycloadditions of 2e proceeded at 40 °C in a high 83% yield and complete diastereoselectivity by using the nickel(0)/PPh_3_ complex (entry 12).^25^ Stáry and co-workers reported that Quinap is a highly effective chiral ligand for the enantioselective synthesis of carbohelicenes by the nickel(0)-catalyzed intramolecular [2 + 2 + 2] cycloaddition.^26^ Thus, we also investigated the enantioselective synthesis of 3e using Quinap as a chiral ligand, but a complex mixture of products was generated (entry 13).

**Fig. 3:**
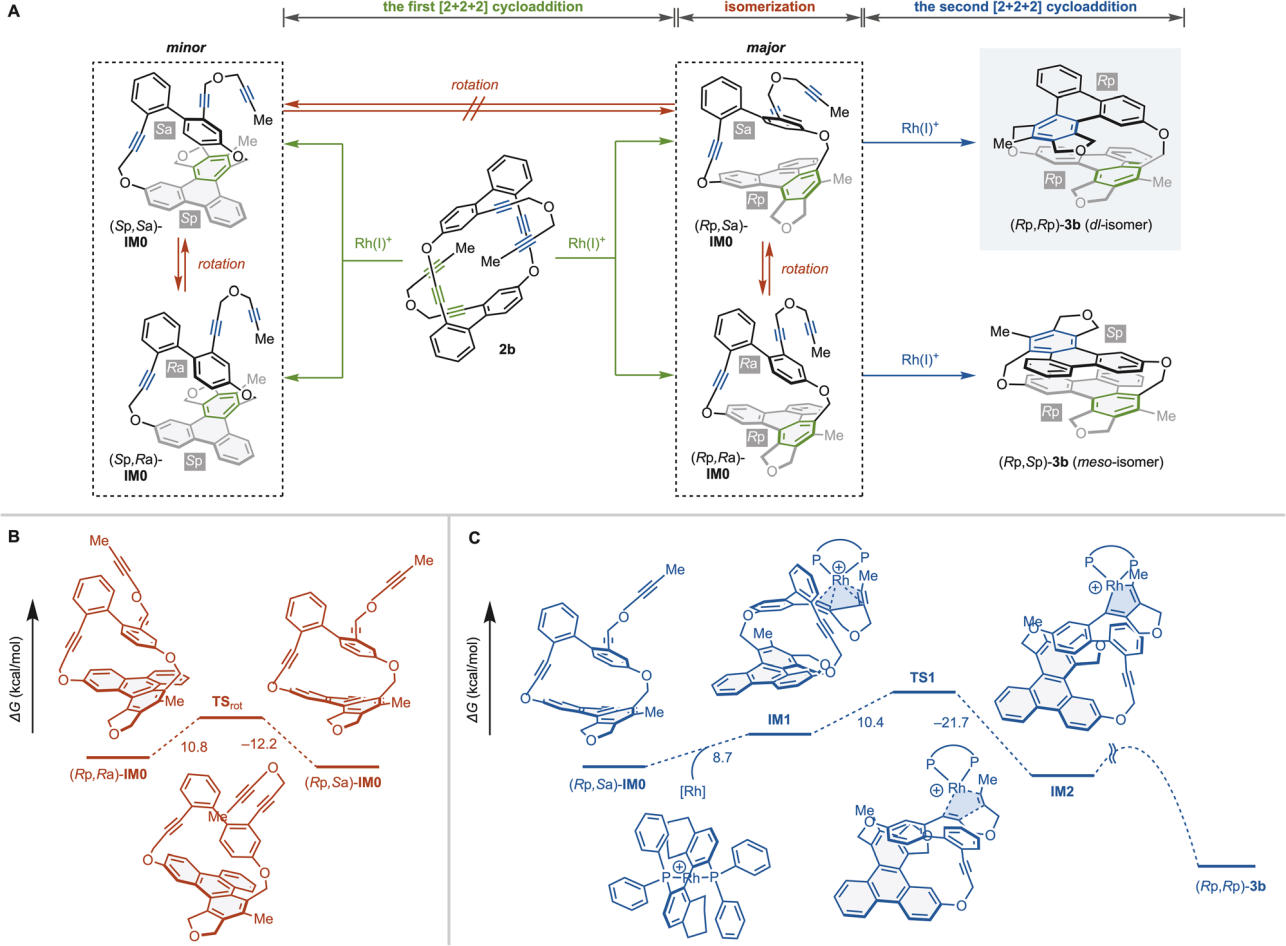
Analyses for steps determining enantio- and diastereoselectivities to produce (*R*p,*R*p)-3b from 2b. The changes of the Gibbs free energies (kcal mol^−1^) are calculated at the M06/6-31G(d) (C, H, O, P) and LANL2DZ (Rh) level. (A) Reaction pathways of Rh-catalyzed [2 + 2 + 2] cycloaddition of **2b** leading to **3b**. (B) Rotation isomerization of axial chirality. (C) The second [2 + 2 + 2] cycloaddition to produce (*R*p,*R*p)-**3b**.

The above intramolecular double [2 + 2 + 2] cycloaddition reactions yield cyclophanes 3 with high enantioselectivity and complete diastereoselectivity. Thus, we analyzed the steps in which stereoselectivities are determined using cyclic hexayne 2b and (*S*)-H_8_-BINAP as a substrate and ligand, respectively (Fig. 3). The first [2 + 2 + 2] cycloaddition of 2b produces four possible intermediates (*R*p,*S*a)-IM0, (*R*p,*R*a)-IM0, (*S*p,*R*a)-IM0, and (*S*p,*S*a)-IM0 with one planar chiral triphenylene moiety and one axially chiral biphenyl moiety (Fig. 3a). The triphenylene moieties of IM0 are sufficiently large relative to the tether length that those are non-rotatable at room temperature,^19*c*–*f*^ and thus the first [2 + 2 + 2] cycloaddition would enantioselectively determine the planar chirality, producing (*R*p,*S*a)-IM0 and (*R*p,*R*a)-IM0.

The step determining the diastereoselectivity may be the rotational isomerization of the axial chirality or the first or second [2 + 2 + 2] cycloaddition (Fig. 3a). We performed DFT calculations at the M06/6-31G(d)&LANL2DZ level to elucidate which step determines the diastereoselectivity. DFT calculations estimate the activation energies of the biphenyl rotation to be Δ*G*^‡^_TSrot-(*R*p,*R*a)-IM0_ = 10.8 kcal mol^−1^ and Δ*G*^‡^_TSrot-(*R*p,*S*a)-IM0_ = 12.2 kcal mol^−1^, indicating that the rotational isomerization proceeds rapidly even at room temperature (Fig. 3b). We then explored the reaction pathway of the second [2 + 2 + 2] cycloaddition of (*R*p,*S*a)-IM0 leading to (*R*p,*R*p)-3b (Fig. 3c).^27,28^ We found that the activation energy for the rate-limiting five-membered rodacycle formation [(*R*p,*S*a)-IM0 → IM1 → TS1, Δ*G*^‡^ = 19.1 kcal mol^−1^] is more than 6 kcal mol^−1^ larger than the rotational isomerization between (*R*p,*S*a)-IM0 and (*R*p,*R*a)-IM0. Thus we can conclude that the second [2 + 2 + 2] cycloaddition kinetically determines the diastereoselectivity.

**Fig. 4:**
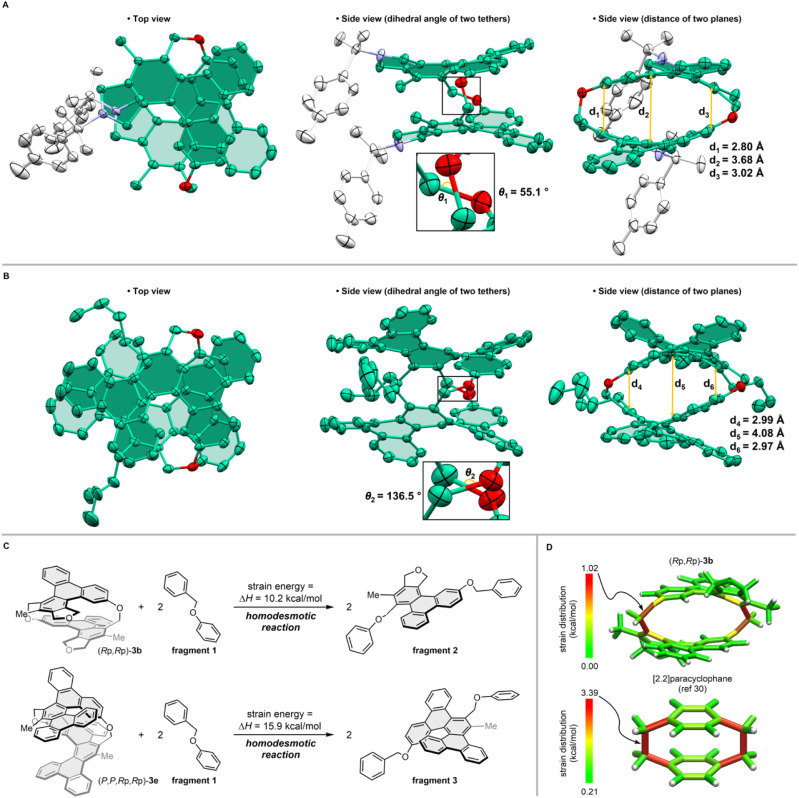
X-ray crystal structures (a and b) and strain analyses (c and d). Hydrogen atoms are omitted for clarity. Atoms and the Ts group are colored green (carbon), red (oxygen), purple (nitrogen), and white (Ts group). (A) Unimolecular structures of [2.2]triphenylenophane (±)-3a. (B) Unimolecular structures of [2.2][5]helicenophane (±)-3e. (C) Strain energies of [2.2]triphenylenophane 3b and [2.2][5]helicenophane 3e. (D) StrainViz of 3b and [2.2]paracyclophane.

We performed single-crystal X-ray diffraction analyses of [2.2]triphenylenophane (±)-3a (Fig. 4a) and [2.2][5]helicenophane (±)-3e (Fig. 4b).^29^ Viewed from the side, the facing triphenylenes and [5]helicenes strongly repel each other intramolecularly, forming curved structures with bulges in their center. Measuring the carbon–carbon distance between the two planes of [2.2]triphenylenophane (±)-3a revealed that although the shortest interplane distance (2.80 Å) is close to the value of [2.2]paracyclophane with the same diatomic bridge (2.83 Å), the longest interplane distance in the middle (3.68 Å) is considerably longer than that of [2.2]paracyclophane (3.09 Å). This central curvature is even more pronounced in [2.2][5]helicenophane (±)-3e, where the longest interplane distance is 4.08 Å. The top views of (±)-3a and (±)-3e show that the two oxamethylene tether chains are twisted rather than parallel as in [2.2]paracyclophane, with a larger twist angle in (±)-3e (136.5°) than in (±)-3a (55.1°). This large twist in (±)-3e may be due to the helical structure of the two [5]helicenes twisting and overlapping each other so that they do not repel each other. The helical chirality of the two [5]helicenes in 3e was identical, and they were tethered together to form a long helicene-like structure. The dihedral angles of the two [5]helicenes were 51.9° and 55.0°, slightly smaller than the 56.2° of dibenzo[*f*,*j*]picene^30^ (Fig. S5†).

We further evaluated the strain energies by DFT calculations at the B3LYP/6-31G(d) level. The strain energy calculated based on the homodesmotic reaction method^31^ was greater for [2.2][5]heliceno-phane 3e (15.9 kcal mol^−1^) than for [2.2]triphenylenophane 3b (10.2 kcal mol^−1^) (Fig. 4c). The strain distribution analysis using StrainViz^32^ showed that the strain of [2.2]triphenylenophane 3b was unevenly distributed at the tethered moieties (Fig. 4d). These cyclophanes have smaller strain than [2.2]paracyclophane,^33^ which may be due to strain dispersion by π-extension and increased flexibility of the tether by introducing oxygen atoms.

**Fig. 5:**
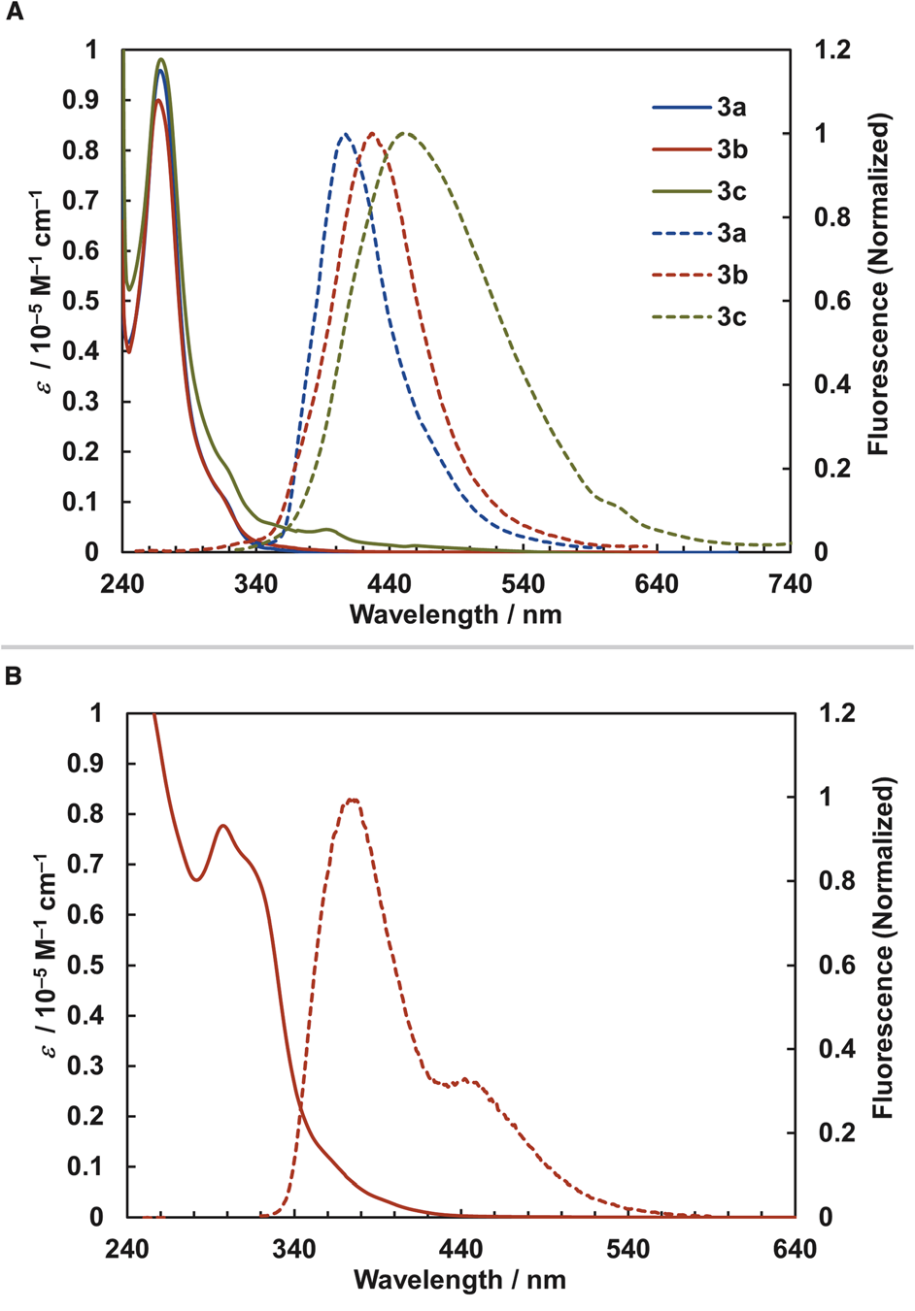
Absorption (solid line) and emission (dashed line) spectra. (A) 3a–3c. (B) 3e

We examined the photophysical properties of [2.2]triphenylenophanes 3a–c (Fig. 5a) and [2.2][5]helicenophane 3e (Fig. 5b) and summarized the data in Table 2. The UV-vis absorption spectra of [2.2]triphenylenophanes 3a–c showed absorption maxima at 267–269 nm, with little change due to substituent differences. On the other hand, the fluorescence spectra showed emission maxima at 406–450 nm, with a pronounced red shift and broadening in the order of 3a, 3b, and 3c. For [2.2][5]helicenophane 3e, the absorption maximum was 297 nm on the longer wavelength compared to 3a–c, resulting in a smaller Stokes shift. This phenomenon may be due to the higher molecular rigidity of helicene than triphenylene, which suppresses the structural relaxation of excited states.^34^ Regarding fluorescence quantum yields, [2.2]triphenylenophanes 3a–c showed very small values (0.6–2.1%) similar to that of [2.2]paracyclophanes without additional chromophores,^35^ much smaller than that of unsubstituted triphenylene(9%).^36^ In contrast, the fluorescence quantum yield of [2.2][5]helicenophane 3e was 12.5%, a significant increase over the 4% of [5]helicene.^37^

**Table 2:** Photophysical and chiroptical data^a^

Compd	Absorption *λ*_max_/nm	Emission *λ*_max_/nm (excitation wavelength/nm)	*Φ*_F_ (excitation wavelength/nm)	[*α*]_D_^b^ (ref. 25)	*g*_abs_ c (wavelength/nm)
(+)-3a	268	406 (310)	0.011 (290)	289	1.9 × 10^−3^ (325)
(+)-3b	267	427 (220)	0.021 (340)	255	1.6 × 10^−3^ (325)
(+)-3c	269	450 (305)	0.006 (300)	246	1.5 × 10^−3^ (331)
(+)-3e	297, 319	375, 446 (300)	0.125 (300)	940	3.9 × 10^−3^ (353)

a In CH_2_Cl_2_ (1.0 × 10^−5^ M) at 25 °C.

b In CHCl_3_ at 25 °C.

c In CHCl_3_ (1.0 × 10^−5^ M) at 25 °C.

**Fig. 6:**
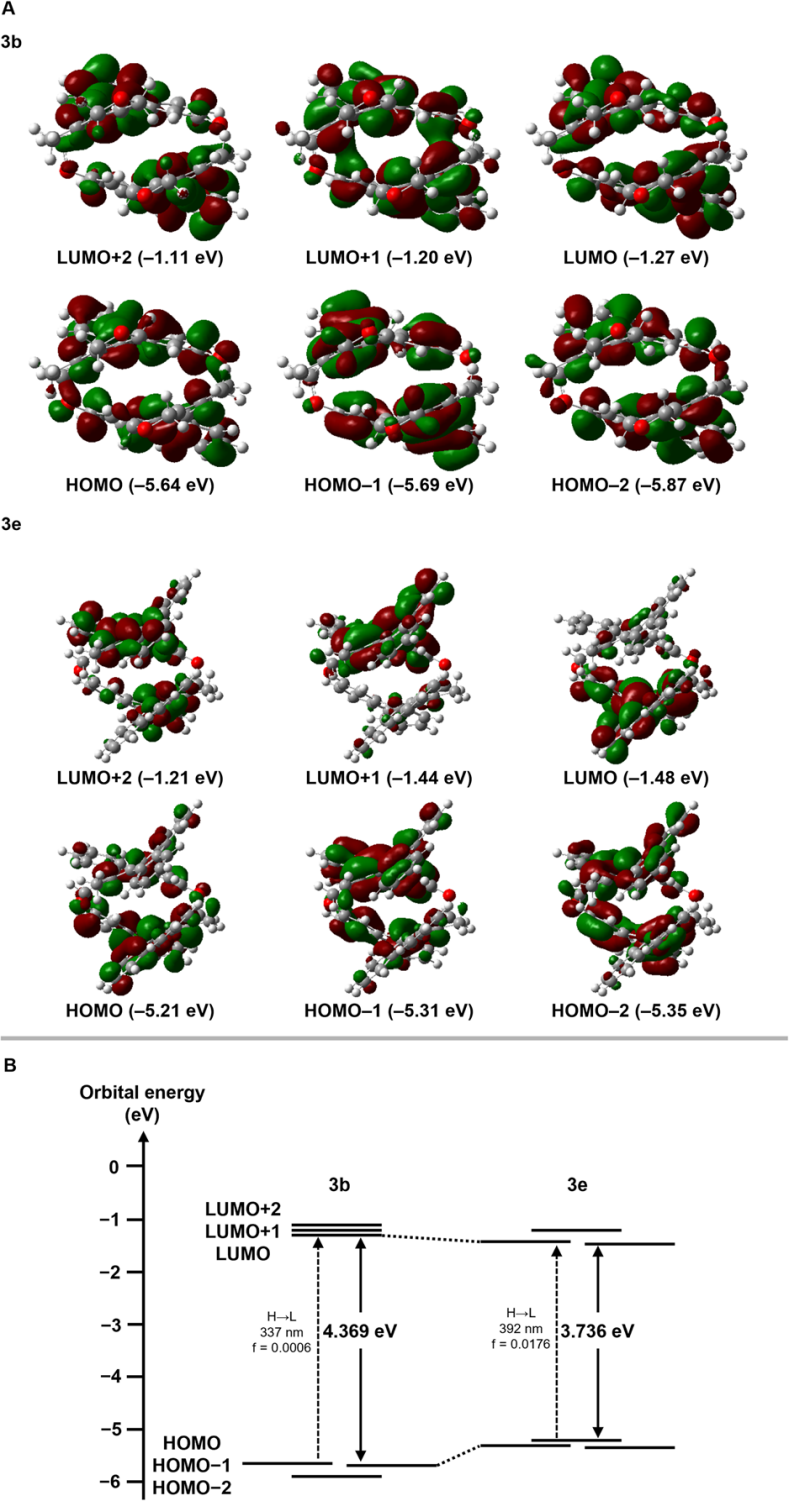
Electronic structures calculated by TD-DFT at the B3LYP/6-31G(d) level. (A) Pictorial representations of six frontier MOs of 3b and 3e. (B) Energy diagrams of 3b and 3e.

To understand the optical properties described above, we examined the electronic properties of [2.2]triphenylenophane 3b and [2.2][5]helicenophane 3e by DFT and TD-DFT calculations at the B3LYP/6-31G(d) level. The frontier orbitals of 3b and 3e are delocalized and distributed throughout the molecule (Fig. 6a). For the transannular interactions between the two aromatic systems, an overlap of molecular orbitals was observed in the LUMO+1 orbital of 3b. In contrast, no overlap was observed in any orbitals of 3e. The energy diagrams of 3b and 3e are shown in Fig. 6b. The energy gap between the HOMO and LUMO orbitals is significantly narrower in 3e (3.736 eV) than in 3b (4.369 eV), supporting the experimental result that 3e has absorption maxima at longer wavelengths than 3b. The diagram reveals that decreasing the energy gap is mainly due to increasing occupied orbitals. This phenomenon may be due to the destabilization of the occupied orbitals of 3e, which has a greater degree of curvature than 3b. This structural dependence is also observed in cyclic π-conjugated compounds such as cycloparaphenylenes.^38^ A comparison of the oscillator strength values of the interorbital transitions determined by TD-DFT calculations suggests that the HOMO → LUMO transition is almost forbidden for 3a (*f* = 0.0006), whereas it is weak but permissible for 3e (*f* = 0.0176).

**Fig. 7:**
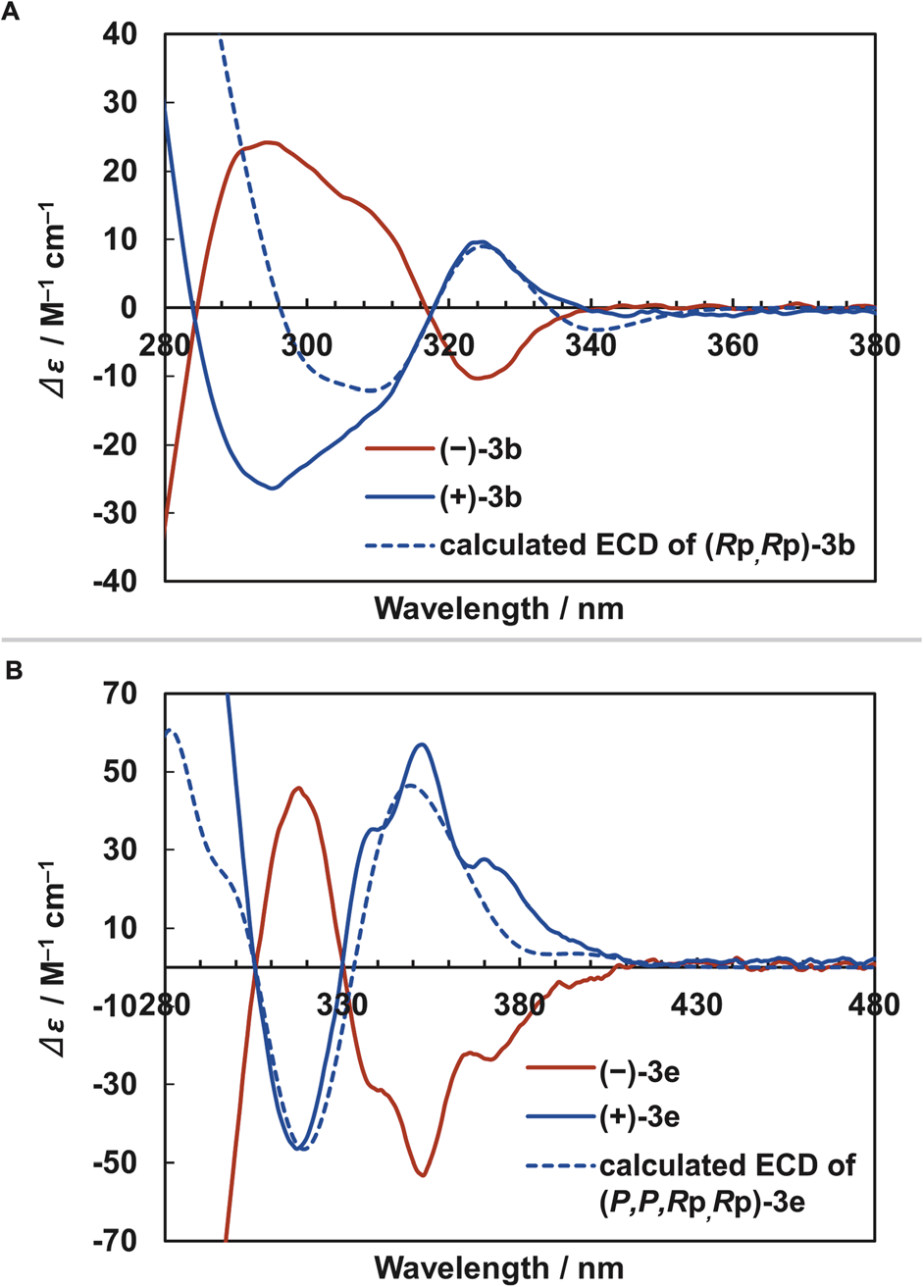
ECD spectra. Dashed blue lines show ECD spectra calculated by TD-DFT at the B3LYP/6-31G(d) level. (A) (–)-3b, (+)-3b, and calculated (*R*p,*R*p)-3b. (B) (–)-3e, (+)-3e, and calculated (*P*,*P*,*R*p,*R*p)-3e.

Finally, we evaluated the chiroptical properties of [2.2]triphenylenophanes 3a–c. The optical rotation values of 3a–c and electronic circular dichroism (ECD) spectra of 3b are shown in Table 2 and Fig. 7a, respectively. The optical rotation values of 3a–c were 246–289°, larger than the planar chiral non-π-extended [2.2]paracyclophane.^39^ The ECD spectra of 3b show a mirror image based on the distinct Cotton effect, and the dissymmetry factor *g*_abs_ is moderate with a maximum value of 1.9 × 10^−3^. This value is comparable to the planar chiral non-π-extended [2.2]paracyclophane.^3*d*,40^ Enantiopure [2.2][5]helicenophanes (+)-3e and (−)-3e were also obtained by optical resolution of (±)-3e using chiral HPLC (Daicel CHIRALPAK IE). The optical rotation value of 3e (940°) was significantly larger than those of 3a–c and slightly larger than that of [2.2][4]helicenophane (878°) (Table 2).^8^ The ECD spectra of (+)-3e and (−)-3e exhibited a mirror-image relationship (Fig. 7b). Similar to the optical rotation values, the dissymmetry factor *g*_abs_ (3.9 × 10^−3^) was significantly larger than those of 3a–c. The signs of the Cotton effects in the ECD spectra of (*R*p,*R*p)-3b and (*P*,*P*,*R*p,*R*p)-3e determined by the DFT calculations at the B3LYP/6-31G(d) level are in good agreement with the experimental spectra of (+)-3b and (+)-3e, and thus the absolute conformations of (+)-3b and (+)-3e was determined to be the *R*p,*R*p and *P*,*P*,*R*p,*R*p configurations, respectively.

## Conclusions

In conclusion, we have succeeded in the diastereo- and enantioselective synthesis of the planar chiral [2.2] triphenylenophanes *via* the base-mediated intermolecular macrocyclization followed by the Rh-catalyzed asymmetric intramolecular double [2 + 2 + 2] cycloaddition. This asymmetric [2.2]cyclophane synthesis is the first example of diastereo- and enantioselective control of two planar chiralities of the [*n*.*n*]cyclophanes. We have also succeeded in the diastereoselective synthesis of planar chiral [2.2][5]helicenophane by the Ni-catalyzed intramolecular double [2 + 2 + 2] cycloaddition. The DFT calculations revealed that the diastereoselectivity of these reactions is kinetically determined by the second [2 + 2 + 2] cycloaddition rather than by rotational isomerization of the axial chirality of the biaryl intermediate produced in the first [2 + 2 + 2] cycloaddition. Single crystal X-ray diffraction analyses showed that the facing triphenylene skeletons strongly repel each other, leading to a curved structure with a bulged central part. Regarding photophysical properties, although fluorescence quantum yields of [2.2]triphenylenophanes (0.6–2.1%) are much smaller than that of unsubstituted triphenylene (9%), that of [2.2][5]helicenophane (12.5%) is much larger than that of [5]helicene (4%). Regarding the transannular interactions between the two aromatic rings, an overlap of molecular orbitals was observed in the [2.2]triphenylenophane, but no overlap was observed in the [2.2][5]helicenophane. Regarding chiroptical properties, the ECD spectra of the [2.2]triphenylenophane and the [2.2][5]helicenophane showed mirror images based on the distinct Cotton effects, and their dissymmetry factors *g*_abs_ are relatively good with maximum values of 1.9 × 10^−3^ for the [2.2]triphenylenophane and 3.9 × 10^−3^ for the [2.2][5]helicenophane. This study demonstrates that the transition-metal-catalyzed intramolecular double [2 + 2 + 2] cycloaddition is an excellent method for the stereoselective synthesis of PAHs-based [2.2]cyclophanes.

## Data availability

Experimental procedures, spectral and crystal data, theoretical calculations, and characterization data for all new compounds are provided in the ESI.† Crystallographic data for compounds (±)-3a (CCDC 2237885) and (±)-3e (CCDC 2237886) have been deposited in the Cambridge Crystallographic Data Centre.

## Author contributions

Y. K. designed the project and carried out experimental works. J. N. carried out X-ray crystal structure analyses and performed computational studies under the guidance of Y. N. Y. K. wrote a draft under the guidance of J. N. and Y. N. K. T. designed, advised, and directed the project and wrote the manuscript.

## Conflicts of interest

There are no conflicts to declare.

## Supplementary Material

SC-014-D3SC00571B-s001

SC-014-D3SC00571B-s002
