# Supplementary material for: Stereoselective synthesis of [2.2]triphenylenophanes via intramolecular double [2 + 2 + 2] cycloadditions
Source: Chem Sci. 2023 Mar 10;14(15):3963–72. doi: 10.1039/d3sc00571b (PMC10094470; doi:10.1039/d3sc00571b)

Electronic Supplementary Material (ESI) for Chemical Science.
This journal is © The Royal Society of Chemistry 2023

Table of Contents

1. **General** **S2**

2. **Synthetic Experiments** **S3**

3. **ECD Spectra** **S22**

4. **Crystal Data** **S23**

5. **Theoretical Calculations** **S26**

6. **References** **S37**

7. **Chiral HPLC Charts** **S38**

8. **^{1}H and ^{13}C NMR Spectra of New Compounds** **S43**

1. General

Dry degassed Et_3N (Et_3N, No. T0424) for the synthesis of **7a–d** was obtained from TCI Chemicals and used as received. Dry-degassed THF (No. 209-18705), CH_2Cl_2 (No. 041-32345), and DMF (No. 044-32075) for the synthesis of **2a–e** and **3a–e** were obtained from Wako Pure Chemical Industries and used as received. THF for the synthesis of **6e**, **S8**, **8**, and **9a–e**, DMF for the synthesis of **2a–e**, CH_2Cl_2 for the synthesis of **1a–e** and **5**, THF for the synthesis of **S8**, CH_3CN for the synthesis of **6c**, toluene for the synthesis of **S5** and **S9–13**, and EtOH for the synthesis of **S5** and **S9–13** were dried over Molecular Sieves 4A. $[Rh(cod)_2]BF_4$ was obtained from Umicore AG. Segphos, H_8-binap, and tol-binap were obtained from Takasago International Corporation. 4-Bromo-3-iodophenol (**4**),[1] 4-nitro-*N*-(prop-2-yn-1-yl)benzenesulfonamide (**S1**),[2] hept-2-yn-1-yl 4-methylbenzenesulfonate (**S2**),[3] 1-bromo-2-(hex-1-yn-1-yl)benzene (**S3**),[4] (2-(((triisopropylsilyl)oxy)ethynyl)phenyl)boronic acid (**S4**),[5] *tert*-butyldiphenyl(prop-2-yn-1-yloxy)silane (**S7**),[6] *N*-(but-2-yn-1-yl)-4-methyl-*N*-(prop-2-yn-1-yl)benzenesulfonamide (**6a**),[7] 1-(prop-2-yn-1-yloxy)but-2-yne (**6b**),[8] and 4-methyl-*N*-(3-phenylprop-2-yn-1-yl)-*N*-(prop-2-yn-1-yl)benzenesulfonamide (**6d**)[9] were prepared according to the literature. Commercially available reagents were purchased from TCI Chemicals, Wako Pure Chemical Industries, Sigma-Aldrich, and Kanto Chemicals and used as received unless otherwise noted. Silica gel column chromatography was performed using silica gel [Silica Gel 60 N (spherical, neutral), Kanto Chemicals] and JIS (Japanese Industrial Standards) special-grade solvents. Silica gel preparative thin-layer chromatography (PTLC) was performed using silica gel (Wakogel® B-5F) and JIS special-grade solvents. Isolation by PTLC was performed by dissolving crude products in dichloromethane and applying it to PTLC, and re-extracted from silica gel using ethyl acetate. All reactions were carried out under an atmosphere of argon or nitrogen in oven-dried glassware with magnetic stirring.

^{1}H and ^{13}C NMR data of new compounds other than **2c** were collected on a Bruker AVANCE III HD 400 spectrometer at ambient temperature. ^{1}H and ^{13}C NMR data of **2c** were collected on a Bruker Avance III 600 spectrometer at ambient temperature. All ^{1}H NMR experiments are reported in δ units, parts per million (ppm), and were measured relative to the signals for residual chloroform (7.26 ppm). All ^{13}C NMR spectra are reported in ppm relative to deuterochloroform (77.01 ppm) and were obtained with ^{1}H decoupling. HRMS data were obtained on a Bruker micrOTOF Focus II. Melting points were determined on a Yanaco MP-S3 and are uncorrected. UV–Vis absorption and fluorescence spectra were recorded on JASCO V-630 and JASCO FP-6200 spectrophotometers, respectively. Fluorescence quantum yields were obtained on a Hamamatsu Photonics, Absolute PL Quantum Yield Measurement System, C11347-01. Chiral HPLC analyses were performed on a JASCO HPLC 2000 series. Optical rotation values were measured on a JASCO P-2200. Electronic circular dichroism (ECD) spectra were obtained on a JASCO J-820 spectrometer. Single crystal X-ray diffraction data were collected using an XtaLAB mini II diffractometer with graphite monochromated Mo-Kα radiation.

2. Synthetic Experiment

2-1. Synthesis of Hexaynes

1-Bromo-2-iodo-4-(methoxymethoxy)benzene (5)

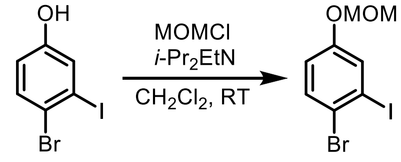

To a solution of **4**[1] (25.4 g, 85.4 mmol) and *N,N*-diisopropylethylamine (22.3 mL, 0.128 mol) in dry dichloromethane (200 mL) was added chloromethyl methyl ether (22.3 mL, 0.188 mol) at 0 °C for a 10 min period, and the reaction mixture was stirred at room temperature for 2 h. Then, the reaction mixture was diluted with water and extracted with dichloromethane (100 mL x 3). The organic layer was washed with brine, dried with Na_2SO_4, filtered, and concentrated under reduced pressure to give **5** (29.1 g, 84.8 mmol, >99% yield).

Colorless oil; ^{1}H NMR ($CDCl_3$, 400 MHz) δ 7.55 (d, J = 2.8 Hz, 1H), 7.48 (d, J = 8.8 Hz, 1H), 6.90 (dd, J = 8.8, 2.9 Hz, 1H), 5.12 (s, 2H), 3.46 (s, 3H); ^{13}C NMR ($CDCl_3$, 100 MHz) δ 156.3, 132.7, 128.0, 121.6, 117.9, 101.0, 94.6, 56.2; HRMS (APCI) calcd for $C_8H_8BrIO_2$ $[M]^+$ 341.8747 found 341.8747.

***N*-(3-(2-Bromo-5-(methoxymethoxy)phenyl)prop-2-yn-1-yl)-*N*-(but-2-yn-1-yl)-4-methylbenzenesulfonamide (7a)**

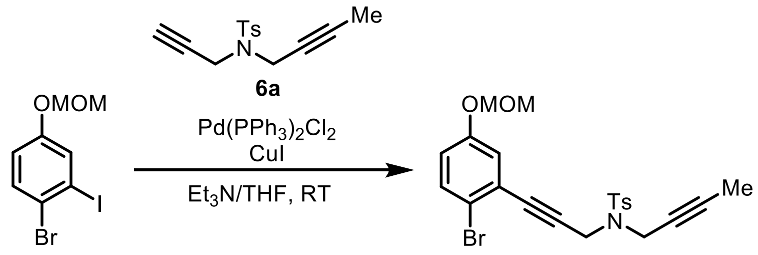

To a mixture of **5** (1.03 g, 3.00 mmol), **6a**[7] (0.823 g, 3.15 mmol), $Pd(PPh_3)_2Cl_2$ (0.105 g, 0.150 mmol), and CuI (0.0286 g, 0.150 mmol) in Et_3N/THF [1:1 (v/v), 10 mL] was stirred at room temperature for 18 h. The reaction mixture was concentrated and purified by silica gel column chromatography (eluent: *n*-hexane/EtOAc = 5:1) to give **7a** (1.16 g, 2.43 mmol, 81% yield).

Red oil; ^{1}H NMR ($CDCl_3$, 400 MHz) δ 7.74 (d, J = 8.3 Hz, 2H), 7.39 (d, J = 8.8 Hz, 1H), 7.24 (d, J = 8.8 Hz, 2H), 6.91 (d, J = 2.9 Hz, 1H), 6.86 (dd, J = 8.8, 3.0 Hz, 1H), 5.12 (s, 2H), 2.04 (s, 2H), 4.18 (q, J = 2.2 Hz, 2H), 3.47 (s, 3H), 2.32 (s, 3H), 1.69 (t, J = 2.4 Hz, 3H); ^{13}C NMR ($CDCl_3$, 100 MHz) δ 156.0, 143.7, 135.4, 133.0, 129.5, 128.0, 125.2, 121.0, 118.3, 117.3, 94.5, 86.5, 84.0, 82.0, 71.6, 56.1, 37.1, 37.1, 21.4, 3.5; HRMS (ESI) calcd for $C_{22}H_{22}BrNO_4SNa$ $[M+Na]^+$ 498.0345 found 498.0384.

1-Bromo-2-(3-(but-2-yn-1-yloxy)prop-1-yn-1-yl)-4-(methoxymethoxy)benzene (7b)

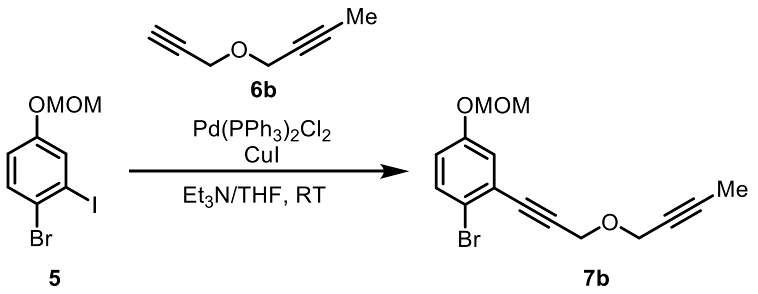

To a mixture of **5** (1.71 g, 5.00 mmol), **6b**[8] (0.568 g, 5.25 mmol), $Pd(PPh_3)_2Cl_2$ (0.175 g, 0.250 mmol), and CuI (0.0500 g, 0.263 mmol) in Et_3N/THF [1:1 (v/v), 10 mL] was stirred at room temperature for 18 h. The reaction mixture was concentrated and purified by silica gel column chromatography (eluent: *n*-hexane/EtOAc = 7:1) to give **7b** (1.27 g, 3.92 mmol, 78% yield).

Yellow oil; ^{1}H NMR ($CDCl_3$, 400 MHz) δ 7.44 (d, J = 8.8 Hz, 1H), 7.17 (d, J = 3.0 Hz, 1H), 6.88 (dd, J = 8.9, 3.0 Hz, 1H), 5.13 (s, 2H), 4.51 (s, 2H), 4.32 (q, J = 2.3 Hz, 2H), 1.88 (t, J = 2.3 Hz, 3H); ^{13}C NMR ($CDCl_3$, 100 MHz) δ 156.1, 133.1, 125.4, 121.0, 118.5, 117.5, 94.5, 94.5, 89.2, 85.0, 74.4, 57.3, 57.0, 56.1, 3.7; HRMS (ESI) calcd for $C_{15}H_{15}BrO_3Na$ $[M+Na]^+$ 345.0097 found 345.0101.

***N*-(Hept-2-yn-1-yl)-4-nitro-*N*-(prop-2-yn-1-yl)benzenesulfonamide (6c)**

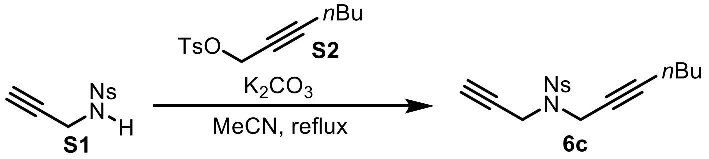

To a mixture of **S1**[2] (1.20 g, 5.00 mmol), **S2**[3] (1.60 g, 6.00 mmol), and K_2CO_3 (2.07 g, 15.0 mmol) in CH_3CN (40 mL) was stirred under reflux for 24 h. Then, the reaction mixture was diluted with water and extracted with ethyl acetate (20 mL x 3). The organic layer was washed with brine, dried with Na_2SO_4, filtered, and concentrated. The residue was purified by silica gel column chromatography (eluent: *n*-hexane/EtOAc = 4:1) to give **6c** (1.51 g, 4.52 mmol, 90% yield).

Orange oil; ^{1}H NMR ($CDCl_3$, 400 MHz) δ 8.08–8.06 (m, 1H), 7.74–7.64 (m, 3H), 4.29 (d, J = 2.4 Hz, 2H), 4.27 (t, J = 2.0 Hz, 2H), 2.22 (t, J = 2.5 Hz, 1H), 2.06 (tt, J = 6.9, 2.3 Hz, 2H), 1.39–1.25 (m, 4H), 0.86 (t, J = 7.2 Hz, 3H); ^{13}C NMR ($CDCl_3$, 100 MHz) δ 148.3, 133.8, 132.6, 131.7, 131.3, 124.2, 86.9, 76.7, 73.8, 72.1, 37.2, 36.5, 30.4, 21.8, 18.2, 13.5; HRMS (ESI) calcd for $C_{16}H_{18}N_2O_4SNa$ $[M+Na]^+$ 357.0879 found 357.0885.

***N*-(3-(2-Bromo-5-(methoxymethoxy)phenyl)prop-2-yn-1-yl)-*N*-(hept-2-yn-1-yl)-4-nitro-benzenesulfonamide (7c)**

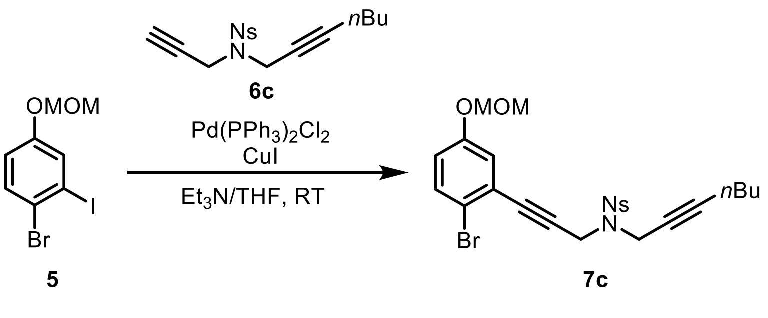

To a mixture of **5** (1.71 g, 5.00 mmol), **6c** (1.69 g, 5.05 mmol), $Pd(PPh_3)_2Cl_2$ (0.176 g, 0.250 mmol), and CuI (0.0476 g, 0.250 mmol) in Et_3N/THF [1:1 (v/v), 20 mL] was stirred at room temperature for 18 h. The reaction mixture was concentrated and purified by silica gel column chromatography (eluent: *n*-hexane/EtOAc = 3:1) to give **7c** (2.21 g, 4.04 mmol, 80% yield).

Red oil; ^{1}H NMR ($CDCl_3$, 400 MHz) δ 8.12–8.10 (m, 1H), 7.68–7.61 (m, 3H), 7.40 (d, *J* = 8.8 Hz, 1H), 7.01 (d, *J* = 2.9 Hz, 1H), 6.87 (dd, *J* = 8.8, 3.0 Hz, 1H), 5.12 (s, 2H), 4.57 (s, 2H), 4.38 (t, *J* = 2.0 Hz, 2H), 3.46 (s, 3H), 2.09 (tt, *J* = 6.9, 2.3 Hz, 2H), 1.41–1.26 (m, 4H), 4.38 (t, *J* = 7.2 Hz, 3H); ^{13}C NMR ($CDCl_3$, 100 MHz) δ 156.0, 148.3, 133.7, 133.1, 132.7, 131.7, 131.3, 124.9, 124.2, 120.8, 118.7, 117.3, 94.5, 86.9, 86.6, 84.0, 72.4, 56.2, 37.5, 37.4, 30.5, 21.9, 18.3, 13.6, ; HRMS (ESI) calcd for $C_{24}H_{25}BrN_2O_6SNa$ $[M+Na]^+$ 571.0509 found 571.0553.

N-(3-(2-Bromo-5-(methoxymethoxy)phenyl)prop-2-yn-1-yl)-4-methyl-*N*-(3-phenylprop-2-yn-1-yl)benzenesulfonamide (7d)

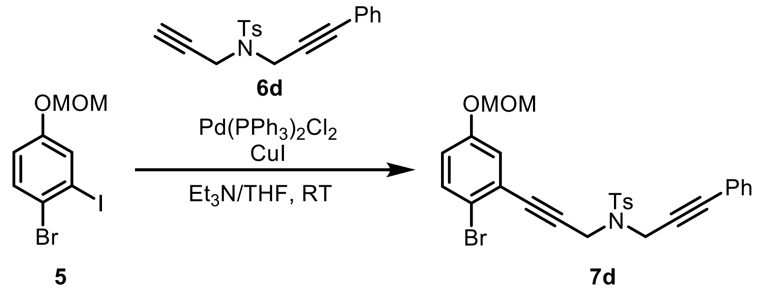

To a mixture of **5** (1.86 g, 5.40 mmol), **6d**[9] (1.93 g, 5.96 mmol), $Pd(PPh_3)_2Cl_2$ (0.190 g, 0.270 mmol), and CuI (0.0514 g, 0.270 mmol) in Et_3N/THF [1:1 (v/v), 15 mL] was stirred at room temperature for 18 h. The reaction mixture was concentrated and purified by silica gel column chromatography (eluent: *n*-hexane/EtOAc = 5:1) to give **7d** (2.47 g, 4.60 mmol, 85% yield).

Red oil; ^{1}H NMR ($CDCl_3$, 400 MHz) δ 7.78 (d, *J* = 8.3 Hz, 2H), 7.40 (d, *J* = 8.8 Hz, 1H), 7.31–7.20 (m, 7H), 6.95 (d, *J* = 2.9 Hz, 1H), 6.86 (dd, *J* = 8.8, 3.0 Hz, 1H), 5.11 (s, 2H), 4.50 (s, 2H), 4.48 (s, 2H), 3.46 (s, 3H), 2.28 (s, 3H); ^{13}C NMR ($CDCl_3$, 100 MHz) δ 156.0, 144.0, 135.2, 133.0, 131.7, 129.6, 128.5, 128.2, 128.0, 125.1, 122.3, 121.0, 118.4, 117.4, 94.5, 86.3, 85.9, 84.3, 81.6, 56.1, 37.6, 37.5, 21.4; HRMS (ESI) calcd for $C_{27}H_{24}BrNO_4SNa$ $[M+Na]^+$ 560.0502 found 560.0545.

2-Ethynyl-2'-(hex-1-yn-1-yl)-1,1'-biphenyl (6e)

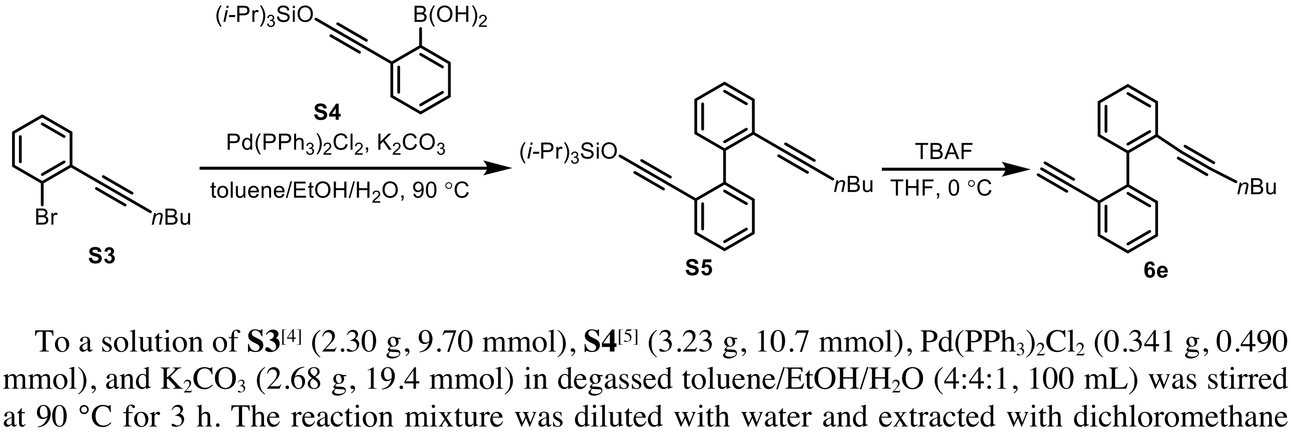

To a solution of **S3**[4] (2.30 g, 9.70 mmol), **S4**[5] (3.23 g, 10.7 mmol), $Pd(PPh_3)_2Cl_2$ (0.341 g, 0.490 mmol), and K_2CO_3 (2.68 g, 19.4 mmol) in degassed toluene/EtOH/H_2O (4:4:1, 100 mL) was stirred at 90 °C for 3 h. The reaction mixture was diluted with water and extracted with dichloromethane (100 mL x 3). The organic layer was washed with brine, dried with Na_2SO_4, filtered, and concentrated. The residue was passed through silica gel column chromatography (eluent: *n*-hexane) to give crude **S5**. This crude **S5** was used for the next reaction without further purification.

To a solution of the crude **S5** in THF (50 mL) was added tetrabutylammonium fluoride (TBAF, 15.0 mL, 15.0 mmol, 1.0 mol/L in THF) at 0 °C. After being stirred at room temperature for 30 min,

the reaction mixture was diluted with water, and extracted with CH_2Cl_2 (100 mL x 3). The organic layer was washed with brine, dried with Na_2SO_4, filtered, and concentrated. The residue was purified by silica gel column chromatography (*n*-hexane) to give **6e** (2.06 g, 8.00 mmol, 82% yield).

Orange oil; ^{1}H NMR ($CDCl_3$, 400 MHz) δ 7.60–7.58 (m, 1H), 7.50–7.48 (m, 1H), 7.40–7.26 (m, 6H), 2.93 (s, 1H), 2.21 (t, J = 6.8 Hz, 2H), 1.36–1.29 (m, 2H), 1.24–1.15 (m, 2H), 0.80 (t, J = 7.3 Hz, 3H); ^{13}C NMR ($CDCl_3$, 100 MHz) δ 144.0, 142.7, 132.9, 132.1, 130.2, 129.8, 128.1, 127.4, 127.1, 126.9, 123.5, 121.6, 93.7, 82.8, 79.8, 79.7, 30.5, 21.6, 19.1, 13.6; HRMS (ESI) calcd for $C_{20}H_{18}Na$ $[M+Na]^+$ 281.1301 found 281.1301.

2-((2-Bromo-5-(methoxymethoxy)phenyl)ethynyl)-2'-(hex-1-yn-1-yl)-1,1'-biphenyl (7e)

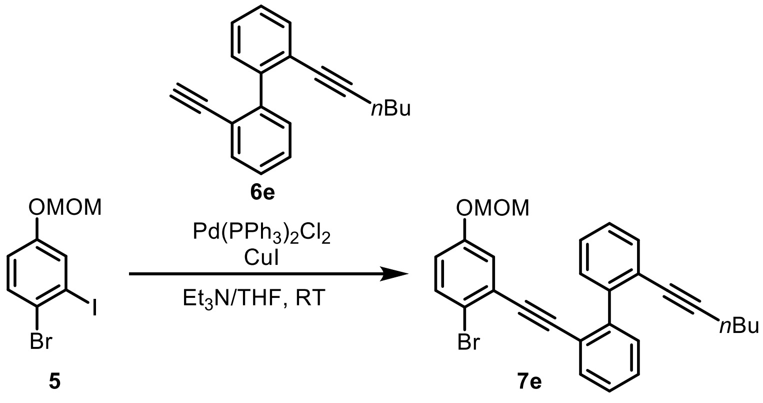

To a mixture of **5** (2.74 g, 8.00 mmol), **6e** (2.07 g, 8.00 mmol), $Pd(PPh_3)_2Cl_2$ (0.281 g, 0.400 mmol), and CuI (0.152 g, 0.800 mmol) in Et_3N/THF [1:1 (v/v), 16 mL] was stirred at room temperature for 18 h. The reaction mixture was concentrated and purified by silica gel column chromatography (eluent: *n*-hexane/EtOAc = 10:1) to give **7e** (3.27 g, 6.90 mmol, 86% yield).

Red oil; ^{1}H NMR ($CDCl_3$, 400 MHz) δ 7.67 (dd, J = 7.4, 1.4 Hz, 1H), 7.50 (dd, J = 7.5, 1.4 Hz, 1H), 7.44 (dd, J = 7.4, 1.5 Hz, 2H), 7.39–7.24 (m, 5H), 6.91 (d, J = 2.9 Hz, 1H), 6.79 (dd, J = 8.8, 3.0 Hz, 1H), 5.08 (s, 2H), 3.44 (s, 3H), 2.21 (t, J = 6.8 Hz, 2H), 1.36–1.29 (m, 2H), 1.26–1.16 (m, 2H), 0.79 (t, J = 7.3 Hz, 3H); ^{13}C NMR ($CDCl_3$, 100 MHz) δ 156.0, 143.7, 142.8, 132.9, 132.4, 132.2, 130.3, 130.2, 128.0, 127.3, 127.1, 127.0, 126.4, 123.6, 122.3, 120.7, 118.0, 117.1, 94.5, 93.7, 93.6, 90.5, 79.9, 56.1, 30.5, 21.7, 19.1, 13.6; HRMS (ESI) calcd for $C_{28}H_{25}BrO_2Na$ $[M+Na]^+$ 495.0930 found 495.0964.

((3-(2-Bromophenyl)prop-2-yn-1-yl)oxy)(*tert*-butyl)diphenylsilane (S8)

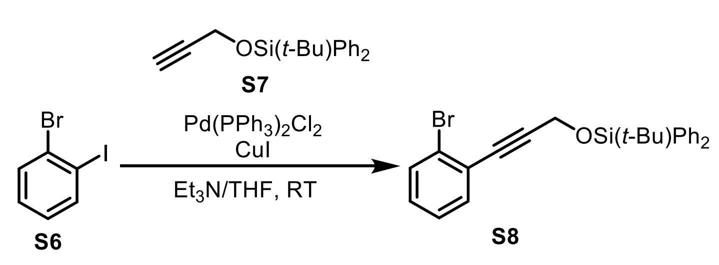

To a mixture of **S6** (17.0 mL, 136 mmol), **S7**[6] (41.2 g, 140.0 mmol), $Pd(PPh_3)_2Cl_2$ (0.0954 g, 0.136 mmol), and CuI (0.0518 g, 0.272 mmol) in Et_3N/THF [1:1 (v/v), 200 mL] was stirred at room temperature for 24 h. The reaction mixture was concentrated and purified by silica gel column chromatography (eluent: *n*-hexane) to give **S8** (60.7 g, 135 mmol, >99% yield).

Yellow oil; ^{1}H NMR ($CDCl_3$, 400 MHz) δ 7.77 (dd, J = 7.7, 1.6 Hz, 4H), 7.55 (d, J = 8.0 Hz, 1H), 7.43–7.34 (m, 7H), 7.23–7.20 (m, 1H), 7.15–7.11 (m, 1H), 4.60 (s, 2H), 1.09 (s, 9H); ^{13}C NMR ($CDCl_3$, 100 MHz) δ 135.7, 133.6, 133.2, 132.4, 129.8, 129.4, 127.8, 126.9, 125.4, 125.1, 92.3, 83.6, 53.3, 26.8, 19.3; HRMS (ESI) calcd for $C_{25}H_{25}BrOSiNa$ $[M+Na]^+$ 471.0750 found 471.0741.

(2-(3-((*tert*-Butyldiphenylsilyl)oxy)prop-1-yn-1-yl)phenyl)boronic acid (8)

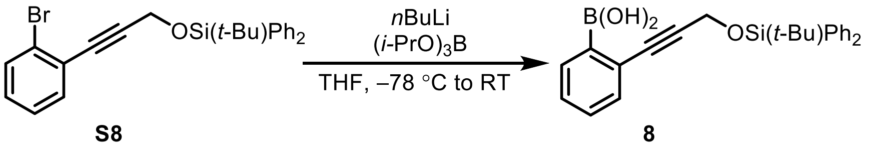

A 1.6 M solution of *n*-BuLi in hexane (53.0 mL, 84.8 mmol) was added dropwise to a solution of **S8** (30.5 g, 67.8 mmol) in THF (300 mL) at –78 °C. After stirring at –78 °C for 30 min, B(O*i*-Pr)$_3$ (31.1 mL, 136 mmol) was added at –78 °C. The reaction mixture was stirred for 16 hours while the temperature was raised to room temperature. The reaction was quenched with 1 M aqueous HCl solution (100 mL), extracted with Et_2O (100 mL x 3), dried over Na_2SO_4, filtered, and concentrated. The residue was purified by silica gel column chromatography (eluent: *n*-hexane/EtOAc = 5:1) to give **8** (23.2 g, 55.9 mmol, 82% yield).

Pale yellow oil; ^{1}H NMR ($CDCl_3$, 400 MHz) δ 7.98–7.96 (m, 1H), 7.73 (dd, J = 7.8, 1.6 Hz, 4H), 7.47–7.35 (m, 9H), 4.57 (s, 2H), 1.09 (s, 9H); ^{13}C NMR ($CDCl_3$, 100 MHz) δ 135.6, 132.8, 132.4, 130.7, 130.0, 128.3, 127.9, 127.9, 126.3, 91.7, 86.0, 53.0, 26.7; HRMS (ESI) calcd for $C_{25}H_{27}BO_3SiNa$ $[M+Na]^+$ 437.1715 found 437.1751.

***N*-(But-2-yn-1-yl)-*N*-(3-(2'-(3-hydroxyprop-1-yn-1-yl)-4-(methoxymethoxy)-[1,1'-biphenyl]-2-yl)prop-2-yn-1-yl)-4-methylbenzenesulfonamide (9a)**

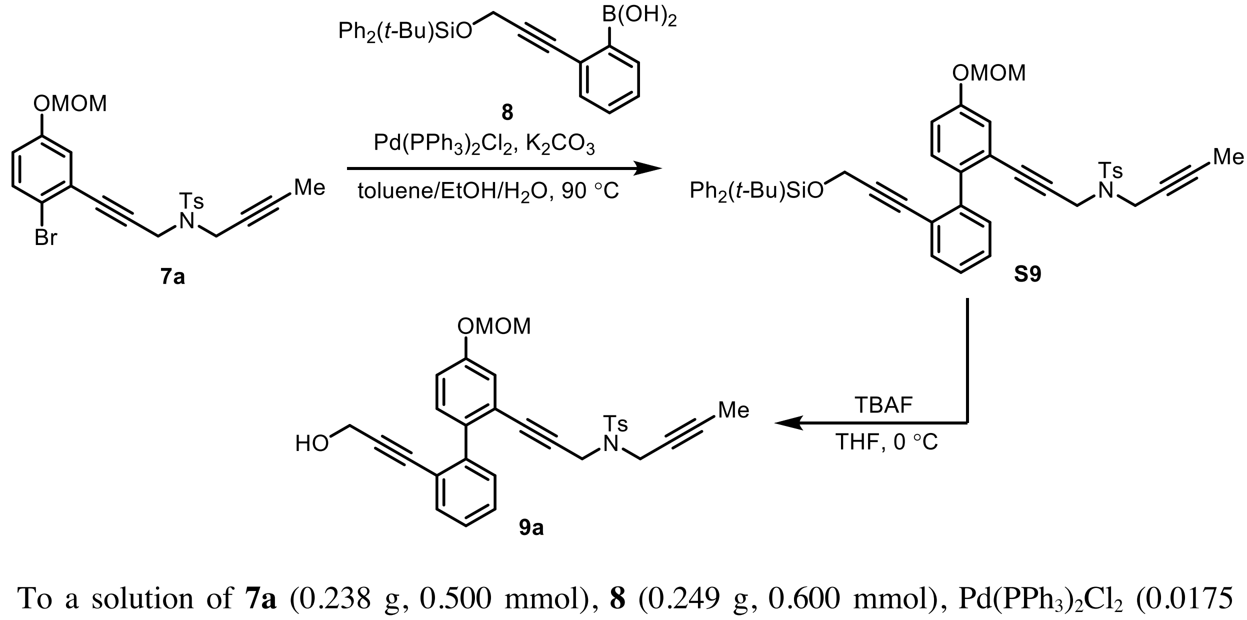

To a solution of **7a** (0.238 g, 0.500 mmol), **8** (0.249 g, 0.600 mmol), $Pd(PPh_3)_2Cl_2$ (0.0175 g, 0.0250 mmol) and K_2CO_3 (0.138 g, 1.00 mmol) in degassed toluene/EtOH/H_2O (4:4:1, 10 mL) was stirred at 90 °C for 16 h. The reaction mixture was diluted with water and extracted with ethyl acetate (20 mL x 3). The organic layer was washed with brine, dried with Na_2SO_4, filtered, and concentrated. The residue was passed through silica gel column chromatography (eluent: *n*-hexane/EtOAc = 2:1) to give crude **S9**. This crude **S9** was used for the next reaction without further purification.

To a solution of the crude **S9** in THF (10 mL) was added TBAF (1.0 mL, 1.0 mmol, 1.0 mol/L in THF) at 0 °C. After being stirred at room temperature for 30 min, the reaction mixture was diluted with water and extracted with CH_2Cl_2 (20 mL x 3). The organic layer was washed with brine, dried with Na_2SO_4, filtered, and concentrated. The residue was purified by silica gel PTLC (eluent: *n*-hexane/EtOAc = 2:1) to give **9a** (0.106 g, 0.201 mmol, 40% yield).

Red oil; ^{1}H NMR ($CDCl_3$, 400 MHz) δ 7.64 (d, J = 8.3 Hz, 2H), 7.50 (dd, J = 7.4, 1.5 Hz, 1H), 7.32–7.21 (m, 6H), 7.04–7.01 (m, 2H), 5.19 (s, 2H), 4.28 (s, 2H), 4.15 (s, 2H), 3.79–3.78 (m, 2H), 3.51 (s, 3H), 2.35 (s, 3H), 1.60 (t, J = 2.4 Hz, 3H), ; ^{13}C NMR ($CDCl_3$, 100 MHz) δ 156.2, 143.6, 142.4, 136.7, 135.5, 132.5, 131.2, 130.2, 129.4, 128.0, 127.9, 127.2, 122.8, 122.1, 119.6, 116.3, 94.5, 90.4, 85.1, 84.9, 84.6, 81.7, 71.6, 56.2, 51.6, 37.0, 36.6, 21.4, 3.4; HRMS (ESI) calcd for $C_{31}H_{29}NO_5SNa$ [M+Na]$^+$ 550.1659 found 550.1613.

3-(2'-(3-(But-2-yn-1-yloxy)prop-1-yn-1-yl)-4'-(methoxymethoxy)-[1,1'-biphenyl]-2-yl)prop-2-yn-1-ol (9b)

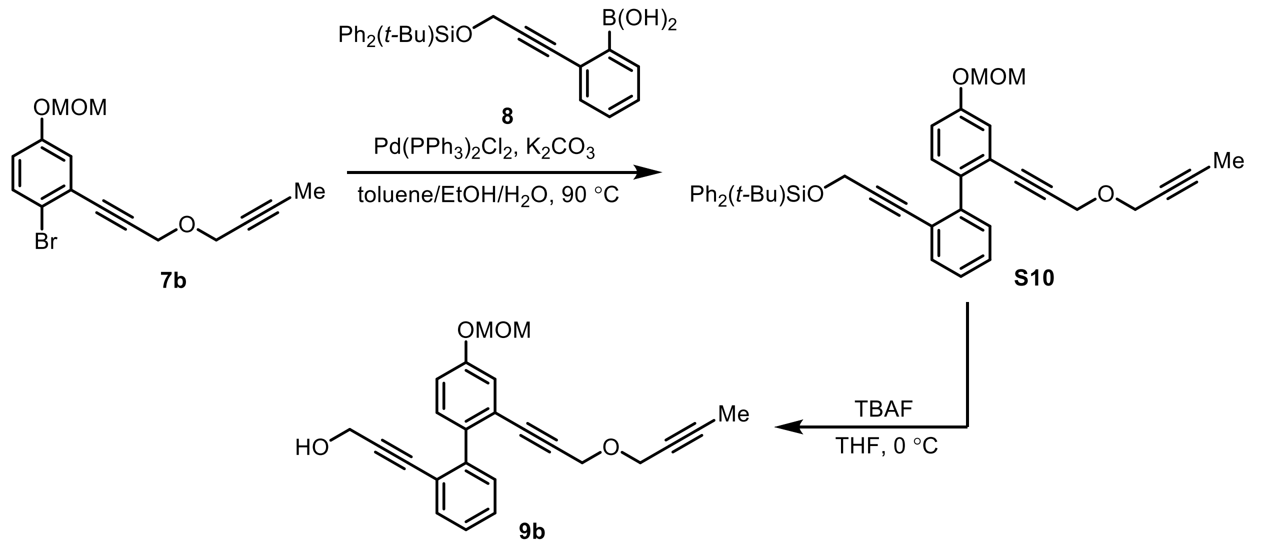

To a solution of **7b** (0.162 g, 0.500 mmol), **8** (0.249 g, 0.600 mmol), $Pd(PPh_3)_2Cl_2$ (0.0175 g, 0.0250 mmol) and K_2CO_3 (0.138 g, 1.00 mmol) in degassed toluene/EtOH/H_2O (4:4:1, 10 mL) was stirred at 90 °C for 4 h. The reaction mixture was diluted with water and extracted with CH_2Cl_2 (20 mL x 3). The organic layer was washed with brine, dried with Na_2SO_4, filtered, and concentrated. The residue was passed through silica gel column chromatography (eluent: *n*-hexane/EtOAc = 4:1) to give crude **S10**. This crude **S10** was used for the next reaction without further purification.

To a solution of the crude **S10** in THF (10 mL) was added TBAF (1.0 mL, 1.0 mmol, 1.0 mol/L in THF) at 0 °C. After being stirred at room temperature for 30 min, the reaction mixture was diluted with water, and extracted with CH_2Cl_2 (20 mL x 3). The organic layer was washed with brine, dried with Na_2SO_4, filtered, and concentrated. The residue was purified by silica gel PTLC (eluent: *n*-hexane/EtOAc = 2:1) to give **9b** (0.0719 g, 0.192 mmol, 38% yield).

Red oil; ^{1}H NMR ($CDCl_3$, 400 MHz) δ 7.44 (d, J = 7.3 Hz, 1H), 7.29–7.18 (m, 4H), 7.14 (d, J = 2.6 Hz, 1H), 6.96 (dd, J = 8.5, 2.6 Hz, 1H), 5.11 (s, 2H), 4.21–4.20 (m, 2H), 4.18 (s, 2H), 3.90 (q, J = 2.3 Hz, 2H), 3.41 (s, 3H), 1.76 (t, J = 2.3 Hz, 3H); ^{13}C NMR ($CDCl_3$, 100 MHz) δ 156.3, 142.5, 136.8, 132.5, 131.3, 130.4, 128.0, 127.2, 123.0, 122.2, 119.6, 116.6, 94.5, 90.5, 87.5, 85.9, 85.1, 83.0, 74.5, 57.0, 56.8, 56.2, 51.6, 3.6; HRMS (ESI) calcd for $C_{24}H_{22}O_4Na$ [M+Na]$^+$ 397.1410 found 397.1441.

***N*-(Hept-2-yn-1-yl)-*N*-(3-(2'-(3-hydroxyprop-1-yn-1-yl)-4-(methoxymethoxy)-[1,1'-biphenyl]-2-yl)prop-2-yn-1-yl)-4-nitrobenzenesulfonamide (9c)**

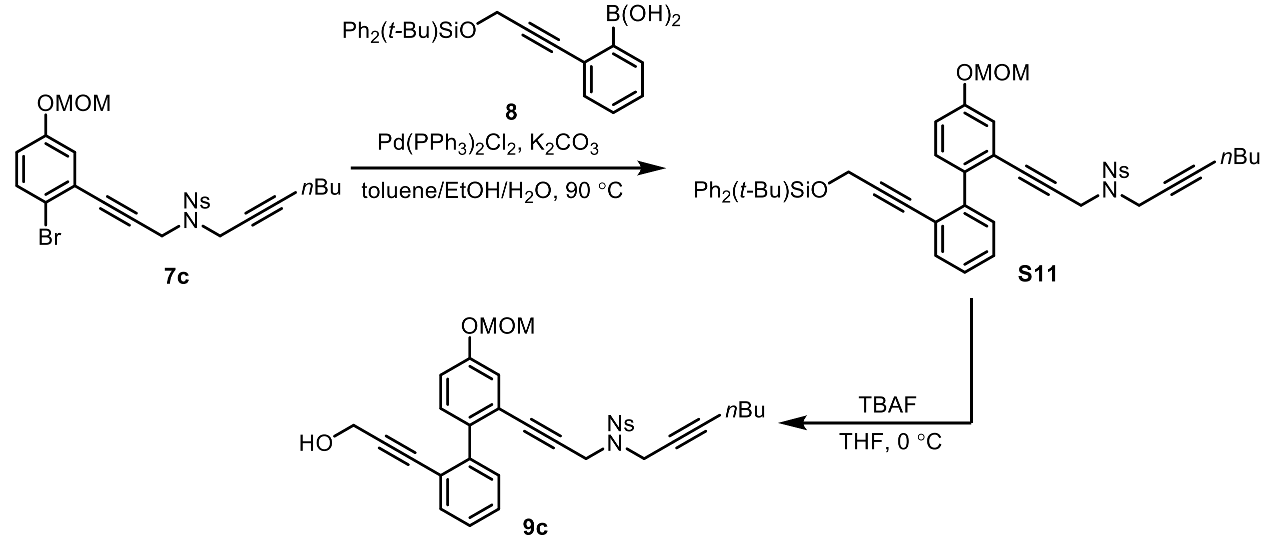

To a solution of **7c** (0.110 g, 0.200 mmol), **8** (0.0995 g, 0.240 mmol), $Pd(PPh_3)_2Cl_2$ (0.00702 g, 0.0100 mmol) and K_2CO_3 (0.0553 g, 0.400 mmol) in degassed toluene/EtOH/H_2O (4:4:1, 9 mL) was stirred at 90 °C for 5 h. The reaction mixture was diluted with water and extracted with CH_2Cl_2 (20 mL x 3). The organic layer was washed with brine, dried with Na_2SO_4, filtered, and concentrated. The residue was passed through silica gel column chromatography (eluent: *n*-hexane/EtOAc = 2:1) to give crude **S11**. This crude **S11** was used for the next reaction without further purification.

To a solution of the crude **S11** in THF (10 mL) was added TBAF (0.5 mL, 0.5 mmol, 1.0 mol/L in THF) at 0 °C. After being stirred at room temperature for 30 min, the reaction mixture was diluted with water, and extracted with CH_2Cl_2 (20 mL x 3). The organic layer was washed with brine, dried with Na_2SO_4, filtered, and concentrated. The residue was purified by silica gel PTLC (eluent: *n*-hexane/EtOAc = 1:1) to give **9c** (0.0493 g, 0.0820 mmol, 41% yield).

Red oil; ^{1}H NMR ($CDCl_3$, 400 MHz) δ 7.98–7.96 (m, 1H), 7.65–7.59 (m, 3H), 7.52–7.49 (m, 1H), 7.35–7.26 (m, 5H), 7.08 (d, J = 2.5 Hz, 1H), 7.04 (dd, J = 8.5, 2.6 Hz, 1H), 5.20 (s, 2H), 4.32 (s, 2H), 4.30 (s, 2H), 3.92 (m, 2H), 3.52 (s, 3H), 2.04–2.01 (m, 2H), 1.34–1.24 (m, 4H), 0.85 (t, J = 7.1 Hz, 3H); ^{13}C NMR ($CDCl_3$, 100 MHz) δ 156.2, 148.2, 142.4, 136.8, 133.6, 132.8, 132.6, 131.6, 131.2, 131.1, 130.2, 128.1, 127.3, 124.2, 122.6, 122.1, 119.4, 116.7, 94.5, 90.5, 86.5, 85.0, 84.9, 84.7, 72.4, 56.2, 51.6, 37.4, 36.9, 30.5, 21.8, 18.2, 13.6; HRMS (ESI) calcd for $C_{33}H_{32}N_2O_7SNa$ $[M+Na]^+$ 623.1822 found 623.1850.

***N*-(3-(2'-(3-Hydroxyprop-1-yn-1-yl)-4-(methoxymethoxy)-[1,1'-biphenyl]-2-yl)prop-2-yn-1-yl)-4-methyl-*N*-(3-phenylprop-2-yn-1-yl)benzenesulfonamide (9d)**

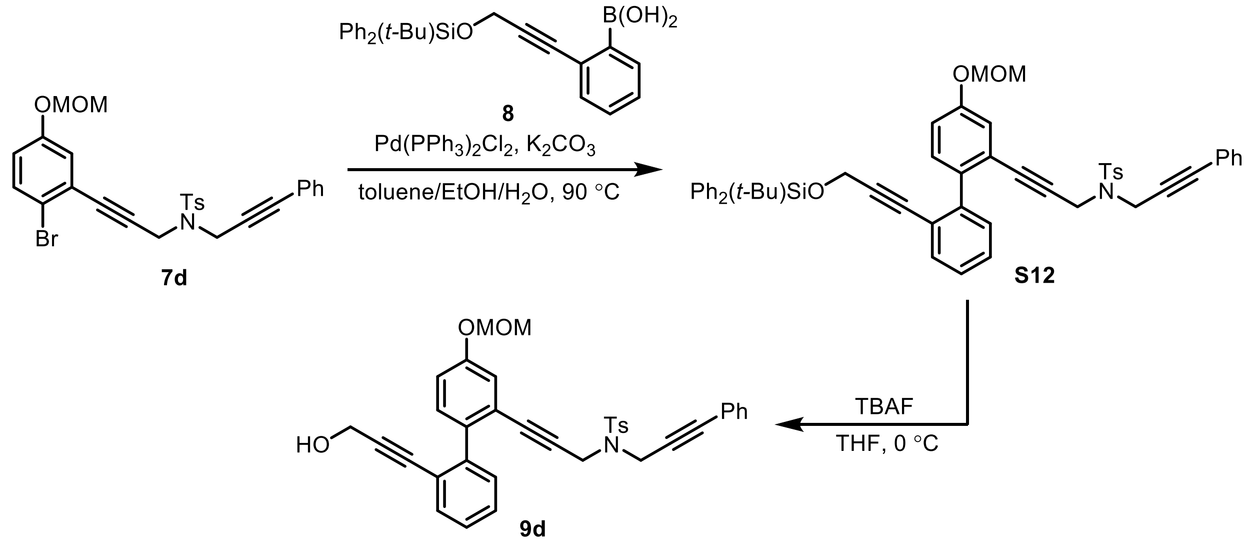

To a solution of **7d** (0.269 g, 0.500 mmol), **8** (0.249 g, 0.600 mmol), $Pd(PPh_3)_2Cl_2$ (0.0175 g, 0.0250 mmol) and K_2CO_3 (0.138 g, 1.00 mmol) in degassed toluene/EtOH/H_2O (4:4:1, 9 mL) was stirred at 90 °C for 5 h. The reaction mixture was diluted with water and extracted with CH_2Cl_2 (20 mL x 3). The organic layer was washed with brine, dried with Na_2SO_4, filtered, and concentrated. The residue was passed through silica gel column chromatography (eluent: *n*-hexane/EtOAc = 6:1) to give crude **S12**. This crude **S12** was used for the next reaction without further purification.

To a solution of the crude **S12** in THF (10 mL) was added TBAF (1.0 mL, 1.0 mmol, 1.0 mol/L in THF) at 0 °C. After being stirred at room temperature for 30 min, the reaction mixture was diluted with water, and extracted with CH_2Cl_2 (20 mL x 3). The organic layer was washed with brine, dried with Na_2SO_4, filtered, and concentrated. The residue was purified by silica gel PTLC (eluent: *n*-hexane/EtOAc = 2:1) to give **9d** (0.109 g, 0.186 mmol, 37% yield).

Red oil; ^{1}H NMR ($CDCl_3$, 400 MHz) δ 7.68 (d, J = 8.3 Hz, 2H), 7.51 (d, J = 7.7 Hz, 1H), 7.32–7.21 (m, 9H), 7.16–7.13 (m, 2H), 7.06–7.02 (m, 2H), 5.19 (s, 2H), 4.28–4.27 (m, 2H), 4.21 (s, 2H), 4.04 (s, 2H), 3.51 (s, 3H), 2.30 (s, 3H); ^{13}C NMR ($CDCl_3$, 100 MHz) δ 156.3, 143.8, 142.4, 136.8, 135.3, 132.6, 131.6, 131.2, 130.2, 129.6, 128.5, 128.2, 128.1, 127.8, 127.3, 122.8, 122.3, 122.2, 119.5, 116.5, 94.5, 90.4, 85.7, 85.0, 84.9, 84.7, 81.6, 56.2, 51.6, 37.3, 36.9, 21.4; HRMS (ESI) calcd for $C_{36}H_{31}NO_5SNa$ $[M+Na]^+$ 612.1815 found 612.1870.

3-(2'-((2'-(Hex-1-yn-1-yl)-[1,1'-biphenyl]-2-yl)ethynyl)-4'-(methoxymethoxy)-[1,1'-biphenyl]-2-yl)prop-2-yn-1-ol (9e)

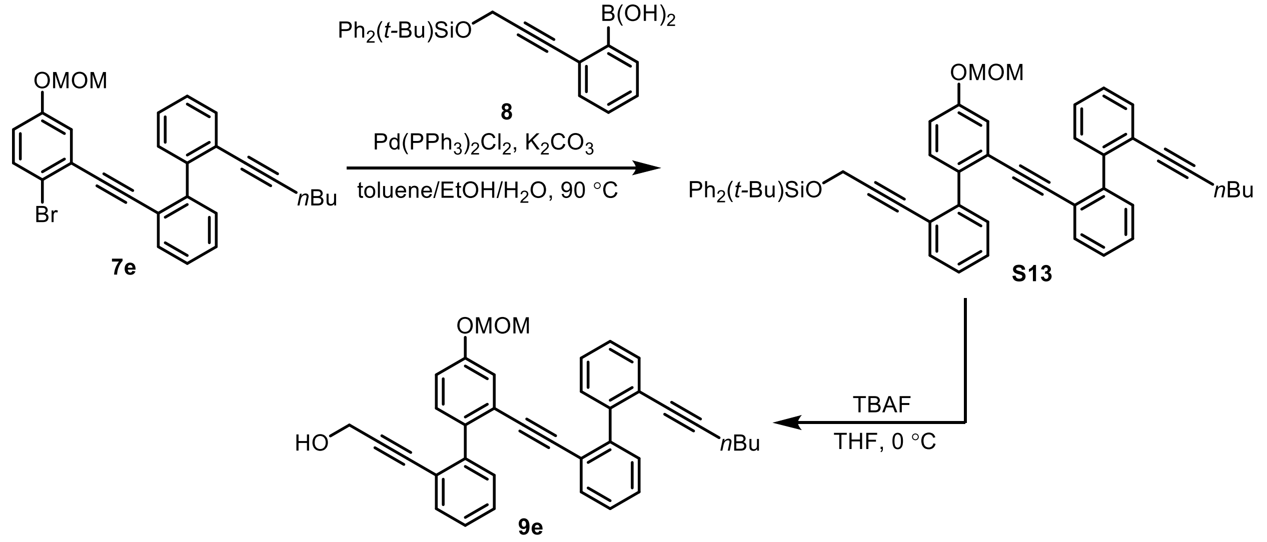

To a solution of **7e** (0.237 g, 0.500 mmol), **8** (0.249 g, 0.600 mmol), $Pd(PPh_3)_2Cl_2$ (0.0175 g, 0.0250 mmol) and K_2CO_3 (0.138 g, 1.00 mmol) in degassed toluene/EtOH/H_2O (4:4:1, 9 mL) was stirred at 90 °C for 5 h. The reaction mixture was diluted with water and extracted with CH_2Cl_2 (20 mL x 3). The organic layer was washed with brine, dried with Na_2SO_4, filtered, and concentrated. The residue was passed through silica gel column chromatography (eluent: *n*-hexane/EtOAc = 4:1) to give crude **S13**. This crude **S13** was used for the next reaction without further purification.

To a solution of the crude **S13** in THF (10 mL) was added TBAF (1.0 mL, 1.0 mmol, 1.0 mol/L in THF) at 0 °C. After being stirred at room temperature for 30 min, the reaction mixture was diluted with water, and extracted with CH_2Cl_2 (20 mL x 3). The organic layer was washed with brine, dried with Na_2SO_4, filtered, and concentrated. The residue was purified by silica gel PTLC (eluent: *n*-hexane/EtOAc = 2:1) to give **9e** (0.179 g, 0.341 mmol, 68% yield).

Red oil; ^{1}H NMR ($CDCl_3$, 400 MHz) δ 7.52–7.47 (m, 2H), 7.42–7.40 (m, 1H), 7.35–7.20 (m, 10H), 6.97 (dd, J = 8.5, 2.7 Hz, 1H), 6.91 (d, J = 2.6 Hz, 1H), 5.15 (s, 2H), 4.25 (s, 2H), 3.49 (s, 3H), 2.19 (t, J = 6.8 Hz, 2H), 1.35–1.14 (m, 4H), 0.78 (t, J = 7.3 Hz, 3H); ^{13}C NMR ($CDCl_3$, 100 MHz) δ 156.2, 143.0, 142.6, 142.5, 136.0, 132.5, 132.2, 132.2, 131.4, 130.7, 130.3, 130.2, 127.8, 127.5, 127.2, 127.1, 127.0, 126.8, 123.9, 123.4, 122.6, 121.9, 120.0, 116.0, 94.5, 93.6, 92.0, 91.5, 90.1, 85.4, 79.9, 56.1, 51.7, 30.5, 21.7, 19.1, 13.6; HRMS (ESI) calcd for $C_{37}H_{32}O_3Na$ $[M+Na]^+$ 547.2244 found 547.2289.

***N*-(3-(2'-(3-Bromoprop-1-yn-1-yl)-4-hydroxy-[1,1'-biphenyl]-2-yl)prop-2-yn-1-yl)-*N*-(but-2-yn-1-yl)-4-methylbenzenesulfonamide (1a)**

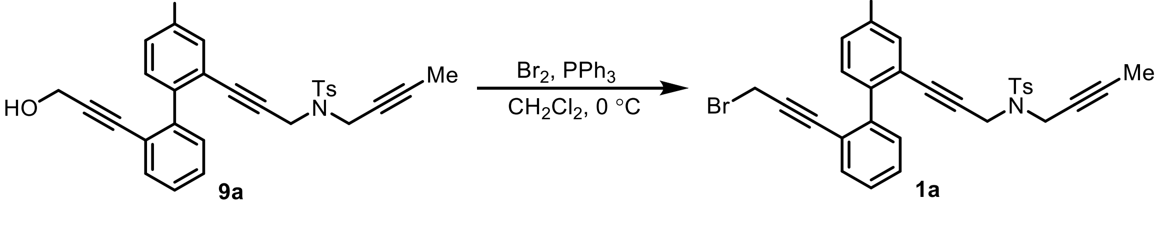

Br_2 (25 μL, 0.480 mmol) and PPh_3 (0.126 g, 0.480 mmol) were dissolved in CH_2Cl_2 (1 mL), and the mixture was stirred at 0 °C for 15 min. To this solution was added a solution of **9a** (0.106 g, 0.200 mmol) in CH_2Cl_2 (1 mL). The mixture was stirred at 0 °C for 1 h and then room temperature for 18 h. The resulting mixture was concentrated and purified by silica gel PTLC (eluent: *n*-

hexane/EtOAc = 2:1) to give **1a** (0.0584 g, 0.107 mmol, 53% yield).

Red oil; ^{1}H NMR ($CDCl_3$, 400 MHz) δ 7.63 (d, J = 8.3 Hz, 2H), 7.51–7.48 (m, 1H), 7.36–7.22 (m, 6H), 6.84 (dd, J = 8.4, 2.7 Hz, 1H), 6.78 (d, J = 2.6 Hz, 1H), 4.17 (s, 2H), 3.98 (s, 2H), 3.77 (m, 2H), 2.36 (s, 3H), 1.63 (t, J = 2.4 Hz, 3H); ^{13}C NMR ($CDCl_3$, 100 MHz) δ 154.6, 143.6, 142.6, 135.4, 135.3, 132.7, 131.5, 130.3, 129.4, 128.3, 127.9, 127.1, 122.8, 121.6, 118.8, 115.6, 87.0, 86.3, 84.8, 84.5, 81.7, 71.6, 37.0, 36.6, 21.5, 15.4, 3.5; HRMS (ESI) calcd for $C_{29}H_{24}BrNO_3SNa$ [M+Na]$^+$ 568.0552 found 568.0499.

2'-(3-Bromoprop-1-yn-1-yl)-2-(3-(but-2-yn-1-yloxy)prop-1-yn-1-yl)-[1,1'-biphenyl]-4-ol (1b)

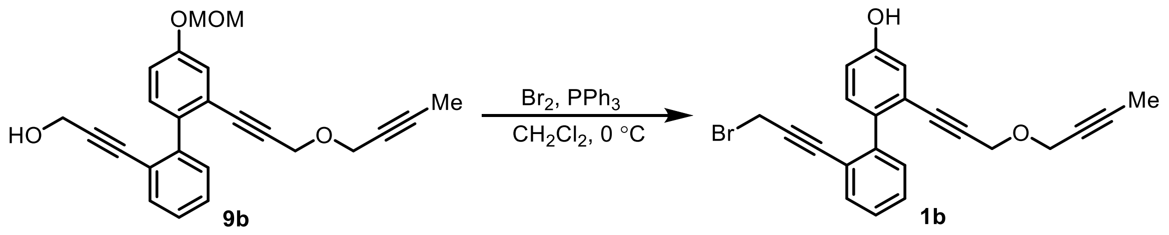

Br_2 (25 μL, 0.480 mmol) and PPh_3 (0.126 g, 0.480 mmol) were dissolved in CH_2Cl_2 (1 mL), and the mixture was stirred at 0 °C for 15 min. To this solution was added a solution of **9b** (0.0749 g, 0.200 mmol) in CH_2Cl_2 (1 mL). The mixture was stirred at 0 °C for 1 h and then room temperature for 18 h. The resulting mixture was concentrated and purified by silica gel PTLC (eluent: *n*-hexane/EtOAc = 2:1) to give **1b** (0.0434 g, 0.110 mmol, 55% yield).

Red oil; ^{1}H NMR ($CDCl_3$, 400 MHz) δ 7.52 (dd, J = 7.7, 1.0 Hz, 1H), 7.41–7.34 (m, 2H), 7.31–7.25 (m, 2H), 7.01 (d, J = 2.7 Hz, 1H), 6.87 (dd, J = 8.4, 2.7 Hz, 1H), 4.28 (s, 2H), 3.99 (s, 2H), 3.97 (q, J = 2.3 Hz, 2H), 1.84 (t, J = 2.3 Hz, 3H); ^{13}C NMR ($CDCl_3$, 100 MHz) δ 154.8, 142.8, 135.5, 132.6, 131.6, 130.5, 128.3, 127.1, 123.0, 121.7, 118.8, 115.7, 87.4, 87.0, 86.4, 85.7, 83.0, 74.4, 57.0, 56.8, 15.5, 3.6; HRMS (ESI) calcd for $C_{22}H_{17}BrO_2Na$ [M+Na]$^+$ 415.0304 found 415.0328.

N-(3-(2'-(3-Bromoprop-1-yn-1-yl)-4-hydroxy-[1,1'-biphenyl]-2-yl)prop-2-yn-1-yl)-*N*-(hept-2-yn-1-yl)-4-nitrobenzenesulfonamide (1c)

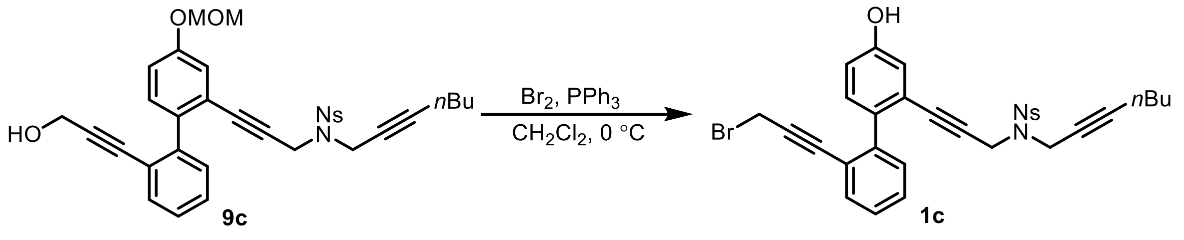

Br_2 (25 μL, 0.480 mmol) and PPh_3 (0.126 g, 0.480 mmol) were dissolved in CH_2Cl_2 (1 mL), and the mixture was stirred at 0 °C for 15 min. To this solution was added a solution of **9c** (0.120 g, 0.200 mmol) in CH_2Cl_2 (1 mL). The mixture was stirred at 0 °C for 1 h and then room temperature for 18 h. The resulting mixture was concentrated and purified by silica gel PTLC (eluent: *n*-hexane/EtOAc = 3:2) to give **1c** (0.0647 g, 0.104 mmol, 52% yield).

Red oil; ^{1}H NMR ($CDCl_3$, 400 MHz) δ 7.95–7.93 (m, 1H), 7.66–7.57 (m, 3H), 7.52–7.49 (m, 1H), 7.37–7.24 (m, 4H), 6.88–6.83 (m, 2H), 4.84 (s, 1H), 4.33 (s, 2H), 4.21 (s, 2H), 3.91–3.90 (m, 2H), 2.06–2.02 (m, 2H), 1.38–1.25 (m, 4H), 0.86 (t, J = 7.2 Hz, 3H); ^{13}C NMR ($CDCl_3$, 100 MHz) δ 154.7, 148.2, 142.6, 135.4, 133.6, 132.7, 132.7, 132.6, 131.6, 131.1, 130.4, 128.5, 127.2, 124.1, 122.5, 121.6, 118.8, 115.8, 87.1, 86.6, 86.3, 84.9, 84.6, 72.4, 37.4, 37.0, 30.5, 21.9, 18.3, 15.5, 13.6; HRMS (ESI) calcd for $C_{31}H_{27}BrN_2O_5SNa$ [M+Na]$^+$ 641.0716 found 641.0777.

N-(3-(2'-(3-Bromoprop-1-yn-1-yl)-4-hydroxy-[1,1'-biphenyl]-2-yl)prop-2-yn-1-yl)-4-methyl-N-(3-phenylprop-2-yn-1-yl)benzenesulfonamide (1d)

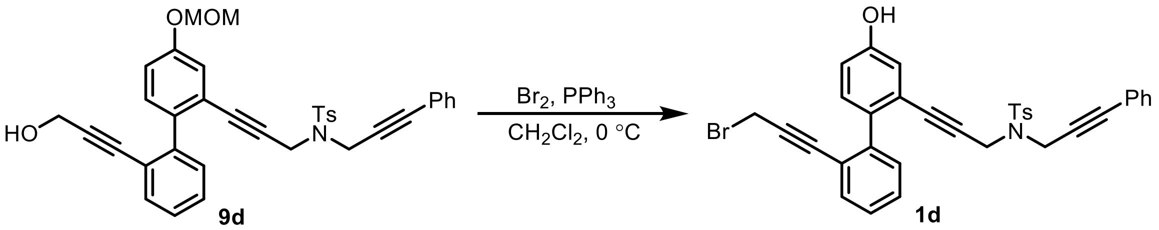

Br_2 (25 μL, 0.480 mmol) and PPh_3 (0.126 g, 0.480 mmol) were dissolved in CH_2Cl_2 (1 mL), and the mixture was stirred at 0 °C for 15 min. To this solution was added a solution of **9d** (0.118 g, 0.200 mmol) in CH_2Cl_2 (1 mL). The mixture was stirred at 0 °C for 1 h and then room temperature for 18 h. The resulting mixture was concentrated and purified by silica gel PTLC (eluent: *n*-hexane/EtOAc = 2:1) to give **1d** (0.0611 g, 0.100 mmol, 50% yield).

Red oil; ^{1}H NMR ($CDCl_3$, 400 MHz) δ 7.66 (d, J = 8.3 Hz, 2H), 7.48 (d, J = 7.6 Hz, 1H), 7.31–7.15 (m, 11H), 6.86 (dd, J = 8.4, 2.7 Hz, 1H), 6.81 (d, J = 2.6 Hz, 1H), 4.23 (s, 2H), 4.04 (s, 2H), 3.94 (s, 2H), 2.28 (s, 3H); ^{13}C NMR ($CDCl_3$, 100 MHz) δ 154.9, 144.0, 142.8, 135.2, 135.1, 132.7, 131.7, 131.4, 130.3, 129.6, 128.5, 128.4, 128.2, 127.8, 127.2, 122.6, 122.3, 121.7, 118.9, 115.8, 87.0, 86.3, 85.8, 85.0, 84.4, 81.6, 37.4, 37.1, 21.4, 15.5; HRMS (ESI) calcd for $C_{34}H_{26}BrNO_3SNa$ $[M+Na]^+$ 630.0709 found 630.0764.

2'-(3-Bromoprop-1-yn-1-yl)-2-((2'-(hex-1-yn-1-yl)-[1,1'-biphenyl]-2-yl)ethynyl)-[1,1'-biphenyl]-4-ol (1e)

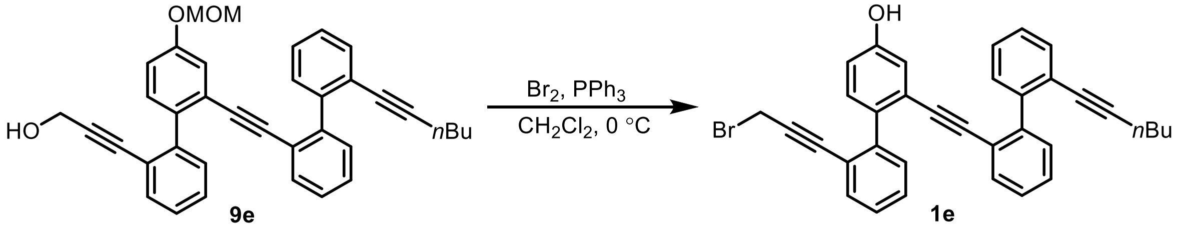

Br_2 (20 μL, 0.384 mmol) and PPh_3 (0.101 g, 0.384 mmol) were dissolved in CH_2Cl_2 (1 mL), and the mixture was stirred at 0 °C for 15 min. To this solution was added a solution of **9e** (0.0839 g, 0.160 mmol) in CH_2Cl_2 (1 mL). The mixture was stirred at 0 °C for 1 h and then room temperature for 18 h. The resulting mixture was concentrated and purified by silica gel PTLC (eluent: *n*-hexane/EtOAc = 3:1) to give **1e** (0.0447 g, 0.0822 mmol, 51% yield).

Red oil; ^{1}H NMR ($CDCl_3$, 400 MHz) δ 7.52–7.47 (m, 2H), 7.41–7.39 (m, 1H), 7.37–7.21 (m, 10H), 6.80 (dd, J = 8.4, 2.7 Hz, 1H), 6.69 (d, J = 2.6 Hz, 1H), 5.18 (s, 1H), 3.96 (s, 2H), 2.19 (t, J = 6.8 Hz, 2H), 1.35–1.15 (m, 6H), 0.78 (t, J = 7.3 Hz, 3H); ^{13}C NMR ($CDCl_3$, 100 MHz) δ154.4, 142.9, 142.7, 142.7, 134.9, 132.6, 132.2, 132.2, 131.7, 130.8, 130.3, 130.2, 128.1, 127.4, 127.2, 127.0, 127.0, 126.8, 123.9, 123.4, 122.6, 121.5, 118.6, 115.1, 93.6, 92.1, 91.2, 86.8, 86.6, 79.9, 30.5, 21.7, 19.1, 15.6, 13.6; HRMS (APCI) calcd for $C_{35}H_{28}BrO$ $[M+H]^+$ 543.1318 found 543.1308.

Hexayne 2a

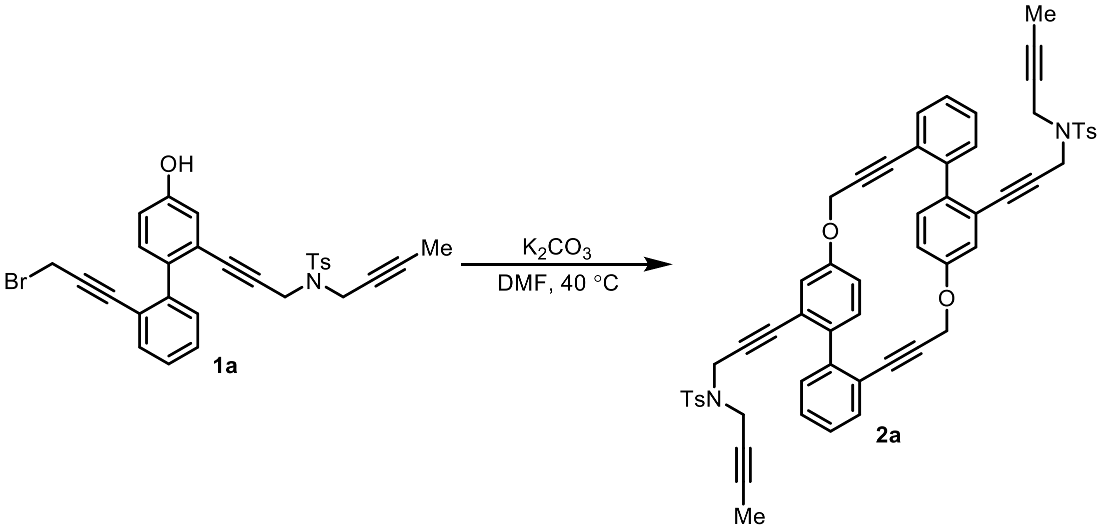

To a Schlenk tube was added a solution of **1a** (0.181 g, 0.332 mmol) and K_2CO_3 (0.229 g, 1.66 mmol) in DMF (15 mL) at room temperature. The mixture was stirred at 40 °C for 18 h. The reaction mixture was diluted with water and extracted with CH_2Cl_2 (100 mL x 3). The organic layer was washed with brine, dried with Na_2SO_4, filtered, and concentrated. The residue was purified by silica gel PTLC (eluent: *n*-hexane/EtOAc = 2:1) to give **2a** (0.0836 g, 0.0898 mmol, 54% yield). The melting point was determined using a sample, re-extracted from PTLC with ethyl acetate, concentrated, and solidified.

Pale yellow solid; mp 124.3–125.2 °C; ^{1}H NMR ($CDCl_3$, 400 MHz) δ 7.62 (d, J = 8.3 Hz, 4H), 7.52–7.50 (m, 2H), 7.31–7.28 (m, 4H), 7.22–7.16 (m, 8H), 6.82 (dd, J = 8.4, 2.7 Hz, 2H), 6.79 (d, J = 2.6 Hz, 2H), 4.80 (s, 3H), 4.16 (s, 4H), 3.78–3.77 (m, 4H), 2.32 (s, 6H), 1.59 (t, J = 2.3 Hz, 6H); ^{13}C NMR ($CDCl_3$, 100 MHz) δ 157.3, 143.6, 142.4, 135.8, 135.4, 131.9, 130.8, 130.4, 129.4, 128.1, 127.9, 127.2, 122.3, 121.7, 118.4, 115.0, 87.3, 87.1, 84.8, 84.6, 81.6, 71.6, 57.1, 37.1, 36.6, 21.5, 3.4; HRMS (ESI) calcd for $C_{58}H_{46}N_2O_6S_2Na$ $[M+Na]^+$ 953.2689 found 953.2700.

^{1}H NMR spectra of the crude (red, top) and purified (blue, bottom) products **2a** are shown in Figure S1 below.

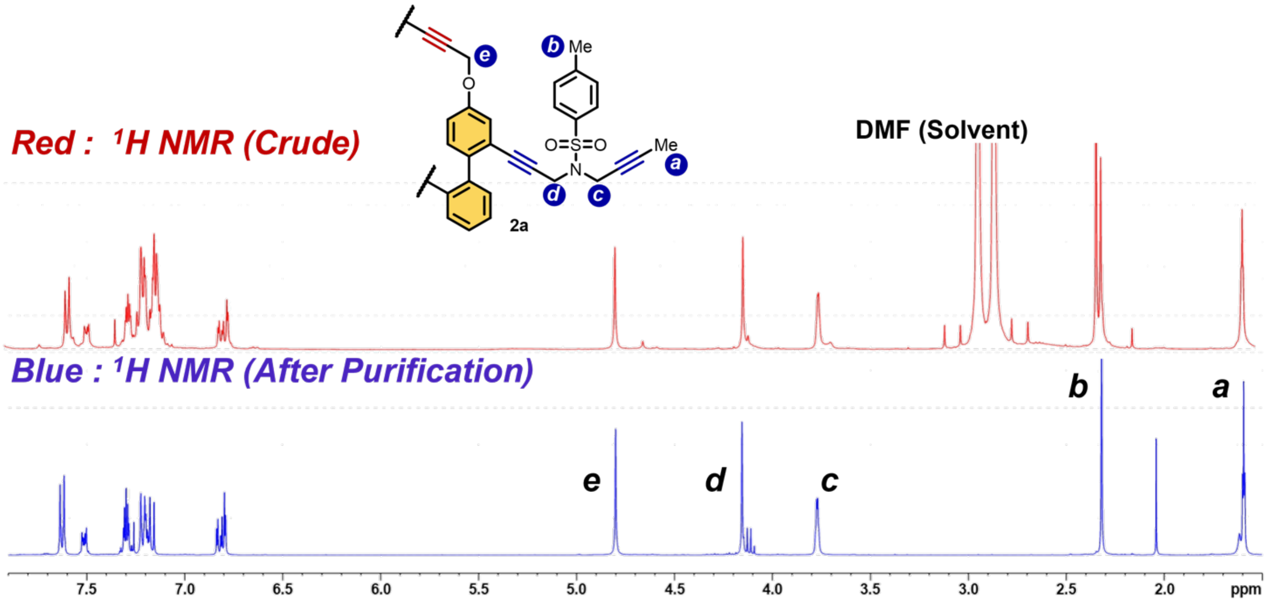

Figure S1. ^{1}H NMR spectra of the crude (red, top) and purified (blue, bottom) products **2a**.

Hexayne 2b

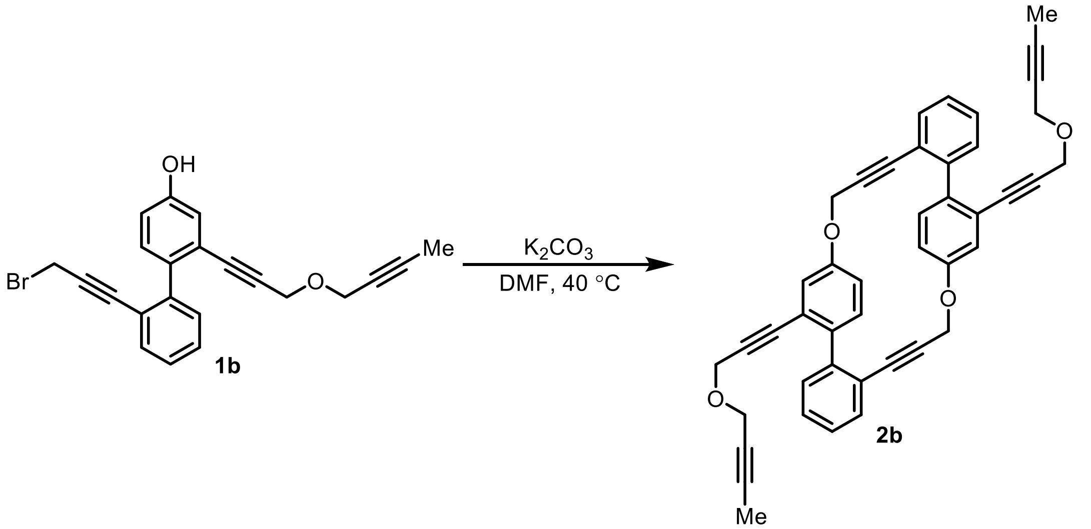

To a Schlenk tube was added a solution of **1b** (60.6 mg, 0.154 mmol) and K_2CO_3 (106 mg, 0.770 mmol) in DMF (6 mL) at room temperature. The mixture was stirred at 40 °C for 18 h. The reaction mixture was diluted with water and extracted with CH_2Cl_2 (100 mL x 3). The organic layer was washed with brine, dried with Na_2SO_4, filtered, and concentrated. The residue was purified by silica gel PTLC (eluent: *n*-hexane/EtOAc = 2:1) to give **2b** (25.2 mg, 0.0403 mmol, 52% yield). The melting point was determined using a sample, re-extracted from PTLC with ethyl acetate, concentrated, and solidified.

Pale yellow solid; mp 138.1–138.9 °C; ^{1}H NMR ($CDCl_3$, 400 MHz) δ 7.52 (d, J = 7.2 Hz, 2H), 7.37–7.30 (m, 6H), 7.21 (d, J = 8.6 Hz, 2H), 7.02 (d, J = 2.7 Hz, 2H), 6.83 (dd, J = 8.6, 2.7 Hz, 2H), 4.80 (s, 4H), 4.27 (s, 4H), 3.95 (q, J = 2.3 Hz, 4H), 1.84 (t, J = 2.3 Hz, 6H); ^{13}C NMR ($CDCl_3$, 100 MHz) δ 157.4, 142.7, 135.9, 131.6, 130.8, 130.5, 128.0, 127.1, 122.5, 121.9, 117.8, 115.4, 87.3, 87.2, 87.2, 85.9, 82.8, 74.6, 57.0, 57.0, 56.6, 3.6; HRMS (ESI) calcd for $C_{44}H_{32}O_4Na$ [M+Na]$^+$ 647.2193 found 647.2139.

Hexayne 2c

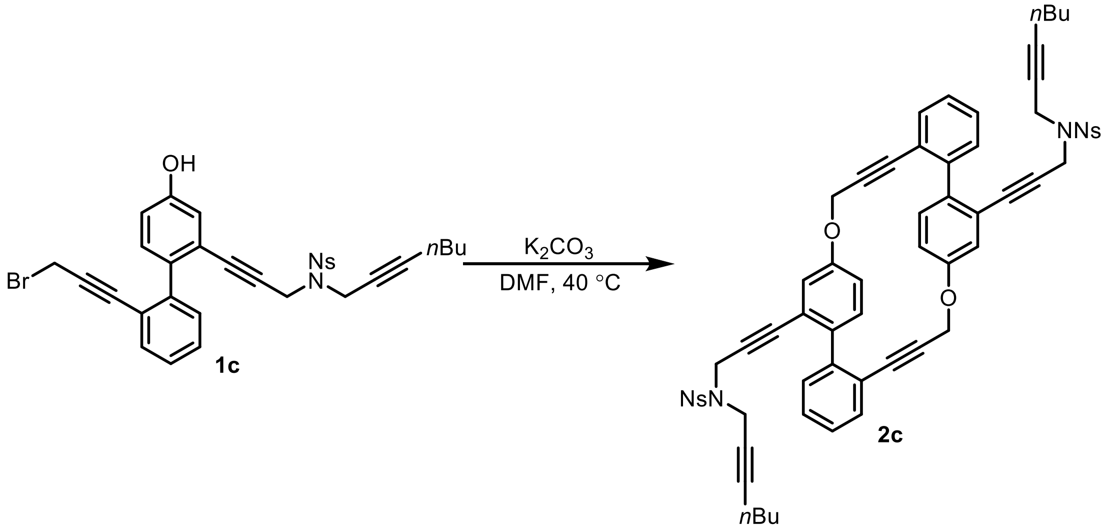

To a Schlenk tube was added a solution of **1c** (83.6 mg, 0.135 mmol) and K_2CO_3 (93.3 mg, 0.675 mmol) in DMF (5 mL) at room temperature. The mixture was stirred at 40 °C for 18 h. The reaction mixture was diluted with water and extracted with CH_2Cl_2 (100 mL x 3). The organic layer was washed with brine, dried with Na_2SO_4, filtered, and concentrated. The residue was purified by silica gel PTLC (eluent: *n*-hexane/EtOAc = 3:2) to give **2c** (41.0 mg, 0.0381 mmol, 56% yield). The melting point was determined using a sample, re-extracted from PTLC with ethyl acetate, concentrated, and solidified.

Pale yellow solid; mp 118.1–118.3 °C; ^{1}H NMR ($CDCl_3$, 600 MHz) δ 7.97–7.94 (m, 2H), 7.60–7.56 (m, 6H), 7.52–7.51 (m, 2H), 7.35–7.29 (m, 4H), 7.23 (dd, J = 5.0, 0.8 Hz, 2H), 7.17 (d, J = 5.7 Hz, 2H), 6.89 (d, J = 2.6 Hz, 2H), 6.83 (dd, J = 8.5, 2.7 Hz, 2H), 4.81 (s, 4H), 4.34 (s, 4H), 3.91 (s, 4H), 2.03–2.00 (m, 4H), 1.33–1.25 (m, 8H), 0.85 (t, J = 7.1 Hz, 6H); ^{13}C NMR ($CDCl_3$, 150 MHz) δ 157.4, 148.2, 142.4, 136.0, 133.5, 132.7, 131.9, 131.5, 131.1, 130.8, 130.3, 128.2, 127.2, 124.1, 122.1, 121.7, 117.9, 115.4, 87.3, 87.1, 86.5, 84.8, 84.7, 72.4, 57.1, 37.4, 36.8, 30.5, 21.8, 18.2, 13.6; HRMS (ESI) calcd for $C_{62}H_{52}N_4O_{10}S_2Na$ [M+Na]$^+$ 1099.3017 found 1099.3081.

Hexayne 2d

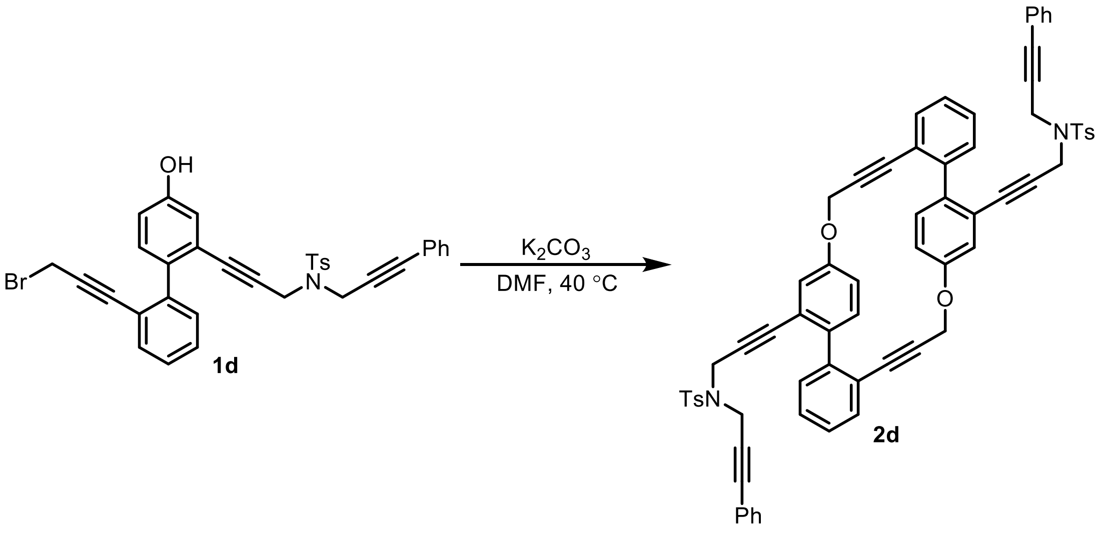

To a Schlenk tube was added a solution of **1d** (93.1 mg, 0.153 mmol) and K_2CO_3 (106 mg, 0.765 mmol) in DMF (6 mL) at room temperature. The mixture was stirred at 40 °C for 18 h. The reaction mixture was diluted with water and extracted with CH_2Cl_2 (100 mL x 3). The organic layer was washed with brine, dried with Na_2SO_4, filtered, and concentrated. The residue was purified by silica gel PTLC (eluent: *n*-hexane/EtOAc = 3:2) to give **2d** (46.3 mg, 0.0438 mmol, 57% yield). The melting point was determined using a sample, re-extracted from PTLC with ethyl acetate, concentrated, and solidified.

Pale yellow solid; mp 148.6–149.2 °C; ^{1}H NMR ($CDCl_3$, 400 MHz) δ 7.66 (d, J = 8.3 Hz, 4H), 7.51 (dd, J = 7.1, 1.7 Hz, 2H), 7.32–7.12 (m, 22H), 6.83–6.81 (m, 4H), 4.76 (s, 4H), 4.23 (s, 4H), 4.02 (s, 4H), 2.25 (s, 6H); ^{13}C NMR ($CDCl_3$, 100 MHz) δ 157.4, 143.8, 142.5, 135.9, 135.2, 131.8, 131.6, 130.7, 130.3, 129.6, 128.4, 128.2, 128.2, 127.8, 127.2, 122.3, 122.3, 121.8, 118.2, 115.2, 87.4, 87.1, 85.7, 85.1, 84.4, 81.7, 57.1, 37.4, 36.9, 21.4; HRMS (ESI) calcd for $C_{68}H_{50}N_2O_6S_2Na$ [M+Na]$^+$ 1077.3002 found 1077.3037.

Hexayne 2e

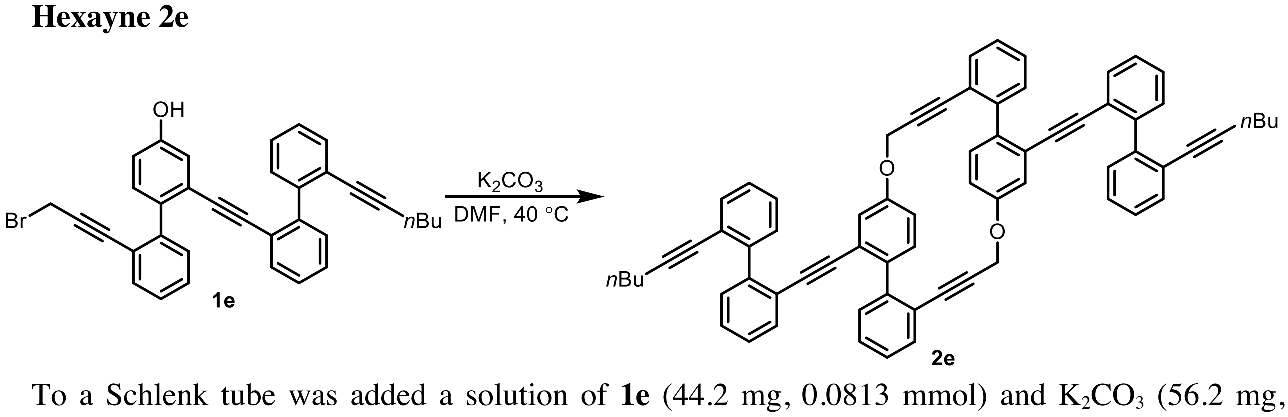

To a Schlenk tube was added a solution of **1e** (44.2 mg, 0.0813 mmol) and K_2CO_3 (56.2 mg, 0.407 mmol) in DMF (3 mL) at room temperature. The mixture was stirred at 40 °C for 18 h. The reaction mixture was diluted with water and extracted with CH_2Cl_2 (100 mL x 3). The organic layer was washed with brine, dried with Na_2SO_4, filtered, and concentrated. The residue was purified by

silica gel PTLC (eluent: *n*-hexane/EtOAc = 5:1) to give **2e** (18.9 mg, 0.0204 mmol, 50% yield). The melting point was determined using a sample, re-extracted from PTLC with ethyl acetate, concentrated, and solidified.

Pale yellow solid; mp 168.3–168.7 °C; ^{1}H NMR ($CDCl_3$, 400 MHz) δ 7.58–7.55 (m, 2H), 7.38–7.30 (m, 10H), 7.26–7.21 (m, 4H), 7.17–7.10 (m, 10H), 6.73 (dd, J = 8.5, 2.7 Hz, 2H), 6.68 (d, J = 2.6 Hz, 2H), 4.72 (s, 4H), 2.13 (t, J = 6.8 Hz, 4H), 1.32–1.10 (m, 12H), 0.75 (t, J = 7.2 Hz, 6H); ^{13}C NMR ($CDCl_3$, 100 MHz) δ 157.4, 142.9, 142.7, 142.5, 135.1, 132.1, 132.1, 131.8, 131.1, 130.9, 130.2, 130.1, 127.8, 127.3, 127.2, 127.0, 126.9, 126.8, 123.2, 123.2, 122.7, 121.8, 118.1, 115.1, 93.4, 91.7, 91.7, 87.4, 87.2, 80.0, 57.1, 30.4, 21.6, 19.1, 13.6; HRMS (ESI) calcd for $C_{70}H_{52}O_2Na$ $[M+Na]^+$ 947.3860 found 947.3901.

2-2. Stereoselective Synthesis of [2.2]Cyclophanes

[2.2]Triphenylenophane (+)-3a

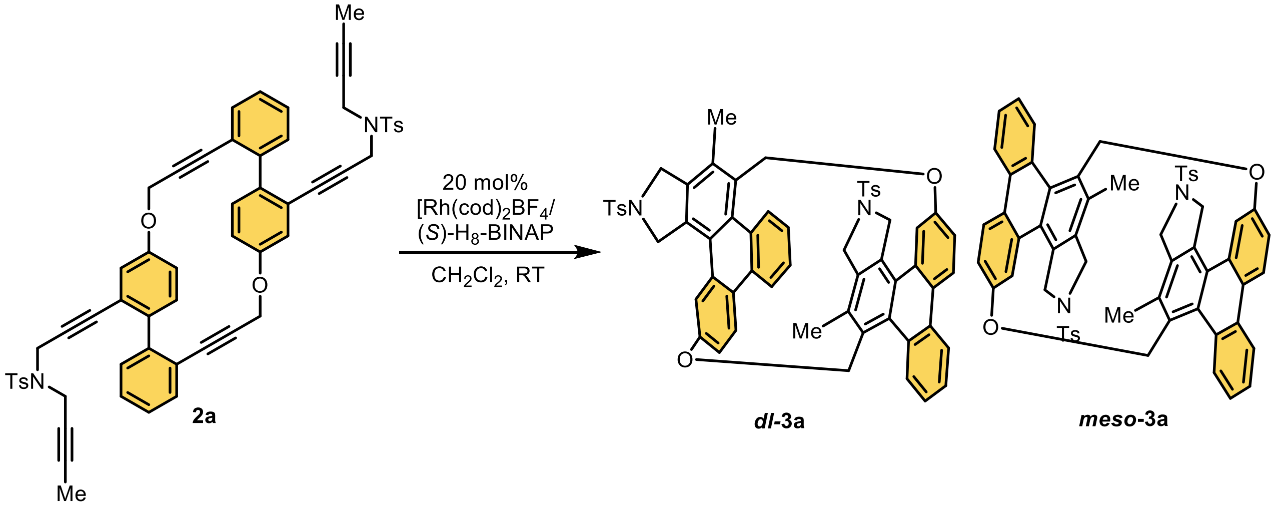

Table S1. Screening of reaction conditions for synthesis of [2.2]triphenylenophane **3a.**

Entry	**2a** [mg]	Ligand	Catalyst [mol%]	**3** / % yield (*dl* / *meso*, *er*)
1	5.24	biphep	80	**3a** / 87 (>20:1, –)
2	4.42	(*S*)-segphos	80	(+)-**3a** / 82 (>20:1, 84:16)
3	5.27	(*S*)-binap	80	(+)-**3a** / 84 (>20:1, 88:12)
4	4.37	(*S*)-H_8-binap	80	(+)-**3a** / 80 (>20:1, 93:7)
5	3.99	(*S*)-H_8-binap	80	(+)-**3a** / 80 (>20:1, 93:7)
6	3.83	(*S*)-xyl-H_8-binap	80	(+)-**3a** / 21 (>20:1, >99:1)
8	11.9	(*S*)-H_8-binap	20	(+)-**3a** / 84 (>20:1, 95:5)
7	102	biphep	20	(+)-**3a** / 80 (>20:1, –)

Ligand and $[Rh(cod)_2]BF_4$ were dissolved in CH_2Cl_2 (2.0 mL), and the mixture was stirred at room temperature for 10 min. H_2 was introduced to the resulting solution in a Schlenk tube. After stirring at room temperature for 30 min, the resulting mixture was concentrated under reduced pressure. The residue was dissolved in CH_2Cl_2 and added to a CH_2Cl_2 solution of **2a** (concentration of **2a**: 2.0 mmol/L). The mixture was stirred at room temperature for 3 h. The resulting mixture was concentrated and purified by silica gel PTLC (eluent: *n*-hexane/EtOAc = 3:1) to give **3a**.

Pale yellow solid; mp 239 °C (decomposition); $[\alpha]^{25}_D$ +289° [c 0.301 mg/cm^3, $CHCl_3$, *er* = 95:5]; ^{1}H NMR ($CDCl_3$, 400 MHz) δ 7.89 (d, J = 7.8 Hz, 2H), 7.73 (d, J = 8.2 Hz, 4H), 7.59 (d, J = 8.8 Hz, 2H), 7.40–7.34 (m, 2H), 7.32 (d, J = 6.8 Hz, 2H), 7.21 (d, J = 8.1 Hz, 4H), 6.63 (dd, J = 8.7, 1.8 Hz, 2H), 5.85 (d, J = 12.4 Hz, 2H), 5.77 (d, J = 12.3 Hz, 2H), 5.62 (d, J = 1.6 Hz, 2H), 4.76 (d, J = 13.9 Hz, 2H), 4.68 (d, J = 14.0 Hz, 2H), 4.63 (d, J = 13.2 Hz, 2H), 4.50 (d, J = 13.1 Hz, 2H), 2.75 (s, 6H), 2.29 (s, 6H); ^{13}C NMR ($CDCl_3$, 100 MHz) δ 155.4, 143.8, 136.4, 135.4, 134.6, 132.6, 131.3, 130.2, 130.0, 130.0, 128.4, 127.5, 126.9, 126.0, 125.2, 125.1, 124.2, 122.9, 121.0, 119.6, 72.8, 55.1, 52.2, 29.7, 21.4, 15.8; HRMS (ESI) calcd for $C_{58}H_{46}N_2O_6S_2Na$ $[M+Na]^+$ 953.2689 found 953.2632; CHIRALPAK IA, CH_2Cl_2/*n*-hexane = 90:10, 1.0 mL min^{-1}, 40 °C , retention times: 3.32 min (major isomer) and 6.49 min (minor isomer).

[2.2]Triphenylenophane (+)-3b

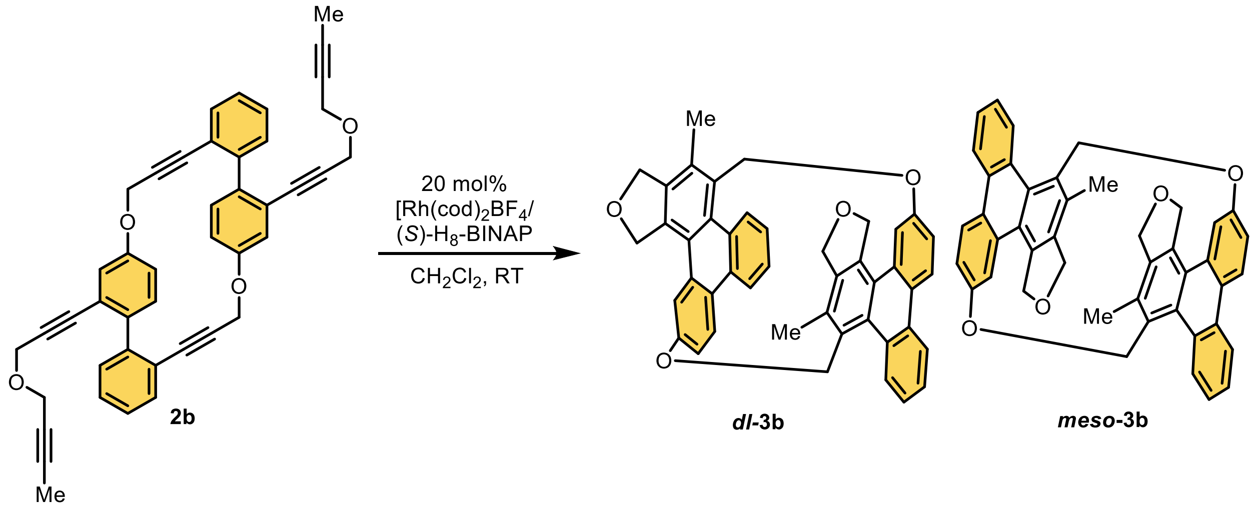

(*S*)-H_8-binap (2.1 mg, 0.0033 mmol) and [Rh(cod)$_2$]BF_4 (1.3 mg, 0.0033 mmol) were dissolved in CH_2Cl_2 (2.0 mL), and the mixture was stirred at room temperature for 10 min. H_2 was introduced to the resulting solution in a Schlenk tube. After stirring at room temperature for 30 min, the resulting mixture was concentrated under reduced pressure. The residue was dissolved in CH_2Cl_2 (5.0 mL) and added to a solution of **2b** (10.2 mg, 0.0163 mmol) in CH_2Cl_2 (1.0 mL). The mixture was stirred at room temperature for 3 h. The resulting mixture was concentrated and purified by silica gel PTLC (eluent: *n*-hexane/EtOAc = 2:1) to give **3b** (8.37 mg, 0.0134 mmol, 82% yield, *dl*/*meso* = >20:1, *er* = 93:7).

Pale yellow solid; mp 212 °C (decomposition); $[\alpha]^{25}_{D}$ +255° [c 0.221 mg/cm^3, $CHCl_3$, *er* = 93:7]; ^{1}H NMR ($CDCl_3$, 400 MHz) δ 7.96 (d, J = 7.9 Hz, 2H), 7.64 (d, J = 8.8 Hz, 2H), 7.44–7.33 (m, 6H), 6.68 (dd, J = 8.7, 2.2 Hz, 2H), 5.92 (d, J = 12.2 Hz, 2H), 5.84 (d, J = 12.0 Hz, 2H), 5.62–5.57 (m, 4H), 5.31 (dd, J = 12.6, 2.2 Hz, 2H), 5.25–5.16 (m, 4H), 2.82 (s, 6H); ^{13}C NMR ($CDCl_3$, 100 MHz) δ 155.7, 138.9, 135.3, 132.7, 132.0, 131.6, 130.3, 129.0, 128.8, 126.7, 126.1, 125.1, 124.7, 124.1, 123.0, 121.0, 120.0, 77.2, 75.3, 73.1, 72.8, 16.2; HRMS (ESI) calcd for $C_{44}H_{32}O_4Na$ [M+Na]$^+$ 647.2193 found 647.2194; CHIRALPAK IC, CH_2Cl_2/*n*-hexane = 90:10, 1.0 mL min^{-1}, 40 °C , retention times: 6.49 min (major isomer) and 8.05 min (minor isomer).

[2.2]Triphenylenophane (+)-3c

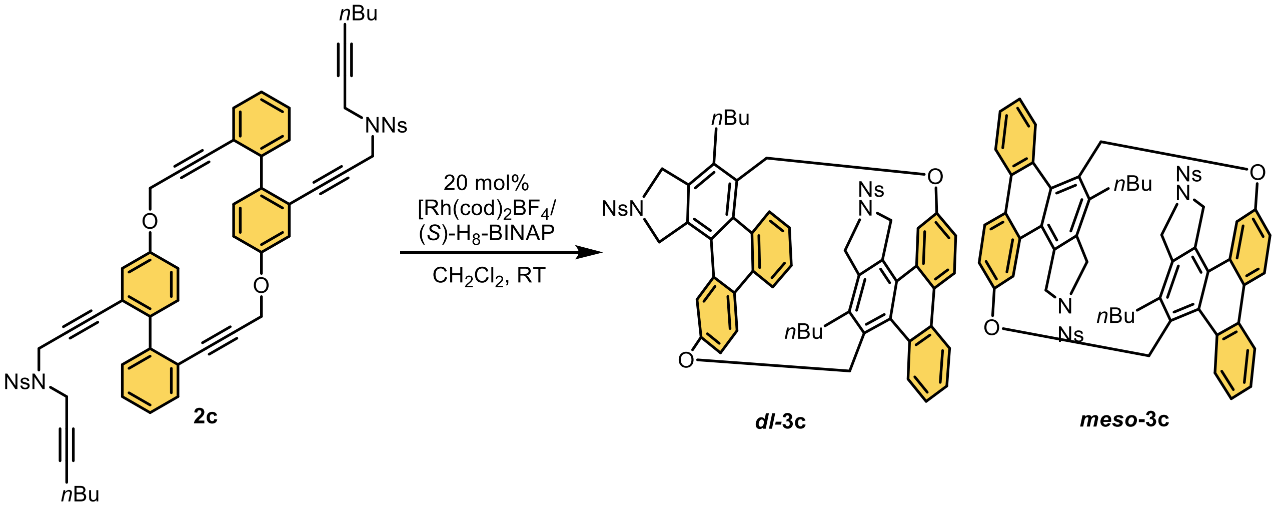

(*S*)-H_8-binap (1.0 mg, 0.0015 mmol) and [Rh(cod)$_2$]BF_4 (0.6 mg, 0.0015 mmol) were dissolved in CH_2Cl_2 (2.0 mL), and the mixture was stirred at room temperature for 10 min. H_2 was introduced to the resulting solution in a Schlenk tube. After stirring at room temperature for 30 min, the resulting mixture was concentrated under reduced pressure. The residue was dissolved in CH_2Cl_2 (2.0 mL) and added to a solution of **2c** (8.13 mg, 0.00755 mmol) in CH_2Cl_2 (1.0 mL). The mixture was stirred

at room temperature for 3 h. The resulting mixture was concentrated and purified by silica gel PTLC (eluent: *n*-hexane/EtOAc = 3:2) to give **3c** (6.67 mg, 0.00620 mmol, 82% yield, *dl*/*meso* = >20:1, *er* = 94:6).

Pale yellow solid; mp 201 °C (decomposition); $[\alpha]^{25}_{D}$ +246° [c 0.190 mg/cm^3, $CHCl_3$, 88% ee]; ^{1}H NMR ($CDCl_3$, 400 MHz) δ 8.11–8.09 (m, 2H), 7.94 (d, J = 7.8 Hz, 2H), 7.71–7.64 (m, 9H), 7.43–7.36 (m, 6H), 6.70 (dd, J = 8.7, 2.0 Hz, 2H), 5.89 (d, J = 12.2 Hz, 2H), 5.86 (d, J = 12.0 Hz, 2H), 5.76 (d, J = 2.0 Hz, 2H), 5.20 (d, J = 13.8 Hz, 2H), 4.94–4.88 (m, 3H), 4.80 (d, J = 12.2 Hz, 2H), 3.36–3.28 (m, 2H), 3.04–2.97 (m, 2H), 2.07–1.95 (m, 4H), 1.75–1.70 (m, 4H), 1.16 (t, J = 7.3 Hz, 6H); ^{13}C NMR ($CDCl_3$, 100 MHz) δ 155.6, 148.2, 138.3, 136.2, 135.6, 133.8, 132.2, 132.1, 131.5, 130.6, 130.5, 130.2, 129.6, 128.6, 128.4, 127.0, 126.3, 125.3, 124.4, 124.3, 123.0, 121.3, 120.0, 73.3, 55.2, 51.9, 33.4, 30.4, 23.5, 14.1, 1.0; HRMS (ESI) calcd for $C_{62}H_{52}N_4O_{10}S_2Na$ $[M+Na]^+$ 1099.3017 found 1099.3015; CHIRALPAK IC, CH_2Cl_2/*n*-hexane = 90:10, 1.0 mL min^{-1}, 40 °C , retention times: 4.53 min (minor isomer) and 4.96 min (major isomer).

[2.2]Triphenylenophane (+)-3d

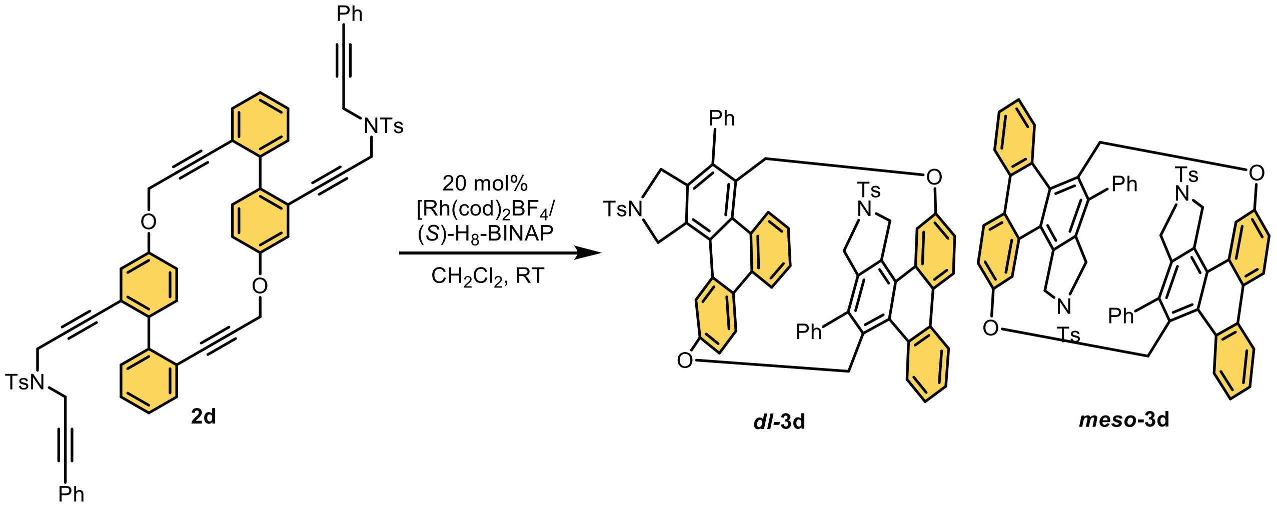

(*S*)-H_8-binap (1.8 mg, 0.0029 mmol) and $[Rh(cod)_2]BF_4$ (1.2 mg, 0.0029 mmol) were dissolved in CH_2Cl_2 (2.0 mL), and the mixture was stirred at room temperature for 10 min. H_2 was introduced to the resulting solution in a Schlenk tube. After stirring at room temperature for 30 min, the resulting mixture was concentrated under reduced pressure. The residue was dissolved in CH_2Cl_2 (4.0 mL) and added to a solution of **2d** (15.4 mg, 0.0146 mmol) in CH_2Cl_2 (1.0 mL). The mixture was stirred at room temperature for 3 h. The resulting mixture was concentrated but was not soluble in any solvents, and thus the desired product could not be isolated.

[2.2][5]Helicenophane (±)-3e

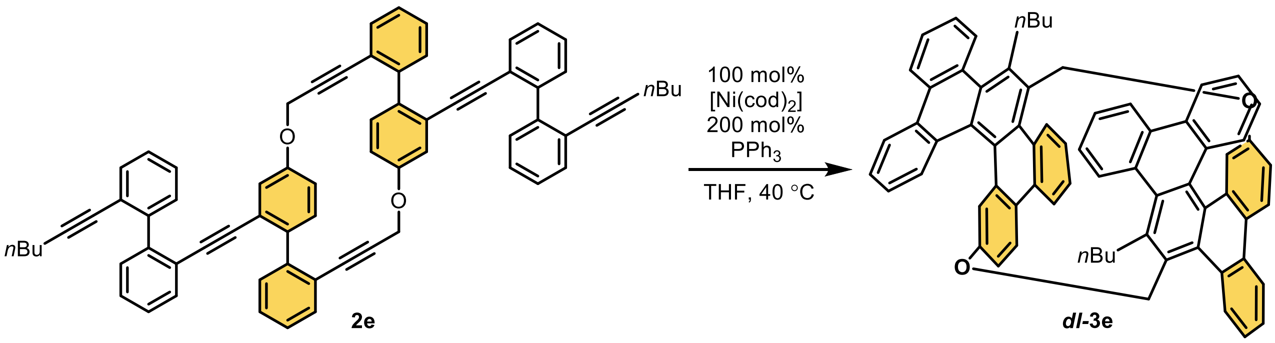

2e ***dl*-3e**

PPh_3 (6.9 mg, 0.026 mmol) and [Ni(cod)$_2$] (3.6 mg, 0.013 mmol) were dissolved in THF (4.0 mL), and the mixture was stirred at room temperature for 10 min. To this solution was added a solution of **2e** (12.2 mg, 0.0132 mmol) in THF (1.0 mL). The mixture was stirred at 40 °C for 16 h. The resulting mixture was concentrated and purified by silica gel PTLC (eluent: *n*-hexane/EtOAc = 5:1) to give **3e** (10.2 mg, 0.0110 mmol, 83% yield, *dl*/*meso* = >20:1).

Pale yellow solid; mp 249 °C (decomposition); ^{1}H NMR ($CDCl_3$, 400 MHz) δ 8.30 (d, J = 8.0 Hz, 2H), 8.22–8.20 (m, 2H), 7.99–7.97 (m, 2H), 7.94 (d, J = 7.2 Hz, 2H), 7.89 (d, J = 8.2 Hz, 2H), 7.73 (d, J = 8.8 Hz, 2H), 7.48–7.36 (m, 2H), 6.94–6.90 (m, 2H), 6.12 (d, J = 14.4 Hz, 2H), 5.91 (dd, J = 8.8, 2.5 Hz, 2H), 5.43 (d, J = 2.3 Hz, 2H), 5.31 (d, J = 14.4 Hz, 2H), 2.04–1.60 (m, 12H), 1.20 (t, J = 7.3 Hz, 6H); ^{13}C NMR ($CDCl_3$, 100 MHz) δ 151.9, 136.5, 134.0, 132.3, 131.4, 131.0, 130.7, 130.5, 130.4, 130.3, 129.8, 129.6, 129.1, 128.9, 128.8, 128.3, 128.2, 127.9, 126.9, 126.6, 126.5, 125.1, 124.6, 123.8, 122.6, 122.6, 121.9, 121.7, 118.0, 115.9, 68.7, 34.3, 33.6, 23.2, 14.1; HRMS (ESI) calcd for $C_{70}H_{52}O_2Na$ $[M+Na]^+$ 947.3860 found 947.3912; CHIRALPAK IE, CH_2Cl_2/*n*-hexane = 50:50, 1.0 mL min^{-1}, 40 °C , retention times: 4.78 min and 8.09 min.

3. ECD Spectra

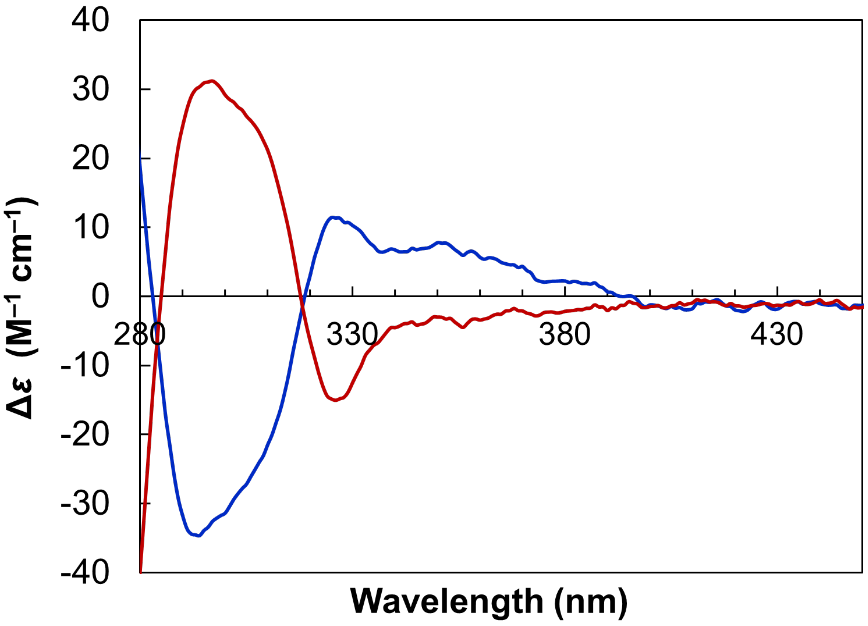

Figure S2. ECD spectra of (+)-**3a** (blue) and (–)-**3a** (red) in $CHCl_3$ 1.0×10^{-5} M at 25 °C.

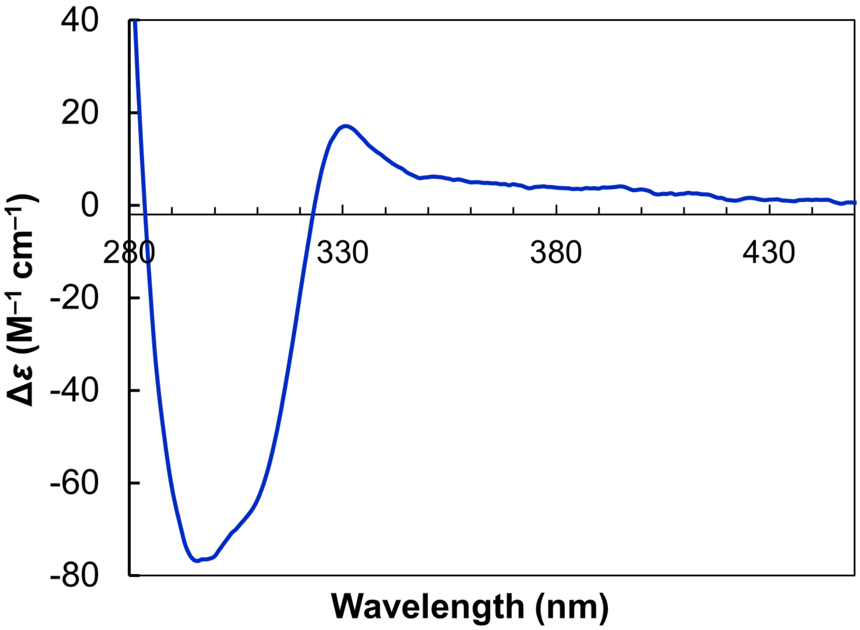

Figure S3. ECD spectra of (+)-**3c** (blue) in $CHCl_3$ 1.0×10^{-5} M at 25 °C.

4. Crystal Data

Single crystals of (±)-**3a** and (±)-**3e** suitable for X-ray crystallographic analyses were obtained by recrystallization from CH_2Cl_2/hexane solutions at room temperature.

Table S2. Crystal data and data collection parameters of [2.2]Triphenylenophane (±)-**3a.**

[2.2]Triphenylenophane (±)-**3a** / CCDC 2237885	
Empirical formula	$C_{58}H_{46}N_2O_6S_2$
Formula weight	931.09
Colour	clear light yellow
Shape	plate
Temperature / K	293.15
Wavelength / Å	0.71073
Crystal system	Monoclinic
Space group	$P2_1/c$
	a = 13.3238(6) Å α = 90°
Unit cell dimensions	b = 20.1104(7) Å β = 96.463(4)°
	c = 16.9292(7) Å γ = 90°
Volume / Å^3	4507.3(3)
Z	4
D_{calc} / g cm^{-3}	1.372
μ (Mo Kα) / mm^{-1}	0.177
F(000)	1952.0
Crystal size / mm^3	0.418 × 0.185 × 0.087
Theta range for data collection / °	4.594 to 61.208
Index ranges	-18<=h<=19, -28<=k<=27, -24<=l<=23
Reflections collected	44991
Independent reflections	13167 [R(int) = 0.1102, R(sigma) = 0.1103]
Data / restraints / parameters	13167 / 0 / 617
Goodness-of-fit on F^2	0.975
Final R indices [I>2sigma(I)]	R_1 = 0.0633, wR_2 = 0.1511
R indices (all data)	R_1 = 0.1235, wR_2 = 0.1810
Largest diff. peak / hole / eÅ^{-3}	0.46 / -0.54

Table S3. Crystal data and data collection parameters of [2.2]Helicenophane (±)-**3e.**

[2.2]Helicenophane (±)-**3e** / CCDC 2237886	
Empirical formula	$C_{70}H_{52}O_2$
Formula weight	925.11
Colour	clear light yellow
Shape	plate
Temperature / K	210
Wavelength / Å	0.71073
Crystal system	Monoclinic
Space group	$P2_1/c$
Unit cell dimensions	a = 12.9227(4) Å α = 90°<br>b = 24.0368(7) Å β = 97.506(3)°<br>c = 17.1320(5) Å γ = 90°
Volume / $Å^3$	5275.9(3)
Z	4
D_{calc} / g cm^{-3}	1.165
μ (Mo Kα) / mm^{-1}	0.068
F(000)	1952.0
Crystal size / mm^3	0.345 × 0.193 × 0.172
Theta range for data collection / °	4.648 to 61.306
Index ranges	-13<=h<=18, -34<=k<=32, -24<=l<=24
Reflections collected	55196
Independent reflections	15579 [R(int) = 0.0491, R(sigma) = 0.0992]
Data / restraints / parameters	15579 / 3 / 671
Goodness-of-fit on F^2	0.986
Final R indices [I>2sigma(I)]	R_1 = 0.0765, wR_2 = 0.1708
R indices (all data)	R_1 = 0.1502, wR_2 = 0.1958
Largest diff. peak / hole / e$Å^{-3}$	0.32 / -0.29

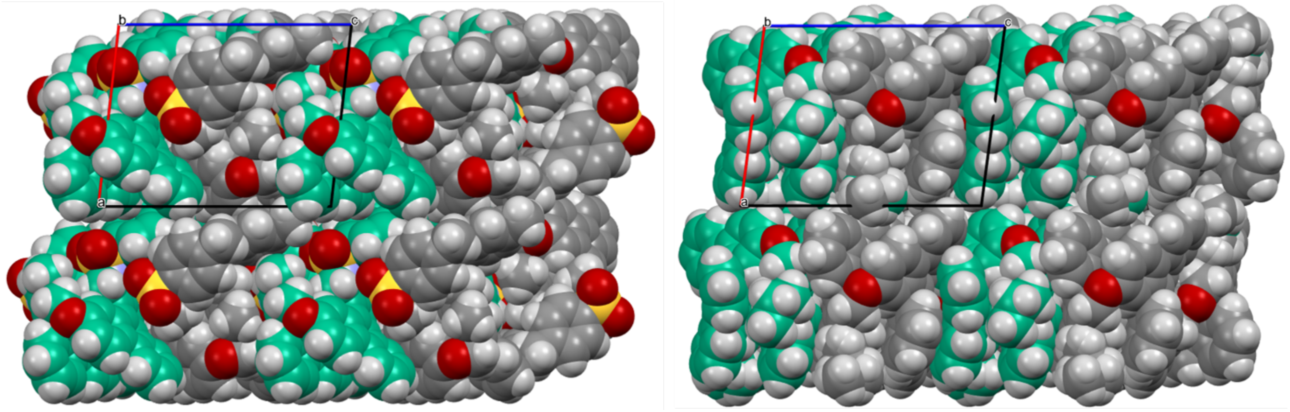

Figure S4. Packing structure of [2.2]triphenylenophane (±)-**3a** (left) and [2.2][5]helicenophane (±)-**3e** (right).

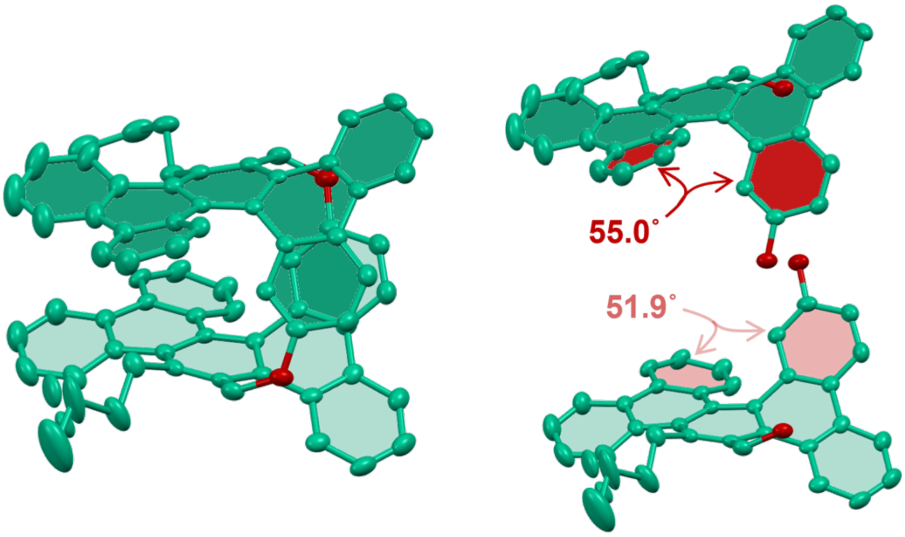

Figure S5. Dihedral angles of [2.2]helicenophane (±)-**3e**, calculated from the mean plane through the six carbons of the benzene ring (shown in red).

5. Theoretical Calculations

All calculations were carried out using the Gaussian 16 program.[10] The hybrid density functional method based on B3LYP[11,12] with 6-31g(d) basis set or M06[13] with a 6-31g(d) basis set (LANL2DZ basis set for Rh) was used for geometry optimizations.

Harmonic vibrational analysis at the same level as optimization was performed to confirm the number of imaginary frequencies for all stationary points (0 for minima and 1 for TSs). The intrinsic reaction coordinate (IRC) method was used to track minimum energy paths from transition structures to the corresponding local minima.

Excitation wavelengths, oscillator strengths, and rotatory strengths were obtained at the density functional level using the time-dependent perturbation theory (TD-DFT) approach. The half-width of the simulated ECD spectrum of (*R*p,*R*p)-**3b** and (*R*p,*R*p)-**3e** are 0.12 eV and 0.15 eV, respectively.

Strain energies of (*R*p,*R*p)-**3b** and (*R*p,*R*p)-**3e** in Figure 5c were evaluated by the homodesmotic reaction method according to the reported procedure[14]. Strain distribution of (*R*p,*R*p)-**3b** in Figure 5d was calculated using the StrainViz program[15] developed by the Jasti group and VMD (v1.9.3) program[16] for visualization. The fragments for StrainViz were prepared from the B3LYP/6-31G(d) optimized structure of **3b**. The total strain (12.2 kcal/mol) was comparable to the strain of the homodesmotic reaction (10.2 kcal/mol). The data of [2.2]paracyclophane in Figure 5d is according to the literature.[17]

Table S4. The Gibbs-free energies, enthalpies, and imaginary frequencies.

compound	*G* (hartree)	*H* (hartree)	imaginary frequency (cm^{-1})
Strain energy			
(*R*p,*R*p)-**3b**	-1996.259503	-1996.159437	
(*R*p,*R*p)-**3e**	-2612.628452	-2612.505928	
**Fragment 1**	-577.647196	-577.596146	
**Fragment 2**	-1575.780806	-1575.684019	
**Fragment 3**	-1883.968937	-1883.861747	
Mechanism study			
(*R*p,*R*a)-**IM0**	-1994.668125		
$\mathbf{TS_{rot}}$	-1994.650844		-30.2870
(*R*p,*S*a)-**IM0**	-1994.670345		
**IM1**	-4485.457055		
**TS1**	-4485.440502		-335.6768
**IM2**	-4485.475079		
$Rh(I)^+$/(*S*)-H_8-BINAP	-2490.800504		

Excitation wavelengths and oscillator strengths

Table S5. TD-DFT vertical one-electron excitations calculated for (*R*p,*R*p)-[2.2]triphenylenophane **3b**.

exited state	energy (eV)	wavelength (nm)	oscillator strength (*f*)[a]	rotational strength (*R*)	description [b]
1	3.6770	337.18	0.0006	–10.0476	H→L (52%), H–1→L+1 (19%)
20	4.5075	275.06	0.2945	–52.9582	H–5→L+1 (16%), H–4→L (26%), H–3→L+5 (11%)
23	4.5728	271.13	0.2298	198.3661	H–4→L (22%), H–4→L+3 (13%), H–3→L+5 (11%)
32	4.8446	255.92	0.3869	–225.1497	H–6→L+1 (10%), H–5→L+3 (20%), H–2→L+5 (20%)

H = HOMO, L = LUMO. [a] Excitation energies with oscillator strength larger than 0.2 are listed. [b] Relative contribution larger than 10% is listed.

Table S6. TD-DFT vertical one-electron excitations calculated for (*P*,*P*,*R*p,*R*p)-[2.2][5]helicenophane **3e**.

exited state	energy (eV)	wavelength (nm)	oscillator strength (*f*)[a]	rotational strength (*R*)	description [b]
1	3.1617	392.15	0.0176	24.9492	H–3→L (10%), H–2→L (24%), H–1→L (15%), H→L (13%), H→L+2 (13%)
14	3.6903	335.97	0.1347	–26.3705	H–3→L+2 (11%), H–2→L+3 (62%)
20	3.8464	322.34	0.2146	–144.3314	H–6→L (10%), H–5→L+1 (16%), H–3→L+2 (23%)
21	3.8625	320.99	0.1558	–185.9287	H–5→L (25%), H–3→L+2 (16%), H–3→L+3 (16%)
22	3.8991	317.98	0.1369	61.0935	H–6→L+1 (15%), H–5→L+1 (31%)
28	4.0852	303.50	0.1047	–47.0089	H–1→L+5 (11%), H→L+4 (26%)

H = HOMO, L = LUMO. [a] Excitation energies with oscillator strength larger than 0.1 are listed. [b] Relative contribution larger than 10% is listed.

DFT optimized structure

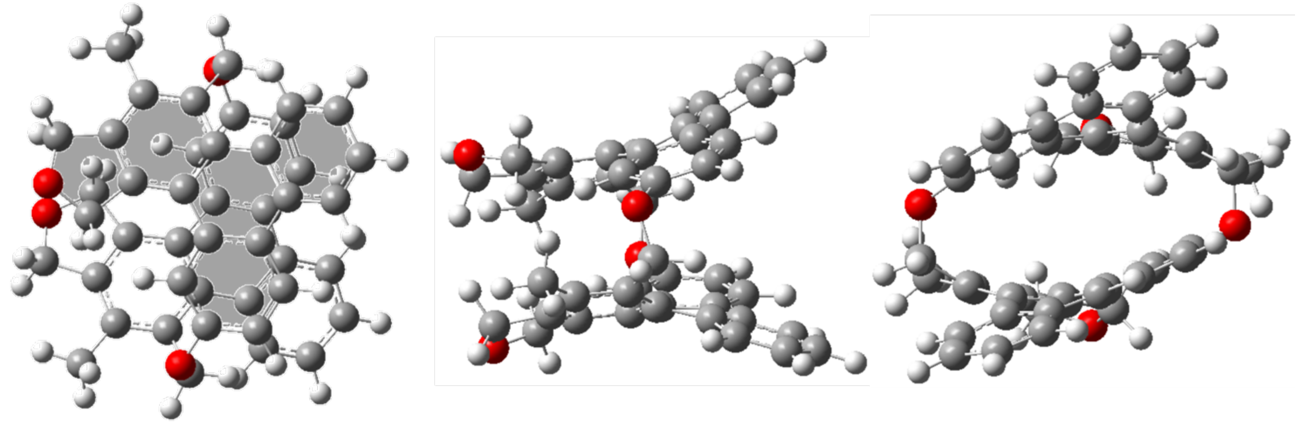

Figure S6. DFT-optimized structure of [2.2]triphenylenophane **3b**

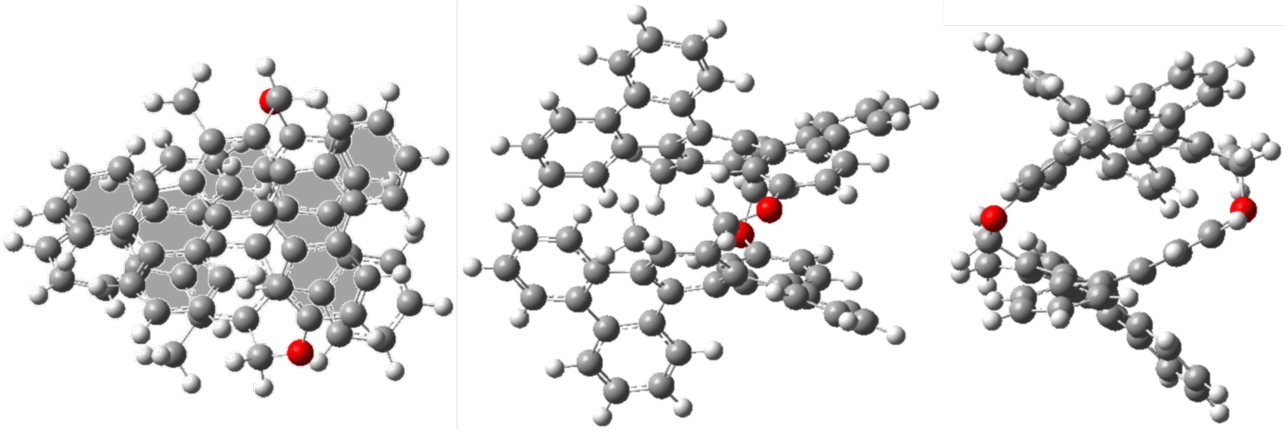

Figure S7. DFT-optimized structure of [2.2][5]helicenophane **3e**

Optimized Geometries

[2.2]Triphenylenophane (*R*p,*R*p)-3b

Sum of electronic and zero-point Energies=
-1996.196211
Sum of electronic and thermal Energies=
-1996.160381
Sum of electronic and thermal Enthalpies=
-1996.159437
Sum of electronic and thermal Free Energies=
-1996.259503

C	-3.47781	2.591989	-2.06724
C	-4.02285	2.546995	-3.34135
C	-3.90751	1.378334	-4.10359
C	-3.27834	0.267166	-3.56371
C	-2.71187	0.293281	-2.27369
C	-2.77361	1.496057	-1.52013
C	-2.08781	-0.8872	-1.67605
C	-1.86793	-0.91641	-0.27291
C	-2.12195	0.284147	0.53507
C	-2.26726	1.530859	-0.14197
C	-2.10773	0.323783	1.942106
C	-2.10174	1.536348	2.62245
C	-1.92969	2.755512	1.956823
C	-1.98166	2.743518	0.552008
C	-1.67354	-1.98839	-2.45121
C	-1.00969	-3.06812	-1.89171
C	-1.21156	-2.03364	0.281051
C	-2.14176	-0.77609	2.985212
O	-2.52887	-0.12413	4.193579
C	-2.1895	1.25869	4.104801
O	0.002517	-4.08308	0.055676
C	-1.43514	3.958419	-0.18363
C	3.477814	-2.59199	-2.06724
C	4.022849	-2.547	-3.34135
C	3.907509	-1.37833	-4.10359
C	3.278342	-0.26717	-3.56371
C	2.711868	-0.29328	-2.27369
C	2.773607	-1.49606	-1.52013
C	2.087813	0.887199	-1.67605
C	1.867927	0.916406	-0.27291
C	2.101736	-1.53635	2.62245
C	1.981662	-2.74352	0.552008
C	1.673541	1.988387	-2.45121
C	1.009687	3.06812	-1.89171
C	0.750473	3.072024	-0.51506
C	1.211558	2.033635	0.281051
C	2.141759	0.776091	2.985212
C	2.1895	-1.25869	4.104801
O	-0.00252	4.083075	0.055676
C	1.435136	-3.95842	-0.18363
C	2.267262	-1.53086	-0.14197
C	1.929688	-2.75551	1.956823
C	-0.75047	-3.07202	-0.51506
O	2.528866	0.124128	4.193579
C	2.107728	-0.32378	1.942106
C	2.12195	-0.28415	0.53507
C	-1.67354	4.029084	2.734504
C	1.673541	-4.02908	2.734504
H	-3.65194	3.469382	-1.45368
H	-4.56595	3.404606	-3.72871
H	-4.34394	1.325134	-5.09719
H	-3.25801	-0.65302	-4.13795
H	-1.82447	-1.97451	-3.52526
H	-0.66114	-3.89037	-2.50941
H	-0.94633	-2.06101	1.32739
H	-2.86551	-1.571	2.772375
H	-1.15226	-1.24715	3.118462
H	-2.97125	1.834695	4.614891
H	-1.23472	1.46124	4.622555
H	-1.59814	3.900711	-1.25955
H	-1.84898	4.900881	0.183196
H	3.651939	-3.46938	-1.45368
H	4.56595	-3.40461	-3.72871
H	4.343941	-1.32513	-5.09719
H	3.258008	0.653023	-4.13795
H	1.824472	1.974513	-3.52526
H	0.66114	3.890369	-2.50941
H	0.946326	2.061012	1.32739
H	2.865509	1.571001	2.772375
H	1.152257	1.247148	3.118462
H	2.971245	-1.8347	4.614891
H	1.234719	-1.46124	4.622555
H	1.59814	-3.90071	-1.25955
H	1.848983	-4.90088	0.183196
H	-2.44206	4.786732	2.534078
H	-1.67033	3.846068	3.812329
H	-0.70832	4.467688	2.461802
H	2.442061	-4.78673	2.534078
H	1.670333	-3.84607	3.812329
H	0.708321	-4.46769	2.461802

[2.2][5]Helicenophane (*R*p,*R*p)-3e

Sum of electronic and zero-point Energies=
-2612.553192
Sum of electronic and thermal Energies=
-2612.506872
Sum of electronic and thermal Enthalpies=
-2612.505928
Sum of electronic and thermal Free Energies=
-2612.628452

C	4.358325	-0.4944	-3.32081
C	4.694959	-0.27861	-4.64959
C	3.917708	0.583563	-5.43318
C	2.831513	1.235949	-4.86795
C	2.470806	1.02808	-3.52326
C	3.229207	0.120512	-2.73779
C	1.337518	1.700717	-2.89339
C	1.276516	1.756145	-1.47566
C	2.295645	1.061243	-0.66798
C	2.938647	-0.02075	-1.30638
C	2.56271	1.26977	0.724507
C	3.0925	0.197291	1.493491
C	3.226911	-1.09198	0.873582
C	3.243347	-1.17188	-0.52068
C	0.277782	2.247186	-3.6447
C	-0.84161	2.788316	-3.0371
C	0.111517	2.277433	-0.87309
C	2.369053	2.579711	1.361023
C	3.386858	0.42381	2.913938
O	-2.04762	3.271252	-0.9898
C	3.284757	-2.54087	-1.19012
C	-4.35833	0.494395	-3.32081
C	-4.69496	0.278605	-4.64959
C	-3.91771	-0.58356	-5.43318
C	-2.83151	-1.23595	-4.86795
C	-2.47081	-1.02808	-3.52326
C	-3.22921	-0.12051	-2.73779
C	-1.33752	-1.70072	-2.89339
C	-1.27652	-1.75615	-1.47566
C	-3.0925	-0.19729	1.493491
C	-3.24335	1.171876	-0.52068
C	-0.27778	-2.24719	-3.6447
C	0.841609	-2.78832	-3.0371
C	0.93348	-2.77346	-1.63618
C	-0.11152	-2.27743	-0.87309
C	-2.36905	-2.57971	1.361023
C	-3.38686	-0.42381	2.913938
O	2.047622	-3.27125	-0.9898
C	-3.28476	2.540869	-1.19012
C	-2.93865	0.02075	-1.30638
C	-3.22691	1.091976	0.873582
C	-0.93348	2.773464	-1.63618
C	-2.56271	-1.26977	0.724507
C	-2.29565	-1.06124	-0.66798
C	-2.86625	-1.57026	3.577815
C	-3.00493	-1.66579	4.979856
C	-3.72639	-0.73455	5.70606
C	-4.37982	0.304513	5.03182
C	-4.21076	0.447788	3.664919
C	-2.24748	-3.76895	0.604118
C	-1.97912	-4.98797	1.199134
C	-1.83334	-5.06393	2.591601
C	-2.04762	-3.93518	3.361425
C	-2.3665	-2.68783	2.777467
C	2.247484	3.76895	0.604118
C	1.979117	4.987974	1.199134
C	1.83334	5.063931	2.591601
C	2.047622	3.935177	3.361425
C	2.366497	2.687831	2.777467
C	2.866245	1.570257	3.577815
C	3.004931	1.665792	4.979856
C	3.726386	0.734554	5.70606
C	4.379815	-0.30451	5.03182
C	4.210756	-0.44779	3.664919
C	3.258016	-2.38604	1.671427
C	-3.25802	2.386036	1.671427
H	5.012767	-1.0997	-2.70164
H	5.577562	-0.75446	-5.06814
H	4.182207	0.769231	-6.47062
H	2.270038	1.945499	-5.46759
H	0.313828	2.203457	-4.72836
H	-1.65447	3.192738	-3.63228
H	-0.00466	2.259335	0.202732

H	3.473011	-2.46153	-2.25968
H	4.048674	-3.18922	-0.75513
H	-5.01277	1.099703	-2.70164
H	-5.57756	0.75446	-5.06814
H	-4.18221	-0.76923	-6.47062
H	-2.27004	-1.9455	-5.46759
H	-0.31383	-2.20346	-4.72836
H	1.654469	-3.19274	-3.63228
H	0.00466	-2.25934	0.202732
H	-3.47301	2.461526	-2.25968
H	-4.04867	3.189223	-0.75513
H	-2.56503	-2.50365	5.508271
H	-3.82032	-0.83938	6.783472
H	-5.0193	0.996591	5.572893
H	-4.75059	1.235577	3.1588
H	-2.3698	-3.72687	-0.46998
H	-1.88738	-5.88021	0.586187
H	-1.59932	-6.01096	3.07028
H	-2.00788	-4.03165	4.44014
H	2.369795	3.726866	-0.46998
H	1.887381	5.88021	0.586187
H	1.599322	6.010955	3.07028
H	2.007875	4.031648	4.44014
H	2.565025	2.503645	5.508271
H	3.820316	0.839381	6.783472
H	5.019298	-0.99659	5.572893
H	4.750587	-1.23558	3.1588
H	2.847811	-2.25023	2.672839
H	4.270887	-2.79749	1.784204
H	2.656543	-3.14279	1.166078
H	-2.84781	2.250225	2.672839
H	-4.27089	2.797492	1.784204
H	-2.65654	3.142794	1.166078

Fragment 1

Sum of electronic and zero-point Energies=
-577.608246
Sum of electronic and thermal Energies=
-577.597090
Sum of electronic and thermal Enthalpies=
-577.596146
Sum of electronic and thermal Free Energies=
-577.647196

C	0.496815	1.370957	-1.05436
O	-0.65559	1.581786	-0.24332
C	-1.58814	0.58154	-0.12933
C	-2.76528	0.936915	0.544273
C	-3.78197	0.002271	0.709034
C	-3.64212	-1.29526	0.205566
C	-2.46813	-1.64291	-0.45912
C	-1.4349	-0.71608	-0.63007
C	1.58882	0.552463	-0.39062
C	2.470677	-0.20271	-1.17224
C	3.518909	-0.90893	-0.57989
C	3.690073	-0.87352	0.804815
C	2.809743	-0.12672	1.590802
C	1.767668	0.585503	0.996891
H	0.210493	0.926741	-2.01741
H	0.862086	2.38408	-1.25811
H	-2.86007	1.950304	0.922248
H	-4.69109	0.289997	1.230771
H	-4.43745	-2.02366	0.334308
H	-2.34063	-2.64973	-0.84857
H	-0.5201	-1.01829	-1.12593
H	2.337231	-0.24008	-2.25193
H	4.195152	-1.49243	-1.19919
H	4.501559	-1.4275	1.269035
H	2.934706	-0.09827	2.670116
H	1.079789	1.163478	1.606671

Fragment 2

Sum of electronic and zero-point Energies=
-1575.715525
Sum of electronic and thermal Energies=
-1575.684963
Sum of electronic and thermal Enthalpies=
-1575.684019
Sum of electronic and thermal Free Energies=
-1575.780806

C	2.786447	-2.40079	-1.52894
C	2.927397	-3.7752	-1.63887
C	1.879955	-4.6108	-1.23465
C	0.695423	-4.05165	-0.7835
C	0.52876	-2.65536	-0.6695
C	1.622083	-1.80772	-0.98824
C	-0.75768	-2.05856	-0.31497
C	-0.97023	-0.66589	-0.53283
C	1.165596	2.350979	-1.43695
C	2.607491	0.525277	-0.86797
C	-1.81987	-2.82842	0.188709
C	-3.07344	-2.29532	0.450275
C	-3.29022	-0.93203	0.206662
C	-2.24575	-0.14067	-0.26625
C	-1.16875	2.447948	-1.49917
C	0.73675	3.738091	-1.8424
O	-4.46934	-0.27885	0.427805
C	3.958502	0.056454	-0.35658
C	1.478836	-0.34795	-0.89774
C	2.460257	1.884137	-1.18561
O	-0.65514	3.622181	-2.1239
C	0.038759	1.564082	-1.24598
C	0.158133	0.196085	-0.91917
O	4.327584	0.925059	0.722009
C	5.525699	0.709573	1.344212
C	6.421742	-0.31692	1.020796
C	7.620773	-0.43292	1.732402
C	7.935807	0.455819	2.757047
C	7.033221	1.476939	3.075326
C	5.838007	1.606395	2.377778
C	-5.59052	-1.03247	0.890485
C	-6.78905	-0.11948	0.982644
C	-7.64336	-0.18906	2.087305
C	-8.78345	0.614276	2.155953
C	-9.07279	1.504481	1.121809
C	-8.21857	1.585559	0.018969
C	-7.08624	0.77576	-0.05281
C	3.621174	2.850067	-1.26446
H	3.569238	-1.76731	-1.93012
H	3.83428	-4.19482	-2.06534
H	1.972763	-5.69011	-1.31856
H	-0.13428	-4.7116	-0.55591
H	-1.66435	-3.87991	0.402935
H	-3.85457	-2.93281	0.847237
H	-2.4623	0.9108	-0.37015
H	-1.91102	2.0015	-2.17119
H	-1.68204	2.71965	-0.56042
H	1.239498	4.108141	-2.74556
H	0.91618	4.475351	-1.03972
H	4.738937	0.093026	-1.13297
H	3.907526	-0.96619	0.021424
H	6.199573	-1.02562	0.231461
H	8.309814	-1.23326	1.474767
H	8.869065	0.356718	3.303652
H	7.262515	2.177867	3.873838
H	5.12599	2.391161	2.61447
H	-5.37126	-1.47667	1.871308
H	-5.78584	-1.85739	0.188095
H	-7.41523	-0.87321	2.901783
H	-9.43782	0.550206	3.021215
H	-9.95599	2.135365	1.175489
H	-8.43585	2.280884	-0.78755
H	-6.4179	0.844133	-0.90593
H	4.563365	2.353429	-1.50953
H	3.434699	3.608726	-2.03246
H	3.774754	3.370459	-0.31226

Fragment 3

Sum of electronic and zero-point Energies=
-1883.898376
Sum of electronic and thermal Energies=
-1883.862691
Sum of electronic and thermal Enthalpies=
-1883.861747
Sum of electronic and thermal Free Energies=
-1883.968937

C	-3.45574	-2.43338	0.664089
C	-3.85882	-3.7293	0.949235
C	-2.93433	-4.77827	0.889179
C	-1.61154	-4.50375	0.583289
C	-1.17706	-3.19463	0.290693
C	-2.12634	-2.13557	0.284789
C	0.225256	-2.89262	0.02817
C	0.666568	-1.54859	0.096669
C	-0.31323	-0.46487	0.270555
C	-1.67898	-0.75366	0.038007
C	0.066587	0.89819	0.499165
C	-0.73402	1.933493	-0.04074
C	-2.07733	1.632696	-0.4241
C	-2.54368	0.313644	-0.35703
C	1.165363	-3.8969	-0.29628
C	2.490382	-3.60563	-0.53901
C	2.022063	-1.25813	-0.19375
C	1.154106	1.274072	1.416338
C	-0.08823	3.245555	-0.23346
O	4.247327	-2.07143	-0.77345
C	-3.92074	0.031411	-0.92368
C	2.926928	-2.26775	-0.48715

C	1.629907	0.367964	2.393746
C	2.553559	0.751251	3.350659
C	3.021022	2.073044	3.382114
C	2.540762	2.988754	2.461719
C	1.607599	2.617383	1.470732
C	1.08564	3.575094	0.499037
C	1.736814	4.799675	0.248813
C	1.274916	5.685881	-0.7103
C	0.155072	5.343035	-1.47537
C	-0.5018	4.143861	-1.24389
O	-4.93774	0.282647	0.061911
C	-6.25122	0.141707	-0.31063
C	-7.19452	0.460574	0.677349
C	-8.55279	0.34858	0.402906
C	-8.99097	-0.08077	-0.85487
C	-8.04951	-0.39684	-1.83111
C	-6.67881	-0.29046	-1.57112
C	4.778028	-0.75011	-0.66162
C	6.270474	-0.80021	-0.88412
C	7.046669	-1.81865	-0.31686
C	8.43012	-1.83014	-0.4892
C	9.056514	-0.81957	-1.22294
C	8.28956	0.198572	-1.78977
C	6.90314	0.203258	-1.6247
C	-3.08065	2.727471	-0.74952
H	-4.16557	-1.62844	0.802961
H	-4.88652	-3.91793	1.247179
H	-3.23873	-5.79558	1.120108
H	-0.88902	-5.31239	0.610968
H	0.838559	-4.92712	-0.38645
H	3.205043	-4.38076	-0.7966
H	2.342631	-0.22726	-0.21049
H	-3.98919	-1.00343	-1.27115
H	-4.10356	0.681106	-1.78713
H	1.242141	-0.64281	2.411687
H	2.890443	0.032489	4.092555
H	3.732331	2.386893	4.141122
H	2.865839	4.021323	2.529995
H	2.645382	5.040335	0.790183
H	1.801188	6.619536	-0.88861
H	-0.18946	5.999193	-2.27003
H	-1.31813	3.875775	-1.89897
H	-6.83742	0.793477	1.647157
H	-9.27492	0.598826	1.175738
H	-10.0527	-0.16685	-1.06692
H	-8.37421	-0.73359	-2.81228
H	-5.96635	-0.54778	-2.34617
H	4.303846	-0.08708	-1.39842
H	4.545948	-0.35291	0.338233
H	6.558261	-2.60789	0.246481
H	9.021325	-2.62906	-0.04918
H	10.13516	-0.82921	-1.35521
H	8.766925	0.984587	-2.36894
H	6.308041	0.992995	-2.07819
H	-2.78959	3.677278	-0.29795
H	-3.20413	2.90078	-1.82759
H	-4.06084	2.463483	-0.34819

(*R*p,*R*a)-**IM0**

Sum of electronic and zero-point Energies=
-1994.594063
Sum of electronic and thermal Energies=
-1994.554400
Sum of electronic and thermal Enthalpies=
-1994.553456
Sum of electronic and thermal Free Energies=
-1994.668125

C	-1.22522	-3.78448	-0.80503
C	-0.14598	-4.42395	-1.38404
C	0.757363	-3.68889	-2.15436
C	0.53668	-2.3422	-2.37033
C	-0.55191	-1.6734	-1.78761
C	-1.41828	-2.39767	-0.94
C	-0.83534	-0.27042	-2.05392
C	-2.09409	0.262728	-1.68669
C	-3.02506	-0.52789	-0.88613
C	-2.54904	-1.72866	-0.30341
C	-4.31539	-0.11355	-0.51723
C	-5.01549	-0.77448	0.477509
C	-4.42023	-1.75535	1.268005
C	-3.16516	-2.22827	0.872495
C	0.109055	0.575558	-2.65653
C	-0.14308	1.915003	-2.86973
C	-1.37706	2.437507	-2.48738
C	-2.34302	1.618851	-1.93859
C	-5.20895	0.994003	-1.01322
O	-6.49776	0.671077	-0.53594
C	-6.37115	-0.14901	0.607393
O	-1.58136	3.790647	-2.59478
C	-2.33859	-2.92824	1.923157
C	-5.09587	-2.23129	2.522114
C	2.453476	4.411802	0.934781
C	3.693278	4.169344	1.5011
C	3.945473	2.934664	2.091549
C	2.95111	1.968152	2.123367
C	1.685372	2.193874	1.571805
C	1.440701	3.438818	0.9559
C	0.664342	1.13106	1.729903
C	0.928656	-0.19886	1.336303
C	2.055468	-0.5399	0.528723
C	0.230953	3.74943	0.263982
C	2.973566	-0.88399	-0.18087
C	-0.71202	4.132812	-0.39435
C	-0.52768	1.383026	2.41264
C	-1.40431	0.359867	2.751098
C	-1.09419	-0.96092	2.43129
C	0.056244	-1.23203	1.699979
C	4.053347	-1.29064	-1.06246
O	-1.93356	-1.94177	2.884167
C	-1.78129	4.437579	-1.33816
O	5.287043	-1.23999	-0.38868
C	6.336565	-1.62559	-1.24504
C	7.608076	-1.56586	-0.54378
C	8.677035	-1.52395	0.015594
H	-1.97058	-4.37245	-0.27202
H	-0.02173	-5.49866	-1.26551
H	1.605508	-4.1828	-2.6259
H	1.201834	-1.80228	-3.04169
H	1.09515	0.188195	-2.9034
H	0.612004	2.584966	-3.27445
H	-3.26994	2.081887	-1.62208
H	-5.251	1.082355	-2.10828
H	-4.89783	1.979503	-0.61063
H	-7.19625	-0.87649	0.605391
H	-6.46132	0.447853	1.536733
H	-2.91292	-3.65838	2.503391
H	-1.45826	-3.44074	1.526215
H	-5.18744	-3.32547	2.554633
H	-6.107	-1.81926	2.614932
H	-4.52979	-1.92841	3.413194
H	2.242922	5.361983	0.447115
H	4.463746	4.937534	1.474293
H	4.917002	2.723575	2.534246
H	3.147155	1.009168	2.600217
H	-0.75469	2.406173	2.710186
H	-2.3269	0.559751	3.293633
H	0.306738	-2.24665	1.394928
H	3.861991	-2.31585	-1.43954
H	4.079062	-0.63368	-1.95592
H	-2.76255	4.165502	-0.91336
H	-1.81791	5.509472	-1.56816
H	6.361815	-0.96692	-2.13603
H	6.15938	-2.65102	-1.62648
C	9.958643	-1.47163	0.697685
H	10.75733	-1.88995	0.0718
H	9.937442	-2.04609	1.632425
H	10.24001	-0.44056	0.946718

TS$_{rot}$

Sum of electronic and zero-point Energies=
-4485.328585
Sum of electronic and thermal Energies=
-4485.249080
Sum of electronic and thermal Enthalpies=
-4485.248136
Sum of electronic and thermal Free Energies=
-4485.440502

C	1.118343	-3.00405	2.073954
C	0.158796	-3.27586	3.030429
C	-0.5458	-2.2251	3.619284
C	-0.25267	-0.92249	3.263386
C	0.706289	-0.62735	2.281825
C	1.382123	-1.69091	1.644052
C	1.053307	0.742295	1.930205
C	2.256607	0.988401	1.229088
C	3.054187	-0.12845	0.729645
C	2.4477	-1.4097	0.683987
C	4.31344	-0.00071	0.125108
C	4.861288	-1.04535	-0.60112
C	4.124278	-2.18579	-0.91462
C	2.895415	-2.36014	-0.26816
C	0.215095	1.822502	2.246739
C	0.51159	3.109756	1.848853
C	1.675378	3.341057	1.116301
C	2.547736	2.306078	0.841278
C	5.317033	1.122844	0.099589
O	6.527718	0.521134	-0.30909
C	6.231293	-0.64136	-1.05639
O	1.893831	4.594967	0.597823
C	1.920769	-3.32705	-0.89525
C	4.622981	-3.15212	-1.95034
C	-2.95932	3.861206	-0.97124

C	-4.28857	3.546756	-0.75951
C	-4.63857	2.205975	-0.70492
C	-3.67731	1.226223	-0.90029
C	-2.32354	1.492838	-1.15798
C	-1.96954	2.87819	-1.15606
C	-1.3816	0.35788	-1.41005
C	-1.77914	-1.01096	-1.52533
C	-3.10458	-1.54374	-1.47305
C	-0.66042	3.448928	-1.2215
C	-4.1644	-2.1312	-1.44016
C	0.36354	4.099271	-1.16991
C	-0.00796	0.597291	-1.58029
C	0.927424	-0.39366	-1.83591
C	0.524596	-1.7219	-1.89734
C	-0.81864	-2.01218	-1.74107
C	-5.50009	-2.72164	-1.41305
O	1.420203	-2.74026	-2.11066
C	1.655139	4.674743	-0.81059
O	-6.51726	-1.82368	-1.02182
C	-6.72497	-1.75267	0.373848
C	-5.63278	-1.12512	1.113077
C	-4.69935	-0.60425	1.677269
H	1.727663	-3.81981	1.689406
H	-0.01773	-4.30229	3.346172
H	-1.29149	-2.42391	4.387336
H	-0.74986	-0.10835	3.787956
H	-0.72803	1.639608	2.758433
H	-0.17115	3.937368	2.029045
H	3.418436	2.531733	0.233129
H	5.472815	1.608515	1.073365
H	5.032063	1.912968	-0.62347
H	7.006143	-1.39366	-0.84866
H	6.258542	-0.43216	-2.14422
H	2.394126	-4.26519	-1.20526
H	1.067488	-3.56898	-0.25279
H	4.684259	-4.17885	-1.56435
H	5.622821	-2.87918	-2.30619
H	3.954152	-3.17263	-2.82097
H	-2.63702	4.900844	-0.98402
H	-5.02701	4.333764	-0.6208
H	-5.66416	1.897459	-0.5065
H	-4.0189	0.203071	-0.83041
H	0.363282	1.612079	-1.50718
H	1.977685	-0.13969	-1.96999
H	-1.1464	-3.04818	-1.8103
H	-5.50791	-3.61582	-0.76214
H	-5.77079	-3.06265	-2.4225
H	2.464087	4.164228	-1.36137
H	1.716934	5.741755	-1.05553
H	-6.91648	-2.76603	0.775232
H	-7.65374	-1.18005	0.498226
C	-3.54924	0.012259	2.313116
H	-3.4714	-0.27728	3.369877
H	-3.60243	1.108199	2.260538
H	-2.61717	-0.29861	1.816543

(*R*p,*S*a)-IM0

Sum of electronic and zero-point Energies=
-1994.595696
Sum of electronic and thermal Energies=
-1994.556085
Sum of electronic and thermal Enthalpies=
-1994.555141
Sum of electronic and thermal Free Energies=
-1994.670345

C	-4.53007	-3.04845	-1.49249
C	-5.58076	-3.11621	-2.38625
C	-6.29768	-1.95854	-2.69522
C	-5.96483	-0.7622	-2.09021
C	-4.89315	-0.66859	-1.18783
C	-4.13881	-1.83147	-0.90286
C	-4.53237	0.580307	-0.53494
C	-3.62573	0.557341	0.546167
C	-3.01309	-0.69253	0.975851
C	-3.07171	-1.79198	0.090273
C	-2.23549	-0.84496	2.13674
C	-1.5133	-2.00294	2.350699
C	-1.34063	-2.96135	1.350291
C	-2.08832	-2.81181	0.182014
C	-5.02051	1.826248	-0.96775
C	-4.59733	3.011377	-0.40798
C	-3.64053	2.982662	0.612958
C	-3.18646	1.7698	1.094372
C	-2.0027	0.042001	3.332442
O	-1.45023	-0.80435	4.319039
C	-0.85278	-1.9211	3.690474
O	-3.1874	4.185137	1.05583
C	-1.55279	-3.44424	-1.08087
C	-0.31559	-4.03811	1.547349
C	1.935116	4.600761	-0.74795
C	3.092388	4.684152	-1.50089
C	3.627204	3.525631	-2.05646
C	2.997476	2.308433	-1.84904
C	1.827931	2.194384	-1.08608
C	1.288907	3.370943	-0.52593
C	1.221505	0.845635	-0.97368
C	2.002727	-0.26482	-0.57461
C	3.319511	-0.10653	-0.04448
C	0.116952	3.439522	0.292799
C	4.432243	-0.00149	0.418891
C	-0.82279	3.751175	0.993982
C	-0.08222	0.597415	-1.40544
C	-0.59621	-0.69226	-1.5102
C	0.194451	-1.78436	-1.14407
C	1.477096	-1.55611	-0.65542
C	5.752393	0.175359	0.998257
O	-0.17397	-3.09438	-1.23431
C	-1.96998	4.184561	1.786841
O	6.70536	-0.57742	0.287892
C	7.986392	-0.41154	0.849192
C	8.965906	-1.18924	0.108965
C	9.798202	-1.82692	-0.48933
H	-4.03086	-3.96809	-1.19102
H	-5.86554	-4.07283	-2.81995
H	-7.13854	-2.00152	-3.38508
H	-6.56917	0.117976	-2.29982
H	-5.71418	1.871176	-1.80472
H	-4.94622	3.974121	-0.77454
H	-2.39882	1.749916	1.832973
H	-2.91585	0.505216	3.734502
H	-1.28764	0.858161	3.098434
H	-1.01919	-2.80665	4.322583
H	0.243153	-1.78785	3.595543
H	-1.54715	-4.54206	-1.0772
H	-2.11741	-3.12269	-1.96543
H	-0.42224	-4.86611	0.839873
H	-0.38868	-4.4531	2.561972
H	0.70446	-3.64304	1.433594
H	1.499715	5.493952	-0.30366
H	3.572359	5.648289	-1.65742
H	4.531163	3.569726	-2.66121
H	3.410376	1.407903	-2.29991
H	-0.70584	1.43757	-1.712
H	-1.60403	-0.83191	-1.89558
H	2.081329	-2.40779	-0.34804
H	5.742736	-0.12744	2.064618
H	6.023273	1.249975	0.986556
H	-2.07283	3.588847	2.70978
H	-1.83469	5.227121	2.09889
H	7.978763	-0.72141	1.913186
H	8.26818	0.660251	0.84422
C	10.79194	-2.59676	-1.21763
H	11.80923	-2.27454	-0.9611
H	10.67188	-2.47633	-2.30172
H	10.71694	-3.66801	-0.9918

IM1

Sum of electronic and zero-point Energies=
-4485.344255
Sum of electronic and thermal Energies=
-4485.263731
Sum of electronic and thermal Enthalpies=
-4485.262787
Sum of electronic and thermal Free Energies=
-4485.457055

C	-8.05755	-2.5929	-0.80342
C	-9.34916	-2.40807	-1.2575
C	-9.97734	-1.17592	-1.06741
C	-9.31243	-0.16336	-0.40286
C	-7.99646	-0.32626	0.058087
C	-7.33573	-1.55717	-0.17928
C	-7.28949	0.73202	0.764837
C	-6.07449	0.430791	1.413281
C	-5.55001	-0.92788	1.393661
C	-6.01078	-1.80289	0.385838
C	-4.54478	-1.40457	2.256556
C	-3.94454	-2.62567	2.019334
C	-4.18768	-3.36271	0.855548
C	-5.20645	-2.9163	0.01329
C	-7.73727	2.065061	0.782804
C	-6.98598	3.076684	1.340669
C	-5.72576	2.783358	1.873634
C	-5.28822	1.473441	1.92528
C	-4.07151	-0.91685	3.601134
O	-3.02211	-1.80156	3.973656
C	-2.99142	-2.93439	3.12702
O	-4.97742	3.843786	2.289526
C	-5.19426	-3.38798	-1.4204
C	-3.31755	-4.54788	0.552182
C	-2.00933	4.653464	-2.13414
C	-1.48372	4.842827	-3.40152
C	-1.26595	3.738956	-4.22123

C	-1.62321	2.473412	-3.77397
C	-2.16641	2.258948	-2.49976
C	-2.32616	3.372388	-1.64838
C	-2.66156	0.890301	-2.20994
C	-2.19482	0.049838	-1.18231
C	-1.26575	0.417013	-0.14821
C	-2.79063	3.305404	-0.29882
C	-0.81791	0.277591	1.009071
C	0.375839	2.616905	1.731459
C	0.5786	3.204176	0.664924
C	-3.09789	3.4623	0.864487
C	-3.61411	0.36948	-3.09604
C	-4.07415	-0.93752	-3.0107
C	-3.56509	-1.78077	-2.02542
C	-2.65356	-1.27634	-1.10994
C	-0.9322	0.162274	2.476745
O	-1.21468	1.403988	3.07955
C	-0.10045	2.268747	3.082168
O	-3.90642	-3.10605	-1.98821
C	-3.56929	3.690469	2.228726
C	0.704875	4.17308	-0.41683
C	7.252387	-3.31214	-2.70652
C	6.823303	-3.95539	-1.39911
C	5.573586	-3.27363	-0.86198
C	5.658412	-1.76183	-0.86625
C	6.650321	-1.11455	-1.61523
C	7.65715	-1.87086	-2.44392
C	4.703152	-0.99576	-0.16515
C	4.728825	0.411329	-0.22064
C	3.683189	-1.72435	0.643731
C	4.072803	-2.29704	1.876356
C	3.134183	-3.00302	2.641138
C	1.818262	-3.10385	2.183115
C	1.425757	-2.52374	0.99186
C	2.361548	-1.84926	0.190439
C	5.477119	-2.10217	2.40214
C	5.866369	-3.12139	3.462829
C	4.803331	-3.18217	4.54641
C	3.492025	-3.66696	3.947779
P	3.381421	1.422063	0.559647
P	1.63786	-0.82202	-1.14219
C	3.840795	3.171523	0.236913
C	3.755659	1.178691	2.340107
C	2.762659	-0.40529	-2.52944
C	0.496118	-1.89317	-2.09653
C	2.703455	0.89143	-3.04427
C	3.406465	1.22503	-4.19915
C	4.171589	0.261595	-4.84728
C	4.213616	-1.04066	-4.35299
C	3.499688	-1.37757	-3.20989
C	0.370162	-3.27331	-1.92538
C	-0.43254	-4.01345	-2.79085
C	-1.09245	-3.38938	-3.84265
C	-0.9512	-2.01686	-4.0328
C	-0.16724	-1.27383	-3.16257
C	3.86615	4.113017	1.271188
C	4.094356	5.459354	0.999041
C	4.301401	5.886102	-0.30822
C	4.258413	4.961234	-1.34861
C	4.014036	3.619452	-1.08092
C	2.860139	0.480949	3.147052
C	3.140319	0.252815	4.491415
C	4.331446	0.72045	5.037209
C	5.250661	1.390993	4.230659

C	4.971957	1.609044	2.886318
C	5.773743	1.031711	-0.91337
C	6.710971	0.27777	-1.59551
Rh	1.013078	1.027137	0.129663
H	-7.61848	-3.58669	-0.87024
H	-9.8833	-3.23184	-1.72686
H	-11.0003	-1.0226	-1.40568
H	-9.83817	0.769158	-0.20895
H	-8.67452	2.328023	0.297294
H	-7.3162	4.112713	1.316271
H	-4.28092	1.253051	2.263544
H	-4.89201	-0.95476	4.339681
H	-3.66125	0.101612	3.63809
H	-3.27189	-3.84492	3.687109
H	-1.95386	-3.08362	2.769379
H	-5.32418	-4.47035	-1.54665
H	-5.96448	-2.88248	-2.01684
H	-3.75624	-5.2193	-0.19269
H	-3.13677	-5.13564	1.462495
H	-2.33391	-4.24273	0.159387
H	-2.1879	5.505378	-1.47906
H	-1.24742	5.846561	-3.74916
H	-0.8471	3.864202	-5.21798
H	-1.49923	1.613549	-4.43376
H	-4.00008	1.020845	-3.8793
H	-4.80105	-1.32199	-3.72483
H	-2.25965	-1.92774	-0.33206
H	-1.76714	-0.50889	2.721001
H	-0.02865	-0.28794	2.924924
H	-0.42941	3.186849	3.587204
H	0.71729	1.831789	3.682147
H	-3.2141	2.893755	2.903547
H	-3.16804	4.637204	2.612662
H	-0.05594	4.954888	-0.29322
H	0.546975	3.707098	-1.39895
H	1.692943	4.647462	-0.41015
H	8.080967	-3.8604	-3.17245
H	6.410377	-3.34302	-3.41963
H	6.633168	-5.02937	-1.52263
H	7.64052	-3.86254	-0.66426
H	5.348807	-3.64732	0.145968
H	4.704124	-3.58175	-1.47141
H	8.62863	-1.85868	-1.92098
H	7.822368	-1.32783	-3.38658
H	1.087939	-3.63988	2.791278
H	0.376807	-2.57114	0.694273
H	5.544356	-1.08603	2.830874
H	6.206047	-2.11199	1.581814
H	5.97516	-4.11675	2.998655
H	6.84695	-2.86182	3.882451
H	5.106116	-3.84247	5.369246
H	4.671352	-2.17557	4.976348
H	2.661946	-3.53691	4.658646
H	3.558284	-4.75443	3.77132
H	2.085647	1.638932	-2.53809
H	3.348136	2.238082	-4.59527
H	4.722598	0.518644	-5.74999
H	4.793396	-1.80238	-4.87259
H	3.50238	-2.40855	-2.85217
H	0.910191	-3.78408	-1.12978
H	-0.53098	-5.08763	-2.64485
H	-1.72112	-3.97137	-4.51347
H	-1.46316	-1.5212	-4.85621
H	-0.05697	-0.1985	-3.31663

H	3.702899	3.799517	2.300913
H	4.114541	6.17608	1.817883
H	4.489976	6.93718	-0.51776
H	4.409309	5.286619	-2.37632
H	3.964746	2.91269	-1.9097
H	1.944045	0.098214	2.698571
H	2.428392	-0.29726	5.107079
H	4.552543	0.553134	6.090053
H	6.191877	1.740496	4.650745
H	5.701816	2.121711	2.258656
H	5.859818	2.114798	-0.9321
H	7.505954	0.780688	-2.14784

TS1

Sum of electronic and zero-point Energies=
-4485.328585
Sum of electronic and thermal Energies=
-4485.249080
Sum of electronic and thermal Enthalpies=
-4485.248136
Sum of electronic and thermal Free Energies=
-4485.440502

C	7.169914	-3.71511	0.314279
C	8.470363	-3.95345	0.712819
C	9.405481	-2.91723	0.665371
C	9.028692	-1.67344	0.197738
C	7.707771	-1.40679	-0.19959
C	6.747786	-2.43879	-0.10675
C	7.298465	-0.10885	-0.71294
C	6.037909	0.036903	-1.33643
C	5.127246	-1.10406	-1.42491
C	5.392321	-2.22467	-0.59894
C	3.939908	-1.14426	-2.18339
C	3.066415	-2.21336	-2.08142
C	3.213842	-3.20249	-1.10925
C	4.351408	-3.14312	-0.30564
C	8.117323	1.026039	-0.57807
C	7.703908	2.276523	-0.97543
C	6.427274	2.429731	-1.52252
C	5.625755	1.321435	-1.73018
C	3.364688	-0.19724	-3.1986
O	2.248798	-0.86051	-3.7685
C	1.905545	-1.99352	-2.99612
O	6.04677	3.708322	-1.7994
C	4.244952	-3.74557	1.073395
C	2.140681	-4.23172	-0.90396
C	2.519259	4.801058	2.358211
C	1.97781	4.945651	3.625975
C	1.758401	3.815997	4.409129
C	2.06231	2.559106	3.899001
C	2.591512	2.385335	2.613765
C	2.84375	3.535753	1.837902
C	2.859381	0.984747	2.191688
C	2.069143	0.32607	1.224841
C	1.088968	1.051378	0.445824
C	3.475406	3.522276	0.555367
C	0.88579	1.862769	-0.53061
C	-0.57868	3.253873	-0.25134
C	-1.0828	3.121098	0.918997

C	4.018048	3.725892	-0.51028
C	3.788468	0.229146	2.912467
C	3.924955	-1.14183	2.729657
C	3.093385	-1.79859	1.824869
C	2.190788	-1.05978	1.066903
C	1.32657	2.487322	-1.81013
O	1.030192	3.852533	-1.86805
C	-0.29876	4.062018	-1.45902
O	3.098581	-3.16508	1.7184
C	4.662728	3.994513	-1.79517
C	-1.28836	3.543774	2.301217
C	-7.75388	-2.62438	2.289459
C	-7.03322	-3.53561	1.30945
C	-5.66889	-2.95503	0.968307
C	-5.72618	-1.5013	0.560263
C	-6.84482	-0.71919	0.886385
C	-8.03042	-1.28319	1.628511
C	-4.64147	-0.91486	-0.11927
C	-4.66628	0.454288	-0.45628
C	-3.50438	-1.77609	-0.56696
C	-3.68682	-2.53885	-1.74675
C	-2.61564	-3.2736	-2.27227
C	-1.3919	-3.26361	-1.60203
C	-1.21936	-2.55177	-0.43165
C	-2.27771	-1.81387	0.118135
C	-5.02016	-2.54147	-2.46086
C	-5.17246	-3.68915	-3.44894
C	-3.96195	-3.75888	-4.364
C	-2.72694	-4.08279	-3.53984
P	-3.13977	1.301651	-1.03986
P	-1.83443	-0.6746	1.490773
C	-3.63901	3.03213	-1.38498
C	-2.76198	0.58802	-2.68325
C	-3.2729	-0.44591	2.609884
C	-0.7012	-1.5808	2.599762
C	-3.93769	0.779526	2.664913
C	-5.00773	0.968119	3.532346
C	-5.41007	-0.06664	4.371258
C	-4.73561	-1.28505	4.343865
C	-3.67004	-1.47421	3.471273
C	-0.59892	-2.97346	2.662235
C	0.239209	-3.57061	3.598491
C	0.966671	-2.78571	4.486786
C	0.852261	-1.39909	4.444178
C	0.029701	-0.79833	3.499731
C	-3.62397	3.583296	-2.66945
C	-3.88505	4.938478	-2.86151
C	-4.16132	5.760403	-1.77576
C	-4.18083	5.223431	-0.48991
C	-3.91367	3.874697	-0.29769
C	-1.45811	0.187483	-2.98909
C	-1.14128	-0.3145	-4.24856
C	-2.13692	-0.42047	-5.21455
C	-3.44282	-0.02799	-4.92126
C	-3.75629	0.47078	-3.66206
C	-5.81054	1.202978	-0.16526
C	-6.88077	0.616047	0.487152
Rh	-1.02309	1.197759	0.198672
H	6.474354	-4.55119	0.263014
H	8.770033	-4.95193	1.024921
H	10.43838	-3.09601	0.957862
H	9.787149	-0.89918	0.108241
H	9.087947	0.935749	-0.09656
H	8.321695	3.159307	-0.82816
H	4.630717	1.460624	-2.13096
H	4.069093	0.078433	-3.99868
H	3.017528	0.73921	-2.73063
H	1.699837	-2.83826	-3.67533
H	0.969943	-1.80612	-2.42476
H	4.053532	-4.82596	1.093649
H	5.140399	-3.54707	1.675864
H	2.556294	-5.19684	-0.58937
H	1.588046	-4.40908	-1.83563
H	1.421812	-3.93834	-0.12355
H	2.729254	5.675702	1.745065
H	1.748169	5.938027	4.009214
H	1.354965	3.911047	5.415722
H	1.891441	1.672113	4.510212
H	4.40479	0.736631	3.654324
H	4.640327	-1.71423	3.31845
H	1.563773	-1.57162	0.338712
H	2.416114	2.38964	-1.86595
H	0.870306	1.942106	-2.66217
H	-0.42594	5.132874	-1.26052
H	-1.00955	3.771415	-2.25805
H	4.146603	3.46502	-2.61677
H	4.59238	5.063543	-2.02966
H	-0.41397	3.23546	2.900023
H	-2.18468	3.105718	2.75725
H	-1.35269	4.640473	2.356241
H	-8.69291	-3.07239	2.638839
H	-7.11874	-2.47755	3.179005
H	-6.91452	-4.54738	1.718377
H	-7.63554	-3.63526	0.390188
H	-5.18045	-3.55544	0.188759
H	-5.01019	-3.0382	1.851568
H	-8.86419	-1.39644	0.914584
H	-8.37451	-0.544	2.367729
H	-0.55828	-3.83409	-2.01573
H	-0.24815	-2.56121	0.062308
H	-5.13233	-1.58442	-2.99841
H	-5.8457	-2.55944	-1.73698
H	-5.27224	-4.64115	-2.90077
H	-6.09959	-3.55916	-4.02224
H	-4.09671	-4.51015	-5.15272
H	-3.82995	-2.78604	-4.86553
H	-1.80913	-3.94125	-4.13157
H	-2.74275	-5.15234	-3.26846
H	-3.62179	1.582339	1.998508
H	-5.52913	1.924004	3.550427
H	-6.24394	0.078509	5.056086
H	-5.03578	-2.09098	5.011982
H	-3.13623	-2.42499	3.469621
H	-1.17633	-3.59828	1.980803
H	0.324397	-4.65496	3.63183
H	1.625546	-3.25581	5.21434
H	1.423241	-0.78479	5.139155
H	-0.04098	0.291727	3.449073
H	-3.39592	2.958643	-3.53161
H	-3.8702	5.349835	-3.869
H	-4.36354	6.818768	-1.92823
H	-4.40171	5.859175	0.365743
H	-3.92591	3.468081	0.714975
H	-0.67157	0.262093	-2.23335
H	-0.11134	-0.59768	-4.46926
H	-1.89664	-0.80562	-6.20415
H	-4.2205	-0.11012	-5.67937
H	-4.77894	0.779781	-3.43586
H	-5.8662	2.254184	-0.44097
H	-7.76789	1.210568	0.710835

IM2

Sum of electronic and zero-point Energies=
-4485.361651
Sum of electronic and thermal Energies=
-4485.281966
Sum of electronic and thermal Enthalpies=
-4485.281021
Sum of electronic and thermal Free Energies=
-4485.475079

C	-6.98171	-4.16319	-0.31594
C	-8.218	-4.5812	-0.76491
C	-9.26694	-3.66139	-0.84182
C	-9.06806	-2.35546	-0.44033
C	-7.81432	-1.90684	0.011935
C	-6.73884	-2.82023	0.035966
C	-7.59575	-0.55112	0.489536
C	-6.38191	-0.20945	1.134519
C	-5.30088	-1.18845	1.248648
C	-5.43564	-2.40665	0.533904
C	-4.06709	-0.97383	1.905173
C	-3.08492	-1.95002	1.898613
C	-3.15983	-3.07995	1.085801
C	-4.29676	-3.21813	0.295716
C	-8.58085	0.441995	0.338287
C	-8.39433	1.734208	0.769339
C	-7.18314	2.076593	1.375521
C	-6.2093	1.11439	1.56938
C	-3.4977	0.212459	2.636945
O	-2.22206	-0.18333	3.115592
C	-1.88474	-1.47337	2.64604
O	-7.03602	3.382544	1.726143
C	-4.08039	-3.81998	-1.07015
C	-1.99513	-4.01739	0.972894
C	-3.09541	4.947324	-1.90476
C	-2.2728	5.134419	-3.00246
C	-1.7167	4.025833	-3.63749
C	-1.98279	2.752373	-3.15407
C	-2.80126	2.539448	-2.03737
C	-3.37719	3.660177	-1.41265
C	-2.99379	1.115811	-1.64106
C	-1.91023	0.369717	-1.09735
C	-0.7483	1.059741	-0.60663
C	-4.20394	3.586291	-0.25039
C	-0.59288	2.313877	-0.04255
C	0.412529	3.226068	-0.58985
C	1.297142	2.708089	-1.48887
C	-4.87324	3.715672	0.752776
C	-4.13404	0.428595	-2.05346
C	-4.19152	-0.96374	-2.04817
C	-3.08432	-1.70441	-1.62562
C	-1.97987	-1.03178	-1.11517
C	-1.24563	3.073329	1.086141
O	-1.01188	4.431439	0.780252
C	0.237898	4.539416	0.133277
O	-3.02162	-3.06878	-1.70438
C	-5.71858	3.851731	1.936007

C	1.818598	3.160496	-2.78743
C	8.210408	-2.37048	-1.83793
C	7.434213	-3.34392	-0.96615
C	6.023808	-2.82105	-0.73825
C	5.994485	-1.38406	-0.27351
C	7.114315	-0.55611	-0.44851
C	8.379755	-1.04869	-1.10478
C	4.831579	-0.85902	0.316853
C	4.779675	0.490017	0.72287
C	3.685677	-1.76292	0.639913
C	3.78619	-2.51071	1.838378
C	2.694571	-3.27139	2.279061
C	1.537624	-3.308	1.502239
C	1.449196	-2.61533	0.311694
C	2.524035	-1.84259	-0.15231
C	5.052636	-2.46327	2.664424
C	5.13961	-3.56698	3.709491
C	3.844774	-3.64716	4.499072
C	2.717027	-4.05425	3.566529
P	3.155242	1.228559	1.157716
P	2.163316	-0.72787	-1.57185
C	3.450008	3.017962	1.427806
C	2.686963	0.607285	2.808014
C	3.65989	-0.51861	-2.60872
C	1.024323	-1.61876	-2.68787
C	4.407249	0.658709	-2.54412
C	5.540815	0.821874	-3.33149
C	5.930176	-0.19052	-4.20411
C	5.182017	-1.36233	-4.28841
C	4.049564	-1.52608	-3.49792
C	0.930477	-3.01214	-2.77449
C	-0.00353	-3.59658	-3.62328
C	-0.83444	-2.80039	-4.40626
C	-0.71864	-1.41475	-4.35625
C	0.202437	-0.82878	-3.49675
C	3.101511	3.644462	2.627941
C	3.19669	5.029063	2.755963
C	3.63635	5.804003	1.689454
C	3.97948	5.190675	0.484897
C	3.877625	3.812225	0.353282
C	1.346637	0.302975	3.061103
C	0.942725	-0.10063	4.331102
C	1.88184	-0.20683	5.352303
C	3.219851	0.105287	5.111066
C	3.621638	0.516339	3.845367
C	5.922198	1.283458	0.589229
C	7.069382	0.758069	0.01698
Rh	1.223892	1.038242	-0.45738
H	-6.19276	-4.90039	-0.17412
H	-8.38067	-5.62564	-1.02319
H	-10.2515	-3.97926	-1.17942
H	-9.91715	-1.67664	-0.4505
H	-9.51421	0.202105	-0.16433
H	-9.14874	2.503848	0.623949
H	-5.27646	1.409889	2.021168
H	-4.10839	0.546372	3.492008
H	-3.37779	1.074729	1.950357
H	-1.61205	-2.11861	3.501107
H	-0.99123	-1.41597	1.990922
H	-3.71322	-4.85366	-1.07526
H	-4.98439	-3.78053	-1.68952
H	-2.31749	-5.035	0.722402
H	-1.45531	-4.07521	1.926617
H	-1.28493	-3.71071	0.18972
H	-3.53759	5.799552	-1.39256
H	-2.07617	6.139693	-3.36991
H	-1.08842	4.154069	-4.51834
H	-1.55967	1.879435	-3.65435
H	-4.97435	0.997128	-2.45179
H	-5.08462	-1.47203	-2.41011
H	-1.14283	-1.60968	-0.72876
H	-2.32749	2.915468	1.165804
H	-0.76895	2.786377	2.045694
H	0.210806	5.400306	-0.54856
H	1.055252	4.690712	0.863076
H	-5.25627	3.353927	2.806904
H	-5.83171	4.909505	2.201463
H	1.089687	2.947176	-3.58654
H	2.77326	2.701595	-3.06806
H	1.941167	4.256332	-2.76166
H	9.192096	-2.77445	-2.1166
H	7.653053	-2.20714	-2.77521
H	7.392723	-4.3418	-1.42164
H	7.948844	-3.45983	0.003088
H	5.48539	-3.46293	-0.02699
H	5.453271	-2.89172	-1.68176
H	9.155249	-1.16056	-0.32788
H	8.754548	-0.26917	-1.78573
H	0.68884	-3.89964	1.847399
H	0.529304	-2.67072	-0.26683
H	5.104018	-1.48373	3.169881
H	5.937232	-2.49449	2.01391
H	5.326273	-4.53523	3.215755
H	5.998788	-3.3805	4.366879
H	3.923637	-4.35855	5.33107
H	3.623741	-2.66022	4.936914
H	1.738764	-3.95199	4.060538
H	2.816438	-5.12602	3.322499
H	4.108367	1.438553	-1.8442
H	6.124459	1.738292	-3.25761
H	6.815359	-0.0646	-4.82547
H	5.478386	-2.15136	-4.97749
H	3.462303	-2.4408	-3.58073
H	1.576506	-3.64559	-2.16645
H	-0.08514	-4.68073	-3.66981
H	-1.5754	-3.26252	-5.05533
H	-1.36747	-0.78827	-4.96696
H	0.268769	0.2584	-3.42121
H	2.750309	3.053613	3.472724
H	2.924361	5.500361	3.698404
H	3.711347	6.884762	1.792534
H	4.326966	5.790574	-0.35477
H	4.139177	3.347107	-0.59838
H	0.600943	0.393881	2.265252
H	-0.11207	-0.30186	4.515547
H	1.570548	-0.51961	6.347549
H	3.949861	0.03687	5.916324
H	4.663758	0.784836	3.660425
H	5.912097	2.320857	0.92021
H	7.95596	1.385429	-0.08834

Rh^{I+}/(*S*)-H_8-binap

Sum of electronic and zero-point Energies=
-2490.727090
Sum of electronic and thermal Energies=
-2490.686484
Sum of electronic and thermal Enthalpies=
-2490.685540
Sum of electronic and thermal Free Energies=
-2490.800504

C	-2.04885	4.014727	3.390769
C	-1.4508	4.566891	2.107584
C	-1.20024	3.433868	1.123719
C	-0.44491	2.271994	1.728404
C	-0.39066	2.117659	3.123978
C	-1.0391	3.100581	4.065561
C	0.172992	1.314884	0.899192
C	0.826312	0.202396	1.471488
C	0.166497	1.504519	-0.58654
C	1.032208	2.464816	-1.1549
C	1.081866	2.612744	-2.55064
C	0.289746	1.793588	-3.35596
C	-0.55383	0.846066	-2.80785
C	-0.64814	0.711476	-1.41823
C	1.946	3.295108	-0.28127
C	2.452714	4.553861	-0.96929
C	3.047222	4.199541	-2.32152
C	1.960571	3.637246	-3.22275
P	1.355356	-1.20359	0.433526
P	-1.55304	-0.72085	-0.74968
C	2.219248	-2.42684	1.476053
C	2.586711	-0.57233	-0.74449
C	-2.55911	-0.18117	0.676346
C	-2.79662	-1.26489	-1.96829
C	-2.47375	-0.85271	1.896527
C	-3.28054	-0.47006	2.963188
C	-4.18594	0.575283	2.809913
C	-4.29231	1.234254	1.586501
C	-3.48405	0.856687	0.520977
C	-3.53466	-0.41517	-2.79877
C	-4.50417	-0.94016	-3.64567
C	-4.74936	-2.31073	-3.66923
C	-4.02961	-3.16345	-2.83831
C	-3.06093	-2.63937	-1.9904
C	3.588792	-2.68846	1.356185
C	4.173907	-3.69858	2.112033
C	3.402679	-4.46036	2.98457
C	2.036345	-4.21801	3.101336
C	1.44494	-3.21325	2.346221
C	2.516068	-0.92716	-2.09262
C	3.479824	-0.46515	-2.98377
C	4.521376	0.337112	-2.52774
C	4.604375	0.681856	-1.1793
C	3.636423	0.233894	-0.28811
C	0.89218	0.079384	2.862098
C	0.298191	1.033731	3.668488
Rh	-0.24662	-2.47499	-0.41258
H	-2.34458	4.819293	4.075837
H	-2.96292	3.446651	3.149264
H	-2.1095	5.315457	1.648811
H	-0.50103	5.080042	2.335872
H	-2.17088	3.055719	0.758137
H	-0.68503	3.810749	0.230477
H	-0.24513	3.714546	4.524193
H	-1.50042	2.547669	4.897202
H	0.350271	1.900542	-4.43966
H	-1.13055	0.197158	-3.46479

H	1.458284	3.551132	0.667557
H	2.811243	2.668811	-0.00054
H	1.62015	5.26473	-1.10627
H	3.190104	5.053389	-0.32783
H	3.838401	3.445776	-2.17909
H	3.517476	5.069968	-2.79624
H	1.316688	4.463444	-3.57014
H	2.391037	3.19787	-4.13486
H	-1.76202	-1.67304	2.001659
H	-3.20439	-0.99216	3.915117
H	-4.82065	0.872605	3.642813
H	-5.01029	2.043356	1.46172
H	-3.57274	1.376809	-0.43404
H	-3.3492	0.658616	-2.79433
H	-5.07321	-0.27413	-4.29123
H	-5.50906	-2.71504	-4.33499
H	-4.22558	-4.23347	-2.84677
H	-2.52312	-3.31531	-1.3145
H	4.201097	-2.10649	0.669706
H	5.240164	-3.8928	2.015566
H	3.866477	-5.25072	3.571294
H	1.42931	-4.81624	3.777745
H	0.372363	-3.02823	2.431277
H	1.690535	-1.55339	-2.43694
H	3.418104	-0.73465	-4.0363
H	5.277602	0.693992	-3.22473
H	5.423923	1.304098	-0.82302
H	3.694709	0.508645	0.767202
H	1.404073	-0.7672	3.316811
H	0.353758	0.934622	4.753214

6. References

[1] K. Yavari, P. Retailleau, A. Voituriez, A. Marinetti, *Chem. Eur. J.* **2013**, *19*, 9939–9947.

[2] X. Sui, T. Zhang, A. B. Pabarue, L. Fu, W. R. Gutekunst, *J. Am. Chem. Soc.* **2020**, *142*, 12942–12947.

[3] T. Kippo, T. Fukuyama, I. Ryu, *Org. Lett.* **2011**, *13*, 3864–3867.

[4] K. S. Kanyiva, T. Marina, S. Nishibe, T. Shibata, *Adv. Synth. Catal.* **2021**, *363*, 2746–2751.

[5] V. Akhmetov, M. Feofanov, D. I. Sharapa, K. Amsharov, *J. Am. Chem. Soc.* **2021**, *143*, 15420–15426.

[6] A. Ghosh, A. C. Brueckner, P. H. Cheong, R. G. Carter, *J. Org. Chem.* **2019**, *84*, 9196–9214.

[7] Q. Wang, Y. Jiang, R. Sun. X. Tang, M. Shi, *Chem. Eur. J.* **2016**, *22*, 14739–14745.

[8] H. Chang, M. Jeganmohan, C. Cheng, *Org. Lett.* **2007**, *9*, 503–508.

[9] M. Bao, W. Lu, H. Su, L. Qiu, X. Xu, *Org. Biomol. Chem.* **2018**, *16*, 3258–3265.

[10] M. J. Frisch, G. W. Trucks, H. B. Schlegel, G. E. Scuseria, M. A. Robb, J. R. Cheeseman, J. A. Montgomery Jr, T. Vreven, K. N. Kudin, J. C. Burant, *Gaussian 16, Revis. C.01* **2004**.

[11] a) A. D. Beche, *Phys. Rev.* **1988**, *A38*, 3098–3100; b) A. D. Beche, *J. Chem. Phys.* **1993**, *98*, 1372–1377; c) A. D. Beche, *J. Chem. Phys.* **1993**, *98*, 5648–5652.

[12] C. Lee, W. Yang, R. G. Parr, *Phys. Rev.* **1988**, *B37*, 785–788.

[13] Y. Zhao, D. G. Truhlar, *Theor. Chem. Acc.* **2008**, *120*, 215–241.

[14] Y. Segawa, A. Yagi, H. Ito, K. Itami, *Org. Lett.* **2016**, *18*, 1430–1433.

[15] C. E. Colwell, T. W. Price, T. Stauch, R. Jasti, *Chem. Sci.* **2020**, *11*, 3923–3930.

[16] W. Humphrey, A. Dalke, K. Schulten, VMD: Visual Molecular Dynamics. *J. Mol. Graph.* **1996**, *14*, 33–38.

[17] S. M. Bachrach, *J. Phys. Chem. A* **2011**, *115*, 2396–2401.

7. Chiral HPLC Charts

[2.2]Triphenylenophane 3a

CHIRALPAK IA, CH_2Cl_2/*n*-hexane = 90:10, 1.0 mL min^{-1}, 40 °C

(±)-**3a**

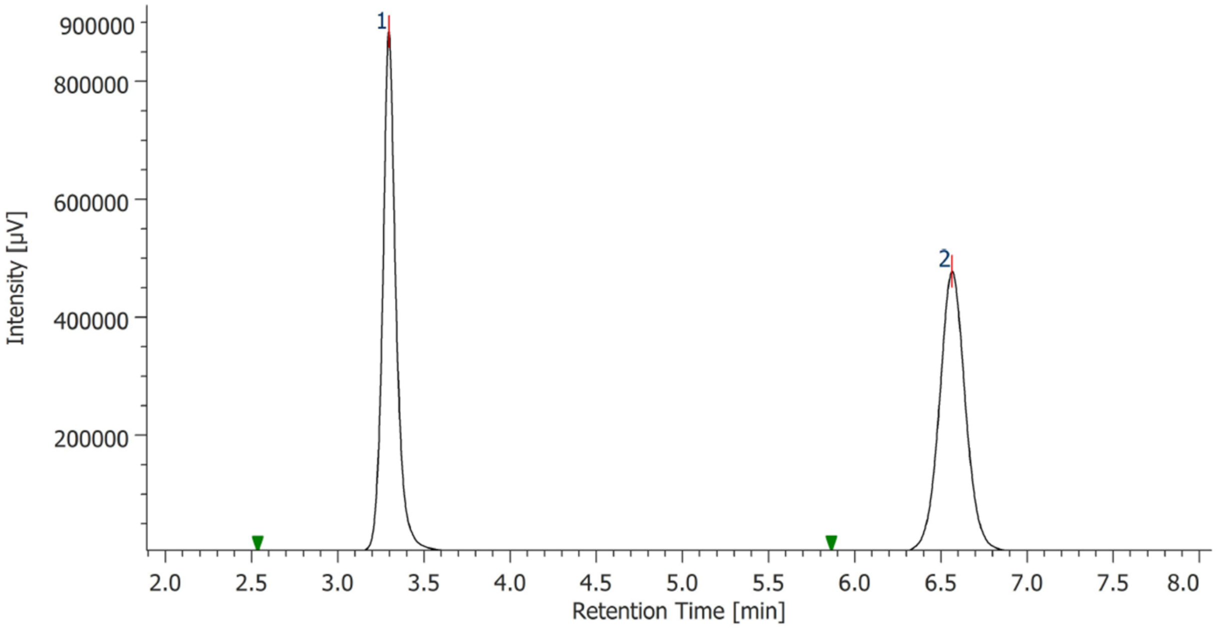

Peak No.	Retention Time (min)	Area (%)
1	3.297	49.088
2	6.563	50.912

(+)-**3a**

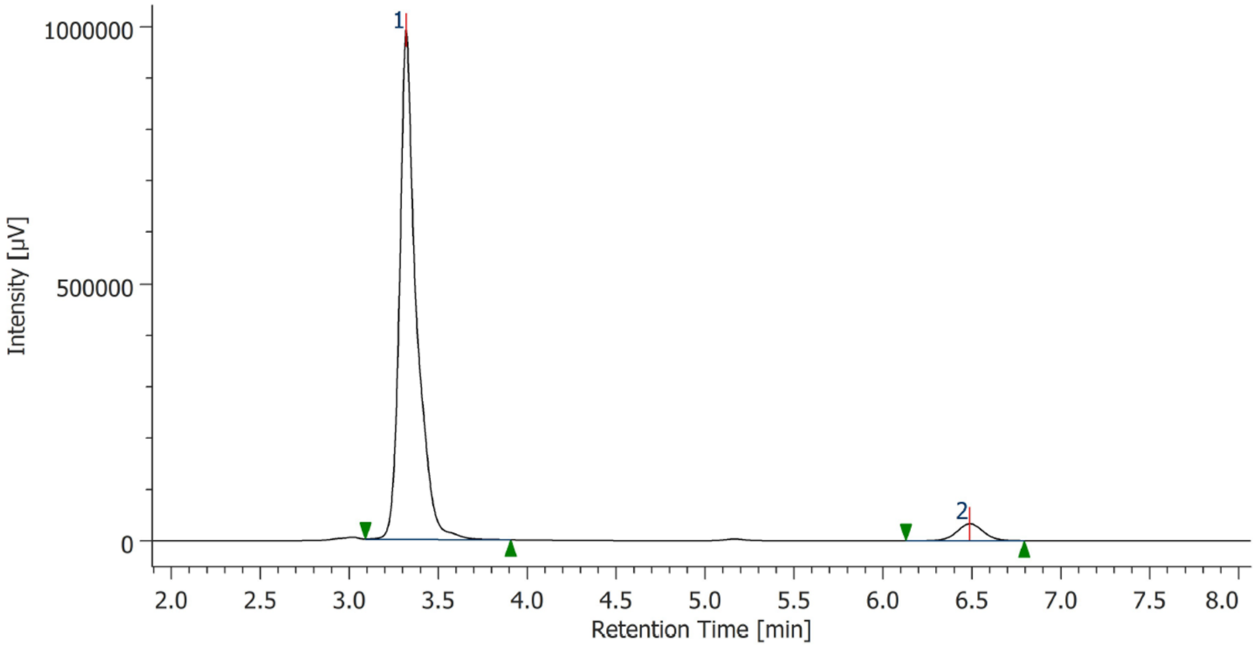

Peak No.	Retention Time (min)	Area (%)
1	3.320	94.925
2	6.487	5.075

[2.2]Triphenylenophane 3b

CHIRALPAK IC, CH_2Cl_2/*n*-hexane = 90:10, 1.0 mL min^{-1}, 40 °C

(±)-**3b**

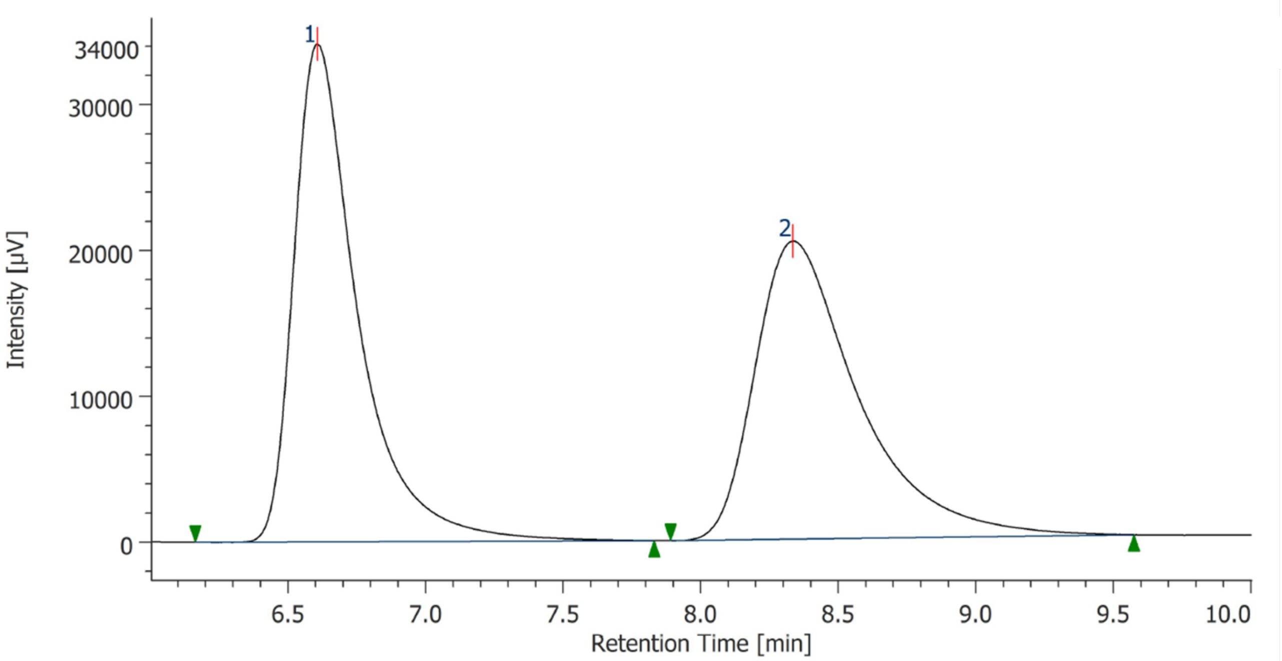

Peak No.	Retention Time (min)	Area (%)
1	6.607	50.880
2	8.333	49.120

(+)-**3b**

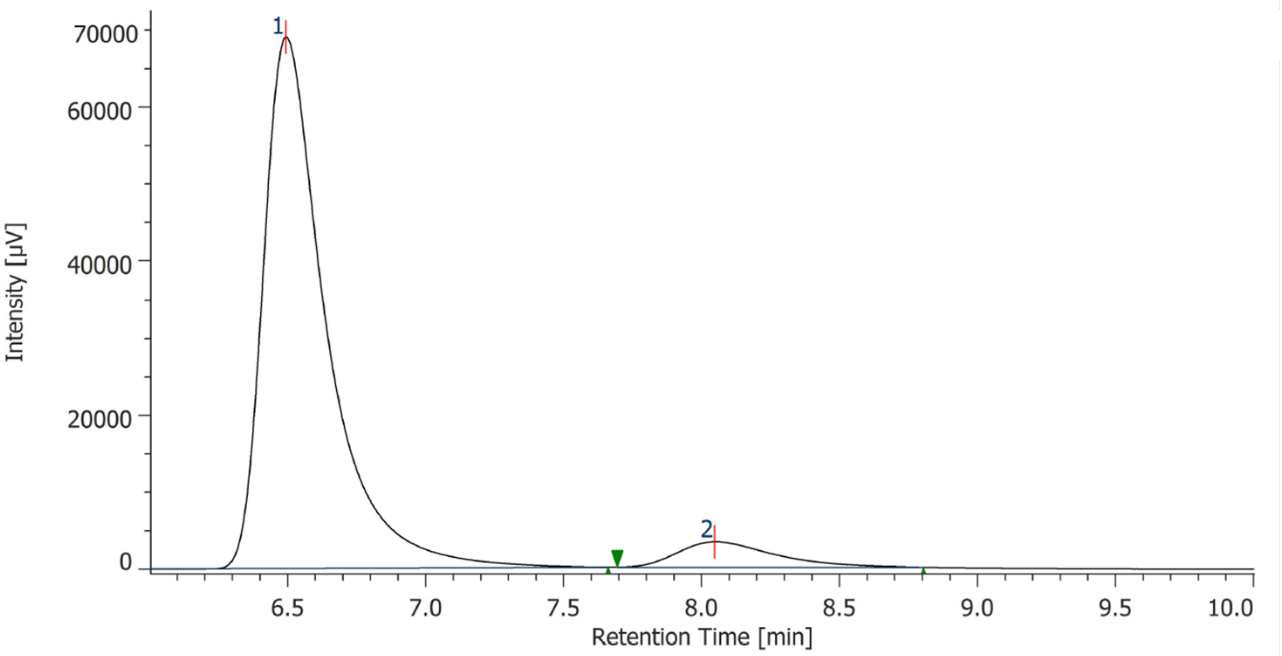

Peak No.	Retention Time (min)	Area (%)
1	6.493	93.091
2	8.047	6.909

[2.2]Triphenylenophane 3c

CHIRALPAK IC, CH_2Cl_2/*n*-hexane = 90:10, 1.0 mL min^{-1}, 40 °C

(±)-**3c**

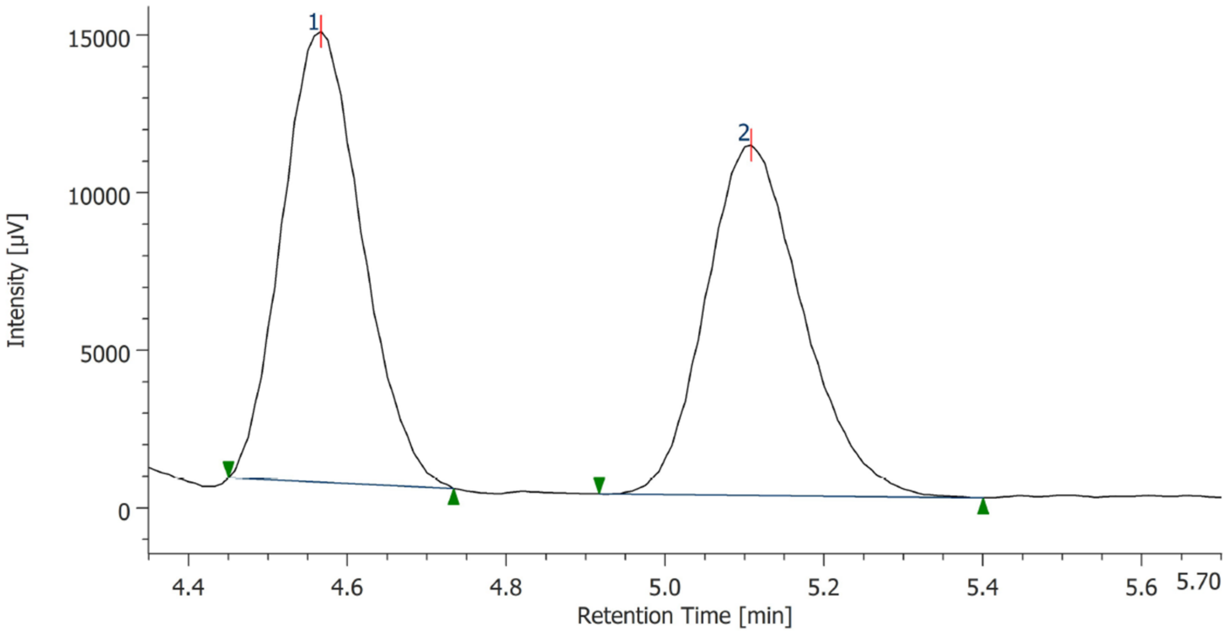

Peak No.	Retention Time (min)	Area (%)
1	4.567	51.444
2	5.108	48.556

(+)-**3c**

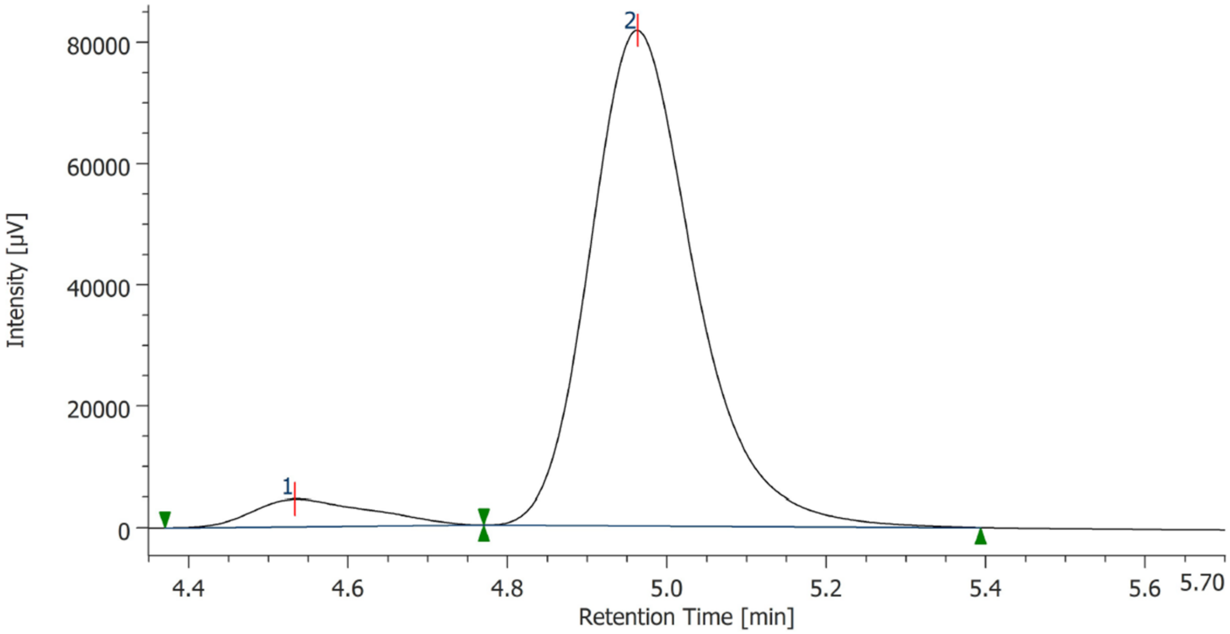

Peak No.	Retention Time (min)	Area (%)
1	4.533	5.916
2	4.963	94.084

[2.2][5]Helicenophane 3e

CHIRALPAK IE, CH_2Cl_2/*n*-hexane = 50:50, 1.0 mL min^{-1}, 40 °C

(±)-**3e**

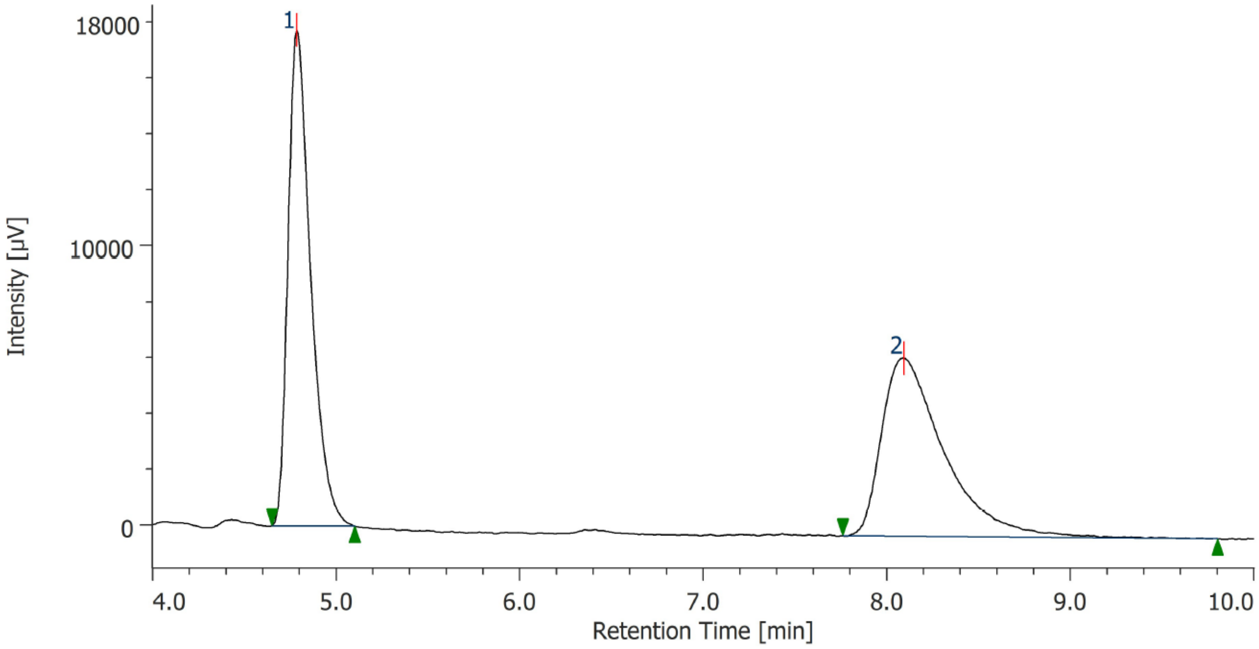

Peak No.	Retention Time (min)	Area (%)
1	4.783	50.34
2	8.092	49.66

(+)-**3e**

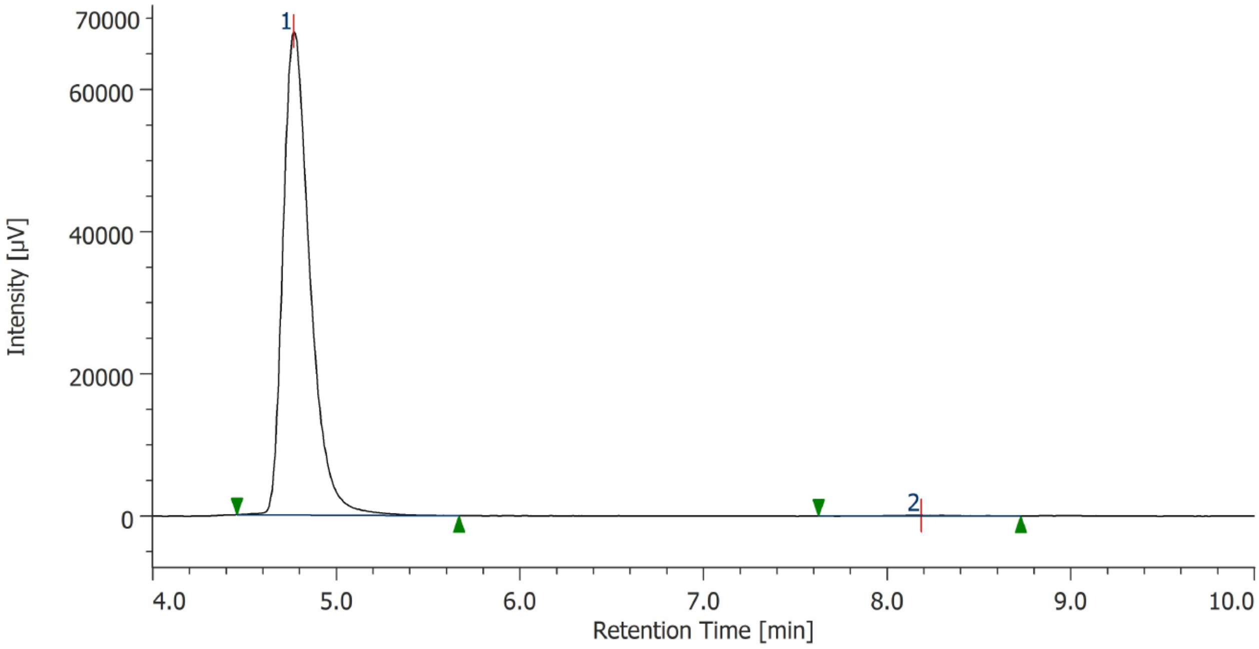

Peak No.	Retention Time (min)	Area (%)
1	4.767	99.734
2	8.183	0.266

(−)-**3e**

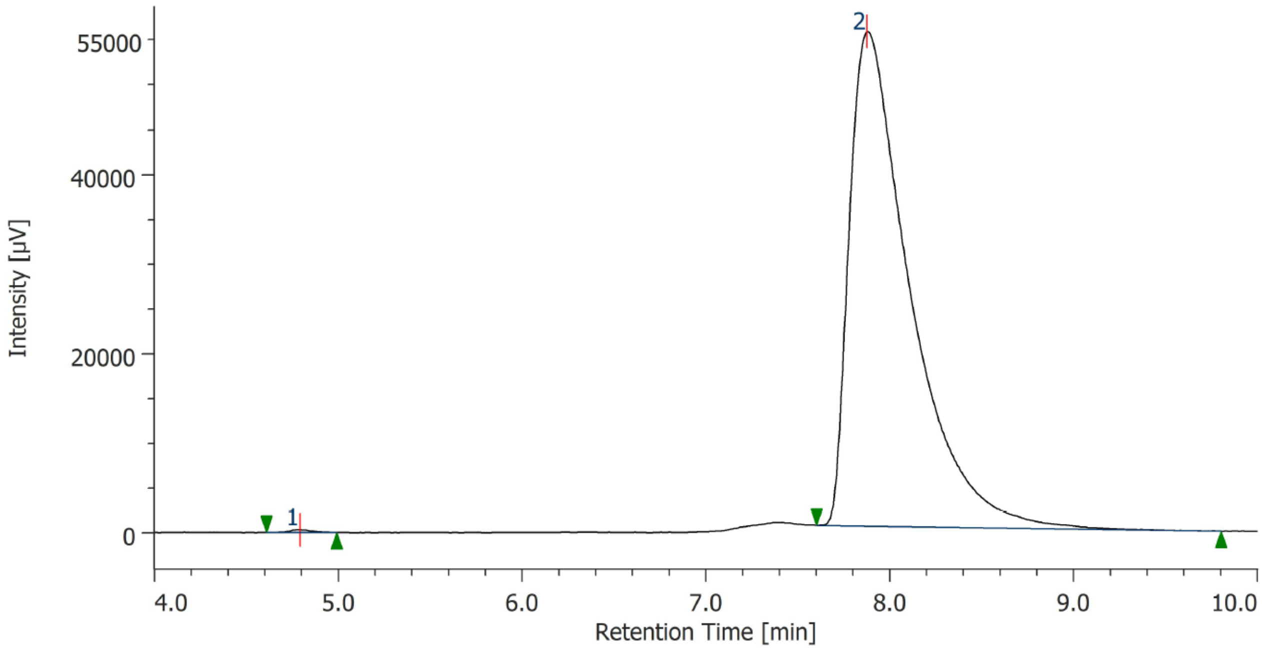

Peak No.	Retention Time (min)	Area (%)
1	4.792	0.209
2	7.875	99.791

8. ^{1}H and ^{13}C NMR Spectra of New Compounds

1-Bromo-2-iodo-4-(methoxymethoxy)benzene (5)

^{1}H NMR ($CDCl_3$, 400 MHz)

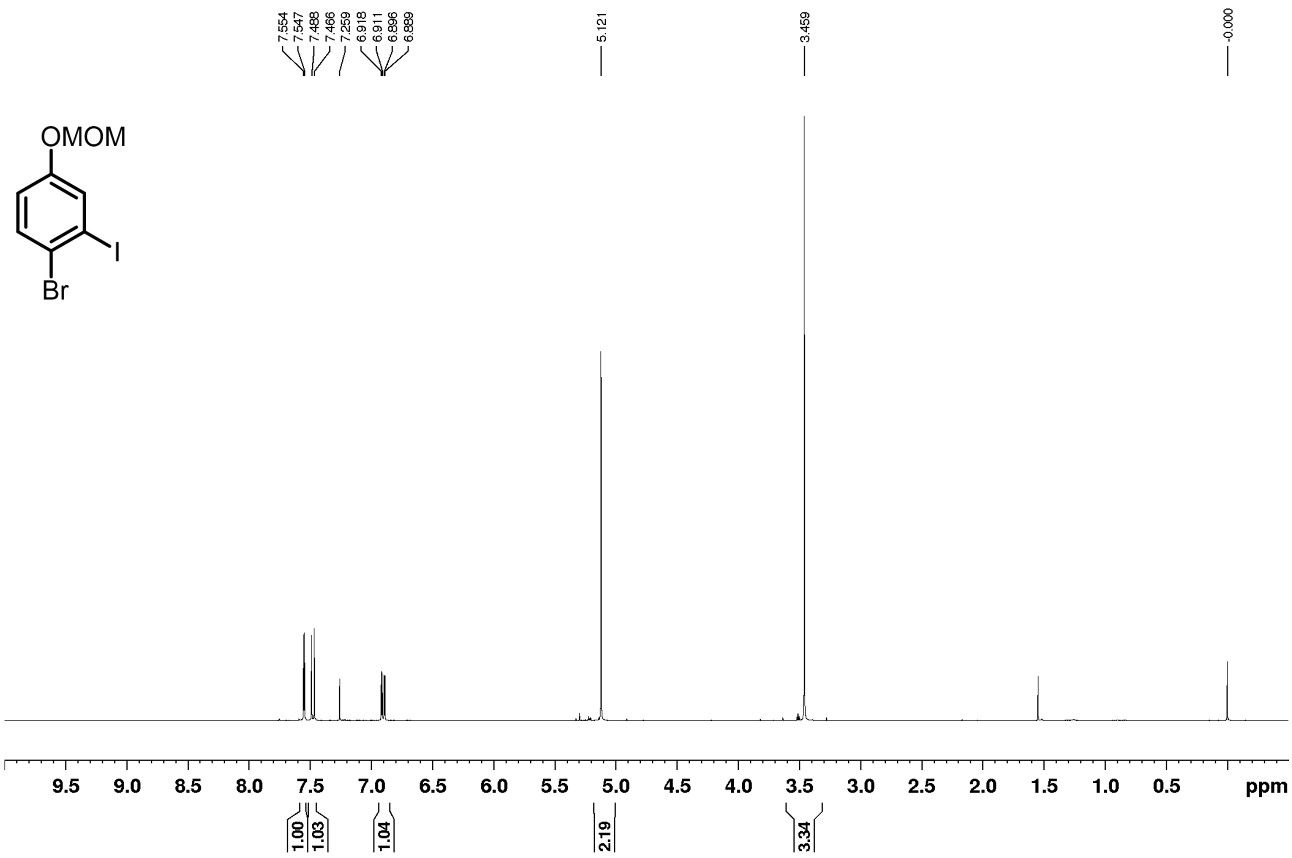

^{13}C NMR ($CDCl_3$, 100 MHz)

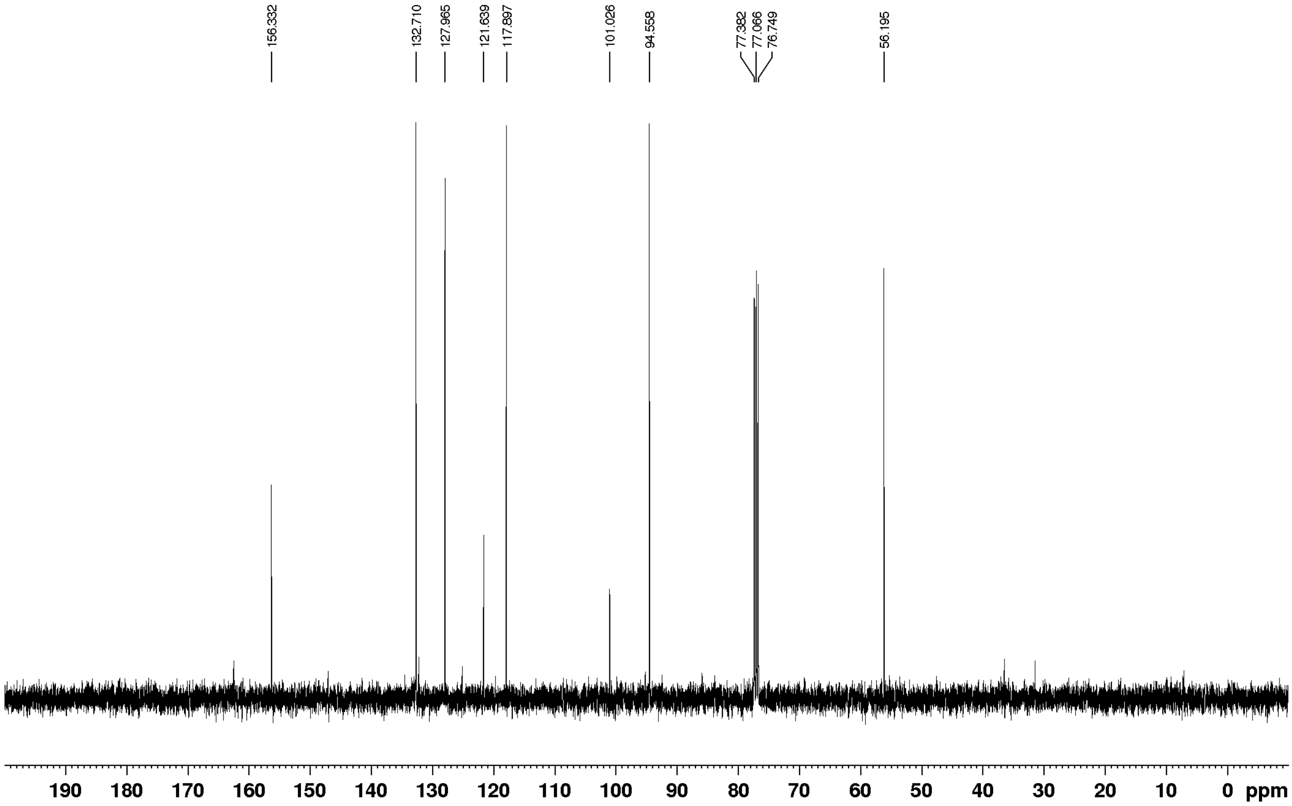

_N_-(Hept-2-yn-1-yl)-4-nitro-_N_-(prop-2-yn-1-yl)benzenesulfonamide (6c)

^{1}H NMR ($CDCl_3$, 400 MHz)

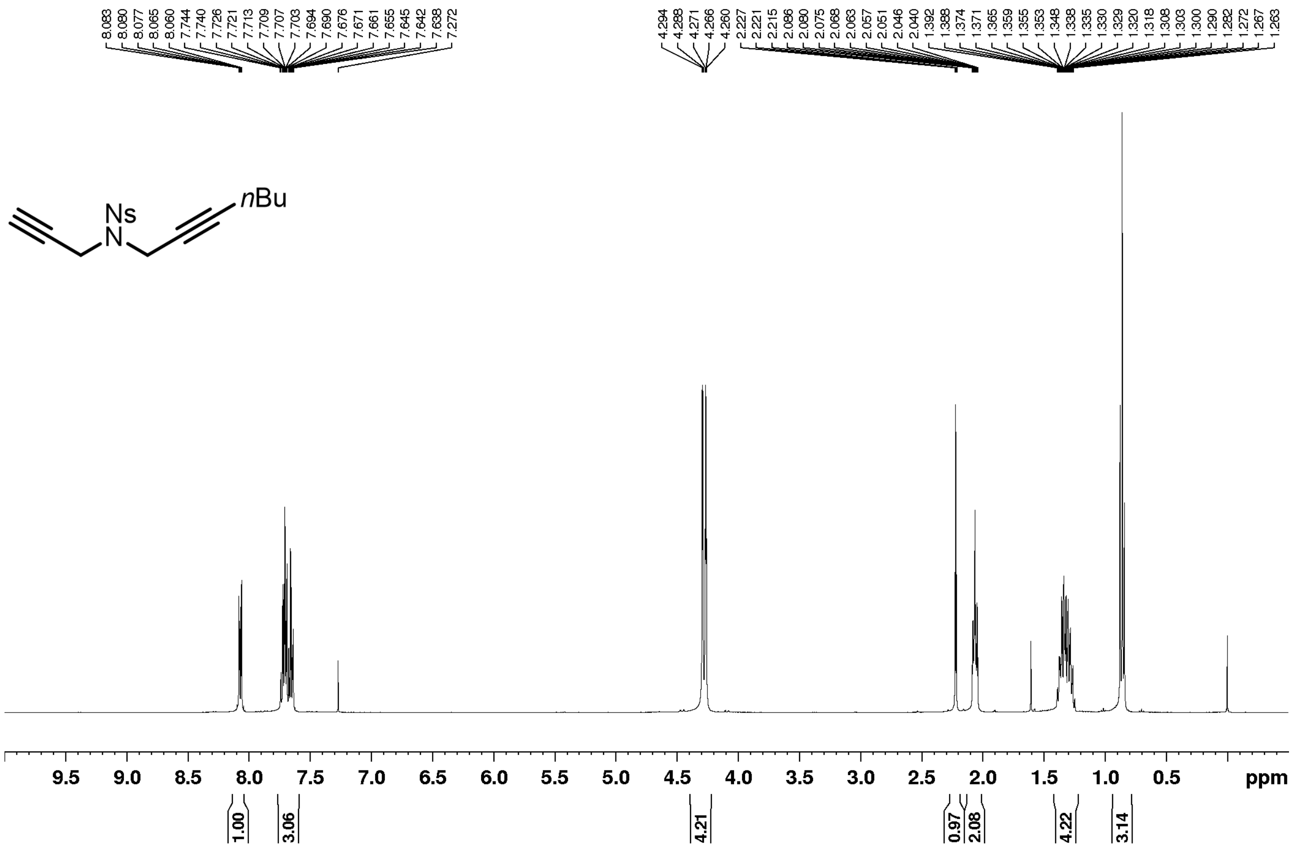

^{13}C NMR ($CDCl_3$, 100 MHz)

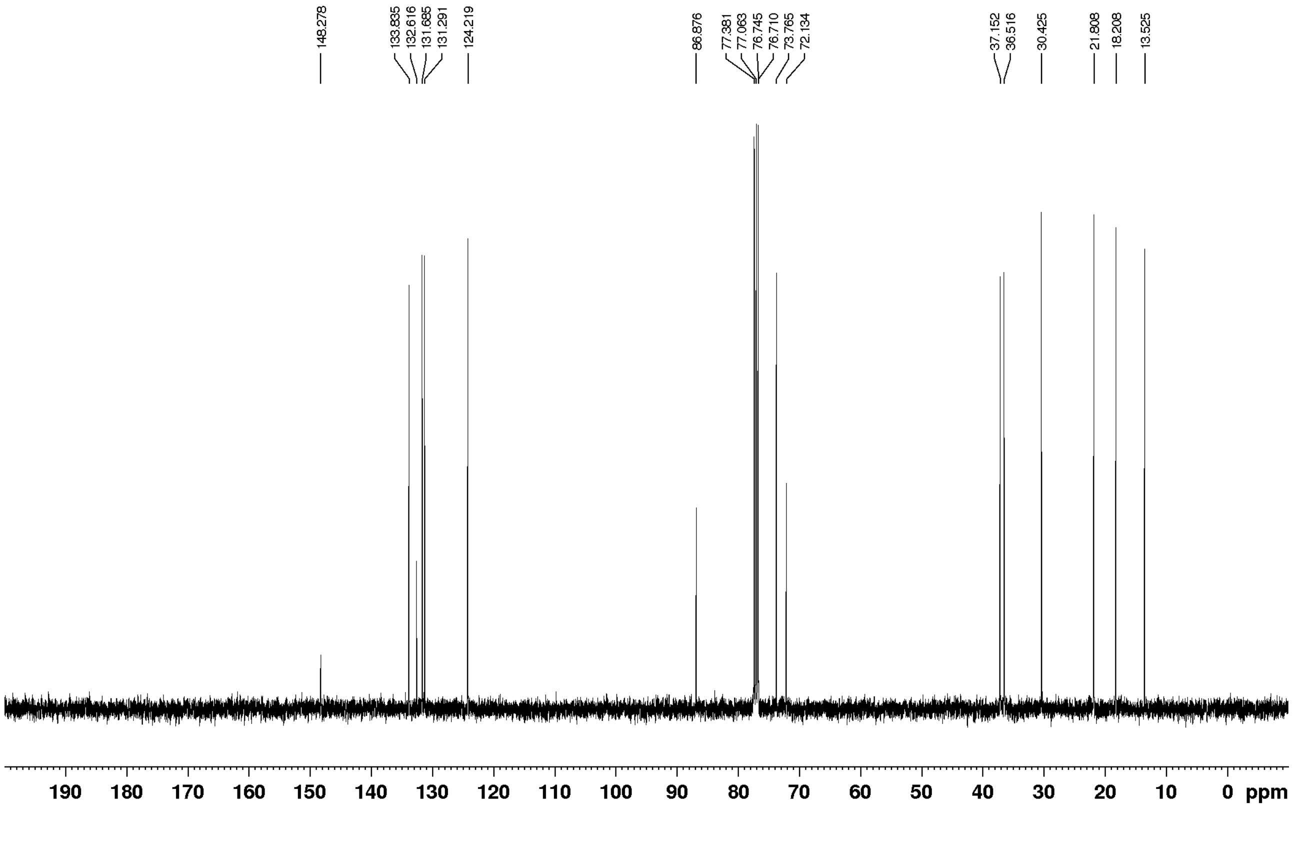

2-Ethynyl-2'-(hex-1-yn-1-yl)-1,1'-biphenyl (6e)

^{1}H NMR ($CDCl_3$, 400 MHz)

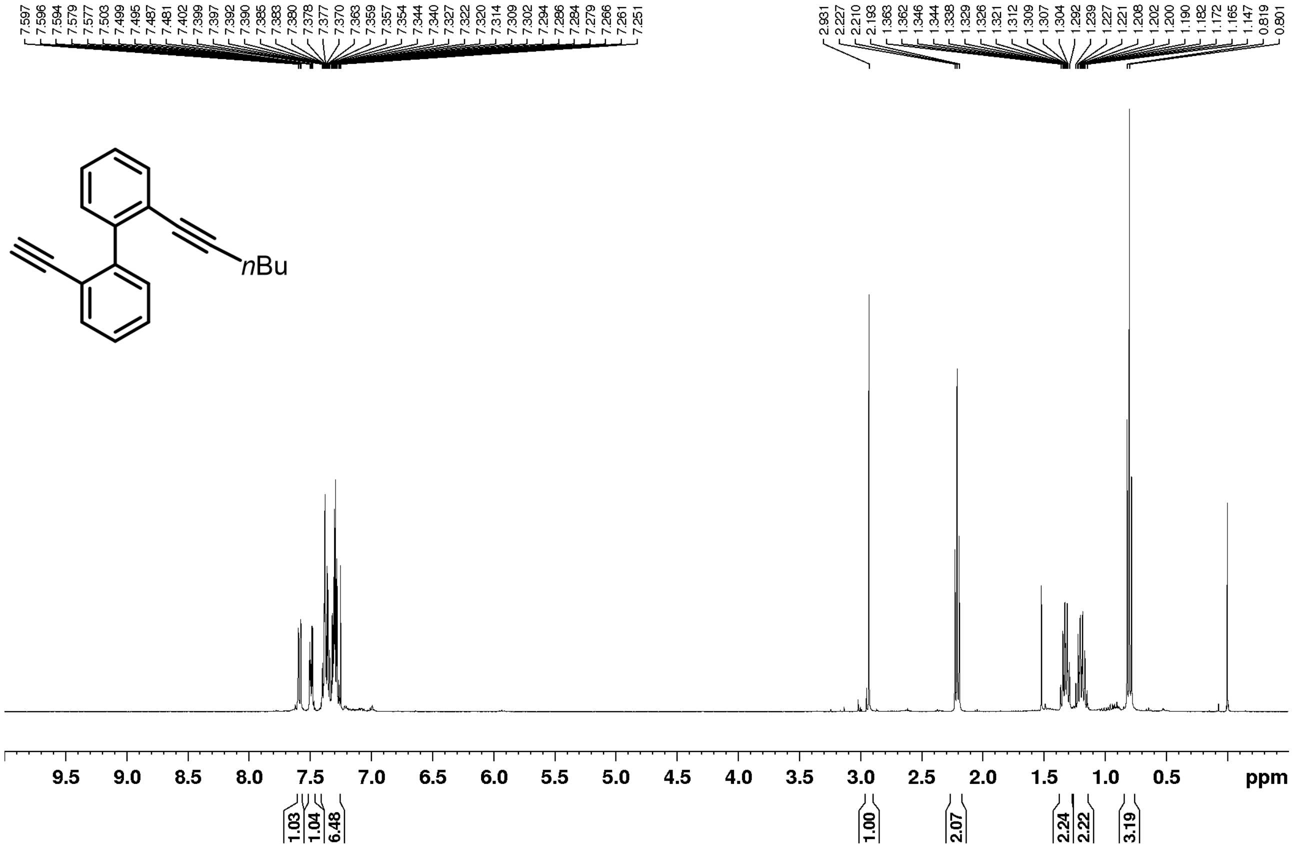

^{13}C NMR ($CDCl_3$, 100 MHz)

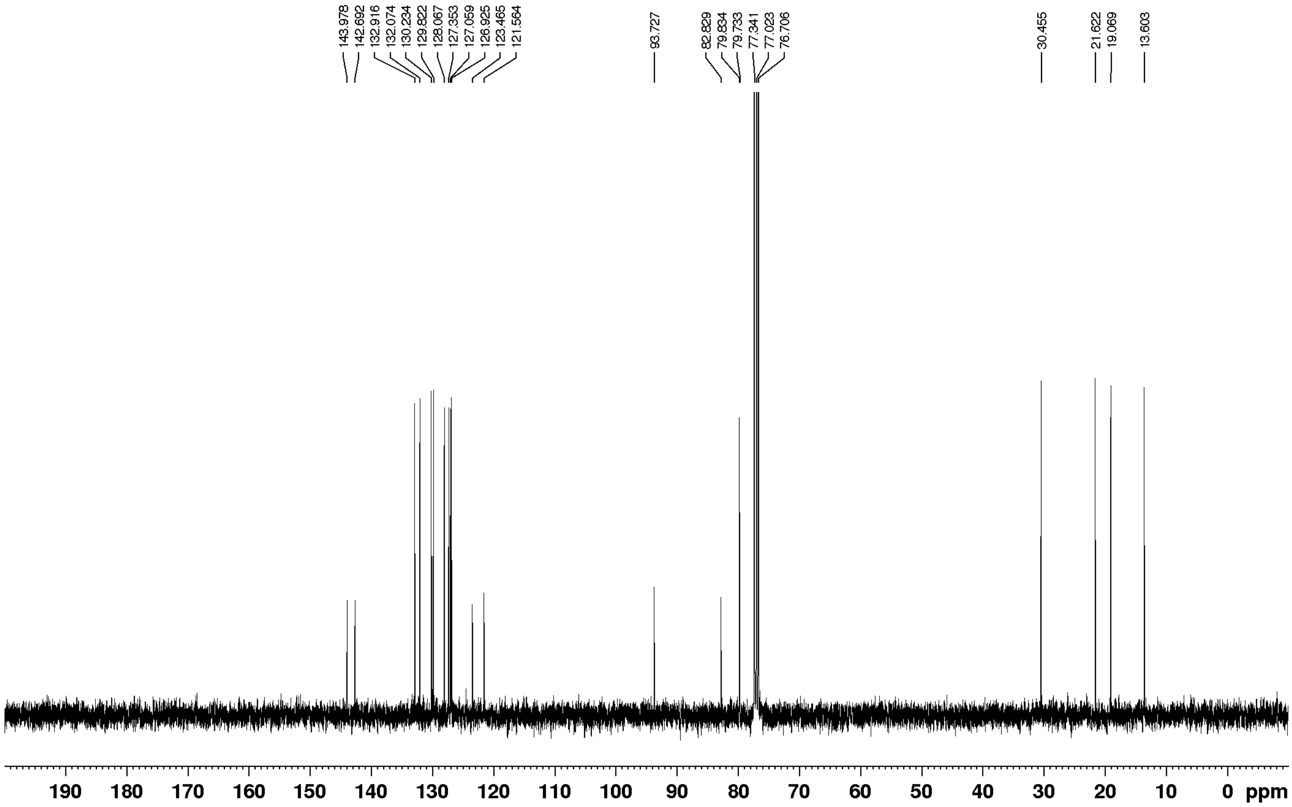

_N_-(3-(2-Bromo-5-(methoxymethoxy)phenyl)prop-2-yn-1-yl)-_N_-(but-2-yn-1-yl)-4-methylbenzenesulfonamide (7a)

^{1}H NMR ($CDCl_3$, 400 MHz)

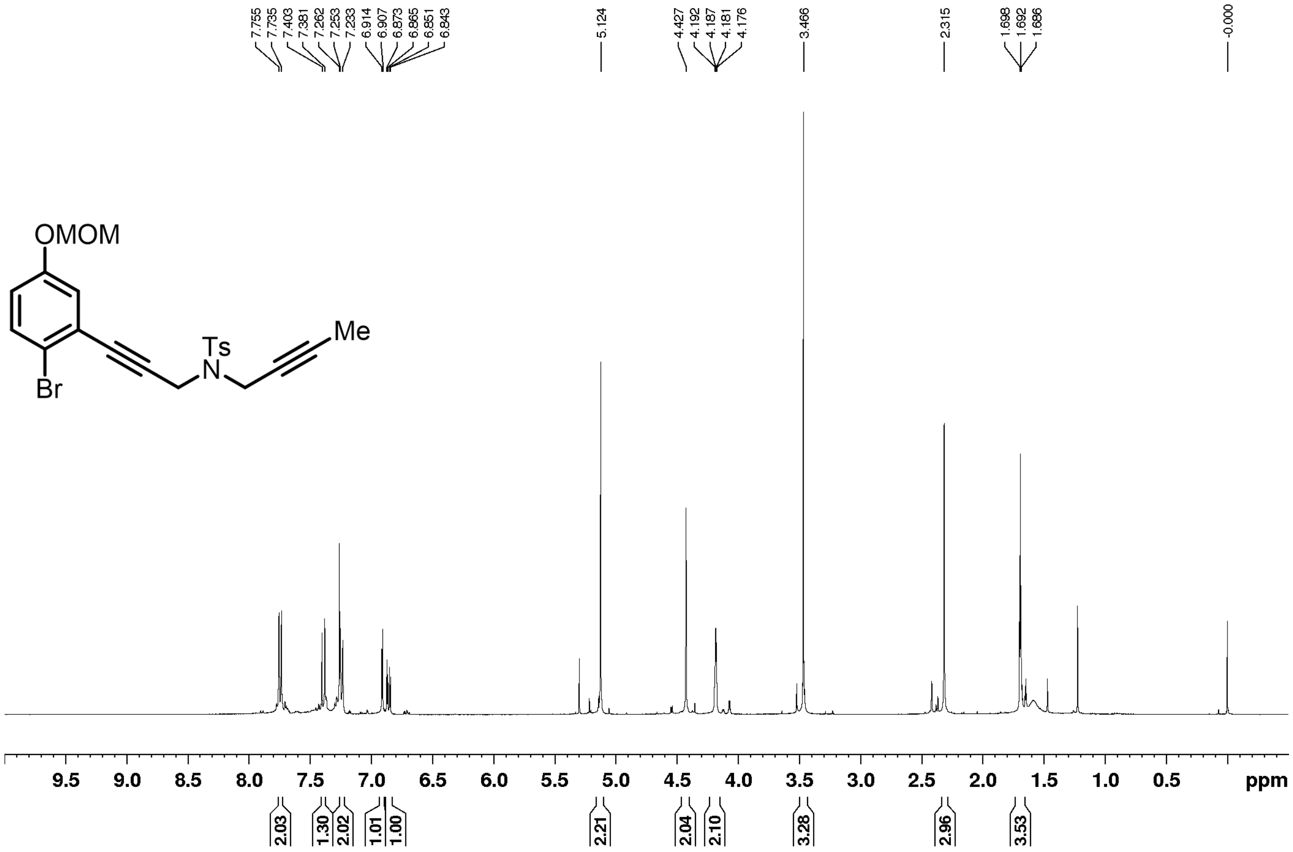

^{13}C NMR ($CDCl_3$, 100 MHz)

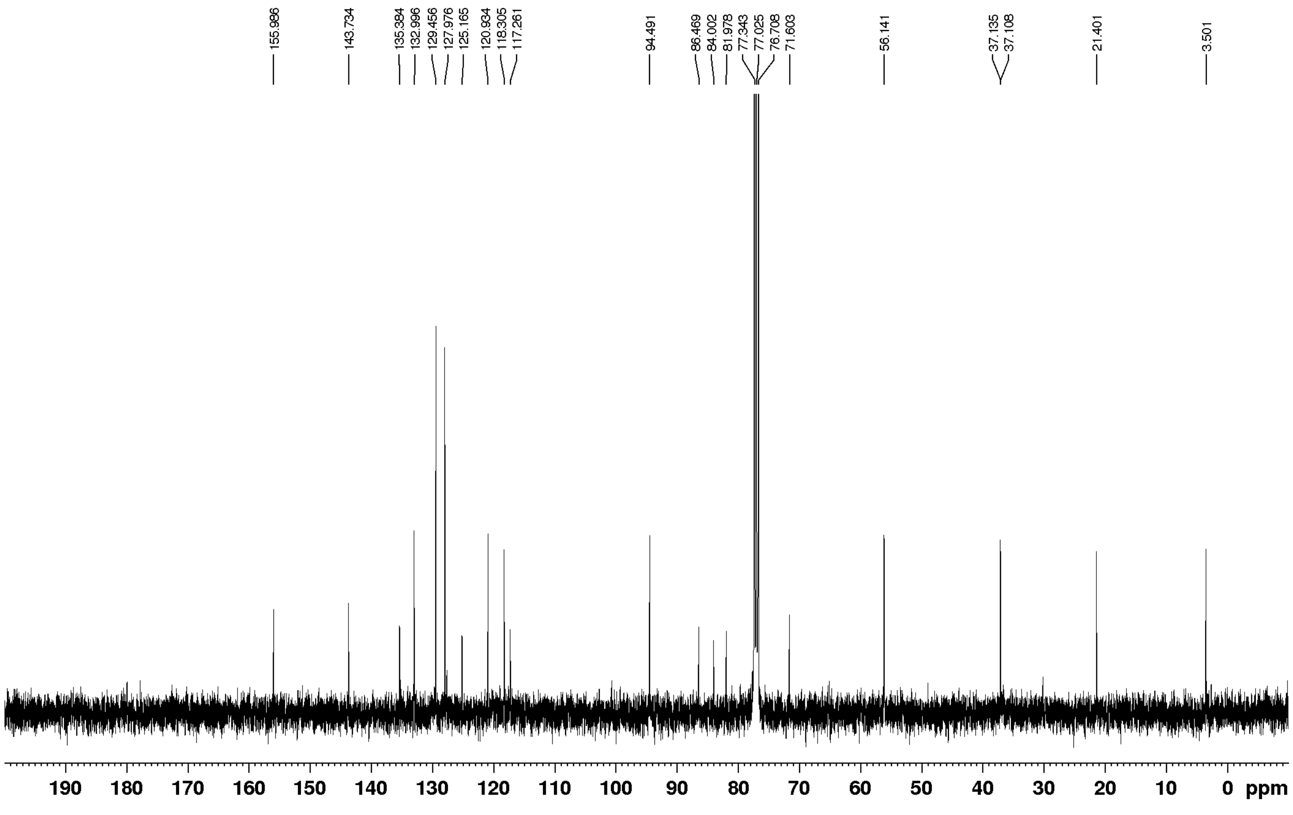

1-Bromo-2-(3-(but-2-yn-1-yloxy)prop-1-yn-1-yl)-4-(methoxymethoxy)benzene (7b)

^{1}H NMR ($CDCl_3$, 400 MHz)

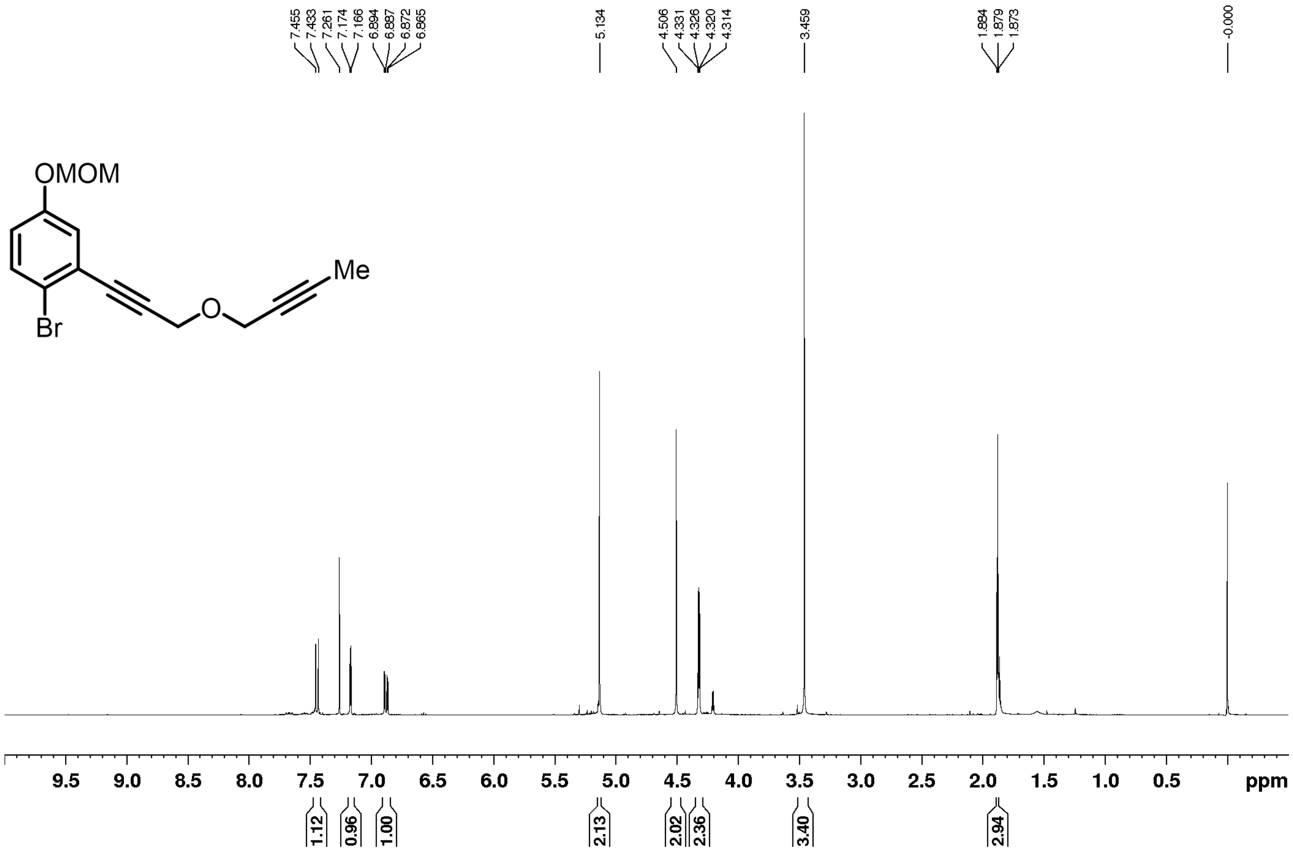

^{13}C NMR ($CDCl_3$, 100 MHz)

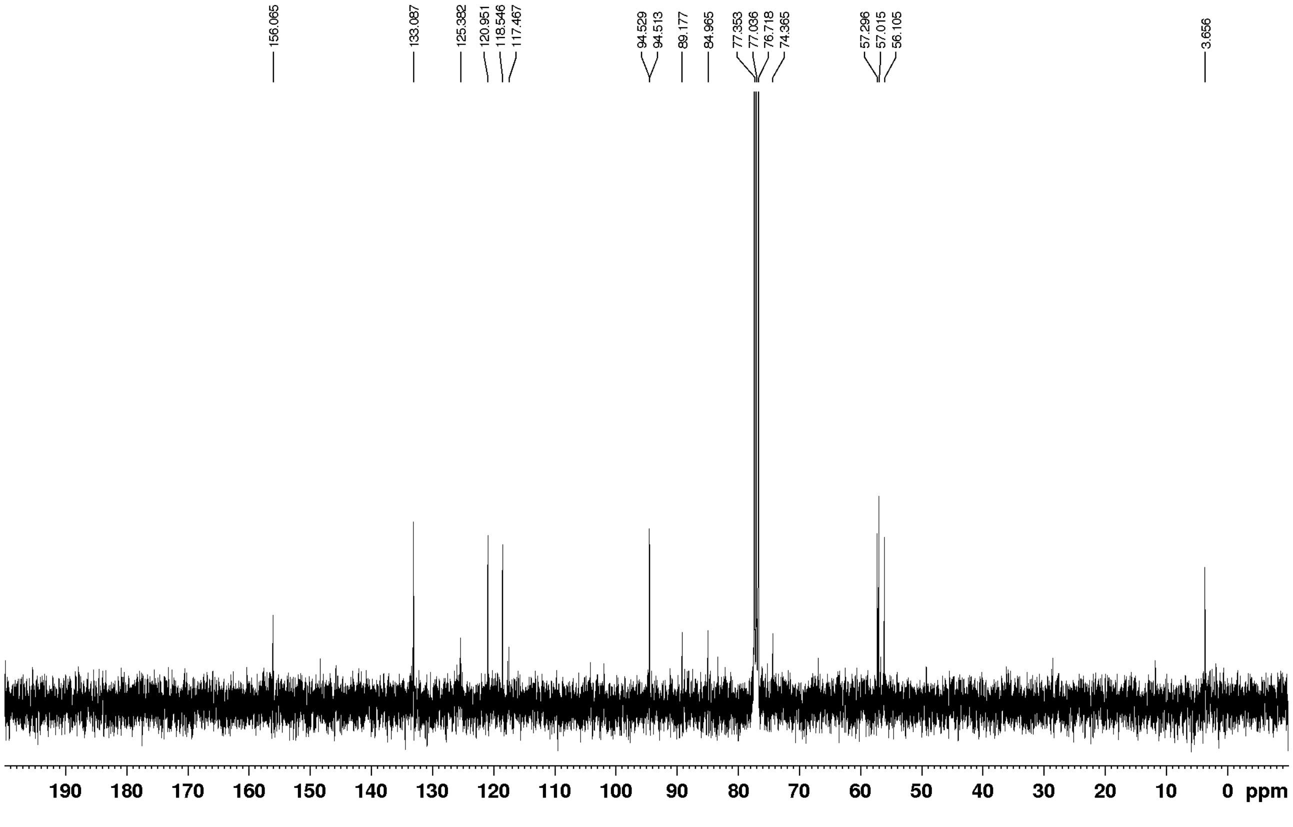

_N_-(3-(2-Bromo-5-(methoxymethoxy)phenyl)prop-2-yn-1-yl)-_N_-(hept-2-yn-1-yl)-4-nitrobenzenesulfonamide (7c)

^{1}H NMR ($CDCl_3$, 400 MHz)

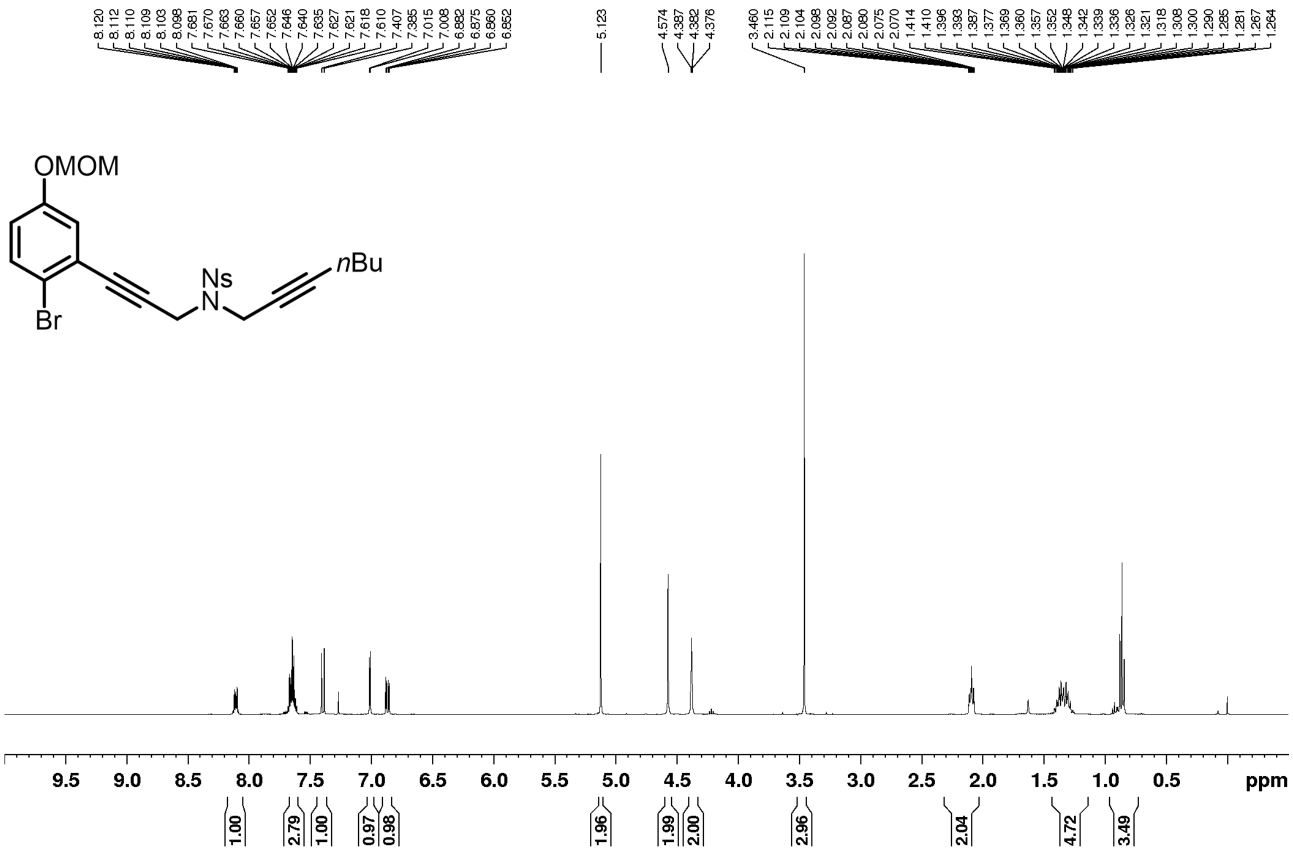

^{13}C NMR ($CDCl_3$, 100 MHz)

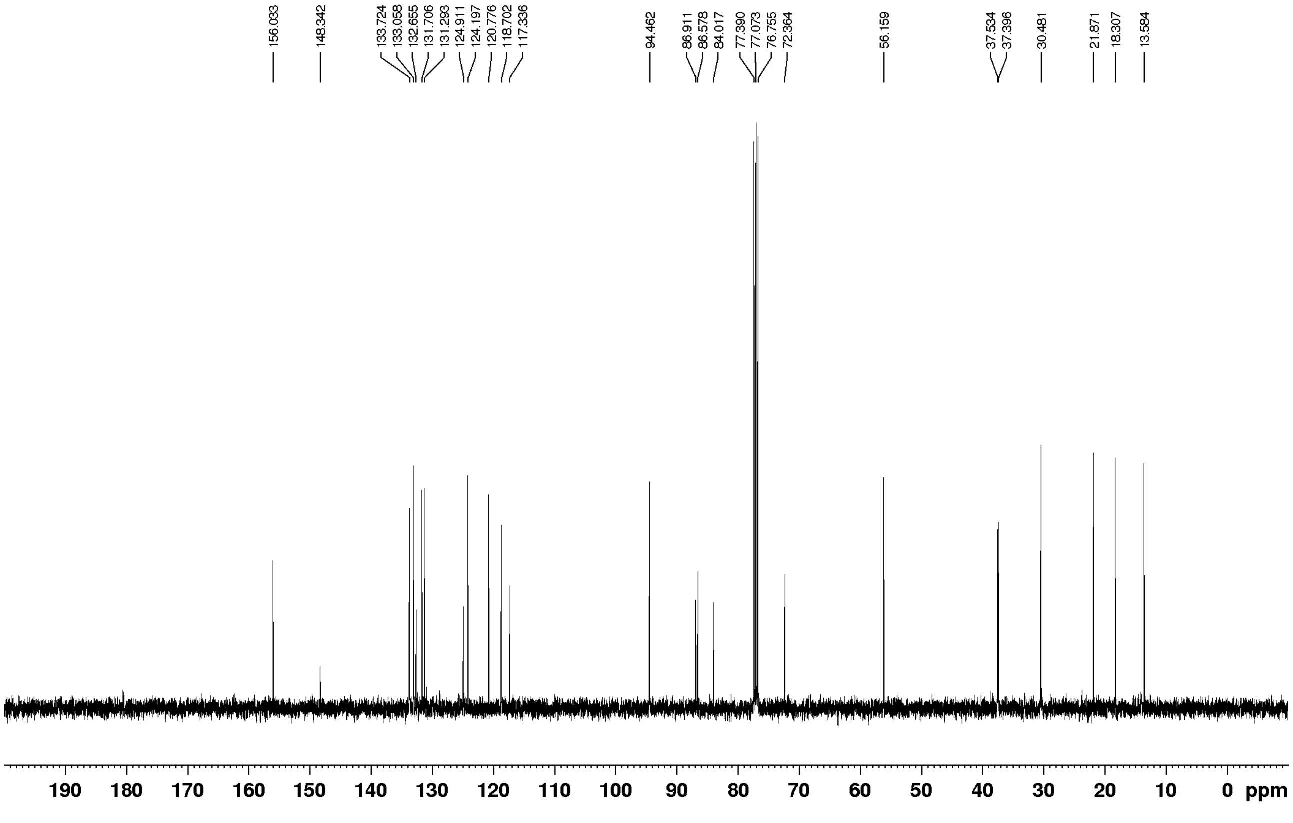

N-(3-(2-Bromo-5-(methoxymethoxy)phenyl)prop-2-yn-1-yl)-4-methyl-N-(3-phenylprop-2-yn-1-yl)benzenesulfonamide (7d)

^{1}H NMR ($CDCl_3$, 400 MHz)

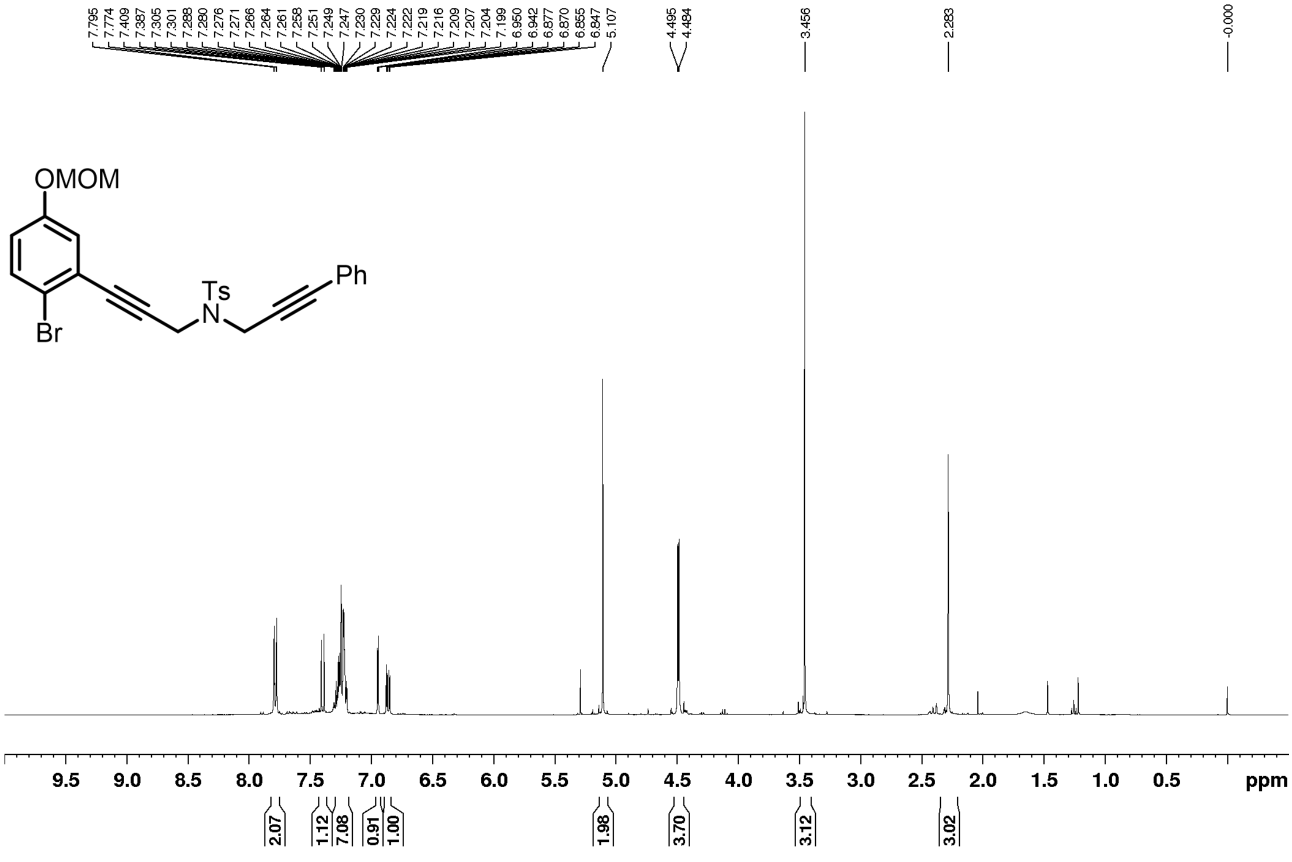

^{13}C NMR ($CDCl_3$, 100 MHz)

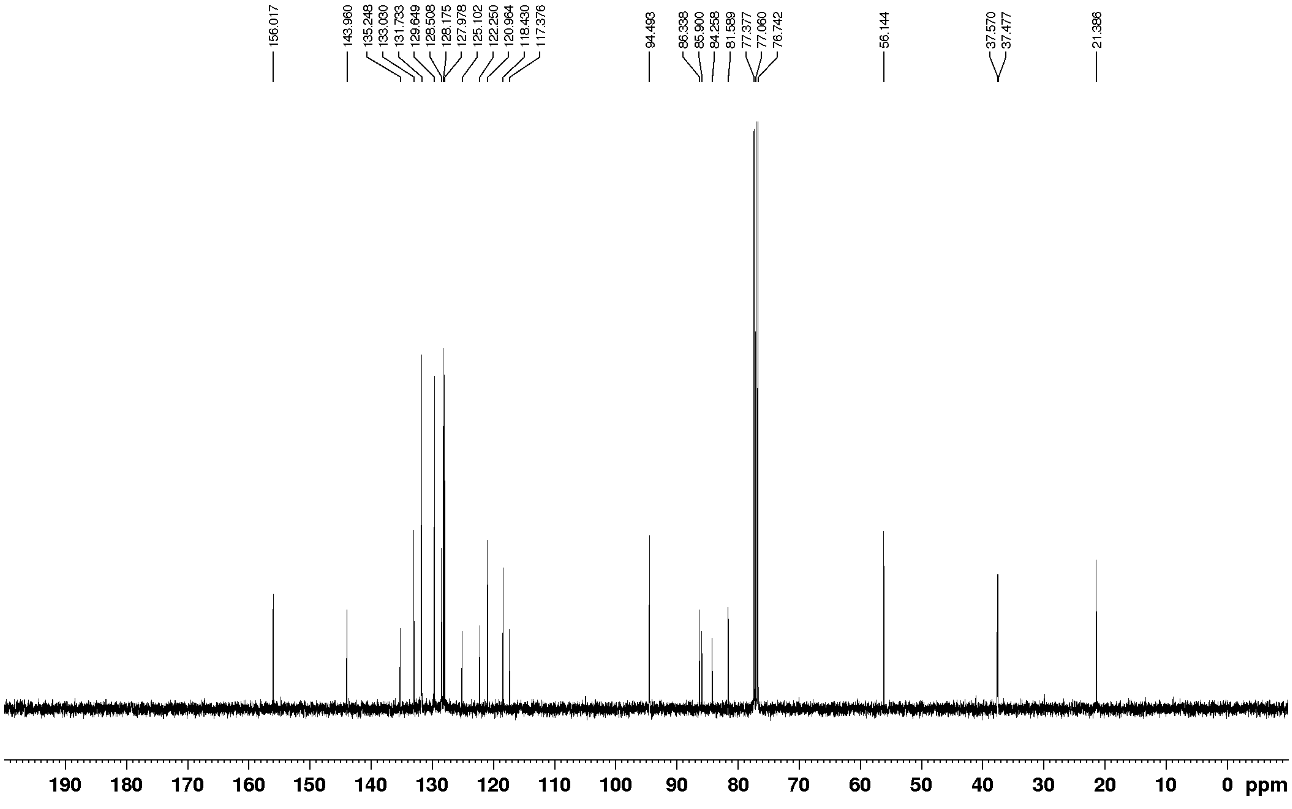

2-((2-Bromo-5-(methoxymethoxy)phenyl)ethynyl)-2'-(hex-1-yn-1-yl)-1,1'-biphenyl (7e)

^{1}H NMR ($CDCl_3$, 400 MHz)

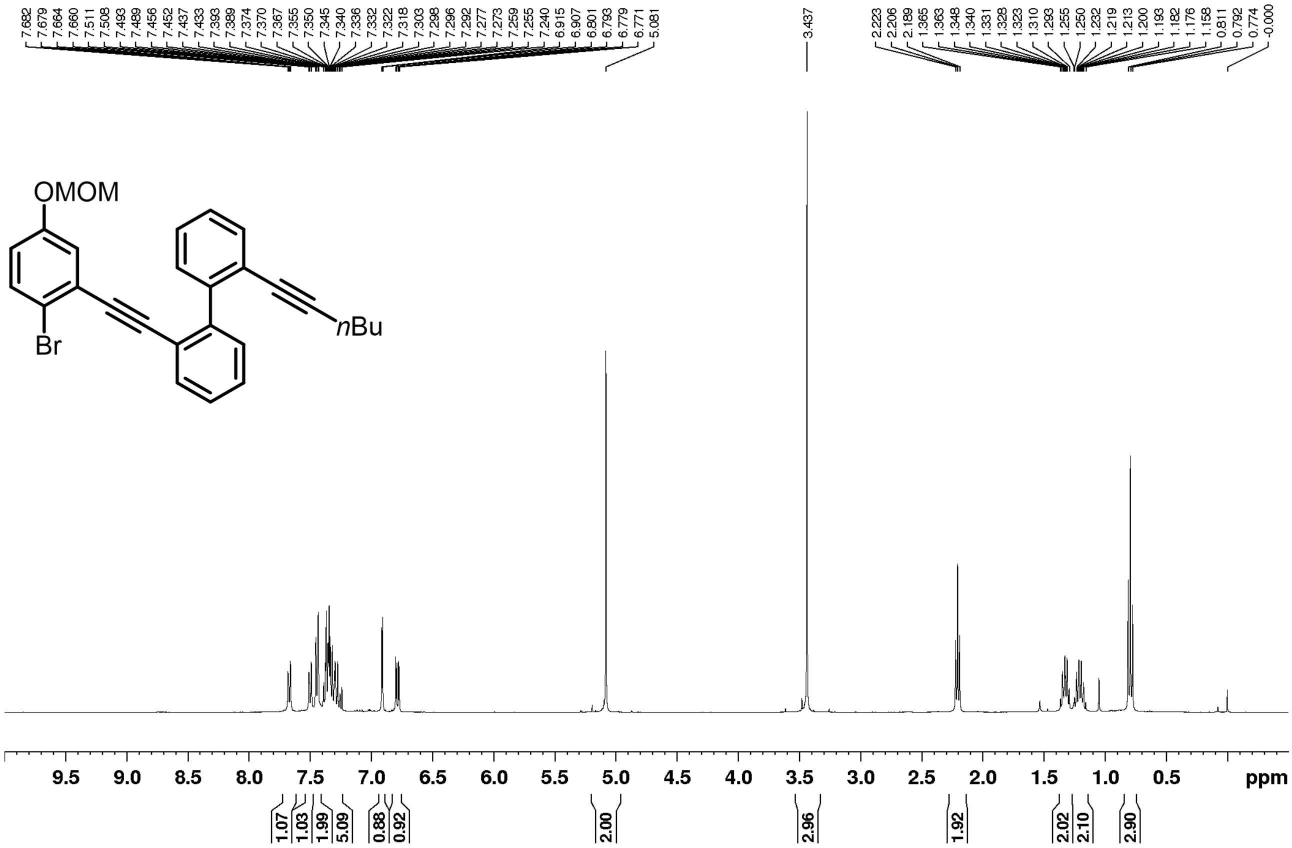

^{13}C NMR ($CDCl_3$, 100 MHz)

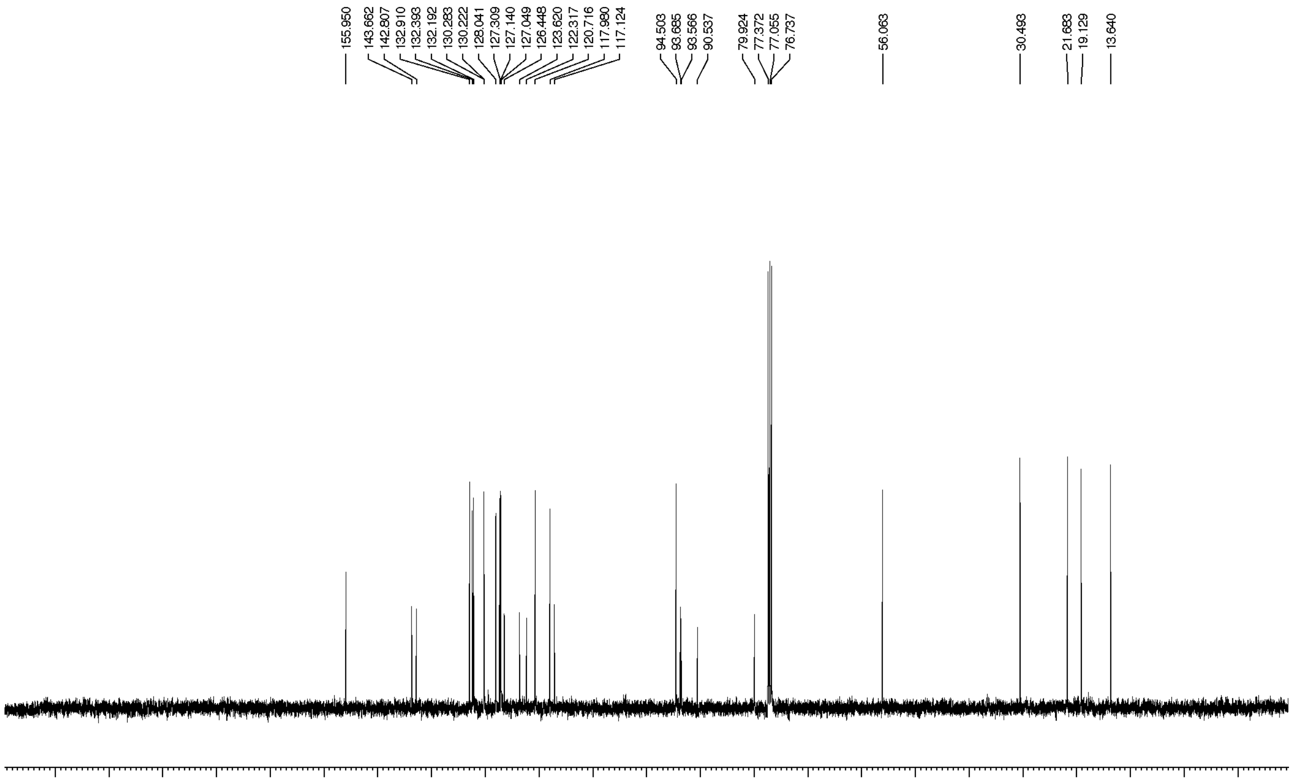

((3-(2-Bromophenyl)prop-2-yn-1-yl)oxy)(*tert*-butyl)diphenylsilane (S8)

^{1}H NMR ($CDCl_3$, 400 MHz)

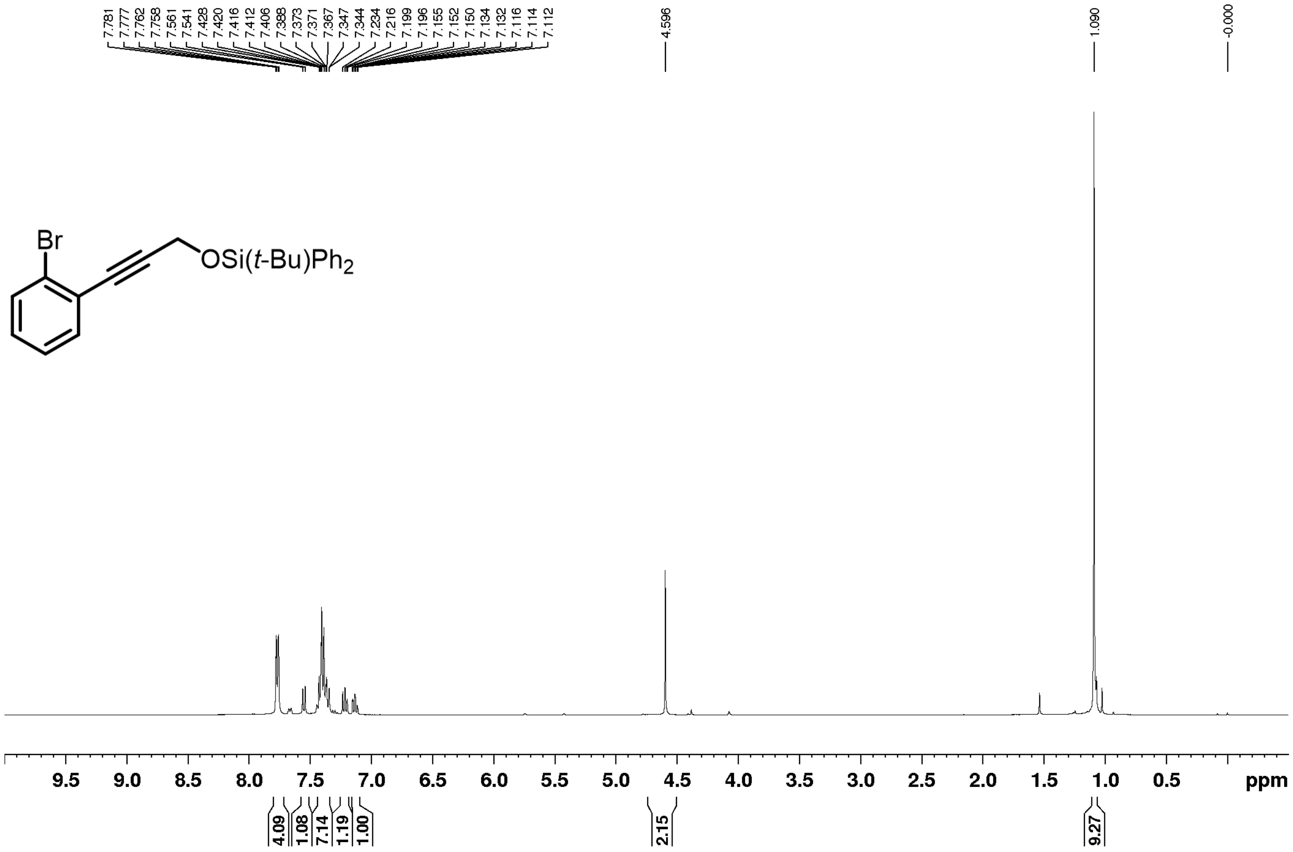

^{13}C NMR ($CDCl_3$, 100 MHz)

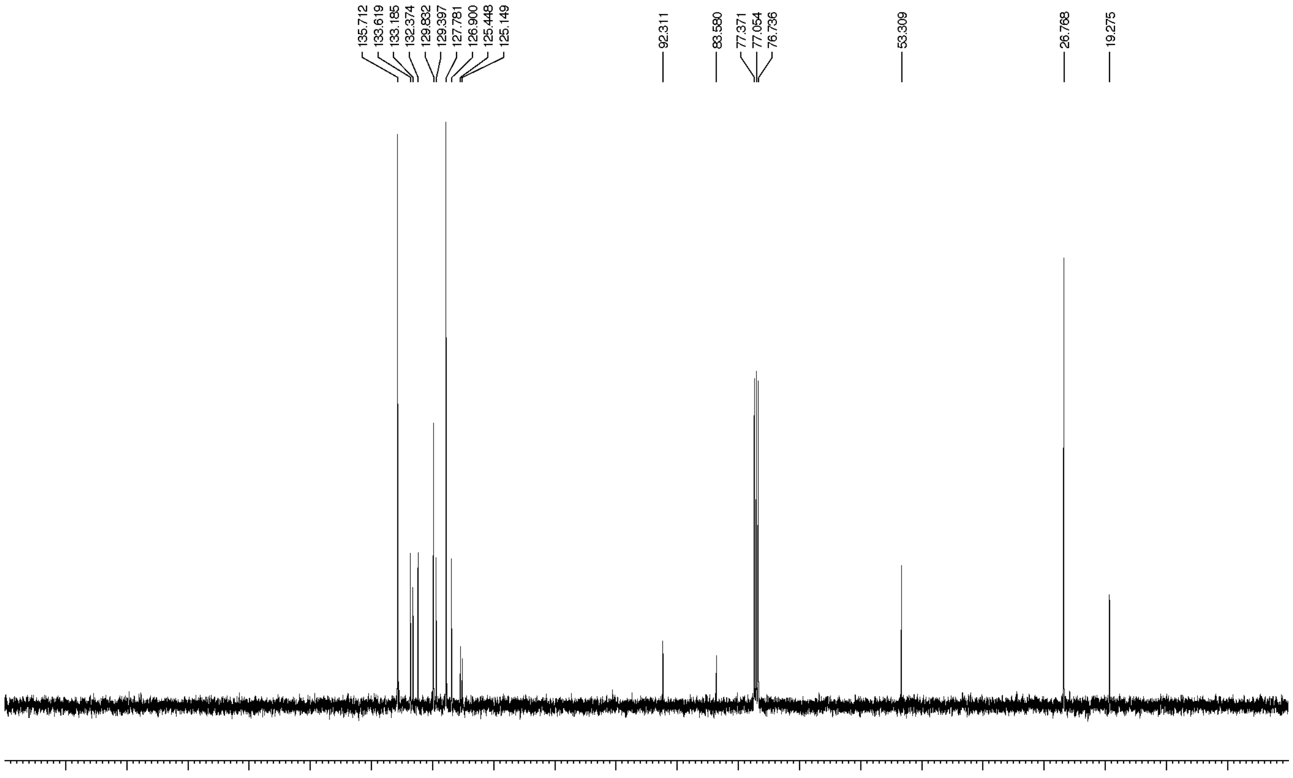

(2-(3-((*tert*-Butyldiphenylsilyl)oxy)prop-1-yn-1-yl)phenyl)boronic acid (8)

^{1}H NMR ($CDCl_3$, 400 MHz)

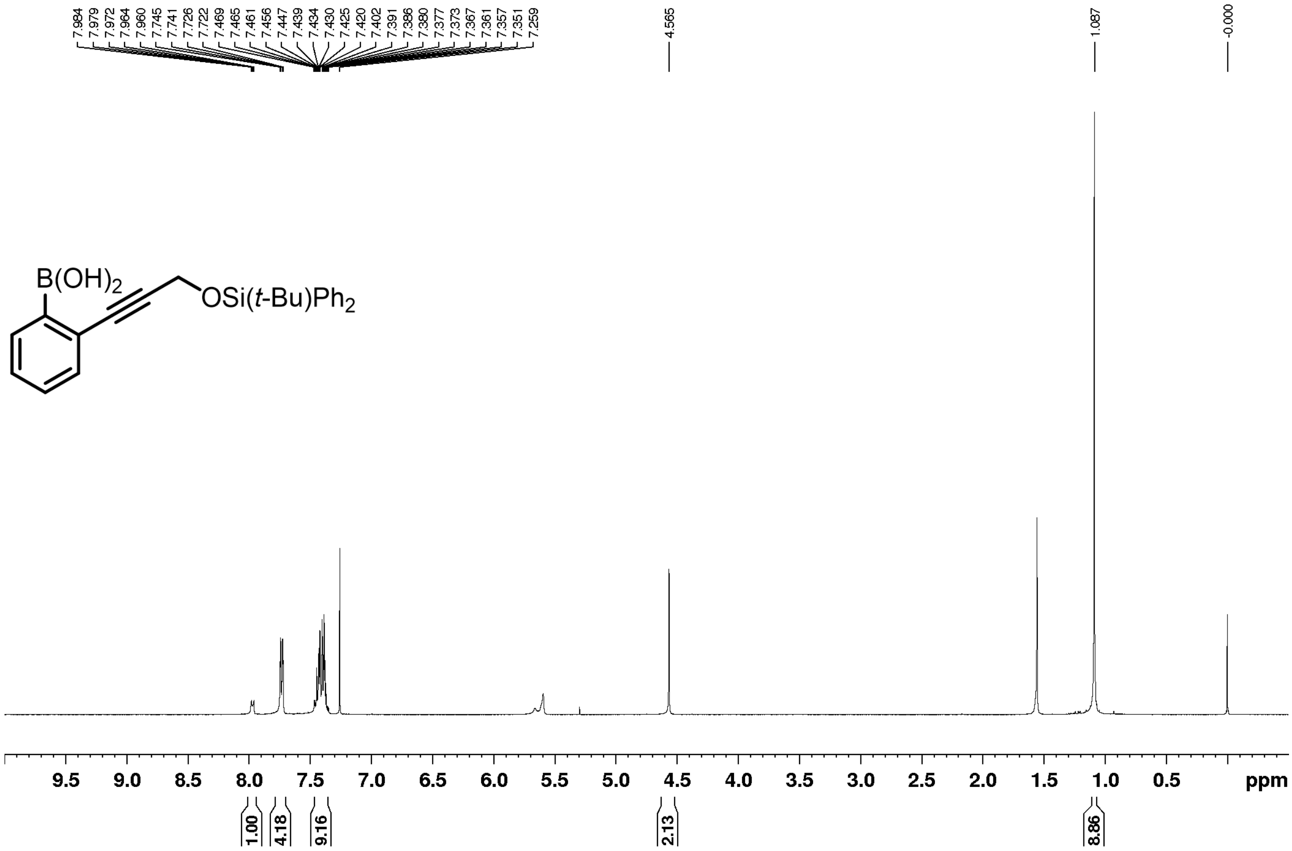

^{13}C NMR ($CDCl_3$, 100 MHz)

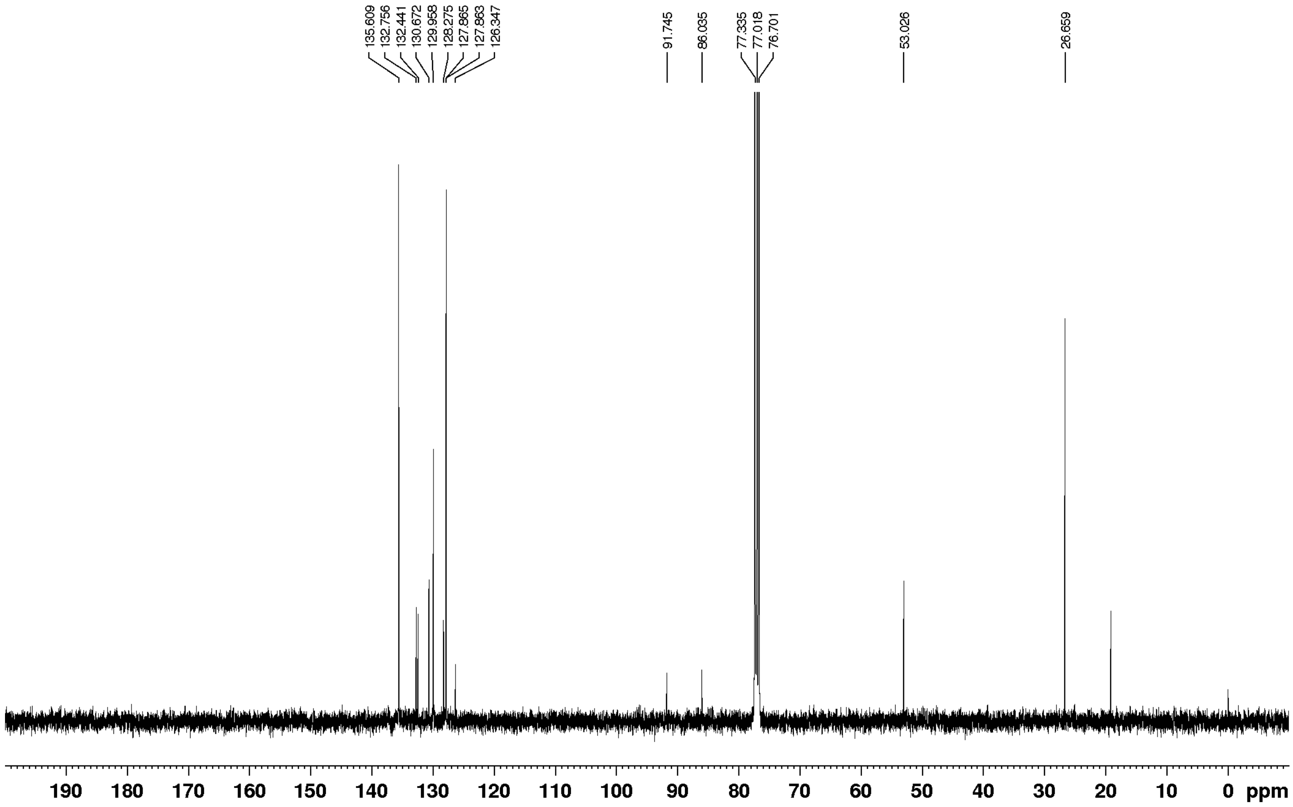

N-(But-2-yn-1-yl)-N-(3-(2'-(3-hydroxyprop-1-yn-1-yl)-4-(methoxymethoxy)-[1,1'-biphenyl]-2-yl)prop-2-yn-1-yl)-4-methylbenzenesulfonamide (9a)

^{1}H NMR ($CDCl_3$, 400 MHz)

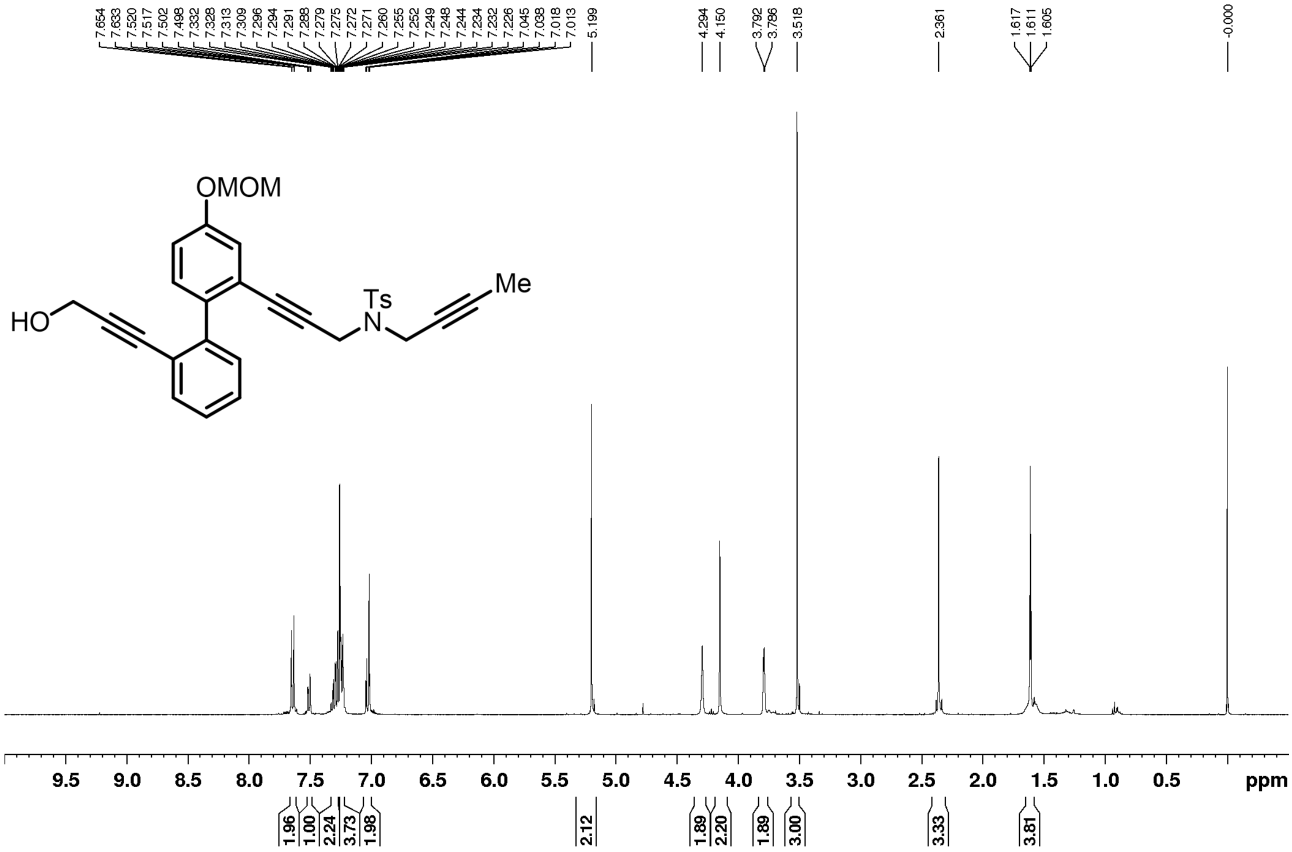

^{13}C NMR ($CDCl_3$, 100 MHz)

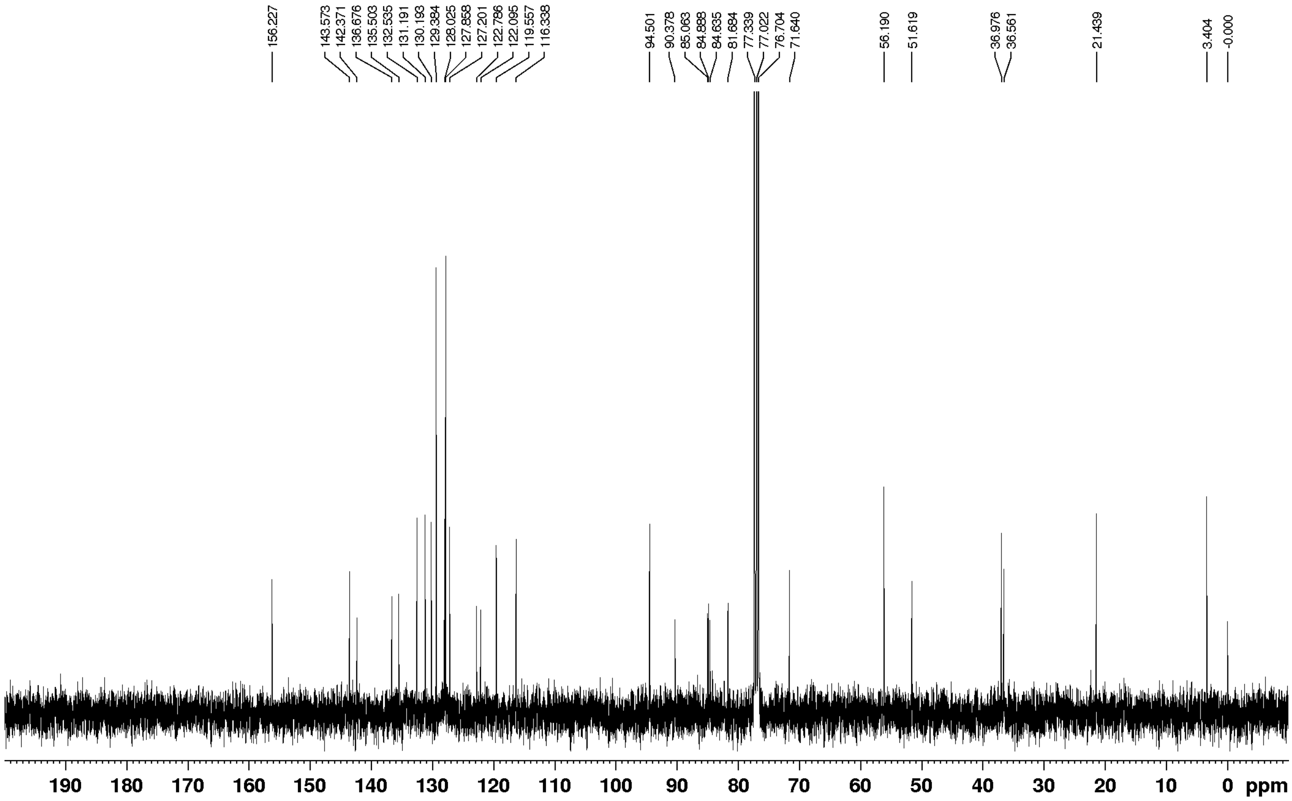

3-(2'-(3-(But-2-yn-1-yloxy)prop-1-yn-1-yl)-4'-(methoxymethoxy)-[1,1'-biphenyl]-2-yl)prop-2-yn-1-ol (9b)

^{1}H NMR ($CDCl_3$, 400 MHz)

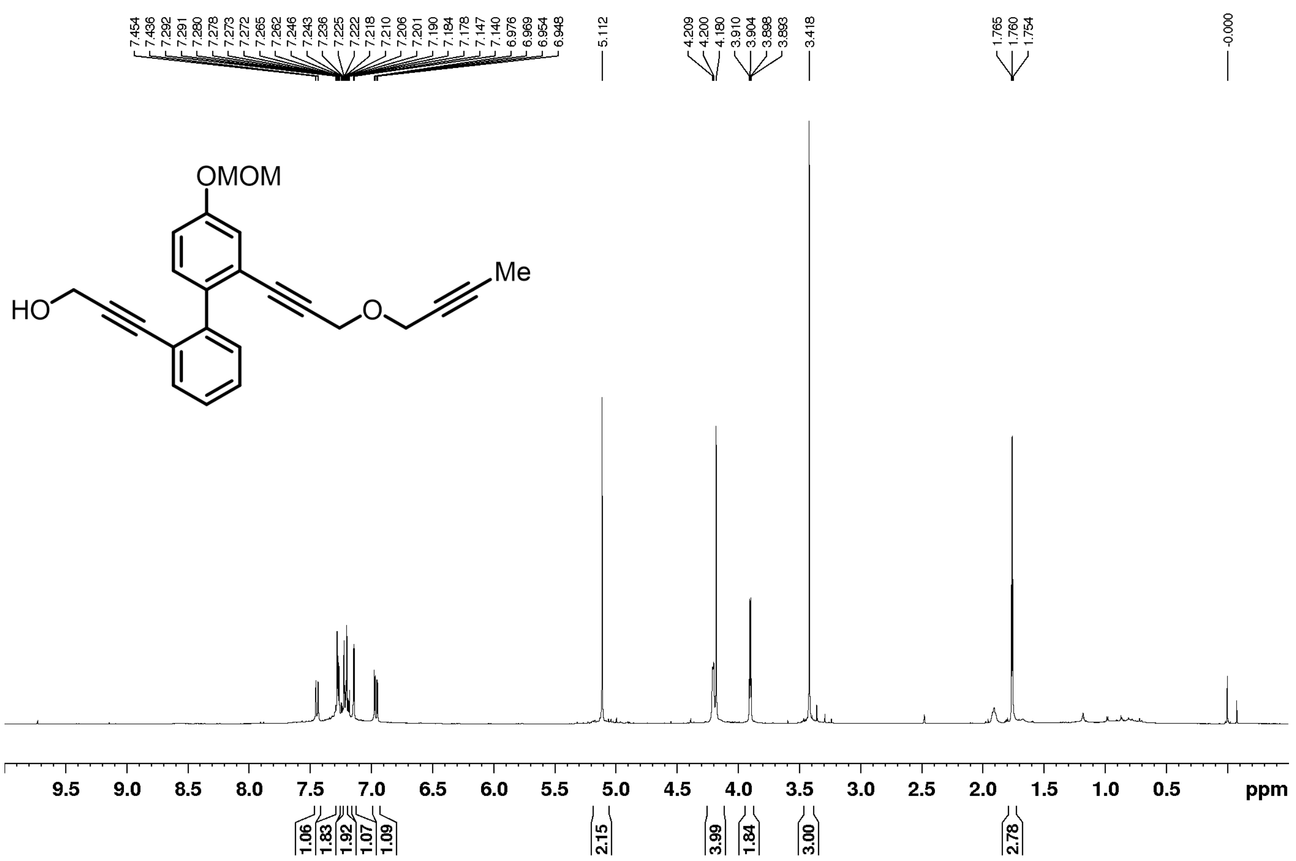

^{13}C NMR ($CDCl_3$, 100 MHz)

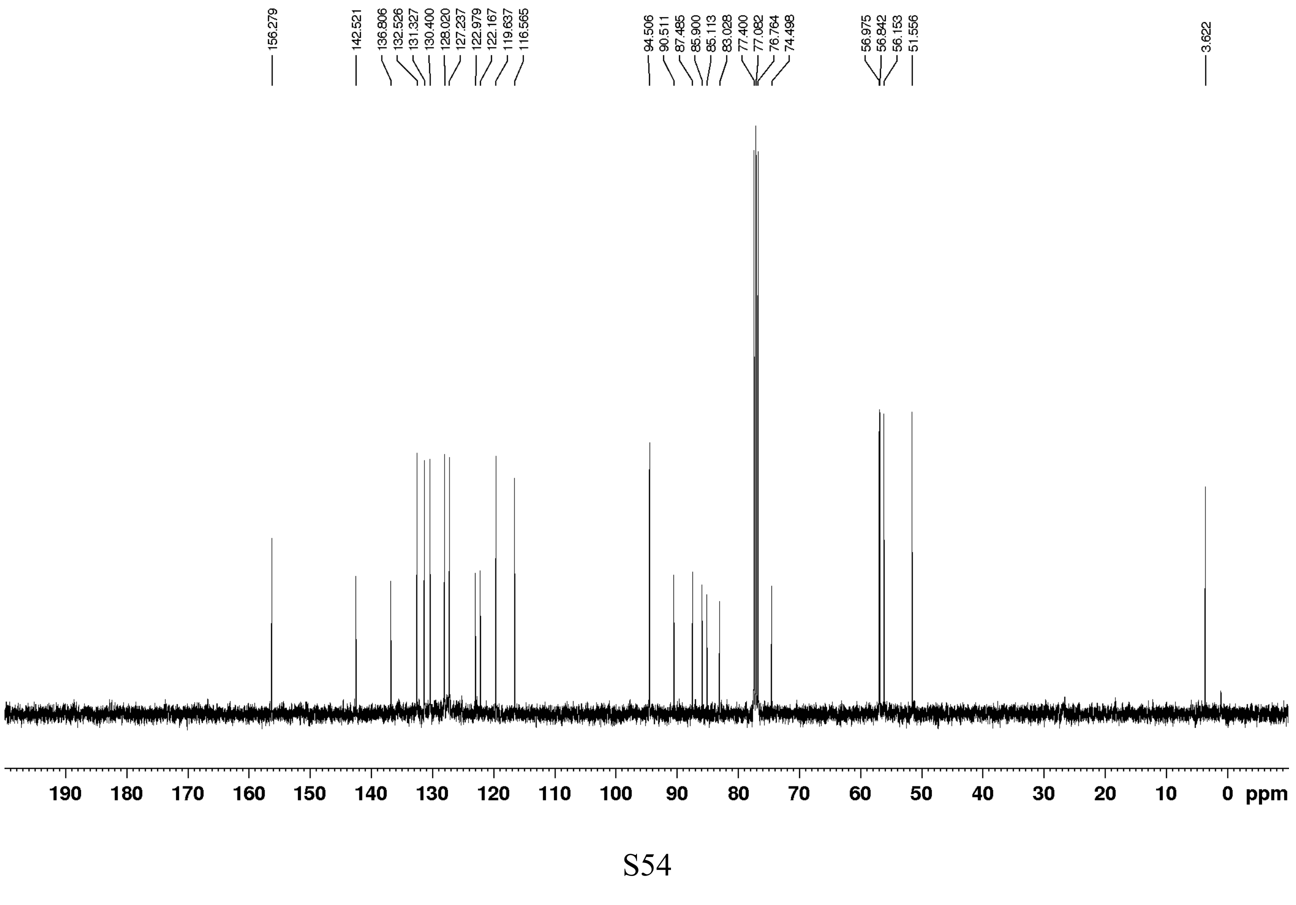

_N_-(Hept-2-yn-1-yl)-_N_-(3-(2'-(3-hydroxyprop-1-yn-1-yl)-4-(methoxymethoxy)-[1,1'-biphenyl]-2-yl)prop-2-yn-1-yl)-4-nitrobenzenesulfonamide (9c)

^{1}H NMR ($CDCl_3$, 400 MHz)

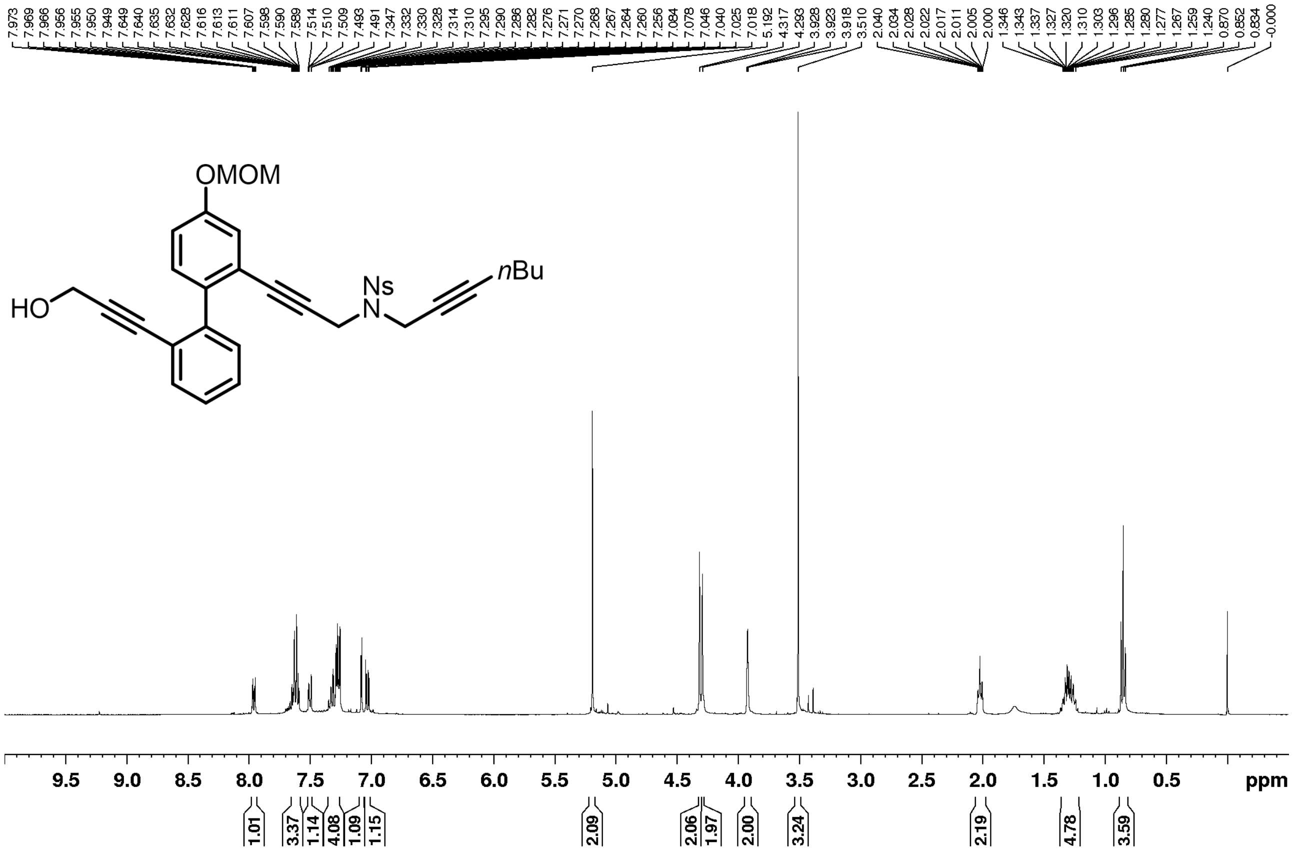

^{13}C NMR ($CDCl_3$, 100 MHz)

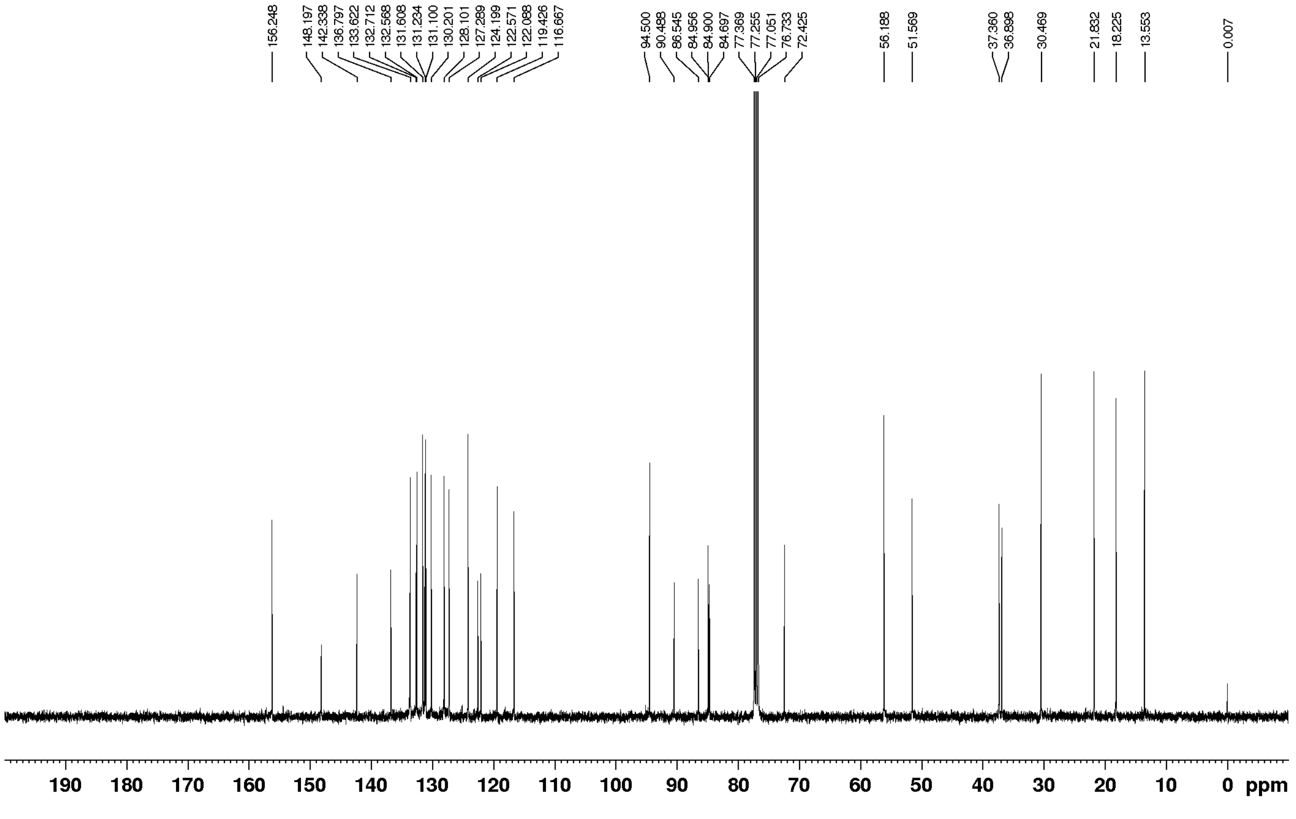

N-(3-(2'-(3-Hydroxyprop-1-yn-1-yl)-4-(methoxymethoxy)-[1,1'-biphenyl]-2-yl)prop-2-yn-1-yl)-4-methyl-N-(3-phenylprop-2-yn-1-yl)benzenesulfonamide (9d)

^{1}H NMR ($CDCl_3$, 400 MHz)

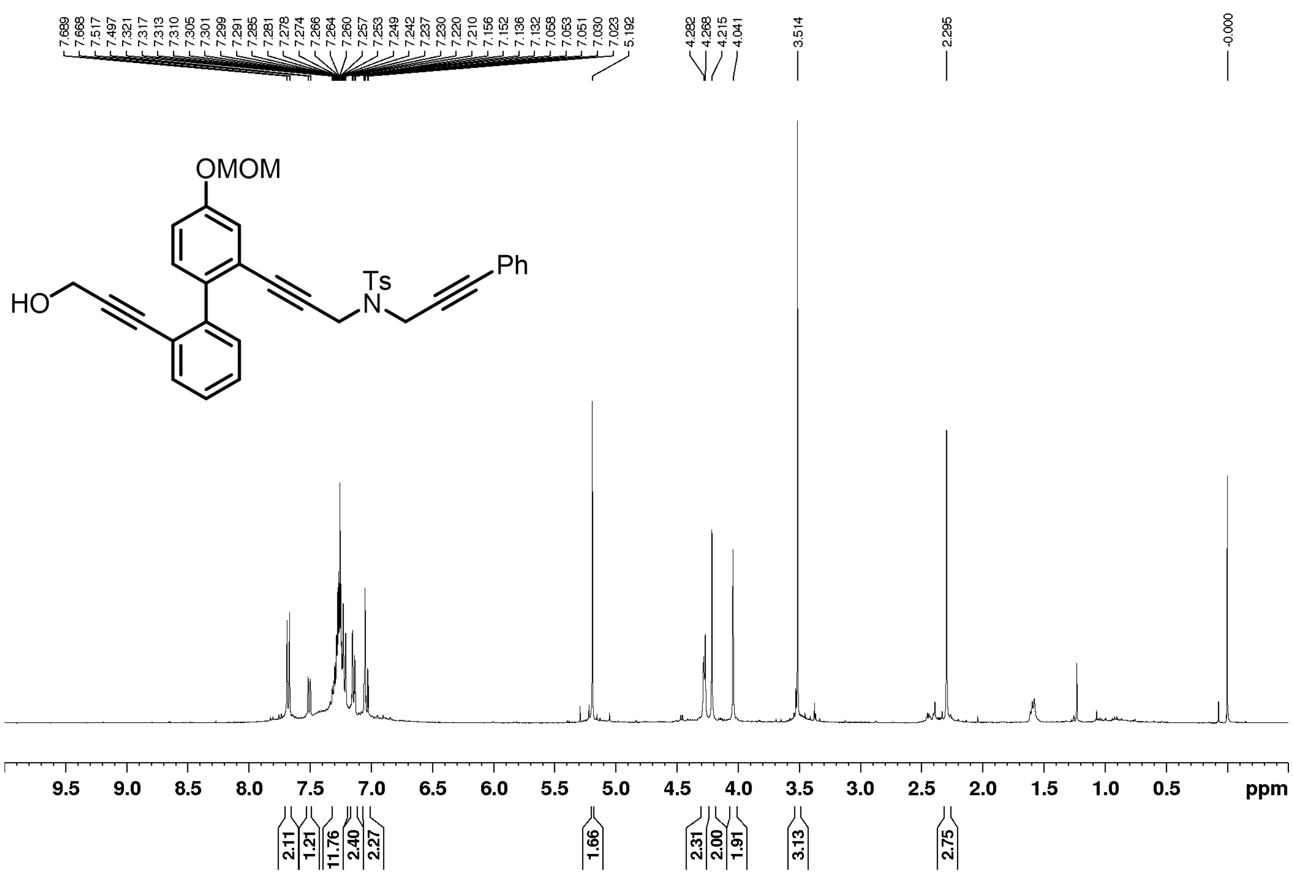

^{13}C NMR ($CDCl_3$, 100 MHz)

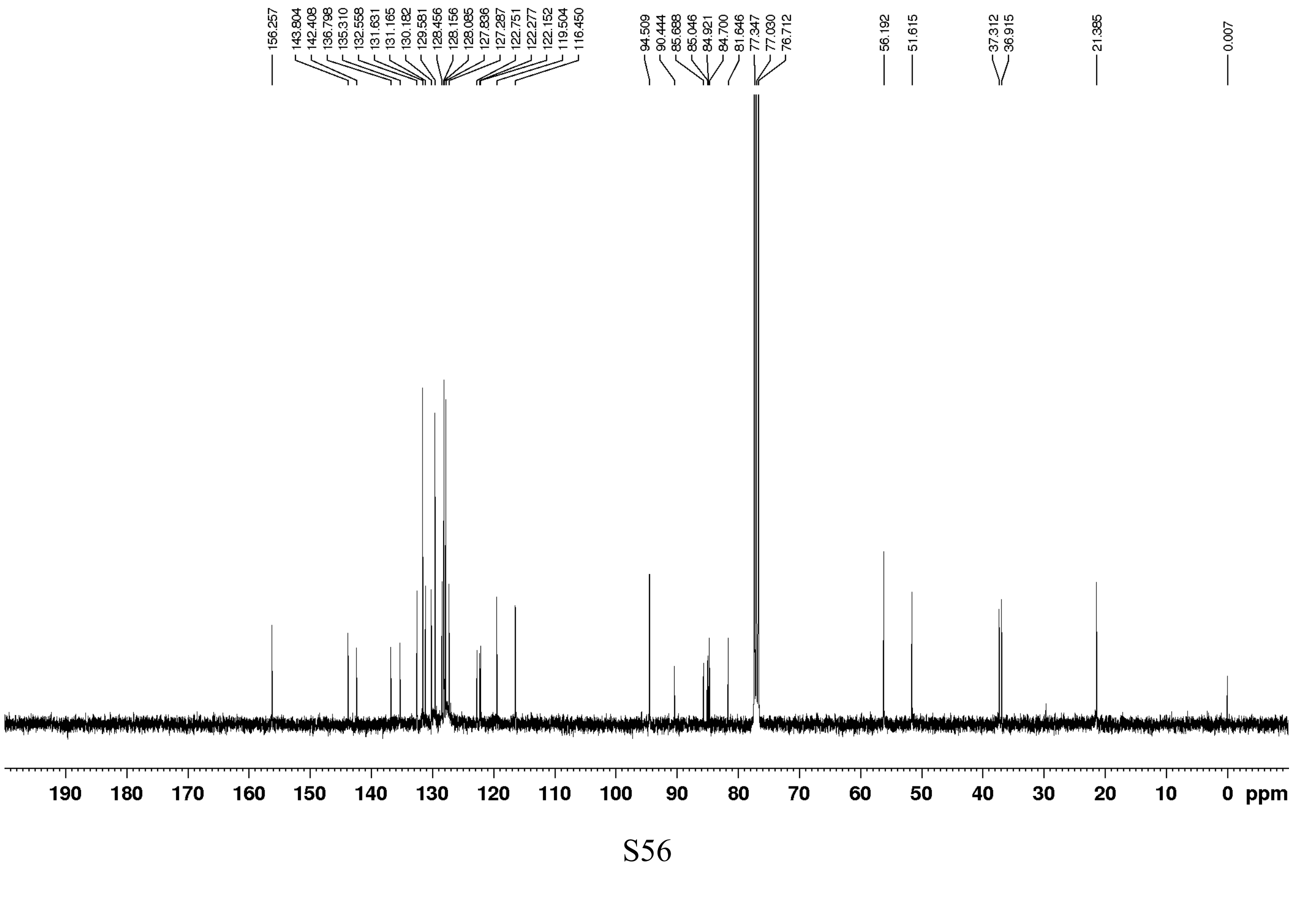

3-(2'-((2'-(Hex-1-yn-1-yl)-[1,1'-biphenyl]-2-yl)ethynyl)-4'-(methoxymethoxy)-[1,1'-biphenyl]-2-yl)prop-2-yn-1-ol (9e)

^{1}H NMR ($CDCl_3$, 400 MHz)

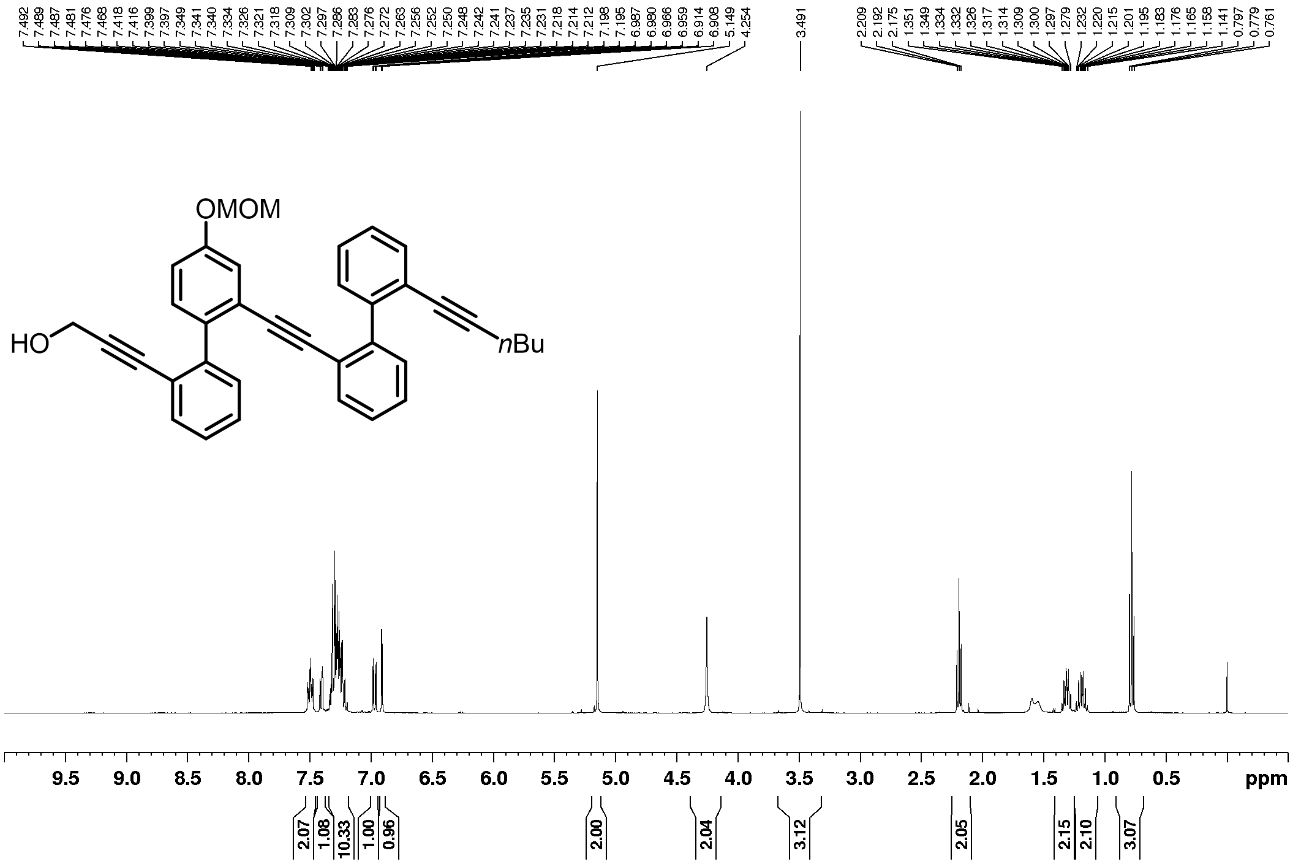

^{13}C NMR ($CDCl_3$, 100 MHz)

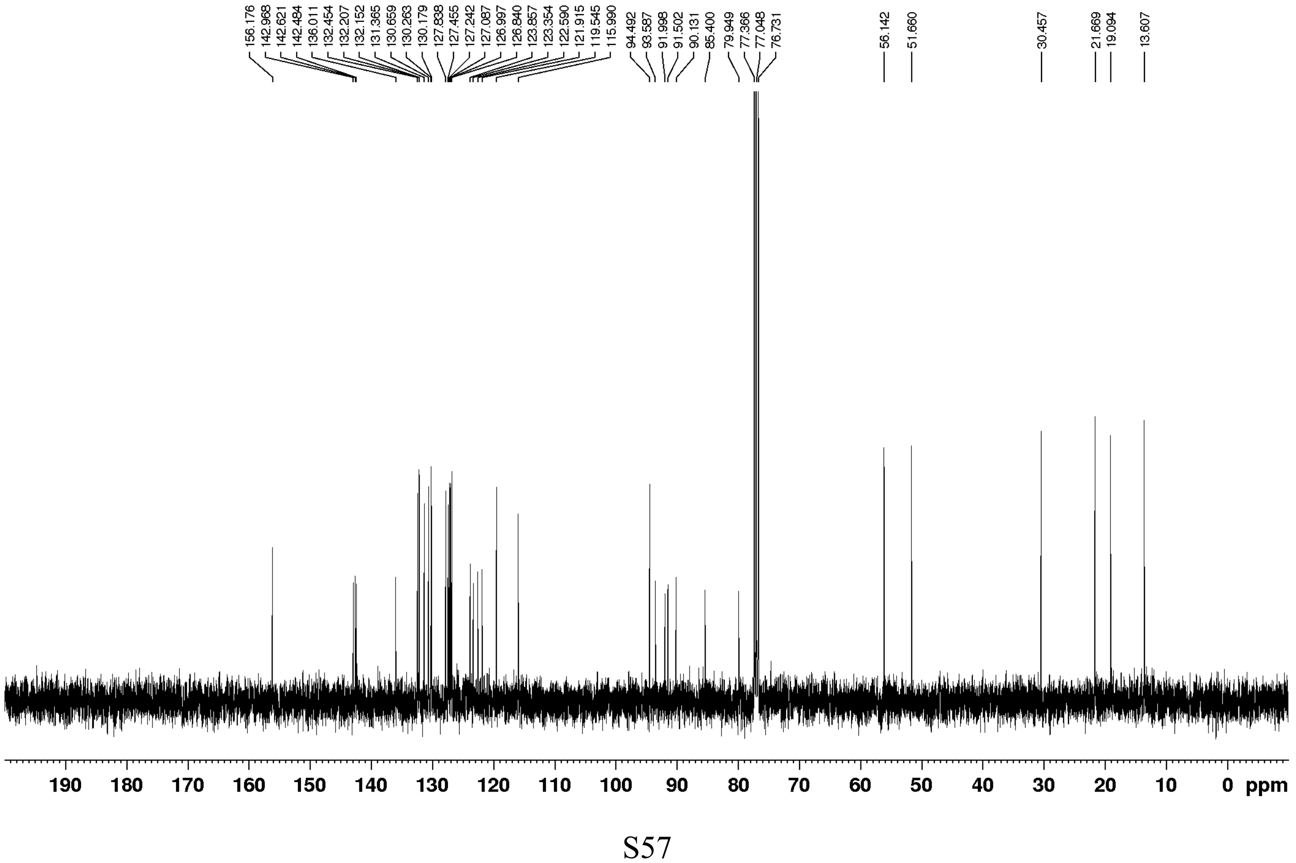

N-(3-(2'-(3-Bromoprop-1-yn-1-yl)-4-hydroxy-[1,1'-biphenyl]-2-yl)prop-2-yn-1-yl)-N-(but-2-yn-1-yl)-4-methylbenzenesulfonamide (1a)

^{1}H NMR ($CDCl_3$, 400 MHz)

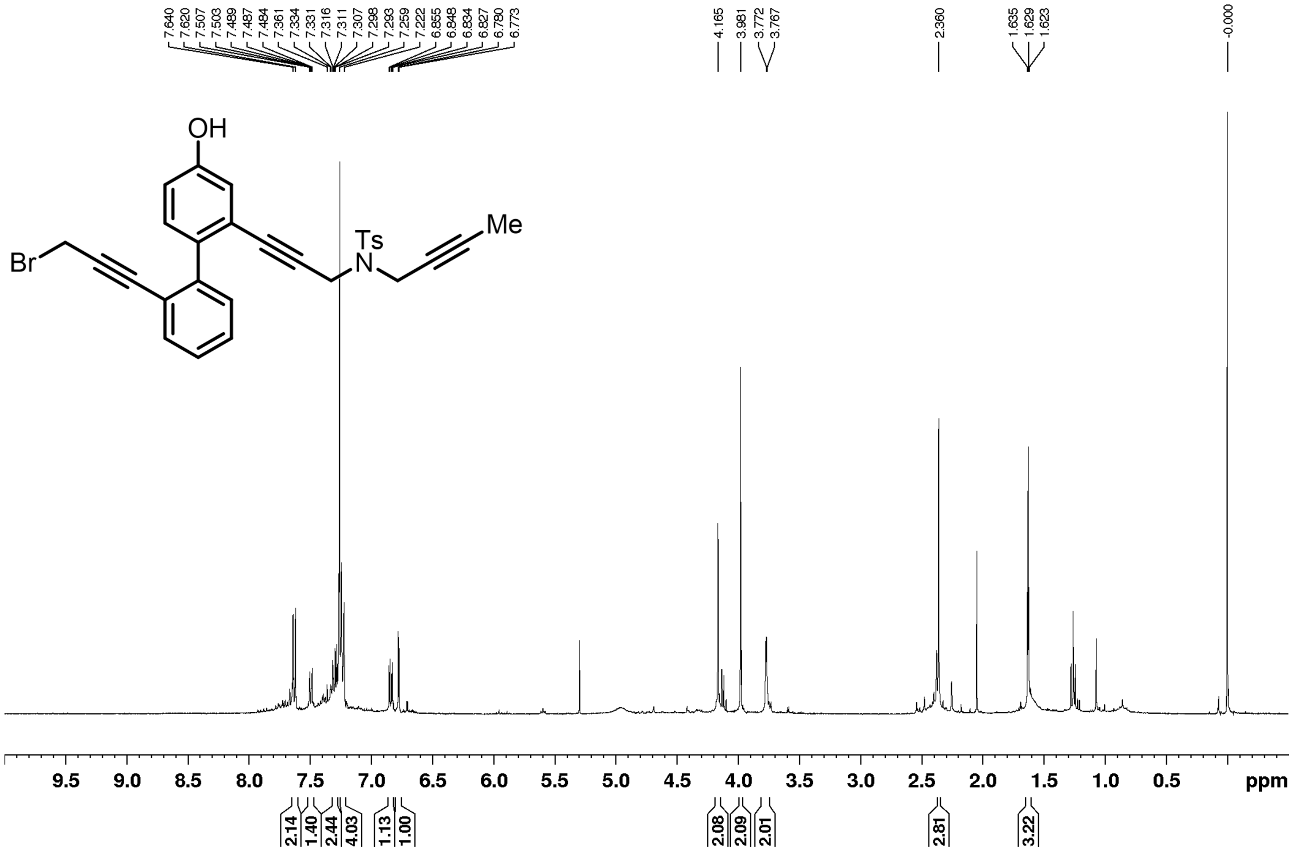

^{13}C NMR ($CDCl_3$, 100 MHz)

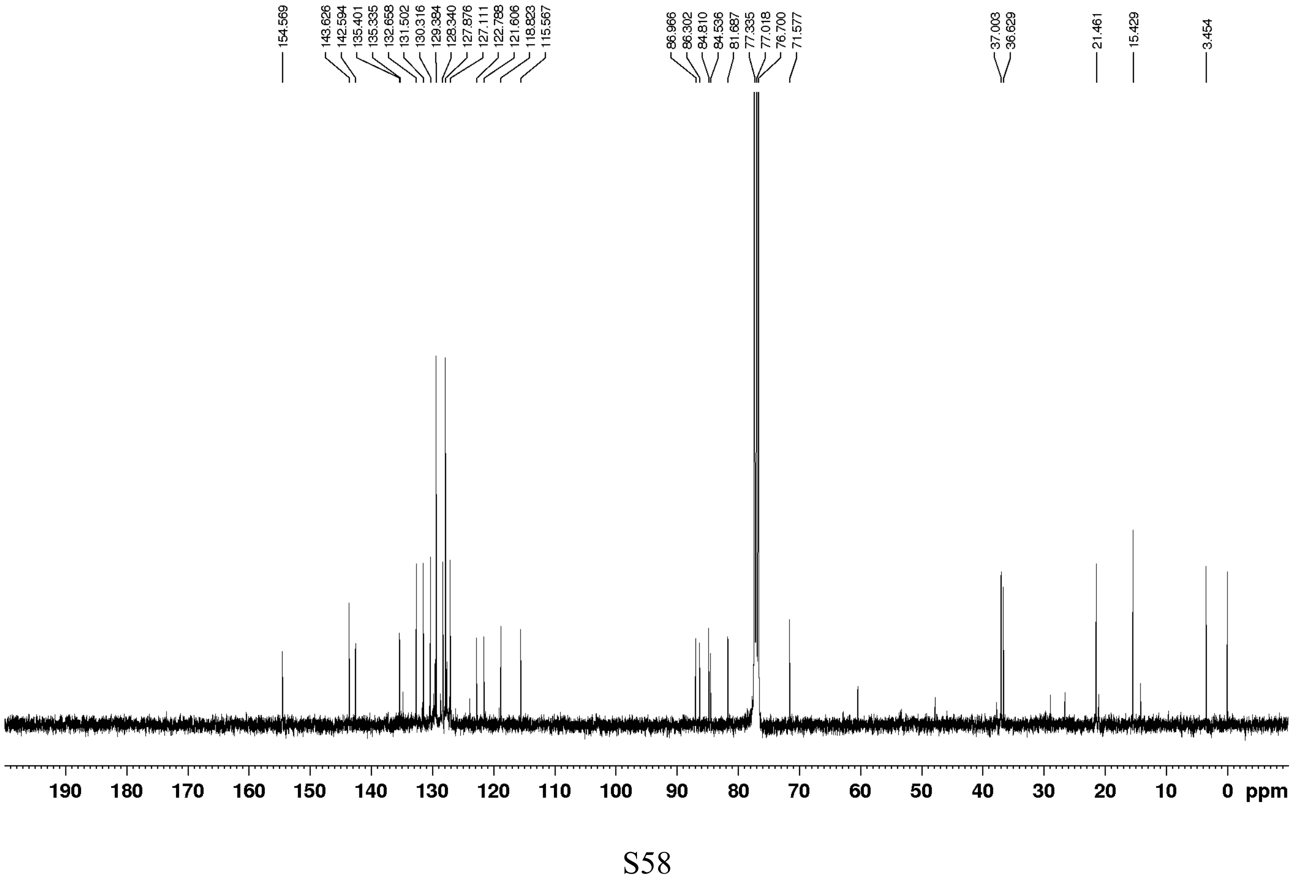

2'-(3-Bromoprop-1-yn-1-yl)-2-(3-(but-2-yn-1-yloxy)prop-1-yn-1-yl)-[1,1'-biphenyl]-4-ol (1b)

^{1}H NMR ($CDCl_3$, 400 MHz)

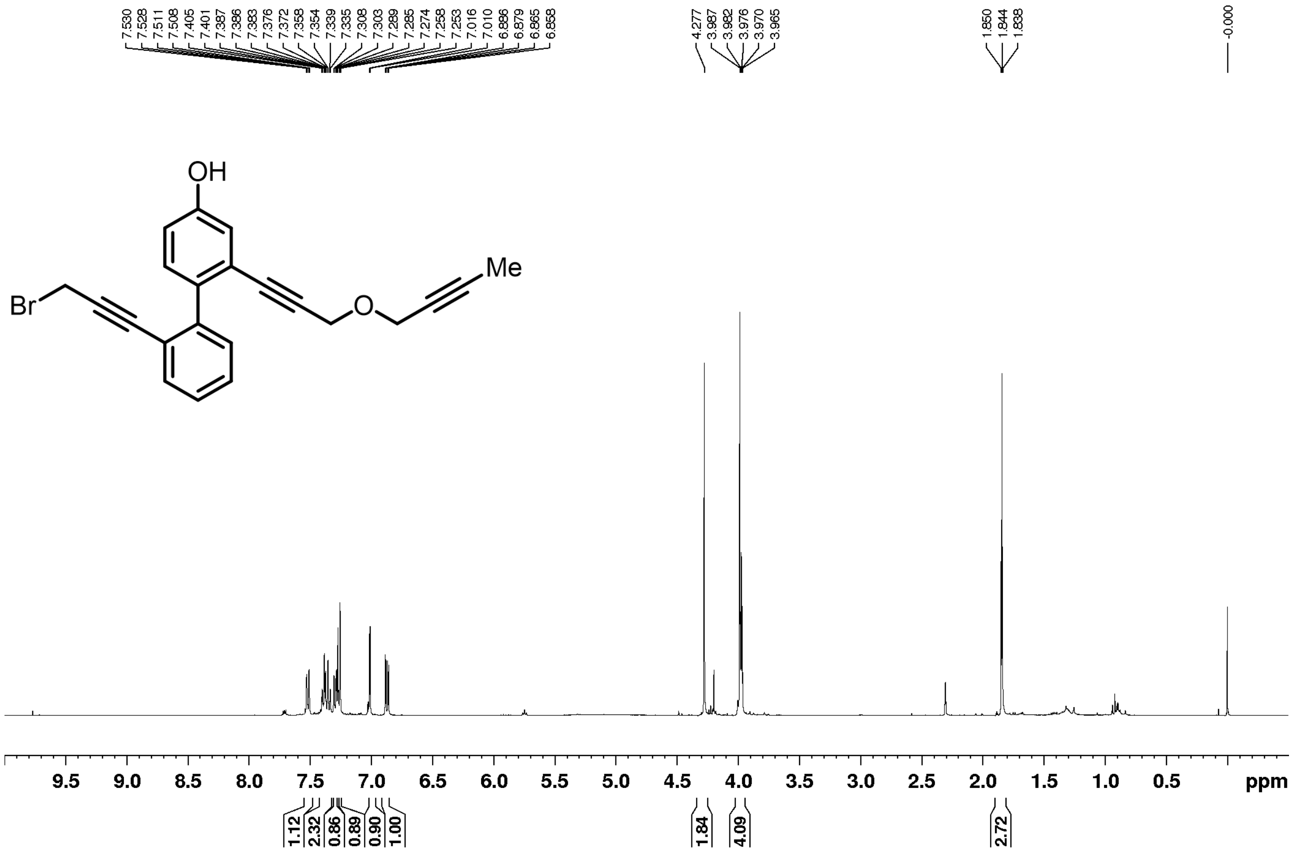

^{13}C NMR ($CDCl_3$, 100 MHz)

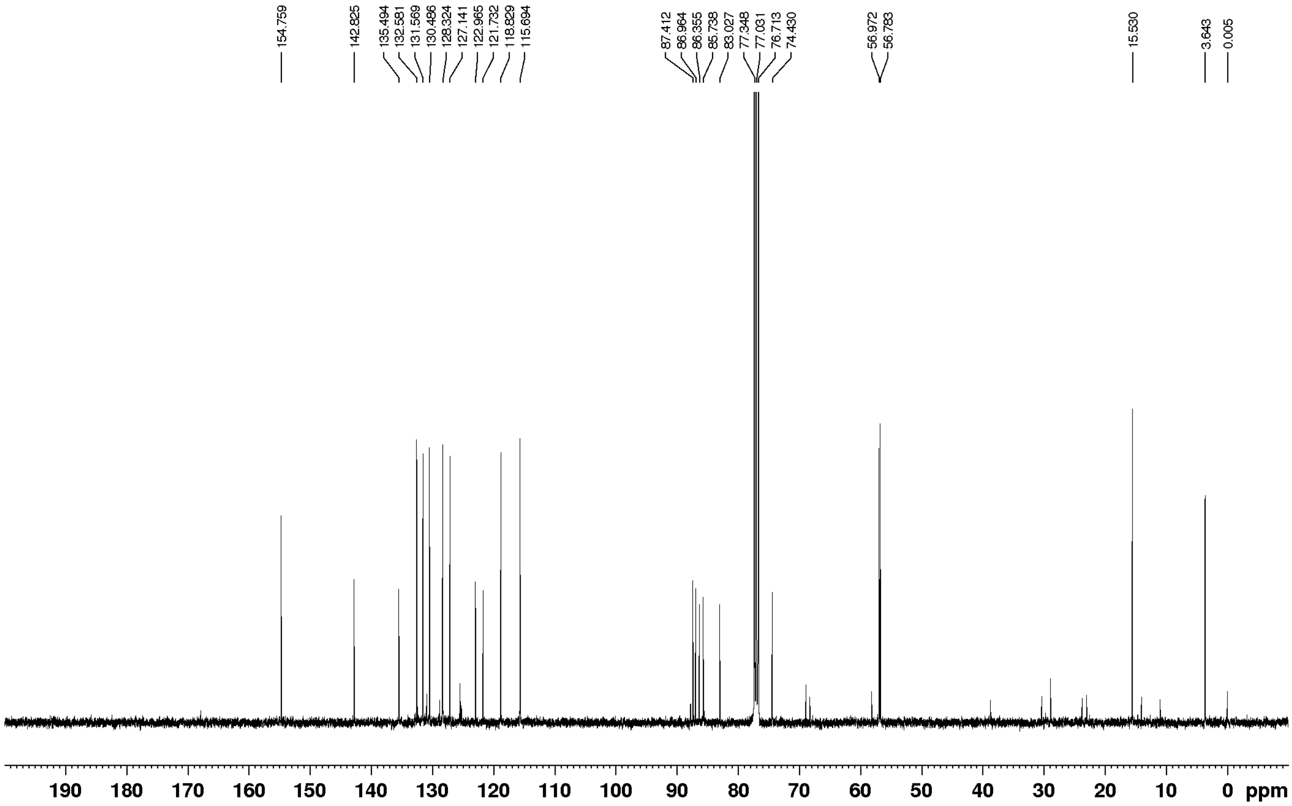

N-(3-(2'-(3-Bromoprop-1-yn-1-yl)-4-hydroxy-[1,1'-biphenyl]-2-yl)prop-2-yn-1-yl)-N-(hept-2-yn-1-yl)-4-nitrobenzenesulfonamide (1c)

^1H NMR ($CDCl_3$, 400 MHz)

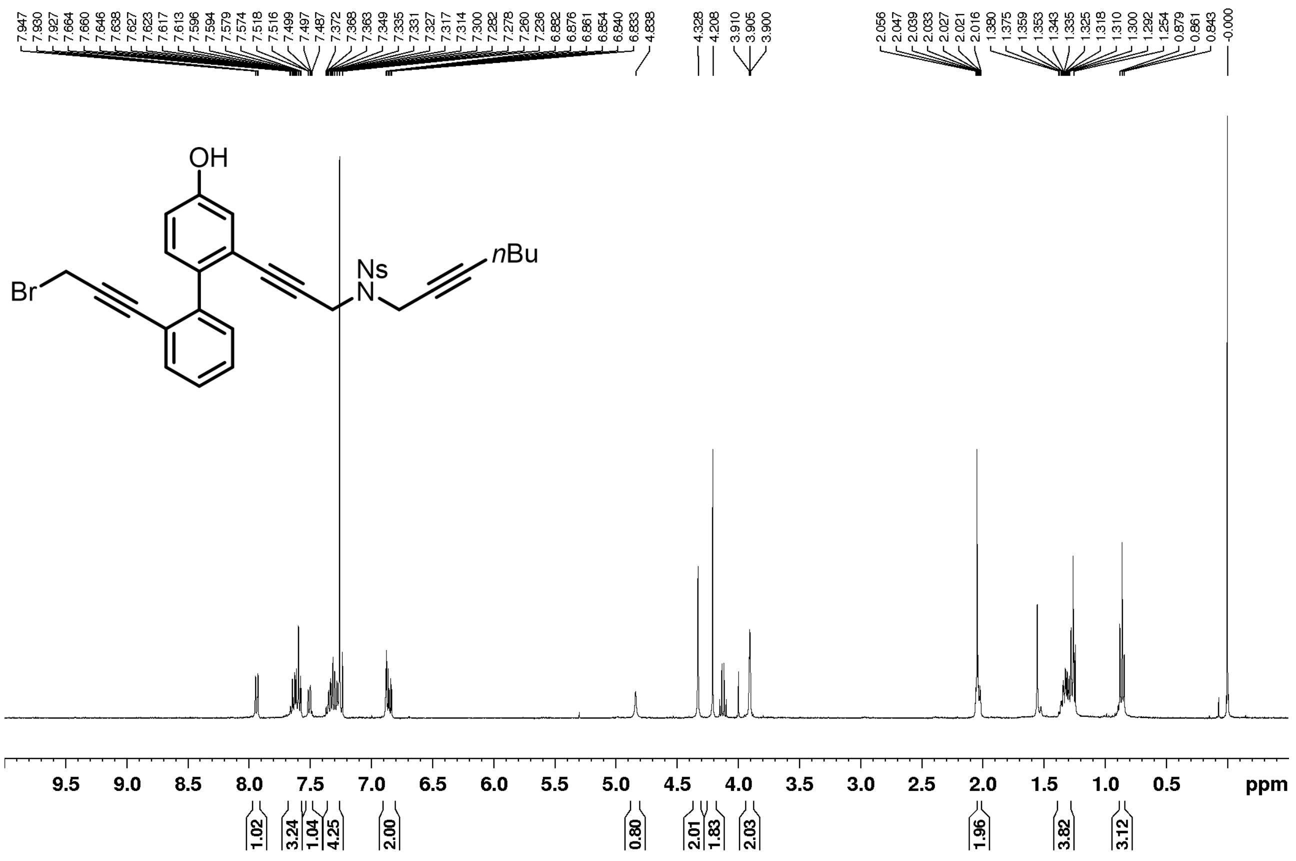

^{13}C NMR ($CDCl_3$, 100 MHz)

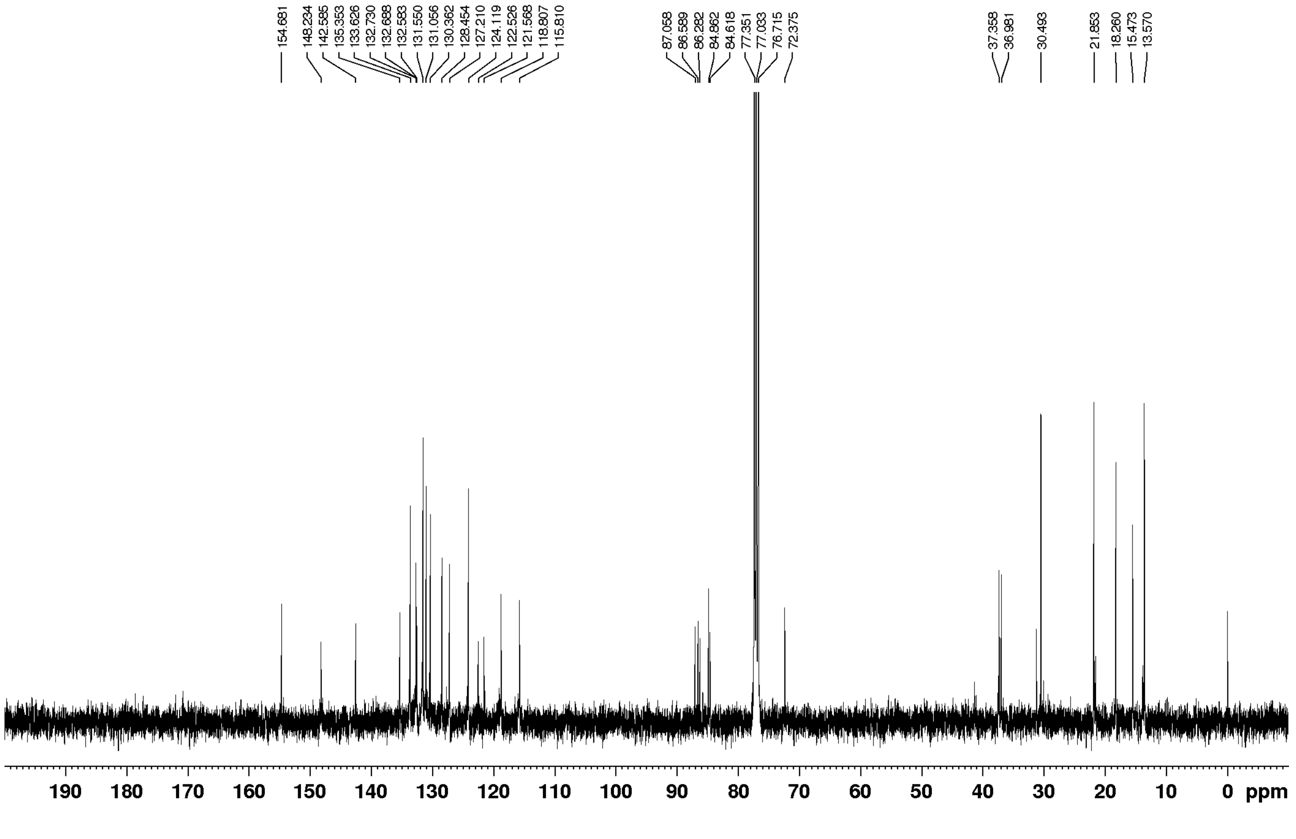

N-(3-(2'-(3-Bromoprop-1-yn-1-yl)-4-hydroxy-[1,1'-biphenyl]-2-yl)prop-2-yn-1-yl)-4-methyl-N-(3-phenylprop-2-yn-1-yl)benzenesulfonamide (1d)

^{1}H NMR ($CDCl_3$, 400 MHz)

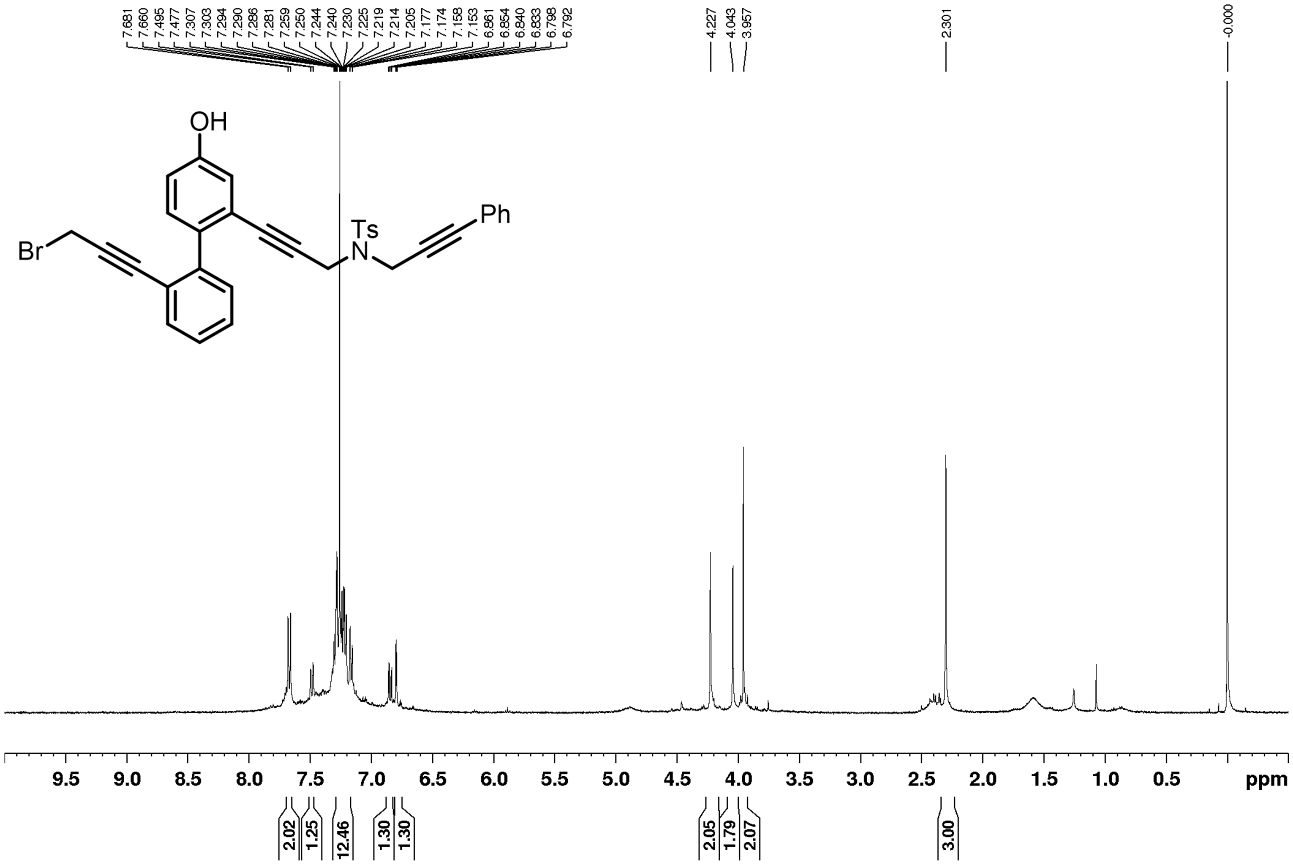

^{13}C NMR ($CDCl_3$, 100 MHz)

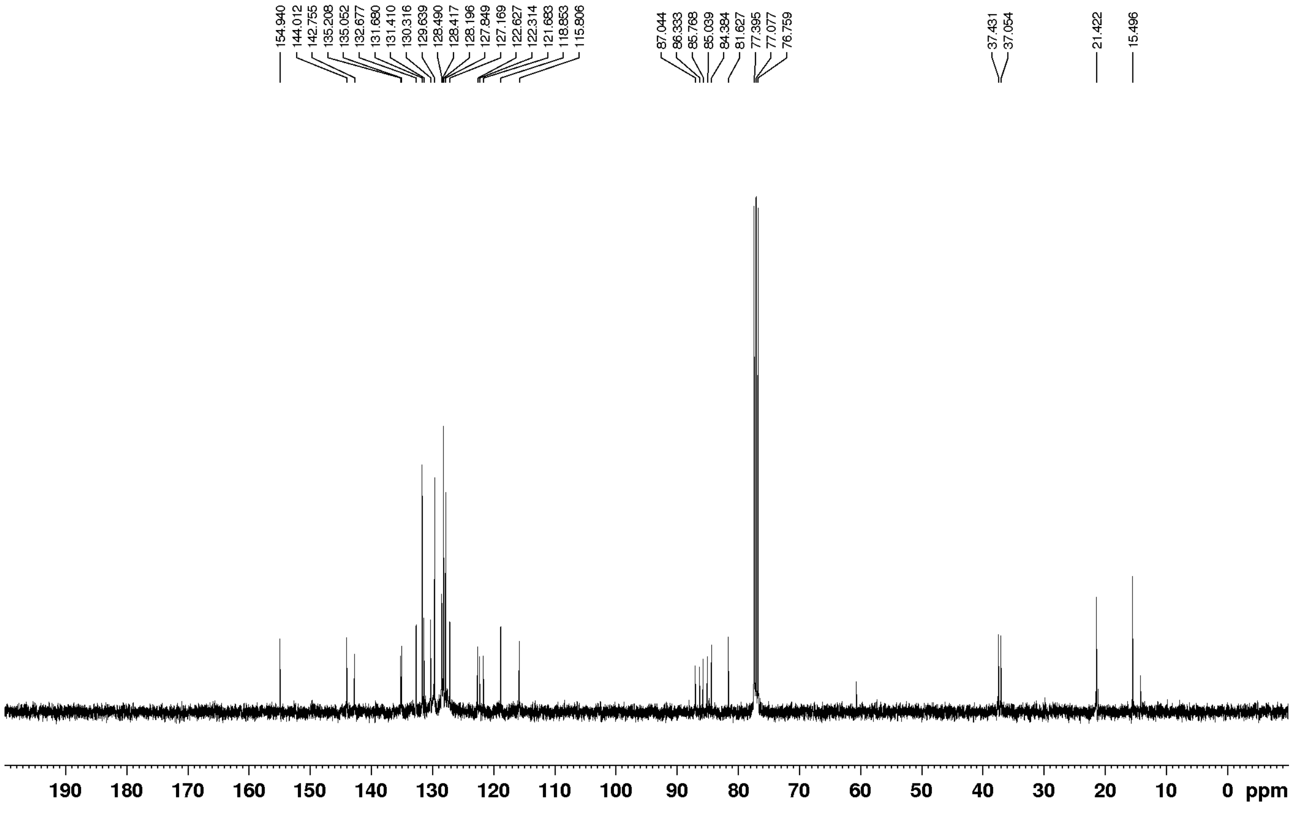

2'-(3-Bromoprop-1-yn-1-yl)-2-((2'-(hex-1-yn-1-yl)-[1,1'-biphenyl]-2-yl)ethynyl)-[1,1'-biphenyl]-4-ol (1e)

^{1}H NMR ($CDCl_3$, 400 MHz)

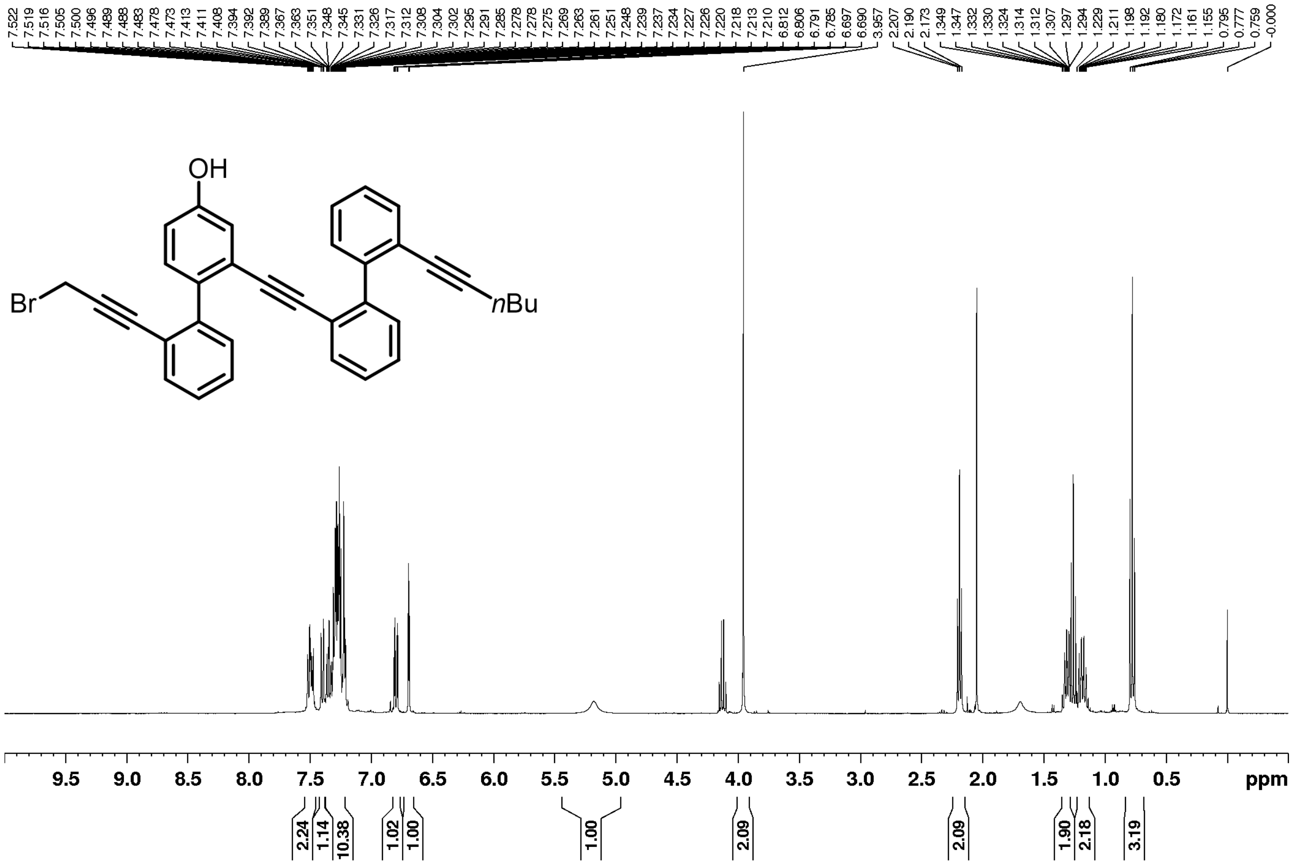

^{13}C NMR ($CDCl_3$, 100 MHz)

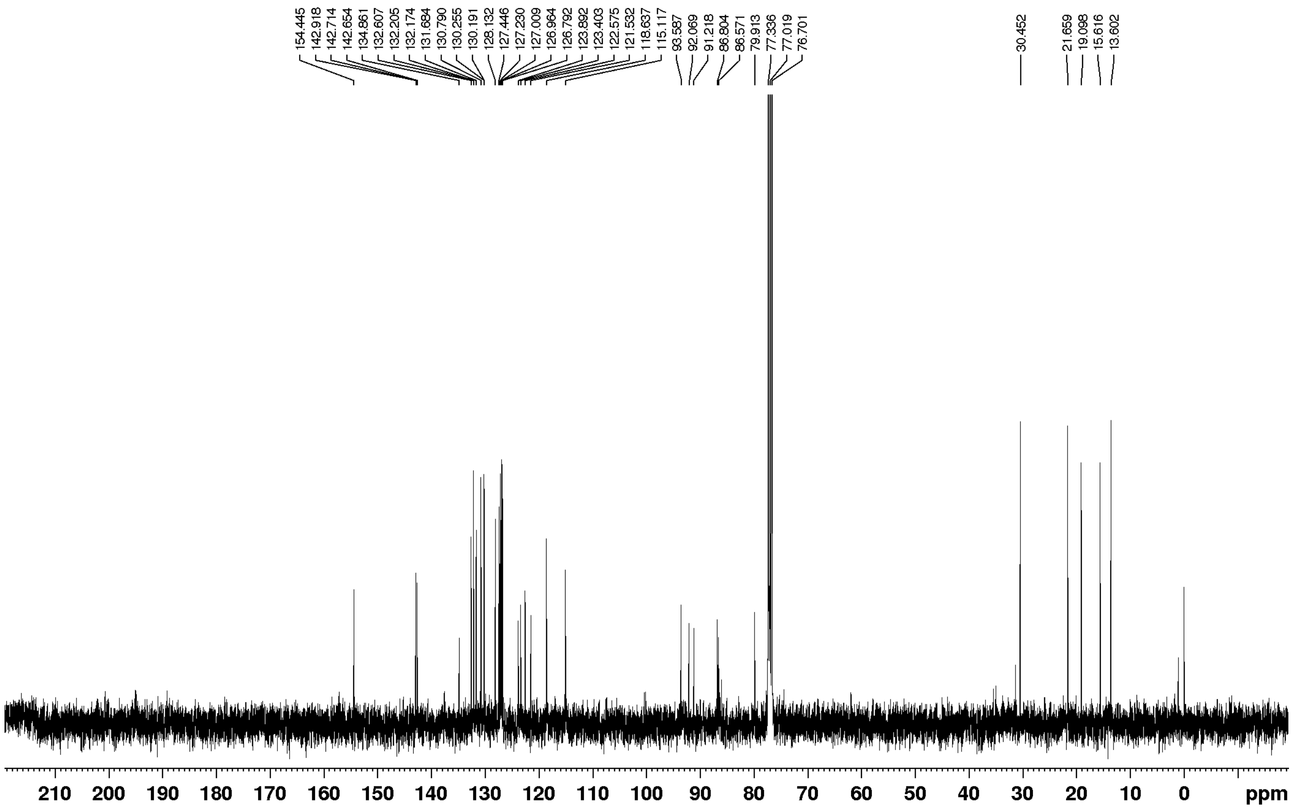

Hexayne 2a

^{1}H NMR ($CDCl_3$, 400 MHz)

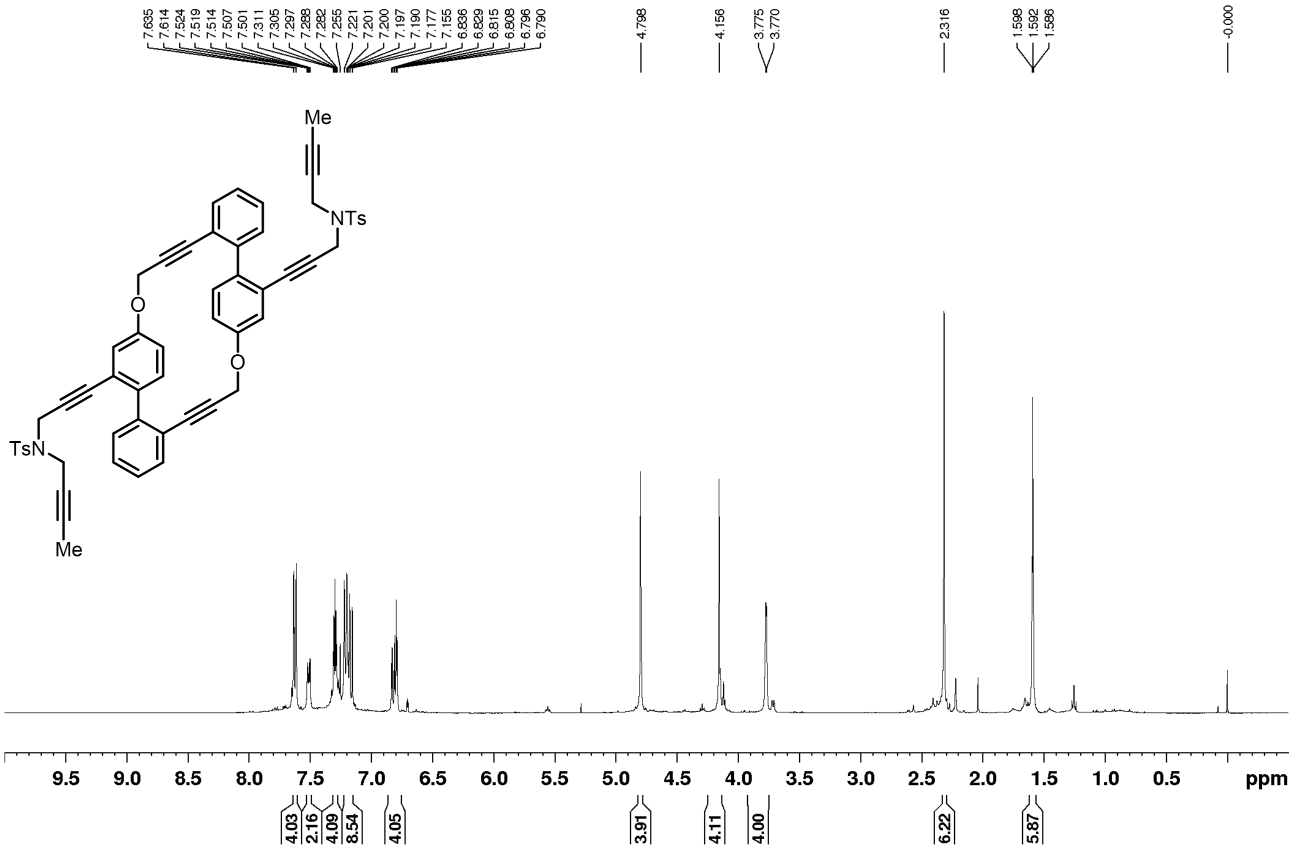

^{13}C NMR ($CDCl_3$, 100 MHz)

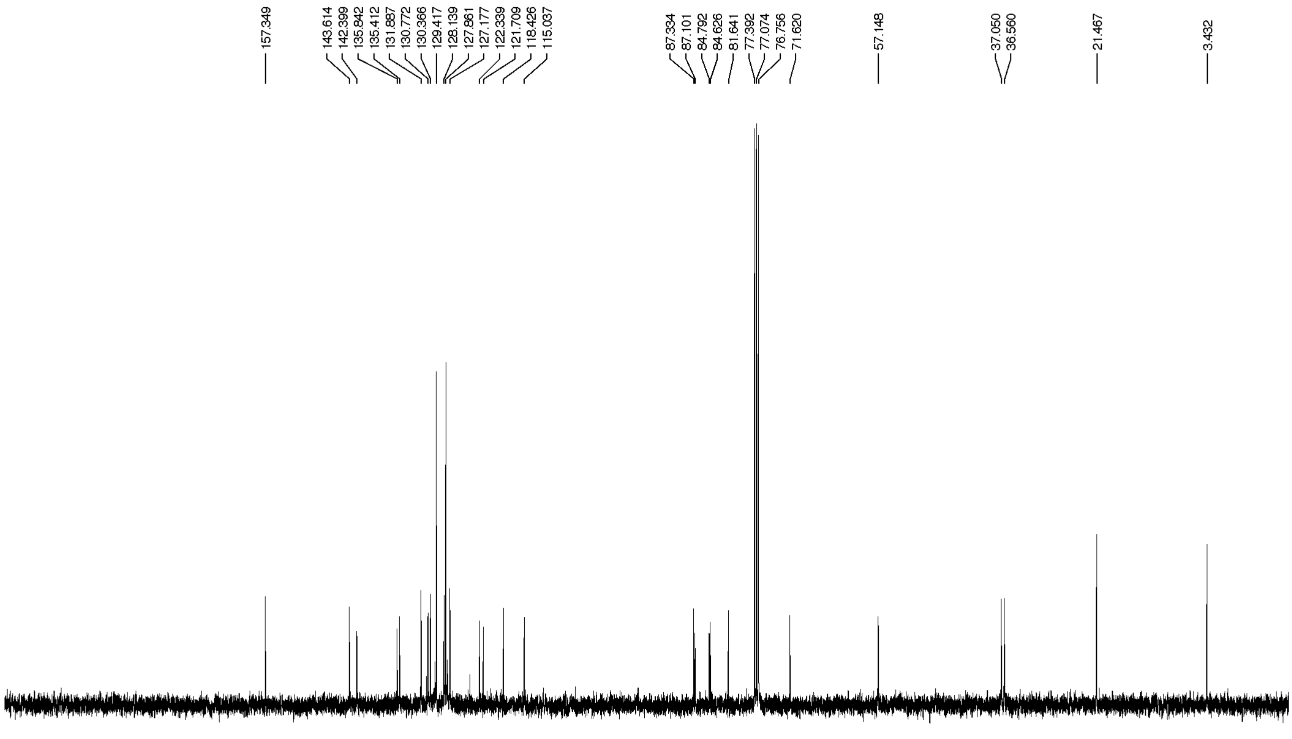

Hexayne 2b

^{1}H NMR ($CDCl_3$, 400 MHz)

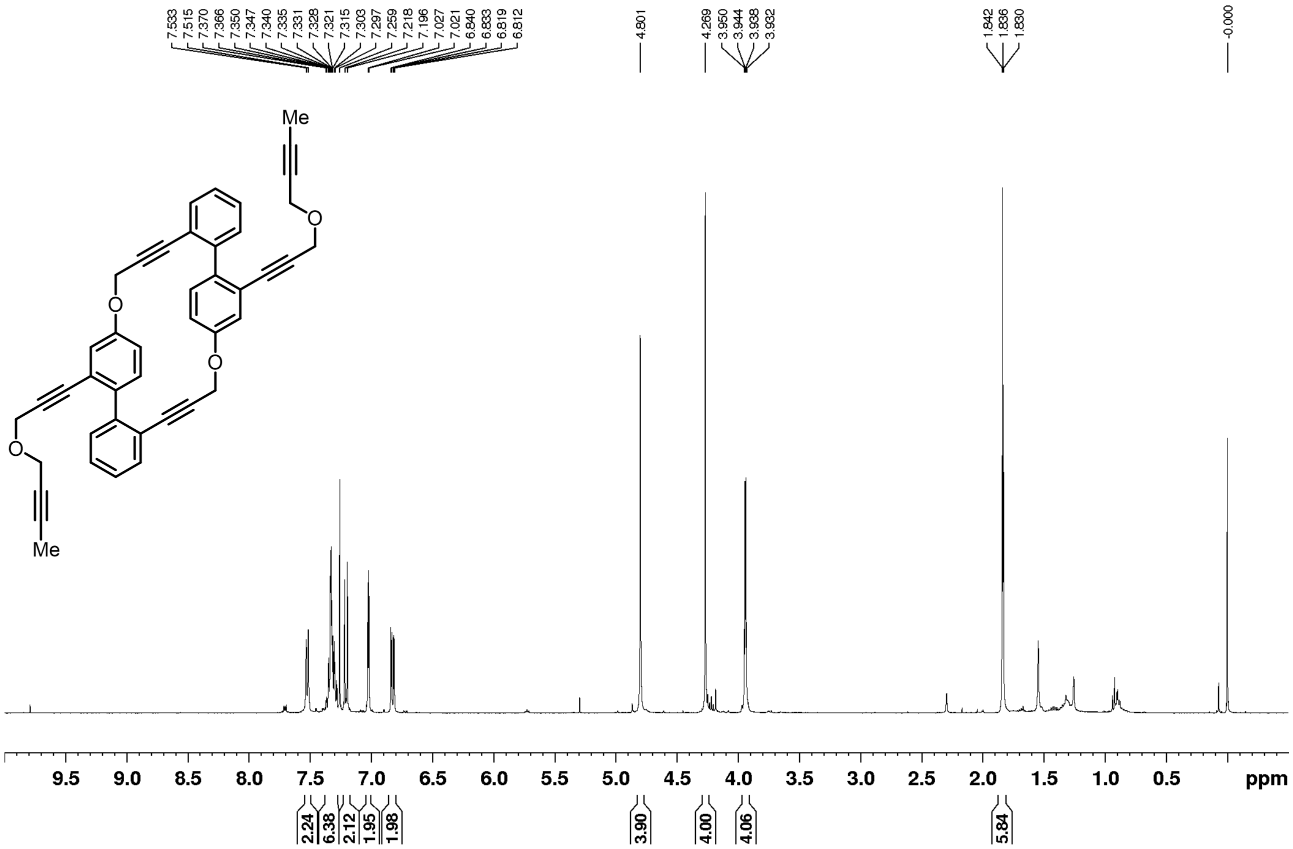

^{13}C NMR ($CDCl_3$, 100 MHz)

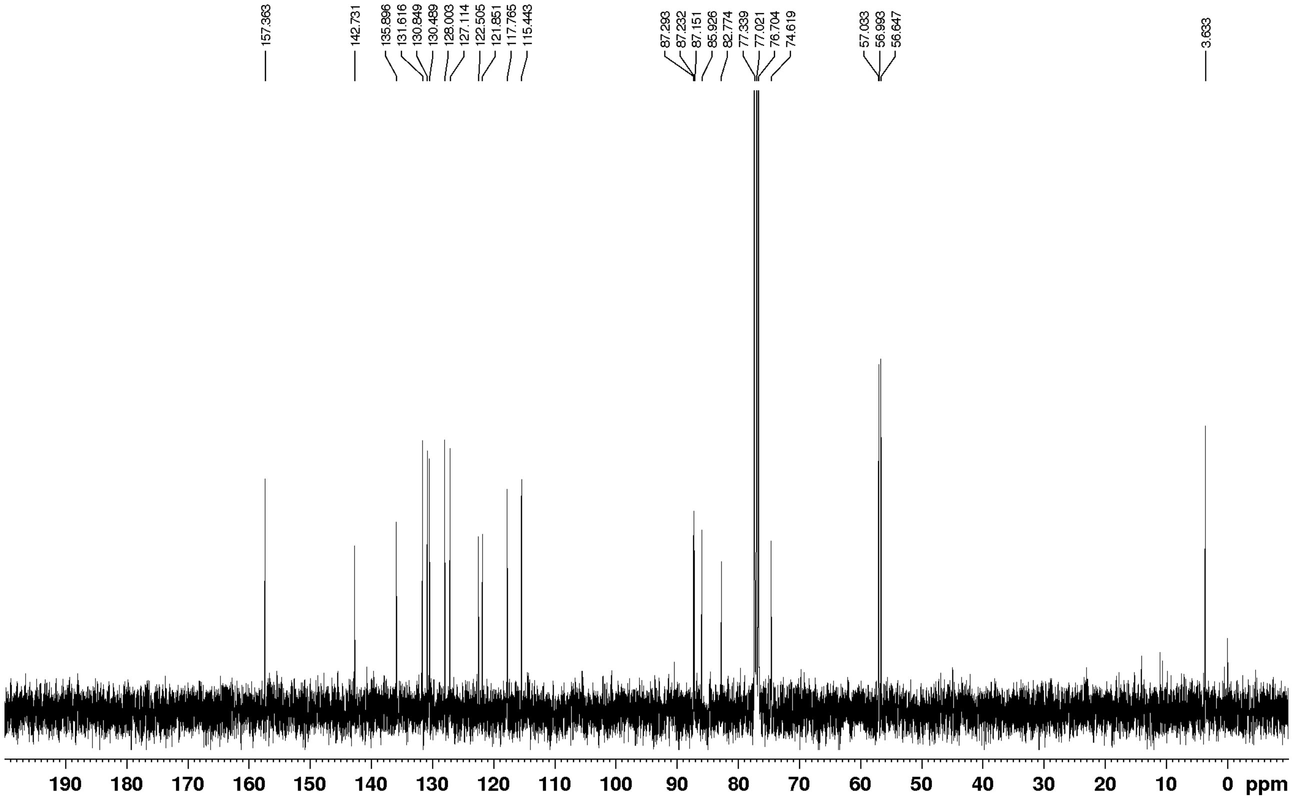

Hexayne 2c

^{1}H NMR ($CDCl_3$, 600 MHz)

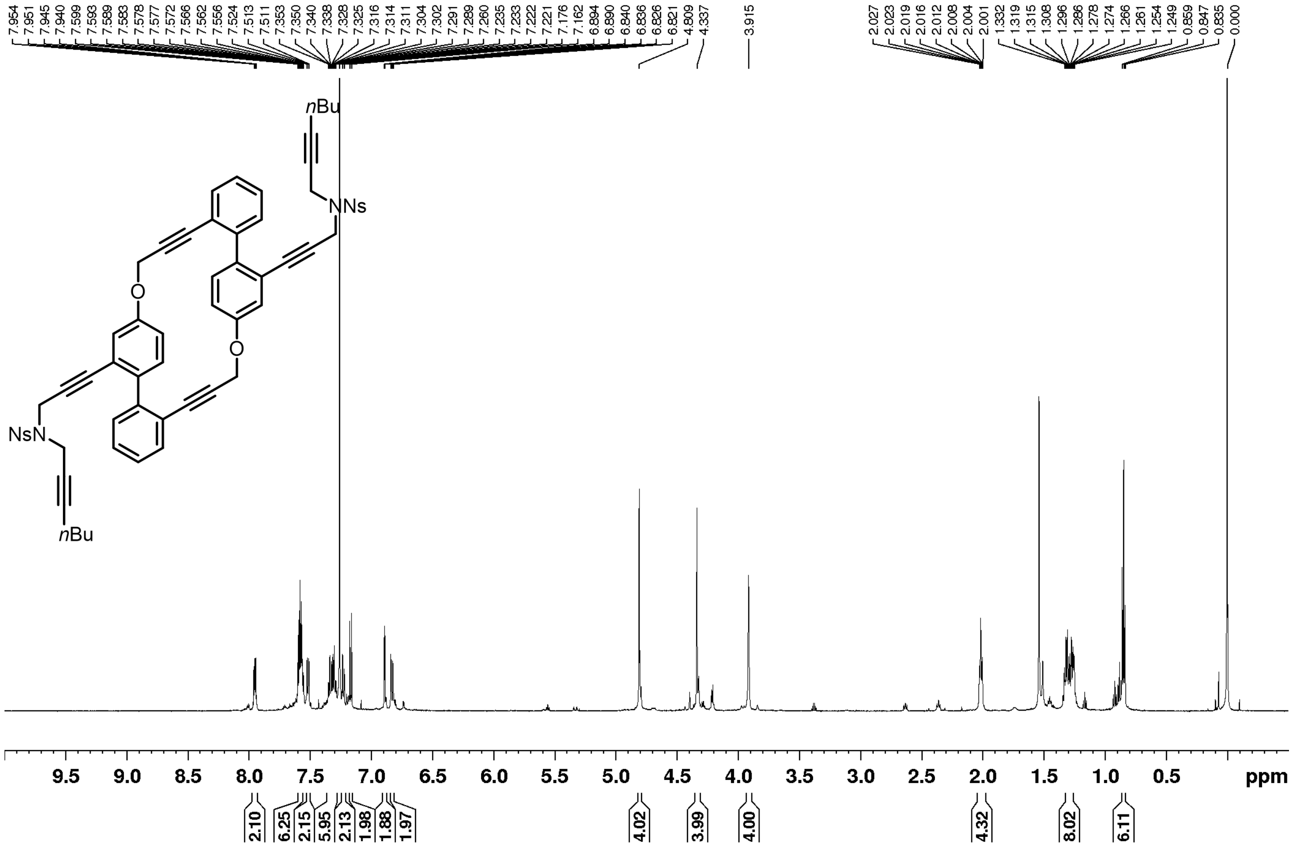

^{13}C NMR ($CDCl_3$, 150 MHz)

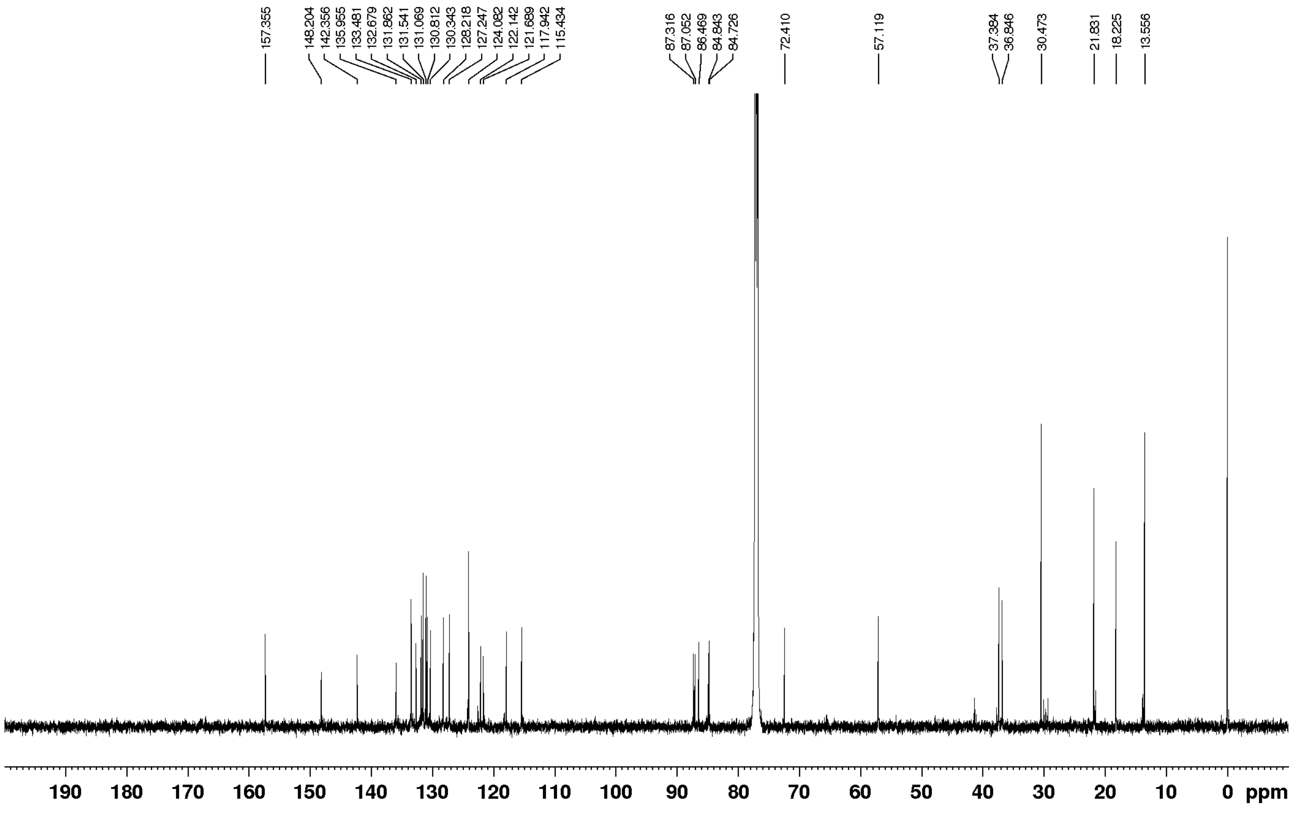

Hexayne 2d

^{1}H NMR ($CDCl_3$, 400 MHz)

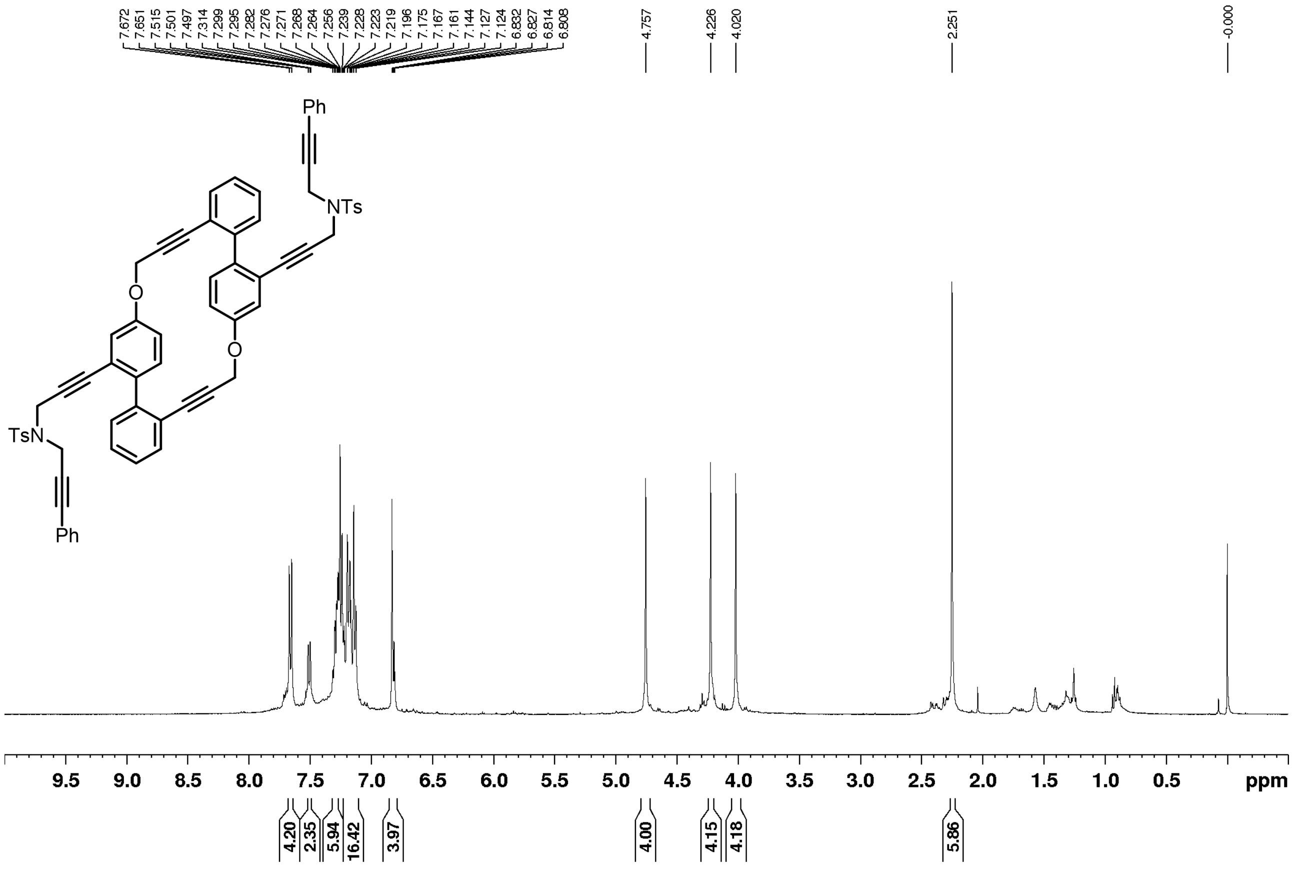

^{13}C NMR ($CDCl_3$, 100 MHz)

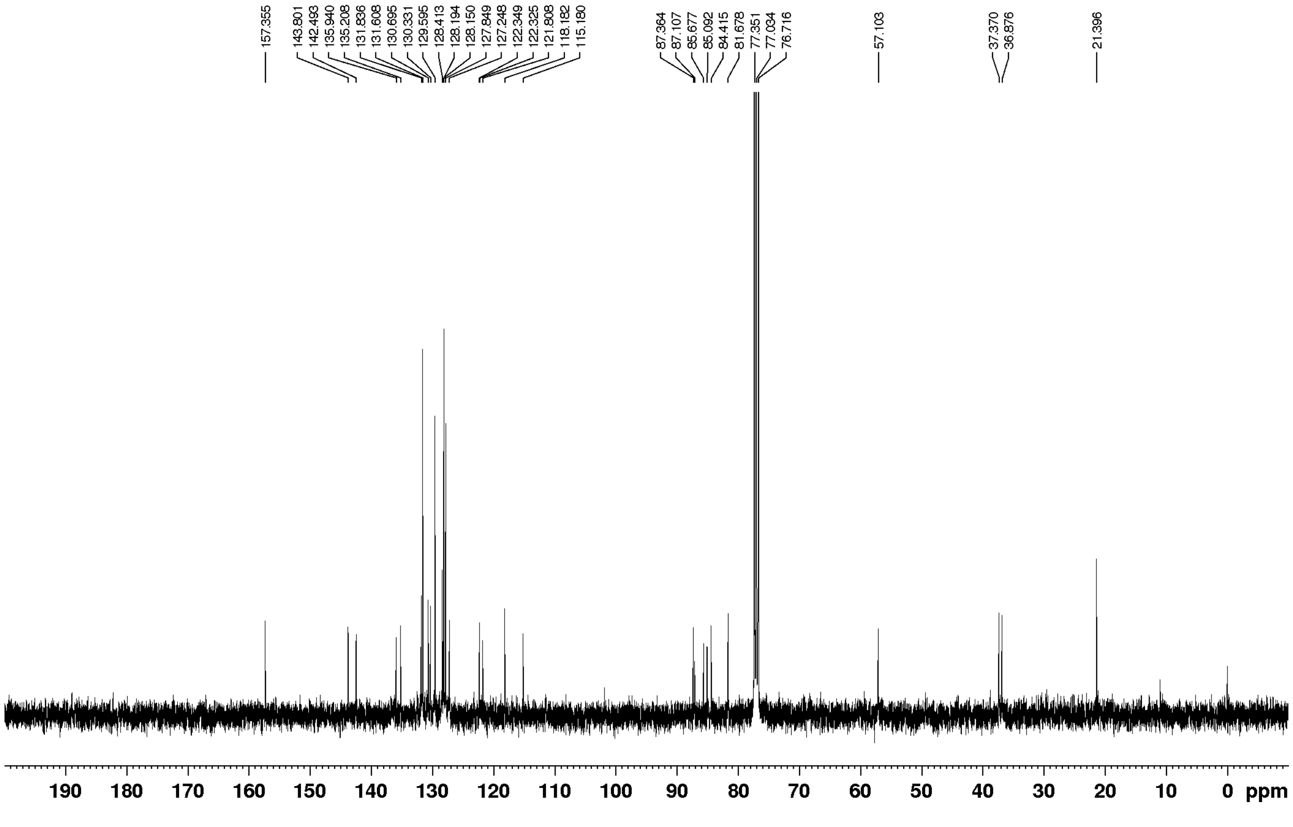

Hexayne 2e

^{1}H NMR ($CDCl_3$, 400 MHz)

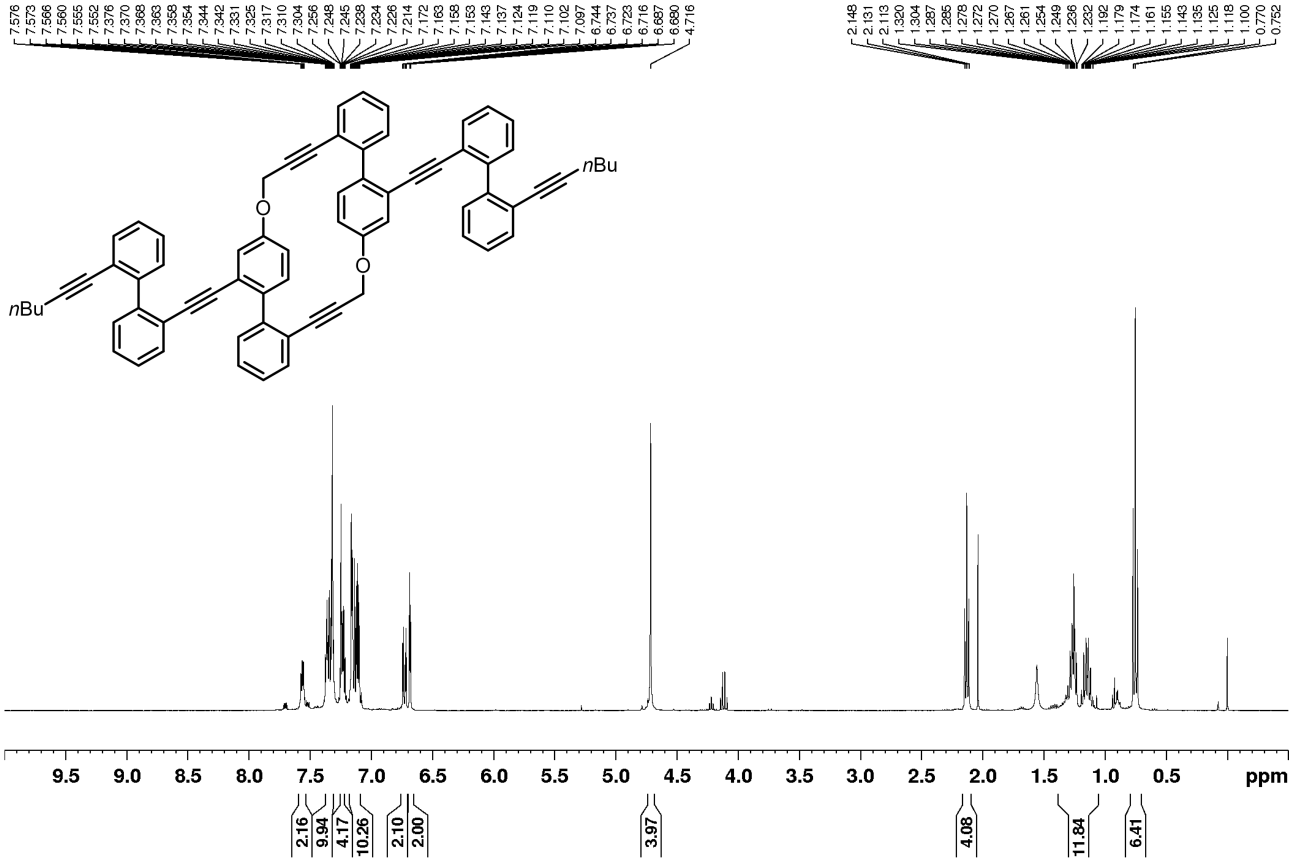

^{13}C NMR ($CDCl_3$, 100 MHz)

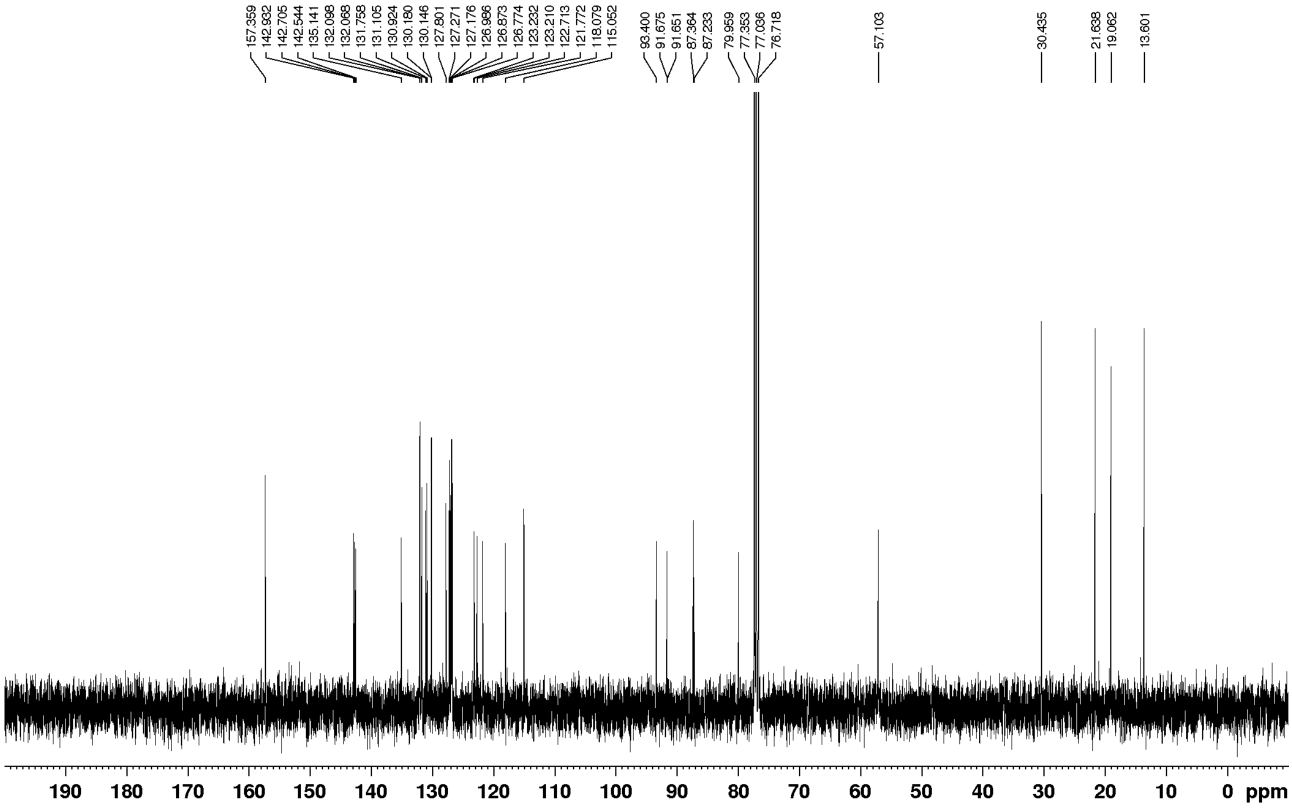

[2.2]Triphenylenophane 3a

^{1}H NMR ($CDCl_3$, 400 MHz)

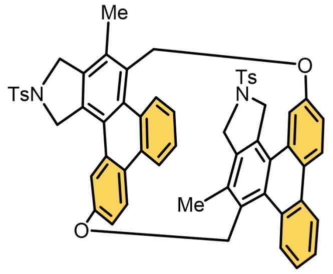

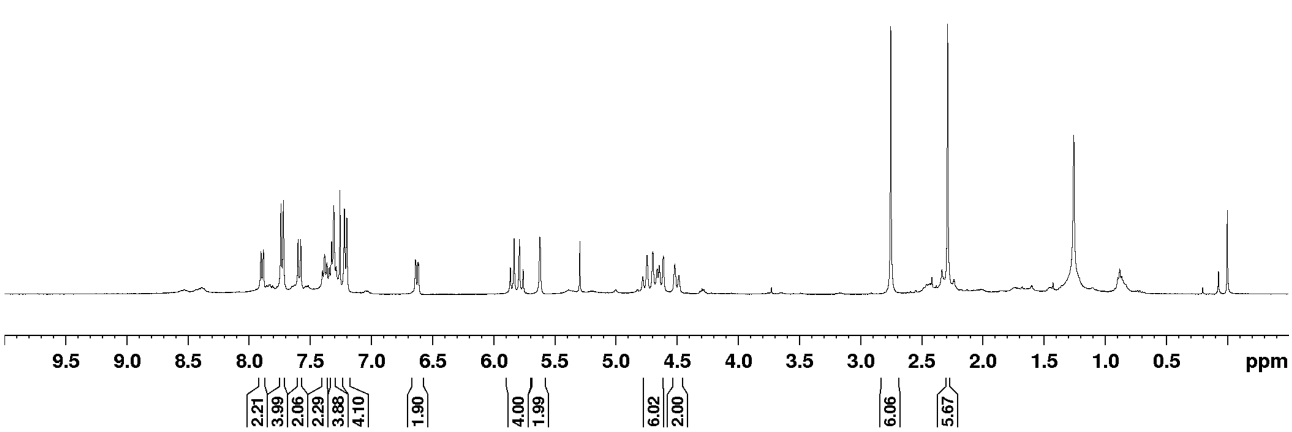

^{13}C NMR ($CDCl_3$, 100 MHz)

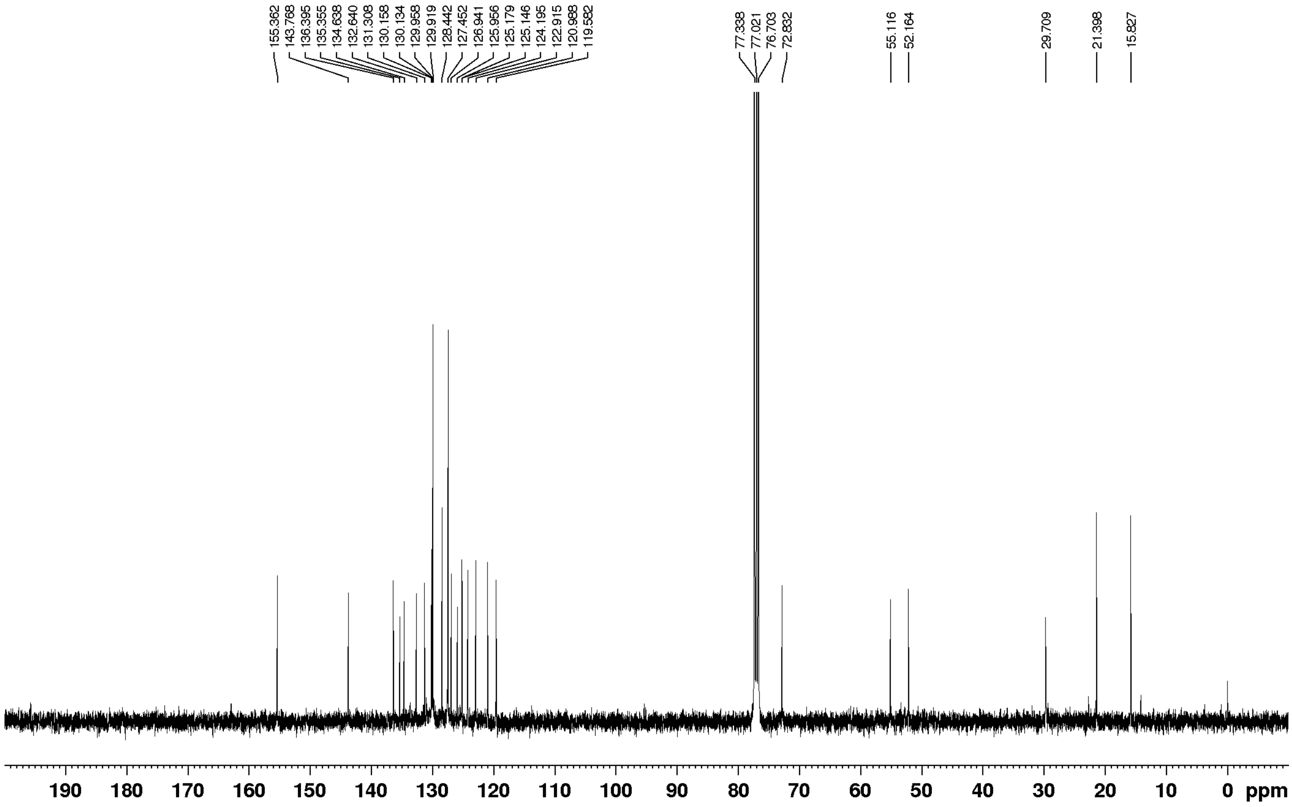

[2.2]Triphenylenophane 3b

^{1}H NMR ($CDCl_3$, 400 MHz)

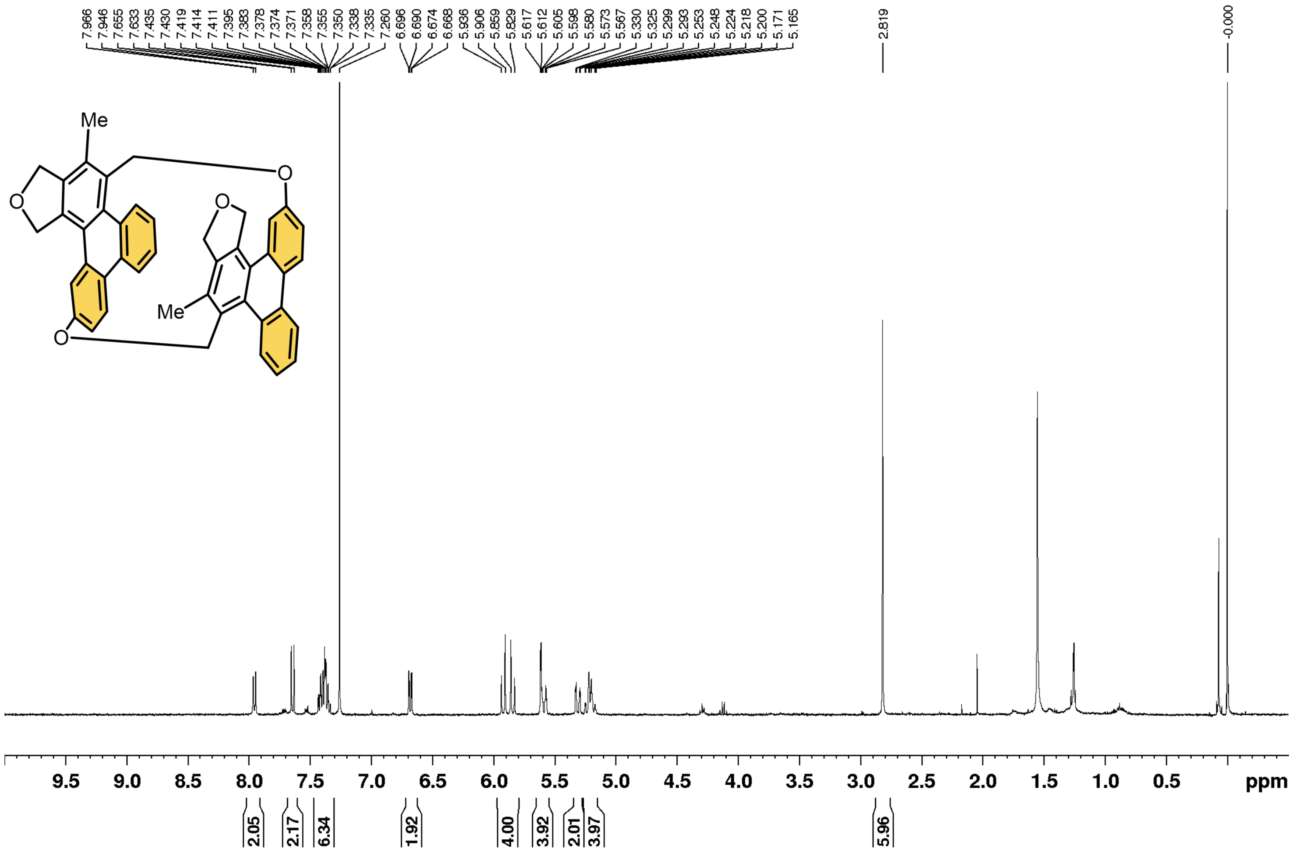

^{13}C NMR ($CDCl_3$, 100 MHz)

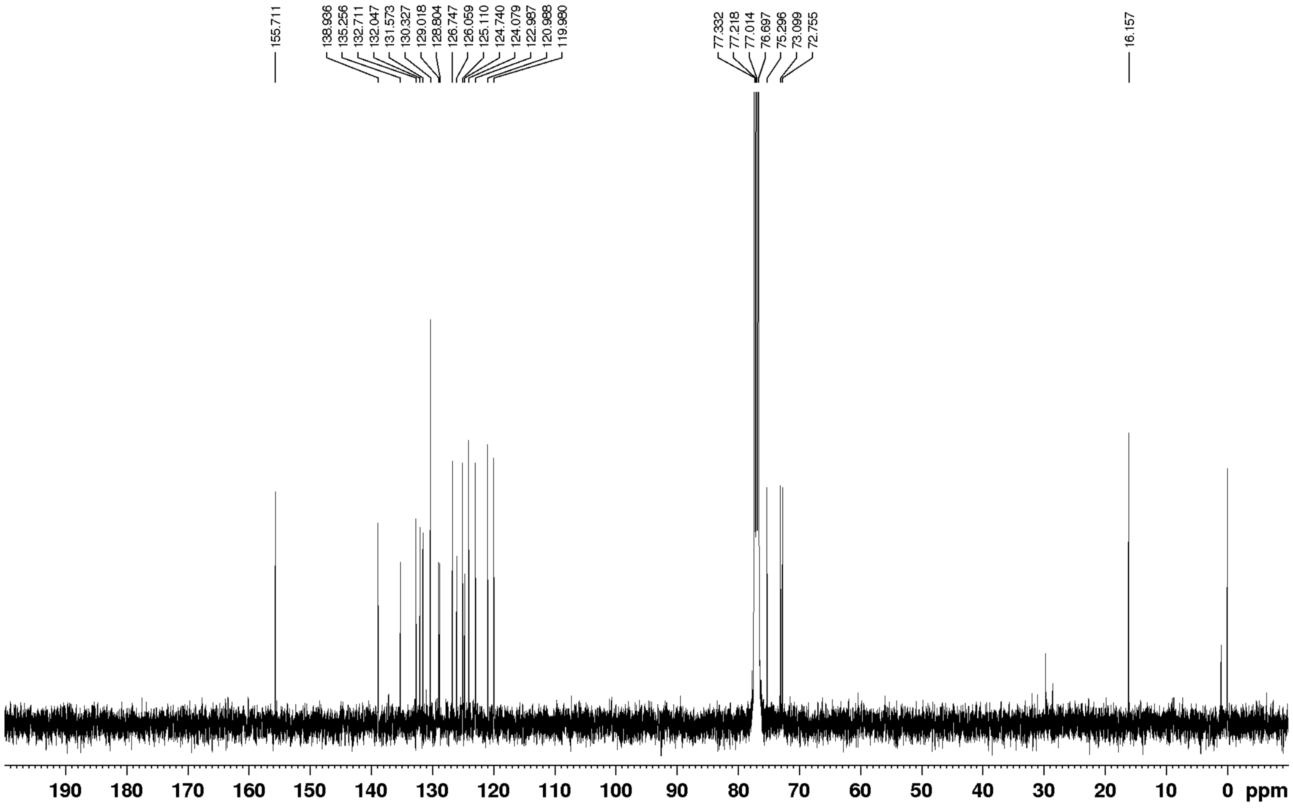

[2.2]Triphenylenophane 3c

^1H NMR ($CDCl_3$, 400 MHz)

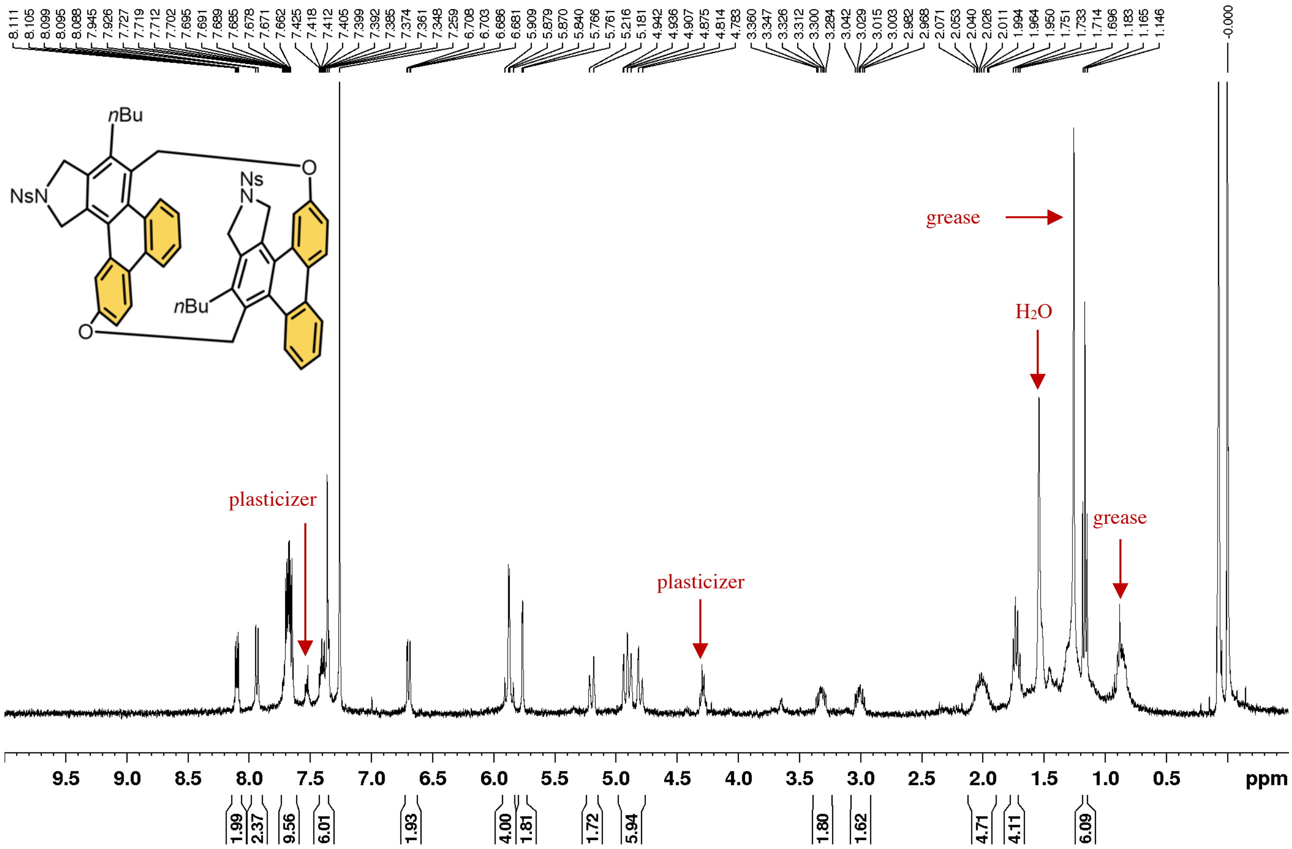

^{13}C NMR ($CDCl_3$, 100 MHz)

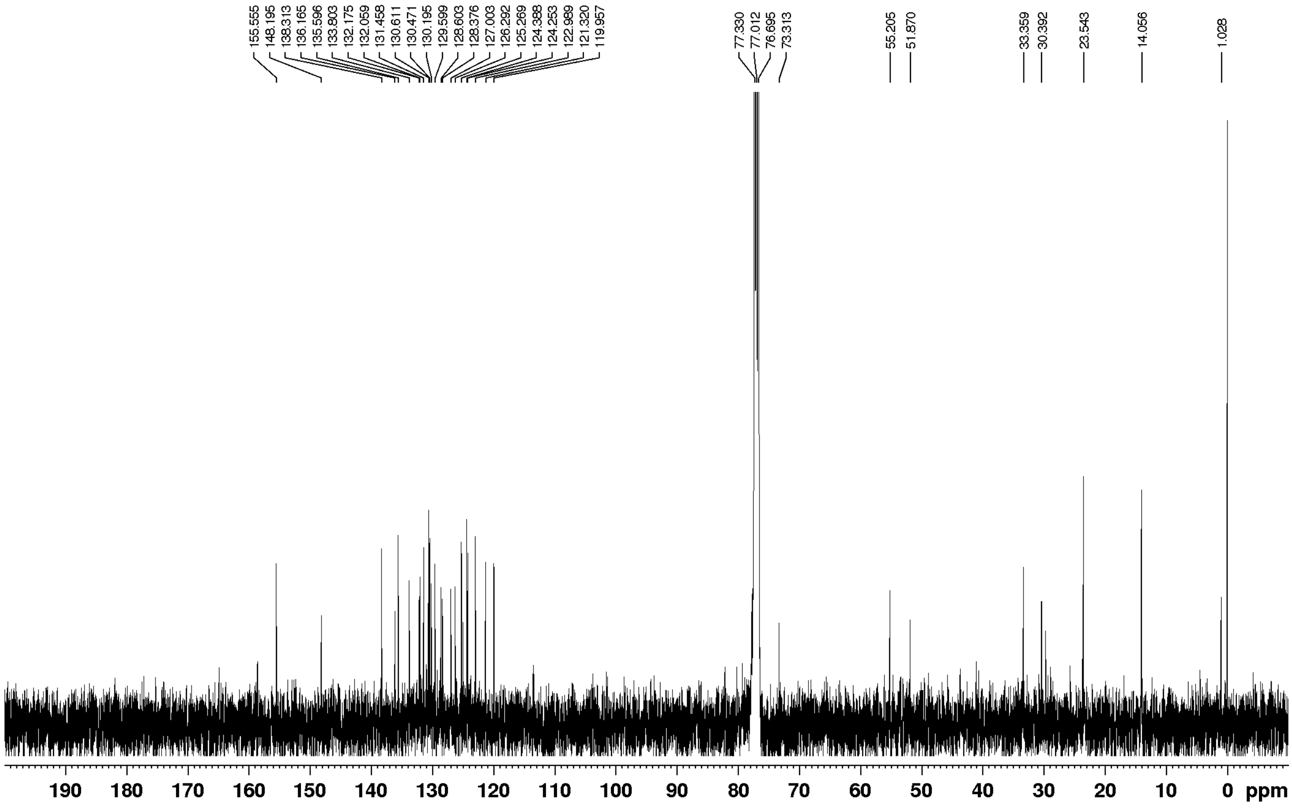

[2.2][5]Helicenophane 3e

^{1}H NMR ($CDCl_3$, 400 MHz)

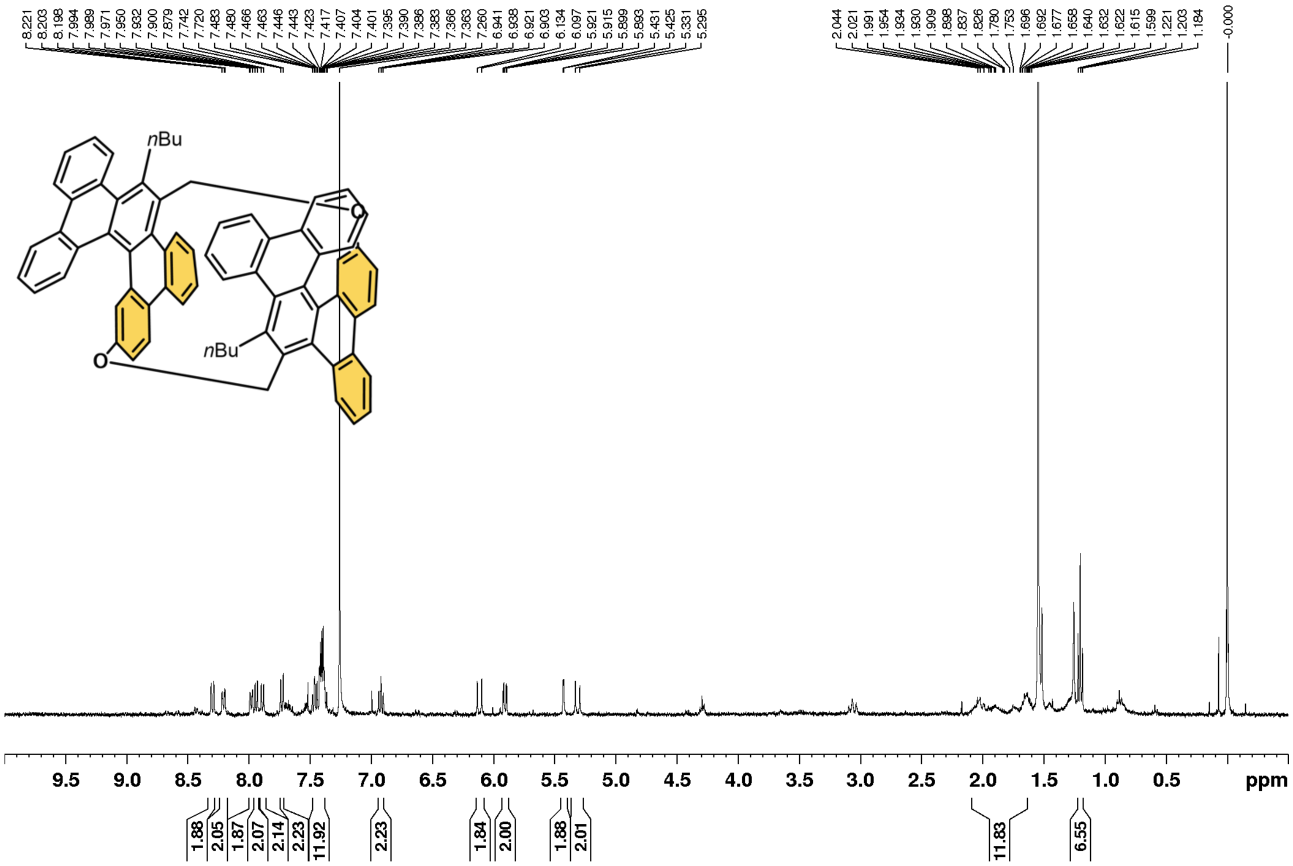

^{13}C NMR ($CDCl_3$, 100 MHz)

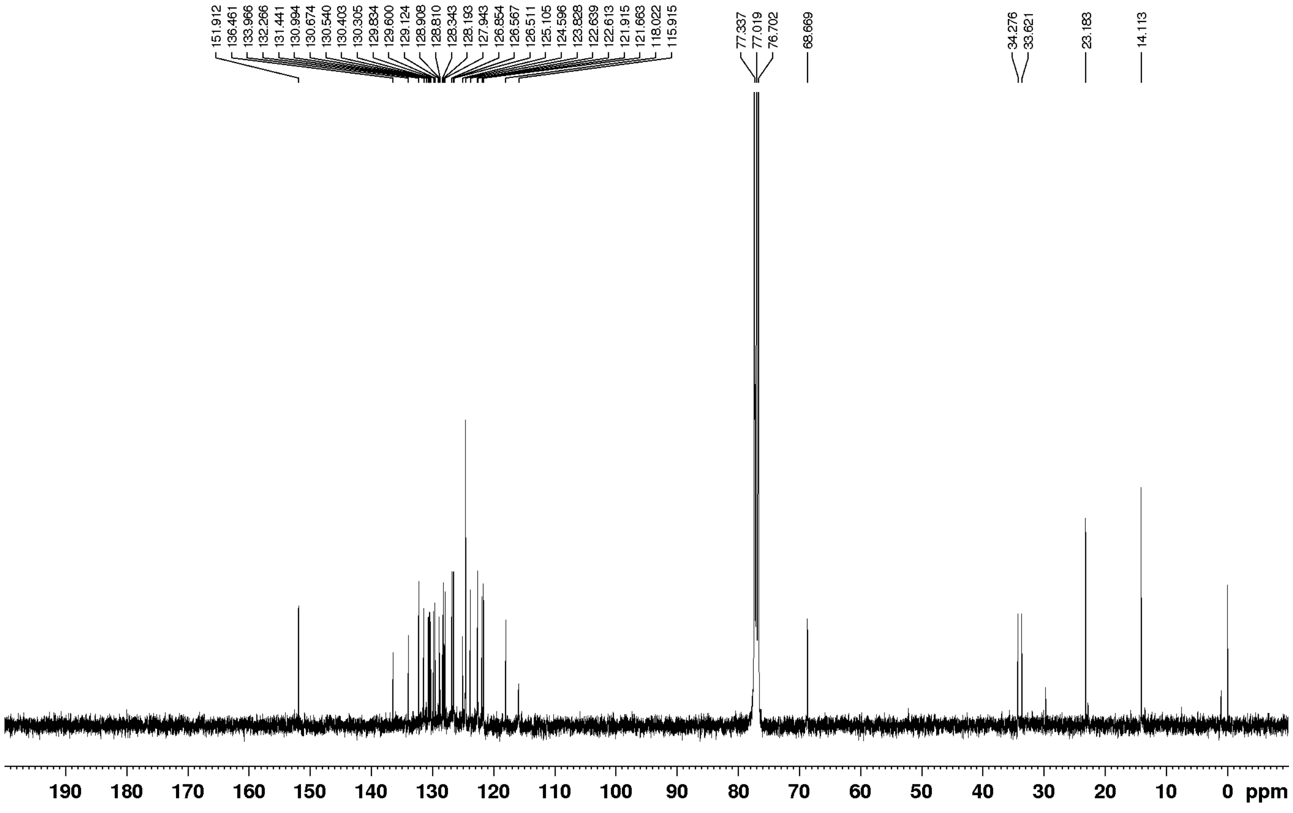

Supplement: SC-014-D3SC00571B-s001 [file SC-014-D3SC00571B-s001.pdf]
